# Supplementary material for: Advancing the safe motherhood initiative: A qualitative and sentiment analysis of local physician’s perspectives on antibiotic self-medication during pregnancy in a low- and middle-income country
Source: PLOS Glob Public Health. 2025 Sep 12;5(9):e0004794. doi: 10.1371/journal.pgph.0004794 (PMC12431270; doi:10.1371/journal.pgph.0004794)
Supplement: S1 File — Transcript 4 (CODES & THEMES by KU).pdf. Transcript 6 (CODES & THEMES by KU).pdf. Transcript 7 (CODES & THEMES, by KU).pdf. Transcript 8 (CODES & THEMES by KU).pdf. Transcript 9 (CODES & THEMES by KU).pdf. Transcript 10 (CODES & THEMES by KU).pdf. Transcript 11 (CODES & THEMES, by KU).pdf. Transcript 12 (CODES & THEMES by KU).pdf. Transcript 13 (CODES & THEMES by KU).pdf. Transcript 14 (CODED & THEMES by KU).pdf. Transcript 15_b (CODED & THEMES by KU). pdf. Transcript 16 (CODES & THEMES by KU).pdf. Transcript 17 (CODES & THEMES by KU).pdf. Transcript 18 (CODES & THEMES by KU).pdf. Transcript 19 (CODES & THEMES by HK).pdf. Transcript 20 (CODES & THEMES by HK).pdf. Transcript 21_b (CODES & THEMES by HK).pdfTranscript 22 (CODES & THEMES by HK).pdf. Transcript 25 (CODES & THEMES by HK).pdf. Transcript 27 (CODES & THEMES by HK).pdf. Transcript Sn1 (CODES & THEMES by RS).pdf Transcript Sn6 (pt3) (CODES & THEMES by RS).pdf. Transcript Sn15_a (CODES & THEMES by RS).pdf. Transcript SN17 (pt3) (CODES & THEMES by RS).pd. Transcript Sn21_a (CODES & THEMES by RS).pdf. (ZIP) [file pgph.0004794.s001.zip › Transcript 4 (CODES & THEMES by KU).pdf]

Text	Initial Codes	Themes
**Transcription interview 4**<br>**Interviewee: XXX (SN 15)**<br>**SN- 15**<br>**Interviewer: (MS), Research Assistant**<br>**Number of speakers :3**<br>**Other Attendees: (KU), Principal Investigator**<br>**Time: 10:55am UK time**<br>**Length of interview recording**<br>**Date: 17/3/23**<br>**Length of interview 24:35**<br><br>1. Interviewee [XXX]: Okay<br>**2. Interviewer [MS]: Okay perfect so did you get the consent form that I sent you and the information sheet?**<br>3. Interviewee [XXX]: erm yes I got the Whatsapp invitation to this meeting<br>**4. Interviewer [MS]: Mhmm, did you have a chance**<br>5. Interviewee [XXX]: *overlap*<br>**6. Interviewer [MS]: Okay**<br>**7.** Interviewee [XXX]: *unclear speech* some logistic issues, some challenges with network<br>**8. Interviewer [MS]: Okay okay. So did you have a chance to look through the information sheet that came through to you about the study?**<br>**9.** Interviewee [XXX]: Hello?<br>**10. Interviewer [MS]: Did you have a chance to read through the information sheet? It looked like this, ill show you**<br>11. Interviewee [XXX]: Okay I've not seen any information apart from the information asking me to join this meeting<br>**12. Interviewer [MS]: Okay so after this you can have a read, it looks like this one second**<br>13. Interviewee [XXX]: Hello<br>**14. Interviewer [MS]: Hi one second sorry**<br>15. Interviewee [XXX]: I said I have not seen any other information except for the one asking me to join this. Okay?<br>**16. Interviewer [MS]: * shared screen with PIS* Okay, that's perfect that's fine. So afterwards we have an information sheet you can read through erm which we will make sure Is emailed to you or sent on whatsapp and it will look like this urm and you can have a read through it okay? Or**		

**do you wanna have a read through it now?**<br>17. Interviewee [XXX]: Okay I can go through it later<br>**18. Interviewer [MS]: Okay, do you want to do it later? Yeah?**<br>19. Interviewee [XXX]: Okay<br>**20. Interviewer [MS]: Perfect and so then before we start I need to go through the consent form with you if that is okay and ill read the consent form, ill read each statement and then put your initials for you because you're unable to do it. Is that okay?**<br>21. Interviewee [XXX]: I didn't understand what you mean<br>**22. Interviewer [MS]: So I need to go through this consent form can you see it on the screen? *no reply* Can you see this on the screen?**<br>23. Principal Investigator [KU]: *participant name* Hello?<br>24. Interviewee [XXX]: Hello sorry there is poor network here I don't know<br>25. Principal Investigator [KU]: *name of interviewer* I think ethics say that he can give verbal consent so we don't necessarily need to go through the items, I will double check the wording er from ethics er but he just needs to be able to say look I consent to this urmm interview err<br>**26. Interviewer [MS]: Okay**<br>27. Principal Investigator [KU]: if *unclear speech* er so just ask him if he consents<br>**28. Interviewer [MS]: Okay**<br>29. Principal Investigator [KU]: and then<br>**30. Interviewer [MS]: Okay, perfect urm okay so if you cant see the form, do you consent to the interview, us going through this interview with you?**<br>31. Interviewee [XXX]: Hello?<br>**32. Interviewer [MS]: Hi I'm just asking, hi can you hear me?**<br>33. Interviewee [XXX]: Yes I can hear you now *unclear speech*<br>**34. Interviewer [MS]: Okay don't worry *overlapping speech***<br>**35.** Interviewee [XXX]: I can see I wasn't hearing what you were saying before but I can hear you now		

36. **Interviewer [MS]: Don't worry don't worry, um I just want to check just because the signals bad so its hard to go through this properly. Do you consent to us going through like doing this interview?**<br>37. *silence *<br>38. Interviewee [XXX]: Hello?<br>**39. Interviewer [MS]: *breathing***<br>40. Principal Investigator [KU]: Dr do you agree, do you agree to take part in the interview?<br>41. Interviewee [XXX]: the interview?<br>42. Principal Investigator [KU]: Yes, do you agree to take part?<br>43. Interviewee [XXX]: Okay this an interview now going on?<br>44. Principal Investigator [KU]: Yes yes<br>45. Interviewee [XXX]: ah ha let me *unclear speech* of the interview, the aim<br>46. Principal Investigator [KU]: Yes but do you consent to take part *line breaking up* do you agree to take part<br>47. Interviewee [XXX]: To take part in the interview?<br>48. Principal Investigator [KU]: Yes<br>49. Interviewee [XXX]: Yes but then whats the focus of the interview, because I wasn't *unclear speech* when she was talking and then also the accent was a challenge<br>50. Principal Investigator [KU]: go on<br>**51. Interviewer [MS]: So the interview is about antibiotics in antenatal care, so that is what the interview is about**<br>52. Interviewee [XXX]: About what<br>**53. Interviewer [MS]: Antibiotics in antenatal pregnancy care**<br>54. Interviewee [XXX]: Okay okay yeah antibiotics in antenatal patients<br>**55. Interviewer [MS]: Yeah exactly urm and the information sheet I sent you has all the information on there, so its just some questions so we just need to consent and youre happy to take part in the interview, before we start**<br>56. Interviewee [XXX]: yes Yes im ready<br>**57. Interviewer [MS]: Yes okay and youre happy that im recording this**<br>58. Interviewee [XXX]: Im okay<br>**59. Interviewer [MS]: yeah and you can stop at anytime you don't need to answer any of the questions you don't want to, if you don't want to continue with the interview**		

**then we can just stop you just need to let me know**		
60. Interviewee [XXX]: not quite clear with the accent *unclear speech* I didn't quite hear the last thing you said		
**61. Interviewer [MS]: pardon**		
62. Interviewee [XXX]: I didn't hear the last thing you said		
**63. Interviewer [MS]: Okay. If you want to stop the interview or if you don't want to answer any of the questions, you don't have to**		
64. Interviewee [XXX]: I don't have to answer any of the questions		
**65. Interviewer [MS]: if you don't want to, if you want to stop the interview you can stop the interview you just have to let me know**		
66. Interviewee [XXX]: Okay		
**67. Interviewer [MS]: Okay. *name of PI* is that okay or do you want me to ask anymore?**		
68. Principal Investigator [KU]: yes no carry on signal is so poor, so were quite lucky to get him eh so carry on with the questions		
69. **Interviewer [MS]: Should we just start the interview then?**		
70. Principal Investigator [KU]: Yes, yes		
71. **Interviewer [MS]: Yeah, that's okay with consent?**		
72. Principal Investigator [KU]: Yes		
**73. Interviewer [MS]: Okay, okay so were going to start the interview now, let me just**		
74. Interviewee [XXX]: Okay		
**75. Interviewer [MS]: Here we go**		
76. Interviewee [XXX]: *background noise*		
**77. Interviewer [MS]: So, do you prescribe antibiotics to pregnant women?**		
**78.** Interviewee [XXX]: Yes I do and *unclear speech* I give yes	**[78] Prescribe antibiotics**	**[1] PRESCRIBING**
**79. Interviewer [MS]: Okay. How long have you been prescribing antibiotics for?**		
**80.** Interviewee [XXX]: emm as long as I can remember, since I graduated *unclear speech*, over over 15 years now	**[80] Prescribe antibiotics (duration, in years)**	
**81. Interviewer [MS]: Okay. So how many times *overlap speech background noise with participant*, Okay**		
82. Interviewee [XXX]: yeah		

**83. Interviewer [MS]: How many times a week do you prescribe antibiotics….. to pregnant women?**		
84. Interviewee [XXX]: ummm on average be about around 3 times in a week	**[84] Prescribe antibiotics (frequency)**	
**85. Interviewer [MS]: Okay**		
**86.** Interviewee [XXX]: On average emm		
**87. Interviewer [MS]: and what are the most common problems that you prescribe antibiotics for?**		
88. Interviewee [XXX]: Hello?		
**89. Interviewer [MS]: Hi**		
**90.** Interviewee [XXX]: Yes *overlap speech*		
**91. Interviewer [MS]: What are the most common, what are the most common problems that you prescribe antibiotics for?**		
92. Interviewee [XXX]: For women? Generally? Or for pregnant women?	**[92] Prescribe antibiotics (conditions prescribed for)**	**[1] PRESCRIBING**
**93. Interviewer [MS]: For pregnant women**		
**94.** Interviewee [XXX]: Okay ah most of them when they have infections	**[94] Prescribe antibiotics (conditions prescribed for - infections)**	
**95. Interviewer [MS]: mhmm**		
96. Interviewee [XXX]: infections of the genital tracts	**[96] Prescribe antibiotics (conditions prescribed for – genital tracts)**	
**97. Interviewer [MS]: mhmm**		
98. Interviewee [XXX]: or infections any other place okay? Outside the genital tracts that the man said that they should be given antibiotics	**[98] Prescribe antibiotics (conditions prescribed for)**	
**99. Interviewer [MS]: mhmm mhmm okay**		
**100.** Interviewee [XXX]: Yeah		
**101. Interviewer [MS]: Where do you, where do pregnant women normally get their antibiotics from?**		
102. Interviewee [XXX]: usually get it from the institution where I'm practising cause I work in the *unclear speech*, I work with the institution *unclear speech*, so when we prescribe and they have the antibiotics they get it from there. If they don't have it within the facility where I am working, then they normally go they go across to nearby pharmacy shops and procure them	**[102] Obtaining antibiotics (sources, nearby shops, pharmacy)**	**[2] OBTAINING**
**103. Interviewer [MS]: Okay, do you know any pregnant women who have taken antibiotics that haven't been prescribed for them?**		
104. Interviewee [XXX]: Can I get the question clearer?		

**105. Interviewer [MS]: do you know of any pregnant women who take antibiotics that haven't been prescribed for them by a doctor?**		
**106.** Interviewee [XXX]: Okay any women that has taken antibiotics not prescribed	**[106]. SM - taking non-prescribed antibiotics (self-medication)**	**[3] SELF-MEDICATION**
**107. Interviewer [MS]: Yeah**		
108. Interviewee [XXX]: Yes, there are several of them and occasionally we come reports that they take antibiotics *unclear speech*, antibiotics that were not prescribed by doctors, by any doctors	**[108]. SM- taking nonprescribed antibiotics**	
**109. Interviewer [MS]: Where do they normally get them from?**		
110. Interviewee [XXX]: Usually they get it across the counter, from chemist shops, from pharmacy shops some from traders in the market that stop and sell antibiotics	**[110]. SM – source of antibiotics (self-medication)**	
**111. Interviewer [MS]: mhmm okay. Do you know of any pregnant women who sometimes take herbal preparations or alternative medications instead of antibiotics?**		
112. Interviewee [XXX]: *unclear speech* a good number of them. There are patients that go to *unclear speech* rural area. Sometimes they will come tell you they *unclear speech* concoction	**112. Herbal self-medication**	**[4] HERBAL SELF-MEDICATION**
**113. Interviewer [MS]: mhmm**		
114. Interviewee [XXX]: for possible infection, for suspected possible infections	**114. Herbs used to treat Infection.**	
**115. Interviewer [MS]: mhmm. What kind of alternative medications do they use? Do you have any examples?**		
116. Interviewee [XXX]: Um they just have some local herbs that do not have *unclear speech* biological, I mean urm how do you call it now, that we don't know the name but the local villages where they get the herbal concoction, they have their name, some of those herbal concoctions or leaves *unclear speech* have not been studied to know their pathological names or their *unclear speech* names	**116. Herbal concoctions/leaves used.**	
**117. Interviewer [MS]: Okay thank you. Urm do you know of any methods that can identify self-medication of antibiotics in pregnant women? So when a woman has taken antibiotics by themselves, not prescribed is there anyway to detect that?**		

118. Interviewee [XXX]: Any way to detect?<br>**119. Interviewer [MS]: If a woman has taken antibiotics without a prescription?**<br>120. Interviewee [XXX]: If a woman is taking antibiotics, I didn't understand the question sorry<br>**121. Interviewer [MS]: Is there anyway you know that just say if a woman came in and she's taken antibiotics by herself, self-medicated without a prescription, is there anyway for you to identity that? Like is there any method at the moment for you to identify that?**<br>122. Interviewee [XXX]: *unclear speech*, the only way I can identify that they are taking antibiotics on their own?<br>**123. Interviewer [MS]: Mhmm**<br>124. Interviewee [XXX]: Theres no way, maybe when they if they if they come and they are complaining of infection *unclear speech*, if you ask them if they are taking any medication outside *unclear speech* and some of them will own up and say yeah they did, or<br>**125. Interviewer [MS]: Mhmm**<br>126. Interviewee [XXX]: maybe when they have problems or some complications in their pregnancy we begin to wonder why. When we ask questions with regard to medications in the pregnancy sometimes they own up and divulge information otherwise theres no other way we can find out *unclear speech*<br>**127. Interviewer [MS]: So just by asking them?**<br>128. Interviewee [XXX]: Hello? You said?<br>**129. Interviewer [MS]: Yeah**<br>130. Interviewee [XXX]: Sorry<br>**131. Interviewer [MS]: So just when you ask them? Then you find out**<br>132. Interviewee [XXX]: Yeah I ask them when we ask them we find out<br>**133. Interviewer [MS]: Okay okay. Do you think it would be useful to have like a test or a tool or questionnaire that could you help identify pregnant women who might be misusing antibiotics without us knowing?**	**122. No way to detect self-medication.**<br><br>**124. Direct questioning**<br><br>**126. Direct questioning/unexplained complications**<br><br>**132. Direct questioning/finding out**	**[5] DETECTING**

134. Interviewee [XXX]: yeah we can have a questionnaire ask them, a good number of them will tell you the truth<br>**135. Interviewer [MS]: mhmm**<br>136. Interviewee [XXX]: especially when they know youre not going to use that against them<br>**137. Interviewer [MS]: Okay so if such a test was available, would you be interested in using it?**<br>138. Interviewee [XXX]: if eh hello?<br>139. **Interviewer [MS]: Would you be interested in using such a questionnaire if it was available?**<br>Interviewee [XXX]: Yes I would<br>**140. Interviewer [MS]: Okay urm and then do you think that a questionnaire or a tool could be used in antenatal care, during appointments, or in A&E? that kind of environment?**<br>141. Interviewee [XXX]: sorry can you come again<br>**142. Interviewer [MS]: Do you think such a tool or questionnaire could be used in antenatal care, could be used in antenatal settings or during appointments, or during A&E? Where do you think it could be used best?**<br>143. Interviewee [XXX]: Yeah I think I can be useful, can be useful *unclear speech* , subsequently *unclear speech*<br>144. **Interviewer [MS]: Okay. Do you think it would be useful for such a test to be like mobile or remote so you don't have to use electricity or internet to use it?**<br>145. Interviewee [XXX]: Sorry can you come again<br>**146. Interviewer [MS]: *clears throat*, do you think it would be useful for a questionnaire to test this to be kinda remote or mobile so you didn't need to use electricity or internet or power for it?**<br>147. Interviewee [XXX]: Im not sure I understand what you mean, sorry<br>**148. Interviewer [MS]: So just say if you had a test or a questionnaire or a tool to detect the antibiotic misuse in pregnant women, do you think it would be better if it was to be without electricity**	**134. Detecting self-medication (using a questionnaire)**<br><br>**136. Detecting - patient trust/suspicion**<br><br>**139. Detecting self-medication (questionnaire acceptability)**	**[5] DETECTING**

***participant starts to say something*, you wouldn't need to have electricity to use it?**<br>149. Interviewee [XXX]: *unclear speech*<br>**150. Interviewer [MS]: Mhmm**<br>151. Interviewee [XXX]: I don't understand the question, to detect how? Use of electricity?<br>**152. Interviewer [MS]: Without, without electricity so would it better?**<br>153. Interviewee [XXX]: Without using electricity?<br>**154. Interviewer [MS]: Yeah**<br>155. Interviewee [XXX]: To do what sorry?<br>**156. Interviewer [MS]: Like if you had a test, for the test?**<br>**157.** Interviewee [XXX]: test, like chemical analysis?<br><br>**Interviewer [MS]: Yes or a test or someway to detect antibiotic misuse in pregnant women. Would it be better if we had some test without electricity?**<br>158. Interviewee [XXX]: Okay *unclear speech*<br>**159. Interviewer [MS]: Yes**<br>160. Interviewee [XXX]: Chemical analysis to detect them<br>**161. Interviewer [MS]: Mhm**<br>162. Interviewee [XXX]: urmm it depends it depends on yeah if yeah it will not likely be better because eh it might be it might be *unclear speech* for the pregnant woman, they may not be able to afford it just to screen them *unclear speech* general screening antibiotics not prescribed by doctors, the cost may be a lot for them and they might not be able to afford that *unclear speech*, theres not a lot in the villages *unclear speech*, it might not be cost effective general screening using maybe a chemical analysis<br>**163. Interviewer [MS]: okay**<br>164. Interviewee [XXX]: from their blood or urine<br>**165. Interviewer [MS]: okay thank you**<br>166. Interviewee [XXX]:* unclear speech*<br>167. **Interviewer [MS]: yes thank you**	**157. Detecting self-medication (chemical analysis)**<br><br>**160. Detecting self-medication (barriers to chemical analysis)**<br><br>**162. Detecting SM - Chemical analysis (affordability)**	**[5] DETECTING**

**168. Interviewer [MS]: so have you come across any methods or guidelines which is about detecting like the side effects of antibiotic self-medication in pregnant women?**<br>169. Interviewee [XXX]: Do I have any what sorry?<br>**170. Interviewer [MS]: Guidelines that kind of help detect the side effects of antibiotic self medication in pregnant women**<br>171. Interviewee [XXX]: Okay not I don't have any for now<br>**172. Interviewer [MS]: Mhmm**<br>173. Interviewee [XXX]: Yes<br>174. **Interviewer [MS]: Okay... that's good to know. Um so as you know antibiotics can called can cause some side effects do you think that such side effects are kind of clear when a patients taking antibiotics?**<br>175. Interviewee [XXX]: *unclear speech*<br>**176. Interviewer [MS]: Yes**<br>**177. *overlap of speech***<br>**178. Interviewer [MS]: If someone had a reaction to antibiotics, is it clear? Is it obvious?**<br>179. Interviewee [XXX]: Whether its clear *distorted speech* sorry I'm not following, I don't understand the question.<br>**180. Interviewer [MS]: Is it obvious if you see someone, a pregnant woman that's having side effects to antibiotics? Is it obvious that there having side effects?**<br>181. Interviewee [XXX]: Okay its not always obvious *unclear speech* its not very obvious as such eh some of them may have the antibiotics *unclear speech* after delivery some may not be very obvious<br>**182. Interviewer [MS]: Mhmm**<br>183. Interviewee [XXX]: Okay<br>**184. Interviewer [MS]: Okay. Do you know any pregnant women who have had side effects of self-medication of antibiotics? So if they've taken it themselves without a prescription?**<br>185. Interviewee [XXX]: ummmm, eh none that I can remember now because they isn't aware of specifically em *unclear	**171. Self-medication/no guidelines**<br><br>**185. Side effects/self-medication**	**[6] GUIDELINES (1/2)**<br><br>**[7] SIDE EFFECTS**

speech*, the antibiotics that have caused the problem<br>**186. Interviewer [MS]: Mhmm**<br>187. Interviewee [XXX]: em theres some women actually that we know that use drugs in early pregnancy and they have malformed fetuses babies delivered preterm or sometimes they have miscarriage and then some obvious abnormality<br>**188. Interviewer [MS]: Mhmm *overlap***<br>189. Interviewee [XXX]: but then its difficult to pin down on the real course of this is result of antibiotics<br>**190. Interviewer [MS]: Mhmm yep okay. Do you know of any guidelines to manage antibiotic self medication in pregnant women?**<br>**191.** Interviewee [XXX]: no I don't know any sorry<br>**192. Interviewer [MS]: That's fine thank you and then the last question so with pregnant women who have self-medicated with antibiotics, sometimes have memory loss or forgetfulness have you ever come across that, would you know how to manage that?**<br>193. Interviewee [XXX]: Can you come again with the question sorry *signal not very clear*<br>**194. Interviewer [MS]: so so if some pregnant women if they take like their antibiotics without them being prescribed, sometimes they might get memory loss or forgetfullness, have you ever come across that, do you know how to manage that, have you ever seen that?**<br>195. Interviewee [XXX]: eh not come across that in my practice<br>196. **Interviewer [MS]: Okay, okay, that's good to know perfect thank you, that is all the questions em so thank you very much for taking part, I know its difficult with the signal em I just wanted to check, do you have an airtime card?**<br>197. Interviewee [XXX]: airtime<br>**198. Interviewer [MS]: yeah**<br>199. Interviewee [XXX]: *unclear speech*	**187. Side effects/self-medication/abnormalities**<br><br>**189. Side effects/uncertainty**<br><br>**191. No guidelines**	**[6] GUIDELINES (2/2)**

200. Interviewer [MS]: no?

201. Interviewee [XXX]: hello?

202. Interviewer [MS]: hi

203. Interviewee [XXX]: I don't understand the question sorry

204. Interviewer [MS]: have you bought an airtime card?

205. Interviewee [XXX]: I have got one

206. Interviewer [MS]: yeah so you can submit *name of a doctor* for a refund for taking part after the interview

207. Interviewee [XXX]: okay *name of a doctor*

208. Interviewer [MS]: yeah

209. Interviewee [XXX]: *unclear overlapping speech*

210. Interviewer [MS]: so you can submit forward the airtime card to him and he will be able to give you a refund

211. Interviewee [XXX]: okay so I can send some airtime to *name of a doctor*

212. Interviewer [MS]: * started to speak then stopped*

213. PI [KU]: So *name of participant* shes referring to the airtime eh that you use for this interview. Did you buy a card for this interview?

214. Interviewee [XXX]: okay yes I did

215. PI [KU]: okay so you could send that card to *name of a doctor*

216. Interviewee [XXX]: okay the card?

217.
Interviewer [MS]: yeah

218. PI [KU]: yeah, the airtime card

219. Interviewee [XXX]: I already I usually buy online

220. PI [KU]: okay have you got a receipt?

221.
Interviewee [XXX]: maybe I will check if they sent me a voucher I've not deleted. I usually delete when they send message with it I usually delete, maybe I will check *some unclear speech*

222. PI [KU]: okay

223. Interviewer [MS]: okay

224.
Interviewee [XXX]: voucher *unclear speech*

225. **Interviewer [MS]: okay great. Um okay so do you have any questions?**

Interviewee [XXX]: hello?<br>**Interviewer [MS]: hi do you have any questions?**<br><br>Interviewee [XXX]: okay yes the question I have, maam can I you said you sent me something which I wasn't able to read for this interview<br>**226. Interviewer [MS]: mhmm**<br>227. Interviewee [XXX]: so then I will still read it, I will still go through the message you sent me, was it send through whatsapp or through my mail, email<br>**228. Interviewer [MS]: both it should be both, your whatsapp and your email**<br>229. Interviewee [XXX]: okay so maybe when I go through it I will understand the whole essence of this exercise. Otherwise if you can just give me briefly the essence of the exercise and so on, if further things are expected from me in future<br>**230. Interviewer [MS]: mhmm**<br>**231.** PI [KU]: *name of interviewer* should I take , should I speak about it?<br>**232. Interviewer [MS]: yeah sure *puts PIS back on screen***<br>233. PI [KU]: *name of participant*, this is *name of Principal investigator*, eh so this project its just to gain a better understanding of the problem of antibiotic misuse eh during pregnancy and eh it's a collaboration between *name of university* and *name of hospital*<br>234. Interviewee [XXX]: yeah<br>235. PI [KU]: we've had liaisons with *name of doctor* and *name of doctor* and some other consultants at *name of hospital* and they've given permission for us to interview eh you and some of your colleagues on this topic<br>236. Interviewee [XXX]: okay<br>237. PI [KU]: so<br>238. *interrupted sound*<br>239. PI [KU]: eh like *name of interviewer* said she sent you some useful information via email, so you can check the information if you have any questions please feel free to text me or email me or emm *name of interviewer* eh and we will be happy to get back to you		

240. Interviewee [XXX]: okay thank you very much and will there be a publication on this?<br>241. PI [KU]: yes<br>242. Interviewee [XXX]: *unclear speech*<br>243. PI [KU]: Yes, we will keep you informed on that once eh we've got all the data together<br>244. Interviewee [XXX]: okay, its alright, thank you very much for the opportunity and em we hope we'll be part of the project of *unclear speech*<br>245. PI [KU]: thank you wonderful<br>246. **Interviewer [MS]: thank you, thank you if you have any if you have any problems accessing the information or you can't find it, just email me or *name of Principal Investigator*and I will send it you again, or whatsapp him**<br>247. Interviewee [XXX]: alright thank you very much<br>**248. Interviewer [MS]: okay no problem thank you so much**<br>249. Interviewee [XXX]: okay nice time *unclear speech*<br>**250. Interviewer [MS]: bye have a good day**<br>251. Interviewee [XXX]: okay and you too<br>**252. Interviewer [MS]: bye**<br>253. *other voices saying bye*<br>254. Interviewee [XXX]: Bye *name of PI*<br>255. PI [KU]: Bye *name of participant*<br>256. Interviewee [XXX]: Bye<br>**257. Interviewer [MS]:*mumbles***<br>258. PI [KU]: Eh well done *name of interviewer* the *end of recording*		
