# Supplementary material for: Advancing the safe motherhood initiative: A qualitative and sentiment analysis of local physician’s perspectives on antibiotic self-medication during pregnancy in a low- and middle-income country
Source: PLOS Glob Public Health. 2025 Sep 12;5(9):e0004794. doi: 10.1371/journal.pgph.0004794 (PMC12431270; doi:10.1371/journal.pgph.0004794)
Supplement: S1 File — Transcript 4 (CODES & THEMES by KU).pdf. Transcript 6 (CODES & THEMES by KU).pdf. Transcript 7 (CODES & THEMES, by KU).pdf. Transcript 8 (CODES & THEMES by KU).pdf. Transcript 9 (CODES & THEMES by KU).pdf. Transcript 10 (CODES & THEMES by KU).pdf. Transcript 11 (CODES & THEMES, by KU).pdf. Transcript 12 (CODES & THEMES by KU).pdf. Transcript 13 (CODES & THEMES by KU).pdf. Transcript 14 (CODED & THEMES by KU).pdf. Transcript 15_b (CODED & THEMES by KU). pdf. Transcript 16 (CODES & THEMES by KU).pdf. Transcript 17 (CODES & THEMES by KU).pdf. Transcript 18 (CODES & THEMES by KU).pdf. Transcript 19 (CODES & THEMES by HK).pdf. Transcript 20 (CODES & THEMES by HK).pdf. Transcript 21_b (CODES & THEMES by HK).pdfTranscript 22 (CODES & THEMES by HK).pdf. Transcript 25 (CODES & THEMES by HK).pdf. Transcript 27 (CODES & THEMES by HK).pdf. Transcript Sn1 (CODES & THEMES by RS).pdf Transcript Sn6 (pt3) (CODES & THEMES by RS).pdf. Transcript Sn15_a (CODES & THEMES by RS).pdf. Transcript SN17 (pt3) (CODES & THEMES by RS).pd. Transcript Sn21_a (CODES & THEMES by RS).pdf. (ZIP) [file pgph.0004794.s001.zip › Transcript 6 (CODES & THEMES by KU).pdf]

Text	Initial Codes	Themes
**Transcription interview 6**<br>**Interviewee: XXX**<br>SN- 28<br>**Interviewer: MS, Research Assistant**<br>**Number of speakers :2**<br>**Time: 1600**<br>**Length of interview recording: 16 minutes**<br>**Date: 17th March 2023**<br><br>1. **Interviewer [MS]: So I sent you on the email and I think *name of Principal Investigator* sent you on the email the participant information sheet and the consent form, um you said you had some questions about the consent form, what was that?**<br>2. Interviewee [XXX]: yeah yeah exactly, it was about the eh where it was written please initial, I mean I wasn't sure<br>3. **Interviewer [MS]: *overlap* oh okay**<br>4. Interviewee [XXX]: *overlap* yeah whether I was going to note anything there in that column, I wasn't sure, so I just wanted to ask question and clarify<br>5. **Interviewer [MS]: So what weve been doing just because its been difficult obviously getting like sending it to people and getting it back, when weve come on the zoom call weve found it quite like useful I can share the screen and if you're happy I can initial it for you we can go through it and then we can do it that way. Or you can initial it all and then send it back to me but then we would have to do that before we start**<br>6. Interviewee [XXX]: mm I ge understand that's fine lets do it your way that's fine<br>7. **Interviewer [MS]: okay *laugh* kay, so did you have a chance to read through the participant information sheet?**<br>8. **Interviewer [MS]: *silence* Hello? Hello? *clears throat* Hello?**<br>9. **Interviewer [MS]: Hello? He has gone out, signal must have gone.**<br><br>10. **Interviewer [MS]: kay**<br>11. ***background noise***<br>12. **Interviewer [MS]: Hi did the signal go?**<br>13. Interviewee [XXX]: *overlapping speech*<br>14. **Interviewer [MS]: don't worry**<br>15. Interviewee [XXX]: *unclear overlapping speech* my wifi<br>16. **Interviewer [MS]:ahh don't worry**		

17. Interviewee [XXX]: im so sorry about it *overlapping speech*<br>**18. Interviewer [MS]: ah don't worry, honestly were used to it don't worry don't worry urm okay so im just going to share my screen with yo, so did you get the did you read the participant information sheet?**<br>19. Interviewee [XXX]: Yeah I did it was clear<br>**20. Interviewer [MS]: This one *overlapping speech* perfect so you have, ive started the recording again so you've had a read through this *showing participant information sheet on screen* participant information sheet**<br>21. Interviewee [XXX]: yeah I did I have *overlapping speech*<br>**22. Interviewer [MS]: perfect**<br>23. Interviewee [XXX]: I read it was very clear *overlapping speech*<br>**24. Interviewer [MS]: perfect and then so this is a consent form can you see it *shared screen with consent form***<br>25. Interviewee [XXX]: I can see it<br>**26. Interviewer [MS]: perfect so ive just put the information sheet date there um so then did you have a read through the consent form? Do you consent to all of the points? Are you happy with it all?**<br>27. Interviewee [XXX]: yes I do<br>**28. Interviewer [MS]: Yes! Okay perfect *overlap* so you've read it all and you consent to all of it, is that your correct initials? Yeh?**<br>29. Interviewee [XXX]: that's right<br>**30. Interviewer [MS]: so youre happy for me to video record and audio record it**<br>31. Interviewee [XXX]: Its fine its fine its okay *overlap*<br>**32. Interviewer [MS]: The whole thing the whole thing *overlapping unclear speech* you just didn't know what to do with the signing that's fine, so then we write your name here perfect, so you consent to taking part that's the main bit isnt it**<br>33. Interviewee [XXX]: mm yeah<br>**34. Interviewer [MS]: Right I'm just gonna, so your initials are *says initials* and I'm just going to sign this great yeah if you're happy with that then that's perfect great perfect so you consent so we can do the questionnaire now**<br>35. Interviewee [XXX]: *slight over lapping speech intermittently* mm<br>**36. Interviewer [MS]: okay perfect so ive just got some questions first of all, urm do you have an airtime card that you're using for this call?**		

37. Interviewee [XXX]: yeah Im actually using my wifi at home so<br>**38. Interviewer [MS]: okay**<br>39. Interviewee [XXX]: mm<br>**40. Interviewer [MS]: okay fine its just if you had an airtime card you were using we would say you could submit it to *name of a doctor* for a refund but you're using your wifi so**<br>41. Interviewee [XXX]: yeah<br>**42. Interviewer [MS]: okay**<br>43. Interviewee [XXX]: yeah<br>**44. Interviewer [MS]: yeah okay perfect right so ive just got some questions, if you've got any questions about the questions then just let me know so do you prescribe antibiotics to pregnant women?**<br>45. Interviewee [XXX]: yes I do<br>**46. Interviewer [MS]: Yes, How long have you been prescribing them to women for?**<br>47. Interviewee [XXX]: I've been doing that for the past ive been practising for 14 years,so ive been doing that for 14 years<br>48. **Interviewer [MS]: okay amazing and then how many times a week do you prescribe antibiotics to pregnant women?**<br>49. Interviewee [XXX]: eh that will be on the average about once a week, because I run antenatal I run antenatal services for once a week atleast so, ill say on the average once a week<br>**50. Interviewer [MS]: okay and what are the 3 most common medical problems that you prescribe antibiotics for?**<br>51. Interviewee [XXX]: the commonest will be upper respiratory tract infections<br>52. **Interviewer [MS]: mhmm, is there any other things, any other conditions?**<br>53. Interviewee [XXX]: I will say usually pharyngitis sometimes, and eh sometimes they actually have eh I will just say pharyngitis<br>**54. Interviewer [MS]: okay okay, so that's like the most common.**<br>55. Interviewee [XXX]: yes<br>**56. Interviewer [MS]: Perfect and do you have any guidance that you use when prescribing antibiotics, any guidelines?**<br>57. Interviewee [XXX]: oh yeah the guidelines is vague, because eh we don't have any clear cut microbiological or antibiotic protocol so<br>**58. Interviewer [MS]: mhmm *overlap***	47. Prescribing antibiotics (years)<br>49. Prescribing antibiotics (freq)<br><br>51. Prescribing antibiotics (respiratory infections)<br>53. Prescribing (other)<br><br>57/59. Vague guidelines on antibiotic prescribing (microbiology)<br><br>61. Self-medication with antibiotics<br><br>63. SM (sometimes)<br>65. SM (at home or OTC)	[1] Prescribing antibiotics (freq, guidelines)<br><br>[3] Self-medication (frequency, source)

59. Interviewee [XXX]: The guide will just be em based on clinical *unclear speech* and based on eh urm results of microbiology<br>**60. Interviewer [MS]: Okay so where do find that pregnant women really get antibiotics from?**<br>61. Interviewee [XXX]: well they get from the hospital when the prescriptions made at the hospital, they pick it up from hospital pharmacy<br>62. **Interviewer [MS]: mhmm, are you aware of any like pregnant women who have taken antibiotics that havent been prescribed for them?**<br>63. Interviewee [XXX]: im sure they do yeah sometimes<br>**64. Interviewer [MS]: *overlapping speech***<br>65. Interviewee [XXX]: *unclear speech* before they get in hospital *unclear speech* they are already taking some at home *unclear speech* or they get to have some over the counter antibiotics here in Nigeria and they do actually have them sometimes from *unclear speech* hospital<br>66. **Interviewer [MS]: okay**<br>**67. *unclear speech***<br>**68. Interviewer [MS]: mm**<br>**69. Interviewer [MS]: Are you aware of any pregnant women who might take like herbal preparations or alternative medications instead of antibiotics?**<br>70. Interviewee [XXX]: that's very rare *unclear speech* very rarely<br>**71. Interviewer [MS]: do you have any examples of any that you've seen before *pause* or heard about?**<br>72. Interviewee [XXX]: Urm I would have heard about it you know but they don't commonly take it when there pregnant, they get take a lot of herbs when there not pregnant but I don't *unclear speech*, a pregnant woman taking herbal preparations *unclear speech*<br>**73. Interviewer [MS]: Okay okay. Do you know of any methods that can detect or identify if women did self-medicate antibiotics in pregnancy?**<br>74. Interviewee [XXX]: No I don't<br>**75. Interviewer [MS]: Okay. Do you think it would could be useful to have like a simple rapid test or Labatory experiment or tool or questionnaire that could help identify pregnant women who might be misusing antibiotics without us knowing?**<br>76. Interviewee [XXX]: Yes I do<br>**77. Interviewer [MS]: What do you think would be useful? Most?**<br>78. Interviewee [XXX]: Well I think a simple structured questionnaire would be useful	70. Herbal use (uncertainty, rarity of use)<br>71. Herbal use (not in pregnancy)<br><br>74/76. Detecting antibiotic misuse<br><br>78. Detection (questionnaire)	[4] Herbal self-medication (rarity, during preg)<br><br>[5] Detecting self-medication (methods e.g., rapid test, questionnaire, setting, communication)

**79. Interviewer [MS]: Mhmm, would you be interested in using something like that?**<br>80. Interviewee [XXX]: Yes I would be<br>**81. Interviewer [MS]: Okay and just say they developed a tool within antenatal care settings do you think it would be useful with antenatal care settings or in routine appointments or in A&E? What kind of environment would it be most useful in?**<br>82. Interviewee [XXX]: well I think it would be most useful in routine antenatal care<br>**83. Interviewer [MS]: mhmm**<br>84. Interviewee [XXX]: *overlap* I think it would be *unclear speech*, also put in a&e,<br>**85. Interviewer [MS]: mhmm**<br>86. Interviewee [XXX]: a&e is abit *unclear speech* that presents to a&e they may not be able to talk, may not be in a position to really express themselves so it sometimes it can be difficult to get correct answer<br>**87. Interviewer [MS]: mmm *overlap***<br>88. Interviewee [XXX]: To the question, but I think most women that come to antenatal care they actually speak for themselves so that's where I think its most suitable<br>**89. Interviewer [MS]: mmm**<br>**90. Interviewer [MS]: And do you think it would be useful for such a test to be remote, to use without internet, mobile?**<br>91. Interviewee [XXX]: mmmm *unclear speech* yeah it can be used remotely, I believe it can be used remotely<br>92. **Interviewer [MS]: mm okay yeah I mean were just talking if there was something what would be most useful**<br>93. Interviewee [XXX]: Yeah yeah but eh yeah even though we do *unclear speech* here in Nigeria, its not that you know common place as you know in the Uk or Europe, a lot of women with antenatal care actually present in hospital physical pregnant so remotely its possible its possible<br>94. **Interviewer [MS]: Have you come across any methods or guidelines which help detect side effects of antibiotic self-medication in pregnant women?**<br>95. Interviewee [XXX]: *unclear speech*<br>**96. Interviewer [MS]: and obviously we know antibiotics can cause side effects like stomach upset,rash, do you think the presence of such side effects in a patient is clear when there its from antibiotics?**<br>97. Interviewee [XXX]: yeah some is clear like a rash um *unclear speech* causing a rash can actually be obvious	82. Detection (antenatal care settings)<br><br>86. Detection (problems with communication)<br>88. Detection (verbal)<br><br>91. Detection (remote areas)<br><br>93. Detection (hospital)<br><br>97. Side effects fr. Antibiotic SM (other possibilities)<br><br>99. Side effects (conflict patient stories vs histories)	[7] Detecting self-medication (side effects as indicators)

but apart from that the symptoms might be linked to something else<br>98. **Interviewer [MS]: mmm mm**<br>99. Interviewee [XXX]: ehh but I think its possible *unclear speech*, the symptom that doesn't present with the story the history can give a clue can give a clue whatever is coming from antibiotics<br>**100. Interviewer [MS]: mhmm mhmm**<br>**101. Interviewer [MS]: Do you know any pregnant women suspected to have developed side effects of self-medication with antibiotics? When they've not been prescribed they've just self medicated**<br>102. Interviewee [XXX]: oh yes<br>**103. Interviewer [MS]:mhmm so you've seen that?**<br>104. Interviewee [XXX]: Yes yes ive seen that. The woman was having a lot of nausea from taking antibiotics that wasn't prescribed.<br>**105. Interviewer [MS]: mhmm mhmm**<br>**106. Interviewer [MS]: and are there any methods or guidelines or protocols that could manage self medication of antibiotics in pregnant women that you know of?**<br>107. Interviewee [XXX]: No<br>**108. Interviewer [MS]: no okay urm and then weve just got a specific area of pregnant women who have self medicated with antibiotics and developed signs of memory loss or forgetfulness , do you know of any management options that would take place if that happened?**<br>109. Interviewee [XXX]: not that I would think of right now<br>**110. Interviewer [MS]: okay perfect, fab, do you have anything else to add about misuse of antibiotics in antenatal care or you know women taking antibiotics that arent prescribed? Do you have anything else to add?**<br>111. Interviewee [XXX]: I will say that an in an environment where we cannot *unclear speech* in Nigeria because there are mindset that whenever you are ill you have to require antibiotics for treatment, that mindset that misconception that women have here, so whenever we have opportunity we talk to women try to get them to understand that you don't have to take antibiotics on your own or get them over the counter, sometimes people will even get antibiotics sent from abroad some of these women<br>**112. Interviewer [MS]: mmmm**	102/104. Developed side effects from antibiotics<br><br>107. Lack of clear guidelines on self-medication<br><br>109. Neurological side effects<br><br>111. Prevailing mindsets (require antibiotics for sickness; get them OTC)<br><br>113. Prevailing mindsets (headaches, fever)<br><br>115. Mindsets problematic	[6] Detecting self-medication (no guidelines)<br><br>[8] Cultural mindsets (on antibiotic SM)

113. Interviewee [XXX]: and they think that okay I have a headache, or have a fever and they open the bottle and taking the antibiotics. So it's the *unclear speech* and we are not happy about it. I think its about misconception<br>**114. Interviewer [MS]: mmmm *overlap***<br>115. Interviewee [XXX]: I don't want to say its traditional, its more misconception here and its actually a problem<br>**116. Interviewer [MS]: mmm that's really interesting and useful thank you**<br>117. Interviewee [XXX]: *overlapping speech*<br>**118. Interviewer [MS]: do you have any other questions or anything about the questions ive asked you or the interview?**<br>119. Interviewee [XXX]: so far so good<br>**120. Interviewer [MS]: okay amazing so that's all my questions its just a few questions that I needed to ask you, so that's all really. Thank you so much for your time I really appreciate you coming on after work *laugh***<br>121. Interviewee [XXX]: *overlap speech*, I thought it would be like 40 minutes, I didn't think it would be like 15 minutes<br>**122. Interviewer [MS]: It just depends, yeah, everythings different really, it just depends, but yeah no thank you very much that's very useful and we really appreciate it**<br>123. Interviewee [XXX]: *overlapping unclear speech*<br>**124. Interviewer [MS]: Have a good evening**<br>125. Interviewee [XXX]: alright you too, can I ask feedback, like whats next after this am I going to be able to get feedback, I guess its research so I want to know um by the time *unclear speech*, am I going to be able to access to<br>**126. Interviewer [MS]: Yeah so were hoping to like eventually hopefully write a report and disseminate a report, so when that happens we should be able to let everyone know that's taken part so youll be able to access the report hopefully**<br>127. Interviewee [XXX]: *background noise*<br>**128. Interviewer [MS]: but if you have any other questions you've got my email um you've got the information sheet so that is all done, so if you've got any other questions just let me know**<br>129. Interviewee [XXX]: I'll do so		

**130. Interviewer [MS]: Have a lovely weekend and thank you so much**<br>131. Interviewee [XXX]: Alright bye		
