# Supplementary material for: Advancing the safe motherhood initiative: A qualitative and sentiment analysis of local physician’s perspectives on antibiotic self-medication during pregnancy in a low- and middle-income country
Source: PLOS Glob Public Health. 2025 Sep 12;5(9):e0004794. doi: 10.1371/journal.pgph.0004794 (PMC12431270; doi:10.1371/journal.pgph.0004794)
Supplement: S1 File — Transcript 4 (CODES & THEMES by KU).pdf. Transcript 6 (CODES & THEMES by KU).pdf. Transcript 7 (CODES & THEMES, by KU).pdf. Transcript 8 (CODES & THEMES by KU).pdf. Transcript 9 (CODES & THEMES by KU).pdf. Transcript 10 (CODES & THEMES by KU).pdf. Transcript 11 (CODES & THEMES, by KU).pdf. Transcript 12 (CODES & THEMES by KU).pdf. Transcript 13 (CODES & THEMES by KU).pdf. Transcript 14 (CODED & THEMES by KU).pdf. Transcript 15_b (CODED & THEMES by KU). pdf. Transcript 16 (CODES & THEMES by KU).pdf. Transcript 17 (CODES & THEMES by KU).pdf. Transcript 18 (CODES & THEMES by KU).pdf. Transcript 19 (CODES & THEMES by HK).pdf. Transcript 20 (CODES & THEMES by HK).pdf. Transcript 21_b (CODES & THEMES by HK).pdfTranscript 22 (CODES & THEMES by HK).pdf. Transcript 25 (CODES & THEMES by HK).pdf. Transcript 27 (CODES & THEMES by HK).pdf. Transcript Sn1 (CODES & THEMES by RS).pdf Transcript Sn6 (pt3) (CODES & THEMES by RS).pdf. Transcript Sn15_a (CODES & THEMES by RS).pdf. Transcript SN17 (pt3) (CODES & THEMES by RS).pd. Transcript Sn21_a (CODES & THEMES by RS).pdf. (ZIP) [file pgph.0004794.s001.zip › Transcript 7 (CODES & THEMES, by KU).pdf]

| Text/transcript | Initial codes | Themes |
|---|---|---|
| **Transcription interview 7**<br>**Interviewee: XXX**<br>**Interviewer: (MS), Research Assistant**<br>**Number of speakers :3**<br>**Other Attendees: (KU), Principal Investigator**<br>**Time: 3.28pm**<br>**Length of interview recording: 15 minutes 46 seconds**<br>**Date: 30/3/23** | | |
| **1. Interviewer [MS]: Okay amazing. So the first thing we need to do is just go through the consent form that I sent you and sign it. Is that okay? so I'm just gonna share it on the screen** | | |
| 2. Interviewee [XXX]: okay *unclear speech* not too good. Let me put on my video *unclear speech* (puts video on) *background noise, unclear speech* | | |
| **3. Interviewer [MS]: Yeah If that's okay with you** | | |
| **4. *background noise overlapping*** | | |
| **5. Interviewer [MS]: So *showing participant information sheet, speech/audio not working*** | | |
| **6. *Participant Information Sheet off* can you hear me still when I put that on yeah?** | | |
| 7. Interviewee [XXX]: *overlapping unclear speech/background noise* | | |
| **8. Interviewer [MS]: okay amazing so** | | |
| **9. *overlapping unclear speech and background noise*** | | |
| **10. Interviewer [MS]: okay good so *broken up speech* (brought up Participant Information Sheet on zoom) *unable to hear MS speaking*** | | |
| 11. Principal Investigator [KU] : Eh so Ah *name of interviewee* , I will just send a message to her I think we have lost the sound. | | |
| 12. Interviewee [XXX]: Okay *unclear speech* hear her | | |
| **13. Interviewer [MS]: I'm back I'm back, the problem I'm having is when I share things on the screen it stops my audio for some reason** | | |
| 14. Interviewee [XXX]: Okay regarding the document I have read it through several times | | |
| **15. Interviewer [MS]: Okay, so you've read the participant information sheet and then** | | |
| 16. Interviewee [XXX]: yes *overlapping speech* | | |
| **17. Interviewer [MS]: through the consent form** | | |
| 18. Interviewee [XXX]: consent form yes | | |
| **19. Interviewer [MS]: Okay so do you consent to all of the consent form?** | | |
| 20. Interviewee [XXX]: Yes *unclear speech* | | |
| **21. Interviewer [MS]: perfect so if you consent to all of it, then after the interview I'll put your initials in all the boxes and write your name on the consent form, is that okay?** | | |
| 22. Interviewee [XXX]: That's alright | | |
| **23. Interviewer [MS]: Okay perfect, thank you and then once its all been signed we can send you a copy as well if we can try *unclear speech*** | | |
| **24. *overlapping unclear speech*** | | |
| **25. Interviewer [MS]: but yeah if you consent then that's perfect** | | |
| **26.** Interviewee [XXX]: That's okay | | |
| **27. Interviewer [MS]: okay. Also do you have an airtime card?** | | |
| 28. Interviewee [XXX]: hello? | | |
| **29. Interviewer [MS]: Hi. Do you have an airtime card?** | | |

| | | |
|---|---|---|
| 30. Interviewee [XXX]: Yes, I do | | |
| **31. Interviewer [MS]: Okay. Are you using it for this call?** | | |
| 32. Interviewee [XXX]: yes that's what I'm using | | |
| **33. Interviewer [MS]: okay so if you've got an airtime card you can submit it for a refund after the interview. Will it be the same *name of PI*** | | |
| 34. Principal Investigator [KU] : eh so he would submit the card to *name of a dr* | | |
| **35. Interviewer [MS]: okay** | | |
| 36. Principal Investigator [KU] : or somebody in *location name* who could contact *name of a dr* | | |
| **37. Interviewer [MS]: okay okay. Is that okay *name of interviewee*** | | |
| 38. Interviewee [XXX]: * slightly broken up speech* No worries I can contact *unclear word* directly | | |
| 39. *overlapping speech, unclear which male voice speaking saying no problem* | | |
| **40. Interviewer [MS]: perfect okay, so I am just going to start the interview now and ask you some questions. Is that okay?** | | |
| 41. Interviewee [XXX]: *unclear speech* | | |
| **42. Interviewer [MS]: okay** | | |
| 43. **Interviewer [MS]: So do you prescribe antibiotics to pregnant women?** | 43. Prescribing antibiotics | |
| 44. Interviewee [XXX]: of course, definitely so | | [1] Prescribing antibiotics (frequency, setting) |
| 45. **Interviewer [MS]: Okay, how long have you been prescribing them for?** | | |
| 46. Interviewee [XXX]: a very long time | | |
| **47. Interviewer [MS]: How many years? *overlapping unclear speech*** | | |
| 48. Interviewee [XXX]: *unclear speech* | | |
| 49. **Interviewer [MS]: okay okay** | | |
| 50. **Interviewer [MS]: So how many times a week are you prescribing them for women now?** | | |
| 51. Interviewee [XXX]: like weekly in most cases | 51. Prescribe regularly (weekly) | |
| **52. Interviewer [MS]: okay** | | |
| 53. Interviewee [XXX]: every week, the obstetric ladies every week, sometimes on the wards so its like eh lets say twice or *unclear speech* three times a week | | |
| **54. Interviewer [MS]: mmhm okay** | | |
| 55. Interviewee [XXX]: *overlapping speech* when on the ward rounds and then *unclear speech* clinics | 53/55. Prescribe regularly, on ward rounds/at clinic | |
| 56. **Interviewer [MS]: Great. What are the 3 most common medical problems that you prescribe the antibiotics for?** | | |
| 57. Interviewee [XXX]: *unclear speech* women, most of the prescriptions of pregnant women are for urinary tract infections or upper respiratory tract infections | 57. To treat UTIs | |
| 58. **Interviewer [MS]: Okay. Do you use any guidelines when you're prescribing antibiotics?** | | |
| 59. Interviewee [XXX]: I'm an Obstetrician gynaecologists so there are there are guidelines of prescriptions from different bodies | | |
| **60. Interviewer [MS]: Mhmm mhmm** | | |
| 61. Interviewee [XXX]: for different conditions there are guidelines and of course we use microscopy | | |
| **62. Interviewer [MS]: Mhmm *overlap*** | | |
| 63. Interviewee [XXX]: To guide us *unclear speech* prescription in the hospital | | |
| 64. **Interviewer [MS]: Okay great. Where do you find that pregnant women generally get their antibiotics from? Like the hospital pharmacy, or outside of the hospital?** | | |
| 65. Interviewee [XXX]: In most cases the patients buy it from the hospital pharmacy, when the hospitals out of it they buy it from outside pharmaceuticals | 65. Obtaining antibiotics | [2] Obtaining antibiotics (hosp, phar) |
| 66. **Interviewer [MS]: Okay, so are you aware of any pregnant women who may have taken antibiotics that haven't been prescribed for them?** | | |
| 67. Interviewee [XXX]: very common in Nigeria | 67. Frequency of self-medication with antibiotics | |

| | | |
|---|---|---|
| **68. Interviewer [MS]: Dya have any examples or more details that you could tell us about?** | | |
| 69. Interviewee [XXX]: *unclear speech* when you see women when they come and they complain *unclear speech* I'm going to give you this *unclear speech* what have you taken before *unclear speech* who prescribed *unclear speech* they say they went to the chemist and they bought it on their own | 69. Self-medication (obtained from chemist) | [3] Self-medication (freq, source) |
| **70. Interviewer [MS]: Okay** | | |
| **71.** Interviewee [XXX]: so mostly the condition *unclear speech* | | |
| **72. Interviewer [MS]: mhmm. So are you aware of any pregnant women who might take like herbal preparations or alternative mediations that work like antibiotics, instead of antibiotics?** | | |
| 73. Interviewee [XXX]: yes they do *unclear speech* herbal medications, yeah its well-practiced | 73. Herbal self-medication | [4] Herbal self-medication (cultural, evidence, detecting) |
| **74. Interviewer [MS]: okay do you have any examples?** | | |
| 75. Interviewee [XXX]: the herbal preparations they are not medical preparations actually | 75. Herbs/not medical | |
| **76. Interviewer [MS]: mhmm** | | |
| 77. Interviewee [XXX]: herbal preparations according traditional medicine | 77. Herbs/traditional medicine | |
| **78. Interviewer [MS]: Okay okay. Do they bring them to hospital ever, have you ever like have they ever shown you them?** | | |
| 79. Interviewee [XXX]: You said? | | |
| **80. Interviewer [MS]: have they ever shown you any when they've come into hospital?** | | |
| 81. Interviewee [XXX]: *unclear speech* occasionally if they have some symptoms that you feel are drug reactions | 81. Evidence of herbal use | |
| **82. Interviewer [MS]: mhmm** | | |
| 83. Interviewee [XXX]: Tell them to bring what what they are taking you see | 83. Asking about herbal use | |
| **84. Interviewer [MS]: mhmm** | | |
| 85. Interviewee [XXX]: The concoctions | | |
| **86. Interviewer [MS]: mhmm okay** | | |
| **87. *overlapping speech noted*** | | |
| 88. **Interviewer [MS]: Do you know of any ways at the moment to identify or detect when women are self-medicating with antibiotics in pregnancy?** | | |
| 89. Interviewee [XXX]: you said please pardon? | | |
| 90. **Interviewer [MS]: Do you know of any methods that can detect or identify when women are self-medicating with antibiotics in pregnancy?** | | |
| 91. Interviewee [XXX]: you mean you mean clinically or investigation wise | | |
| **92. Interviewer [MS]: yeah either** | | |
| **93.** Interviewee [XXX]: what we try to do is we discuss we talk to them, when they come for follow ups when they are pregnant we talk to them *unclear speech* if you're taking something you must tell in most cases if you are open and simple to them they will tell you they have taken something *unclear speech* have taken something we don't do any investigations to assess if they have taken something antibiotics over the counter | 93. Ways to detect antibiotic SM (discussion with patient/no investigation) | [5] Detecting SM (methods…) |
| 94. **Interviewer [MS]: Okay. Do you think it could be useful to have like a simple rapid test or tool or questionnaire that could help identify pregnant women who have been misusing antibiotics without us knowing?** | | |
| 95. Interviewee [XXX]: A questionnaire will do because *unclear speech* question truthfully *unclear speech* | 95. Need for detection method (questionnaire) | |
| **96. Interviewer [MS]: mhmm** | | |
| 97. Interviewee [XXX]: I must tell you during the health docs antenatal clinic pregnant women the nurses tell them not to self medicate *overlap unclear speech*. Self-medication is a very common thing in this country in Nigeria *unclear speech* advised not to self medicate during pregnancy especially because of the harm likely harm to the baby | 97. Nurses input (they advise on SM, as very common) | |
| **98. Interviewer [MS]: Okay, so if such a tool or questionnaire was available, would you be interested in using it?** | | |

| | | |
|---|---|---|
| 99. Interviewee [XXX]: Yes it will give us that insight on numbers of women that do that because it is common even more common now I think | 99. Value of a tool for detecting SM | |
| **100. Interviewer [MS]: mhmm** | | |
| 101. Interviewee [XXX]: yes | | |
| **102. Interviewer [MS]: Okay** | | |
| **103. *over-lapping speech*** | | |
| 104. **Interviewer [MS]: So if we had a tool such em as this do you think it could be useful to use in antenatal settings, or during routine appointments, or during A&E?** | | |
| 105. Interviewee [XXX]: Yes | 105/107. Useful in antenatal care settings. | |
| **106. Interviewer [MS]: Yeah you think it could be used in all those settings or better somewhere else?** | | |
| 107. Interviewee [XXX]: *overlapping speech* antenatal clinics and then postnatal clinics | | |
| **108. Interviewer [MS]: mhmm** | | |
| 109. Interviewee [XXX]: *fast speech* In the postnatal clinics they are feeding they are breastfeeding their babies. Babies are outside already but they are breastfeeding these babies, *unclear speech* passed through the breastmilk to get to the babies | 109. Risks of antibiotic SM in pregnancy (breast feeding) | [7] SM detection (side effects, risks of SM) 109, 110, 147 to 159, 166 |
| **110. Interviewer [MS]: mhmm** | | |
| 111. Interviewee [XXX]: *overlapping speech* so antibiotics actually eh not recommended for children and eh when they are nursing their children *unclear speech* those antibiotics might get to the breastmilk and then transfer to the baby | 111. Risks of antibiotic SM in pregnancy (not recommended for children) | |
| **112. Interviewer [MS]: mmm** | | |
| 113. Interviewee [XXX]: *overlapping unclear speech* | | |
| 114. **Interviewer [MS]: That's true, definitely. Dya think it would be useful for such a test or tool to be mobile, like remote, easy to use without having to use electricity?** | | |
| 115. Interviewee [XXX]: You said? | | |
| **116. Interviewer [MS]: Just say if we had a tool we could use dya think it would be better or useful if it was like easy to use or remote so we didn't need to use electricity to use it?** | | |
| 117. Interviewee [XXX]: It's okay *unclear speech* challenging at times | | |
| **118. Interviewer [MS]: mmm** | | |
| **119.** Interviewee [XXX]: *unclear speech* can function very well here *unclear speech* | | |
| **120. Interviewer [MS]: Okay, so it wouldn't really matter?** | | |
| **121.** Interviewee [XXX]: Yes it wouldn't matter | | |
| 122. **Interviewer [MS]: Okay, so have you come across any methods or guidelines which help detect side effects of antibiotic self-medication in pregnant women?** | | |
| 123. Interviewee [XXX]: Of course *unclear speech*, so I teach students medical students *unclear speech* and I have doctors under me that I train so there are guidelines and there are actually texts, there are books about drug use in pregnancy and antibiotics Is a whole chapter on that | 123. Side effects of antibiotic SM (guidelines) | [7] Side effects |
| **124. Interviewer [MS]: mmm** | | |
| 125. Interviewee [XXX]: So the doctors get to know and I teach the students so there are books and there are *unclear speech* from that | 125. Side effects (books) | |
| **126. Interviewer [MS]: Okay *overlapping speech*** | | |
| **127.** Interviewee [XXX]:*unclear speech* In pregnancy | | |
| **128. Interviewer [MS]: Okay, Okay. So for when people do self medication of antibiotics** | | |
| 129. Interviewee [XXX]: pardon? | | |
| **130. Interviewer [MS]: So when women self medicate with antibiotics that havent been prescribed is there guidelines for that, for how to deal with the side effects for that?** | | [6] Detecting SM |
| 131. Interviewee [XXX]: In this country there is no guideline like that | 131. Lack of specific guidelines | |

| | | |
|---|---|---|
| **132. Interviewer [MS]: Okay okay** | on side effects from SM | (guidelines) 123, 131, 134-139 |
| **133. *overlapping speech*** | | |
| 134. Interviewee [XXX]: *unclear speech* guidelines that I'm aware of | | |
| **135. Interviewer [MS]: Okay** | 134/136/139. No guidelines | |
| 136. Interviewee [XXX]: *unclear speech* especially in pregnancy | | |
| 137. **Interviewer [MS]: Fine** | | |
| 138. *overlapping speech* | | |
| 139. Interviewee [XXX]: *unclear speech* any guideline not necessarily antibiotics | | |
| **140. Interviewer [MS]: Fine so it could be more from like experience that you have** | | |
| **141.** Interviewee [XXX]: *overlapping speech* yes | | |
| **142. Interviewer [MS]: and things like that** | | |
| 143. Interviewee [XXX]: *unclear speech* | | |
| **144. Interviewer [MS]: Mhmm okay amazing** | | |
| 145. Interviewee [XXX]: So theres a lot of experience *unclear speech* | | |
| **146. Interviewer [MS]: definitely. So antibiotics can cause side effects such as stomach upset as we know, like sometimes stomach upset or people feeling ill. Do you think that those side effects are presence clear in a patient that's kind of taking antibiotics when they have general side effects is it clear?** | | |
| 147. Interviewee [XXX]: Sometimes its clear because they have certain most drugs will have some similar side effects | 147. Side effects in patients | |
| **148. Interviewer [MS]: Mhmm** | | [7] Side effects |
| 149. Interviewee [XXX]: like *unclear speech* vomiting general side effect that caused many drugs *unclear fast speech* when they take and when they self medicate and they take their own drugs they will come and complain *unclear speech* they say yes | 149. Side effects from antibiotic SM | |
| **150. Interviewer [MS]: kay** | | |
| **151. *overlapping speech*** | | |
| **152. Interviewer [MS]: do you know** | | |
| **153. *overlapping speech*** | | |
| **154. Interviewer [MS]: Okay. Do you know of any pregnant women that have been suspected to have developed any side effects of antibiotic self-medication, have you come across that a lot?** | | |
| 155. Interviewee [XXX]:A lot of course | 155. Side effects of antibiotic SM (many women) | |
| **156. Interviewer [MS]: Okay, dya have any examples?** | | |
| 157. Interviewee [XXX]: A lot of them will *unclear speech* examples, *unclear speech* examples. Yeah pregnant sometimes um signs and symptoms *unclear speech*. Sometimes reactions like rash reactions *unclear speech* non-prescription drugs, they buy the drugs over the counter *unclear speech* | 157. Side effects of antibiotic SM (signs/symptoms) | [7] Side effects |
| **158. Interviewer [MS]: Mhmm** | | |
| 159. Interviewee [XXX]: *unclear speech* in most cases they've taken antibiotics too | 159. Side effects | |
| **160. Interviewer [MS]: Mhmm Okay so we kind of touched on this as well before so do you know any methods or guidelines or protocols that look at antibiotic self-mediation in pregnant women specifically?** | | |
| 161. Interviewee [XXX]: No, none that I know | | |
| **162. Interviewer [MS]: No, okay, that's fine** | | |
| **163. *overlapping speech*** | | |
| 164. Interviewee [XXX]: *unclear speech* antibiotics in pregnancy | | |
| **165. Interviewer [MS]: Okay yeah of course. So then this is quite a specific question so its to do with pregnant women who have self medicated with antibiotics, sometimes they might develop memory loss or forgetfulness. Em do you know of any management options if that happened? Or kind of had you ever had to deal with that?** | | |
| 166. Interviewee [XXX]: You know pregnant women are generally forgetful | 166. Neurological effects (of SM) | [7] Side effects |
| **167. Interviewer [MS]: Mhmm** | | |
| 168. Interviewee [XXX]: so *unclear speech* not seen it | | |

| | | |
|---|---|---|
| **169. Interviewer [MS]: Mhmm, okay so you've not have you never experienced that**<br>**170. *Overlapping speech***<br>171. Interviewee [XXX]: I've not *unclear speech*<br>**172. Interviewer [MS]: Okay, amazing that's fine just one of the questions, perfect, so that's all of my questions em do you have any questions about anything to do with the study or anything?**<br>173. Interviewee [XXX]: no for now no<br>**174. Interviewer [MS]: Okay, if you do you've got my email, you've got our email address em**<br>**175. *overlapping speech***<br>**176. Interviewer [MS]: Need to ask, but I really appreciate you taking the time today, I know it was last minute so I really appreciate you taking the time**<br>177. Interviewee [XXX]: Okay thank you so much<br>**178. Interviewer [MS]: No problem so that's all, when its finished you can submit your airtime card as we discussed earlier em**<br>**179. *overlapping speech***<br>**180. Interviewer [MS]: and if you've got any *unclear speech* let us know**<br>181. Interviewee [XXX]: okay no problem about that<br>**182. Interviewer [MS]: Okay great, thank you so much**<br>183. Interviewee [XXX]: Okay<br>**184. Interviewer [MS]: Have a good rest of the day**<br>185. Interviewee [XXX]: *name of PI* nice see you, okay, bye bye<br>**186. Interviewer [MS]: bye**<br>187. Interviewee [XXX]: bye by *name of interviewer*<br>**188. Interviewer [MS]: bye** | | |
