# Supplementary material for: Advancing the safe motherhood initiative: A qualitative and sentiment analysis of local physician’s perspectives on antibiotic self-medication during pregnancy in a low- and middle-income country
Source: PLOS Glob Public Health. 2025 Sep 12;5(9):e0004794. doi: 10.1371/journal.pgph.0004794 (PMC12431270; doi:10.1371/journal.pgph.0004794)
Supplement: S1 File — Transcript 4 (CODES & THEMES by KU).pdf. Transcript 6 (CODES & THEMES by KU).pdf. Transcript 7 (CODES & THEMES, by KU).pdf. Transcript 8 (CODES & THEMES by KU).pdf. Transcript 9 (CODES & THEMES by KU).pdf. Transcript 10 (CODES & THEMES by KU).pdf. Transcript 11 (CODES & THEMES, by KU).pdf. Transcript 12 (CODES & THEMES by KU).pdf. Transcript 13 (CODES & THEMES by KU).pdf. Transcript 14 (CODED & THEMES by KU).pdf. Transcript 15_b (CODED & THEMES by KU). pdf. Transcript 16 (CODES & THEMES by KU).pdf. Transcript 17 (CODES & THEMES by KU).pdf. Transcript 18 (CODES & THEMES by KU).pdf. Transcript 19 (CODES & THEMES by HK).pdf. Transcript 20 (CODES & THEMES by HK).pdf. Transcript 21_b (CODES & THEMES by HK).pdfTranscript 22 (CODES & THEMES by HK).pdf. Transcript 25 (CODES & THEMES by HK).pdf. Transcript 27 (CODES & THEMES by HK).pdf. Transcript Sn1 (CODES & THEMES by RS).pdf Transcript Sn6 (pt3) (CODES & THEMES by RS).pdf. Transcript Sn15_a (CODES & THEMES by RS).pdf. Transcript SN17 (pt3) (CODES & THEMES by RS).pd. Transcript Sn21_a (CODES & THEMES by RS).pdf. (ZIP) [file pgph.0004794.s001.zip › Transcript 8 (CODES & THEMES by KU).pdf]

Text/transcript	Initial Codes	Themes
**Transcription interview 8**<br>**Interviewee: [XXX]**<br>**SN- 31**<br>**Interviewer: (MS), Research Assistant**<br>**Number of speakers :3**<br>**Other Attendees:**<br>**Time: 6.28pm**<br>**Length of interview recording: 22 Minutes 49 seconds**<br>**Date: 30/3/23**<br><br>**1. Interviewer [MS]: Perfect *background noise/participant unclear speech* thank you so much for joining us today and making the time after work * background noise/participant unclear speech* I really appreciate it. So I sent you the information sheet and also the consent form, have you had a chance to read through it?**<br>2. Interviewee [XXX]: I didn't get that sorry<br>**3. Interviewer [MS]: The information sheet and consent form that I sent to you, have you had a chance to read through it?**<br>4. Interviewee [XXX]: Yes yes I went through it that's why I remembered *unclear speech* for the interview<br>**5. Interviewer [MS]: Perfect so I'm just going to share with you *background noise* quickly, *RA shares screen***<br>6. Interviewee [XXX]: Can you hear me?<br>**7. Interviewer [MS]: yeah one second yeah when I share my screen, hang on one second it will work now *shares screen again*. So this is what I sent to you the participant information sheet, is this what have you had a read through it?**<br>8. Interviewee [XXX]: Can you hear me?<br>**9. Interviewer [MS]: yes I can hear you. Can you hear me?**<br>10. Interviewee [XXX]: Yes I can hear you<br>**11. Interviewer [MS]: Can you see this? On the screen?**<br>12. Interviewee [XXX]: Okay I'm trying to zoom in, yes that is the information you sent me earlier		

**13. Interviewer [MS]: yeah yes, so you've had a read through it yeah?**<br>14. Interviewee [XXX]: Yes I did<br>**15. Interviewer [MS]: Perfect, dya have any questions about it?**<br>16. Interviewee [XXX]: no no its okay *unclear speech*<br>**17. Interviewer [MS]: *overlapping speech* perfect, so this is the *sound goes off* *consent form on screen* one sec *RA speaking quietly to self* consent form**<br>18. Interviewee [XXX]: Once it starts sharing the screen *unclear speech*<br>**19. Interviewer [MS]: I know, I figured, can you hear me now?**<br>20. Interviewee [XXX]: yes I can hear you now yes<br>**21. Interviewer [MS]: can you hear me now?**<br>Interviewee [XXX]: Yes I can<br>**22. Interviewer [MS]: right so this is the consent form um so I sent you this to have a look at as well didn't I yeah? Em so did you have a read through the consent form?**<br>23. Interviewee [XXX]: yes I also went through<br>24. **Interviewer [MS]: perfect do you consent to all of the points? Or do you want me to go through every point?**<br>**25.** Interviewee [XXX]: No I consented to all of the point<br>26. **Interviewer [MS]: Fine so are you happy for me to put your initials in here? Are you okay with that? Cause you can't do it. Are you happy for me to do it?**<br>27. Interviewee [XXX]: *overlapping speech* aaactually my full name is *says full name*<br>**28. Interviewer [MS]: So whats your initials sorry**<br>29. Interviewee [XXX]: *says initials*<br>**30. Interviewer [MS]: *confirms initials* perfect. So you're happy for me to sign it all? For you?**<br>31. Interviewee [XXX]: sure sure *unclear speech*<br>**32. Interviewer [MS]: yeah you consent to it all perfect. Um and the information sheet that's here highlighted is the one that you have read already. Um and you're happy**		

**for it to be audio and video recorded. So you consent to all of these points and you're happy to take part in the study yeah?**<br>33. Interviewee [XXX]: Its okay<br>**34. Interviewer [MS]: perfect and then I'm just going to put your. So how do spell your first name?**<br>35. Interviewee [XXX]: *Spells first name*<br>**36. Interviewer [MS]: *confirming spelling***<br>37. Interviewee [XXX]: *Spells first name*<br>**38. Interviewer [MS]: that?**<br>**39.** Interviewee [XXX]: No *corrects name*<br>**40. Interviewer [MS]: *confirms letter***<br>41. Interviewee [XXX]:*confirms letter*<br>**42. Interviewer [MS]: Is that right?**<br>**43.** Interviewee [XXX]: Okay that's fine, that's fine<br>**44. Interviewer [MS]: Is that right yeah?**<br>45. Interviewee [XXX]: yes<br>**46. Interviewer [MS]: perfect and then the date is the *says date* and then because you can't sign it um I just need to put your *clears throat* initials in here *says initials* perfect and kay that's everything and that's my signature and our investigators signature. So that's fine perfect, so youre happy to take part, that's great um fab so what ill just confirm with my manager whose in charge what were hoping to do is after weve done everything ill be able to send you the consent form so you have it for your records as well, that's what were hoping to do urm so then you have it as well okay?**<br>47. Interviewee [XXX]: okay *unclear speech*<br>**48. Interviewer [MS]: I just want to double check, um do you have an airtime card that you're using for this interview? Are you using wifi?**<br>49. Interviewee [XXX]: I didn't get that sorry<br>50. **Interviewer [MS]: are you, do you have an airtime card that you're using for the interview?**<br>51. Interviewee [XXX]: yes that's what im trying to do I don't have any wifi so im using the airtime		

52. **Interviewer [MS]: *unclear speech* so if you're using airtime then you can submit the card or receipt to *name of dr* for a refund for whatever airtime you use during this call**<br>53. Interviewee [XXX]: okay okay<br>**54. Interviewer [MS]: Okay perfect so just remember to do that, so im just going to start asking you some questions um and just answer them to the best of your ability and if you don't want to answer any that's fine, just ask me to move onto the next one. Is that okay?**<br>**55.** Interviewee [XXX]: Alright alright perfect<br>56. **Interviewer [MS]: So do you prescribe antibiotics to pregnant women?**		
57. Interviewee [XXX]:Yes I do<br>**58. Interviewer [MS]: How often, how many times a week?**	**57. Prescribe antibiotics to pregnant women**	**1. PRESCRIBING ANTIBIOTICS**
59. Interviewee [XXX]: ah um either its about its usually *unclear speech* weekly once we see patients at the clinic *clears throat*<br>60. **Interviewer [MS]: and how long have you been prescribing antibiotics to pregnant women?**	**59. Prescribed during patient visits to clinic**	
61. Interviewee [XXX]: ooo mm lets say em more than 10 years now, 10 15<br>62. **Interviewer [MS]: Okay and what are the 3 most common medical problems that you prescribe antibiotics for?**<br>63. Interviewee [XXX]: I didn't get it sorry<br>**64. Interviewer [MS]: what are the 3 most common medical problems that you prescribe antibiotics for?**	**61. Years' experience prescribing antibiotics**	
65. Interviewee [XXX]: Okay urm most common medical condition is upper respiratory tract infections okay then um urinary tract infection ahh whether it was pneumonia or just um just a you know upper respiratory tract infections too then um post partum post partum period is not part of the review<br>**66. Interviewer [MS]: mmhmm yeah um its more the question says pregnant women and were looking at kind of antenatal so we would say when they're pregnant really**	**65. Prescribed for various infections**<br><br>**67. Prescribed to manage various**	

67. Interviewee [XXX]: Okay okay okay fine so most times upper respiratory tract infections, lower respiratory tract infections, urinary tract infections urm a coupe of them any form of sepsis any form of sepsis *unclear speech*antenatal period *unclear speech* boils somewhere that needed to cover antibiotics *unclear speech*. Then of course if they have any investigative procedure In pregnancy they must usually cover with antibiotics, for instance a woman you're managing for cervical incompetence and *unclear speech* antibiotics, if there was um rupture of membranes usually um preterm premature rupture of membranes *unclear speech* conservative management so wide range of eh medication *unclear speech* in pregnancy<br>**68. Interviewer [MS]: Okay so do you have any guidelines that you use when you prescribe antibiotics?**	**pregnancy-related complications/ailments**	
69. Interviewee [XXX]: Yes where I work at the teaching hospital there is *unclear speech*, so we have departmental guidelines In terms of management of patients so *unclear speech* departmental guidelines usually specifies um *clears throat* the range of antibiotic that have been that are ought to be used for certain conditions in pregnancy. Um they may not be specific in terms of brand you ought to use but it gives an idea of what *unclear speech*<br>**70. Interviewer [MS]: Okay *overlapping***<br>71. **Interviewer [MS]: Okay so where do you find antenatally that you know pregnant normally get antibiotics from? Do they get it from like hospital pharmacy or health centre, clinic or they just go to a pharmacy, where do they normally get their antibiotics from?**	**69. Guidelines on antibiotic use/not specific**	
72. Interviewee [XXX]:well um because of our environment *unclear speech* setting, um when we prescribe at the teaching hospital eh it is expected they collect it at the teaching hospital, central pharmacy. Um however we also know that at some point	**72. Obtaining ATB, Self-medication/OTC**	**2. OBTAINING ATB**

they may actually get to get them go outside of the hospital to get it over the counter<br>**73. Interviewer [MS]: mhmm**<br>74. Interviewee [XXX]: Some of them may even get those antibiotics *unclear speech*<br>**75. Interviewer [MS]: mmm**<br>76. Interviewee [XXX]: so they can easily get antibiotics over the counter even without prescription<br>**77. Interviewer [MS]: mhmm, so so similar are you aware of any pregnant women who take antibiotics that haven't been prescribed for them?**<br>78. Interviewee [XXX]: sure sure sure a number of them do, well with the increase in awareness and knowledge those who are knowledgeable enough may want to wait and assist *car horn outside*, that the prescription comes from their doctor, but *unclear speech* some will still go ahead either that um they have some relative who are medical inclined they might not be doctors but give them that verbal prescription<br>**79. Interviewer [MS]: Okay fine so you see it a lot at work?**<br>80. Interviewee [XXX]: oh a number of them yeah yeah<br>81. **Interviewer [MS]: okay are you also aware of pregnant women who might take like herbal preparations or alternative medications instead of antibiotics?**<br>82. Interviewee [XXX]: sure sure this is *unclear speech* for certain *unclear speech* Nigerial *car horn in background* they have a number of *unclear speech* villages that may not be accessible to *coughs* excuse me gynaecologists around them so they do *unclear speech* when they are being seen by *unclear speech* they have a lot of them having take these med *unclear speech*<br>**83. Interviewer [MS]: Dya have any examples of any like herbal preparations or alternative medications that they use, like any names of any?**	**76. Obtaining ATB, Self-medication/OTC, without a prescription**<br><br>**78. Self-medication/obtained from relatives**<br><br>**82. Herbal self-medication**	**3. SELF-MEDICATION**<br><br>**4. HERBAL SELF-MEDICATION**

84. Interviewee [XXX]: *laughs* I cant exactly say but quite often they come around and mention what I actually want to find out is it um what actually was the form of the preparation, do you have to cook the leaves before you drink it are you gonna chew the leaves are you gonna squeeze the *unclear speech* from the leaves *unclear speech*, I just wanna find out what form of herbal preparation<br>**85. Interviewer [MS]: mmhmm**<br>86. Interviewee [XXX]: I don't really care to find out the name exactly *unclear speech*<br>**87. Interviewer [MS]: mhmm mhm, more want to know whats in it yeah**<br>88. Interviewee [XXX]: *unclear speech* want to know you may not know what is in it they are told this is what we have to do<br>**89. Interviewer [MS]: oh**<br>**90.** Interviewee [XXX]: take it in the morning, take it at night either you squeeze the juice cook it *unclear speech* and take water whatever *unclear speech* just go ahead and take it<br>91. **Interviewer [MS]: Okay. So do you know of any methods that are there to identify or detect self-medication of antibiotics in pregnant women? Like how would you know?**<br>92. Interviewee [XXX]: Can you go through that again?<br>93. **Interviewer [MS]: so do you know of any methods or any ways that can identify when a woman is self medicating antibiotics that havent been prescribed?**<br>94. Interviewee [XXX]: basically what we do is history taking<br>**95. Interviewer [MS]: Mhmm**<br>96. Interviewee [XXX]: By the time you try to find out though history taking, that's the *unclear* way eh by the time you're taking a DETAILED history *unclear speech* they have been on self medication antibiotic<br>**97. Interviewer [MS]: Mhmm. Do you think it would be useful to have like a simple rapid test or a lab test or a tool or a questionnaire that could help identify**	**84. Type/preparation of herbs used in self-medication**<br>**86. Identifying/naming herbs used**<br>**88. Content of herbs**<br>**90. Using the herbs**<br>**94. Detecting self-medication (history taking)**<br>**96. History taking occurs well after the fact (i.e., after onset of self-medication)**	**5. DETECTING SELF-MEDICATION (METHODS)**

**pregnant women who might be misusing antibiotics?**<br>98. *can hear children in the background*<br>99. Interviewee [XXX]: sure sure I think its very important its very important<br>**100. Interviewer [MS]: What kind of thing could you imagine would be useful?**<br>101. Interviewee [XXX]: ummm ahh I think ill talk about in respect to antimicrobial stewardship ahhh but ive not been able to have *unclear speech* maybe a point of care test at the point they are at antenatal care *unclear speech* going to be really available and cost effective I think its gonna be nice<br>**102. Interviewer [MS]: Mhmm**<br>103. Interviewee [XXX]: management of those patient when they come *unclear speech* with antibiotics<br>**104. Interviewer [MS]: Mhmm, so If such a tool was available, would you be interested in using it?**<br>105. Interviewee [XXX]: sure sure<br>**106. Interviewer [MS]: *overlapping speech***<br>107. Interviewee [XXX]: because of the sorry?<br>**108. Interviewer [MS]: why do you would use it the most?**<br>109. Interviewee [XXX]: ah weve had quite a number of patients who were on self medication because of the poverty because of the financial handicap um because of non-availability of gynecologists obstetricians I think *unclear speech*they go ahead to do self medication so um even after when you've done all by the time they come to you you can be able to use an available tool *unclear speech* whether they've *unclear speech* I think its quite useful<br>110. **Interviewer [MS]: Mhmm so do you think, would you could it be used in like antenatal care settings, or routine appointments, or like A&E places like that?** *car horn in background* **Do you think it would be useful to use it there?**	**99. Need for method to detect self-medication**<br><br>**101. Need for cost-effective Point-of-Care test**<br><br>**105. Interest in detection tool**<br><br>**109. Importance of detecting self-medication in patients self-medicating for different reasons**	

111. Interviewee [XXX]: yes I think so yes it would be very useful *unclear speech*	**111. Detection in antenatal care settings**	
112. **Interviewer [MS]: Mhmm, and do you think it would be useful for such a test to be like mobile and remote and easy to use like do you think it would need internet?**		
113. Interviewee [XXX]: sure sure sure readily available *overlapping speech* very easy to use point of care test	**113. Readily available and easy to use point of care test, to detect antibiotic self-medication**	
**114. Interviewer [MS]: Mhmm**	**115. Easy to use**	
115. Interviewee [XXX]: I think that will be if its *unclear speech* then *unclear speech* simple easy to use *unclear speech*		
**116. Interviewer [MS]: Would you want like without electricity you would be able to use it, like a mobile test?**	**117. Mobile/not requiring electric power**	
117. Interviewee [XXX]: Sure sure I think so that would also be nice	**119. Appealing to patients**	
**118. Interviewer [MS]: okay**		
119. Interviewee [XXX]: I think it could easily reach out to those patients and they can easily have that test done		
**120. Interviewer [MS]: mhmm amazing. Have you come across any methods or guidelines which help detect side effects of antibiotic self-medication in pregnant women?**		
121. Interviewee [XXX]: ahh ive not been able to come across any method all you do is to get in touch with the pharmacy and in the case of complications from side effects of antibiotic	**121. Side effects of antibiotic self-medication**	
**122. Interviewer [MS]: fine so its not like a specific guideline for self medication**	**123. Guidelines on self-medication**	**[6] GUIDELINES**
123. Interviewee [XXX]: I I don't think so, what I know is they have a pharmacal vigilance unit I don't know how active it is but I don't think specific guideline but I think they have a unit *unclear speech*		
124. **Interviewer [MS]: Okay, so as we know antibiotics anyway can cause side effects so like stomach upset, feeling unwell, um do you think when someone has such side effects from antibiotics that it's clear?**		

125. Interviewee [XXX]: it may not be clear for some patients	**125. Identifying antibiotic side effects**	
**126. Interviewer [MS]: mhmm**		**[7] SIDE EFFECTS**
127. Interviewee [XXX]: but some patients actually have a way of identifying the particular drug that they are reacting to. So sometimes you can have patients coming in when you give them 2 antibiotics con *unclear word* and they can say that this side effect feels she feels like this is a particular drug *unclear speech* antibiotics	**127. Identifying antibiotic side effects**	
**128. Interviewer [MS]: mhmm**		
129. Interviewee [XXX]: so somehow *unclear speech* may identify larger proportion *unclear speech* some of them are vague symptoms of those side effects	**129. Vague symptoms of side-effects**	
130. **Interviewer [MS]: Do you know of any pregnant women that have had like suspected like side effects from taking antibiotics without a prescription so like self medicating?**		
131. Interviewee [XXX]: ahh I think I do ah not to clear but I think ive had the time I the time I identified the pharmacovigulance unit from the pharmacy department something took me there *unclear speech* I think that was a case of a side effect from drugs *unclear speech* pharmacovigulance unit there	**131. Side effects from self-medication with antibiotics**	
**132. Interviewer [MS]: *overlapping speech***		
133. Interviewee [XXX]: *unclear speech* common I don't think its common *unclear speech*	**133. Uncommon (identifying side effects from antibiotic SM)**	
**134. Interviewer [MS]: Okay so its not common to see side effects from self-medication?of antibiotics?**		
135. Interviewee [XXX]: ah yes I don't think, its there side effects is there but I don't think its common	**135. Uncommon (identifying side effects from antibiotic SM)**	
136. **Interviewer [MS]: Okay, em do you know similar to previous questions, do you know of any methods or guidelines to manage antibiotic self-medication in pregnant women?**		

137. Interviewee [XXX]: There is no methods or guideline, ive not come across any method<br>138. **Interviewer [MS]: Okay *overlapping unclear speech* okay that's fine and then this is regards to a specific area, so sometimes pregnant women who have self medicated with antibiotics, sometimes might develop signs of memory loss, or forgetfullness, are there any management options that you know if that happened, like what would you do?**<br>139. Interviewee [XXX]: okay, eh first ah *unclear word* management, of course you have to stop the drugs<br>**140. Interviewer [MS]: mhmm**<br>141. Interviewee [XXX]: em then ah the patient is managed um symptomatically based on this symptom<br>**142. Interviewer [MS]: mhmm**<br>143. Interviewee [XXX]: like you mentioned the memory loss and all that too. Um there is no specific guideline on that but I know I manage patients symptomatically patient symptom the patient presenting the side effects<br>**144. Interviewer [MS]: mhmm. Okay that's great that's good to know perfect so that's all the questions um so that was really helpful thank you very very much. Um that do you have any questions about anything?**<br>145. Interviewee [XXX]: ahhh I am a little bit curious in trying to know if there is modality for identifying patients that actually self medicate to use readily available mobile as you mentioned am curious to know<br>**146. Interviewer [MS]: so were so once all the research has been done and weve potentially we have all the results and well write a paper then hopefully there will be further research, this is kind of the beginning stage so once we've got the data and everything well be able if you know if participants want the report we should be able to kind of show you and send you the report once everythings**	**137. Guidelines for managing SM**<br><br>**143. Guidelines on SM (lack of)**<br><br>**145. Way to detect SM (using mobile device)**	**[6] GUIDELINES**

**completed umm so we can keep you updated with further progress, its just hoping there will be further work done after this project hopefully**<br>147. Interviewee [XXX]: wow I will be glad to have this feedback<br>**148. Interviewer [MS]: yeah yeah definitely. We should be able to send you feedback once weve done everything and once the study is kind of finished mm so we can keep you updated but thank you so much for taking time out , I know you've had a busy day and after work so I really appreciate it, um if you've got any other questions you've got my number for the study you've got my email mm so yeah that's everything and just remember to send your airtime card to voucher to *name of dr* for the call and he will be able to give you a refund**<br>149. Interviewee [XXX]: ok ok<br>**150. Interviewer [MS]: perfect thank you so much, thank you so much I hope you have a good night and let me know if you've got any other questions**<br>151. Interviewee [XXX]: thank you very much *unclear speech*<br>**152. Interviewer [MS]: no problem no problem bye**<br>**153.** Interviewee [XXX]: bye		
