# Supplementary material for: Advancing the safe motherhood initiative: A qualitative and sentiment analysis of local physician’s perspectives on antibiotic self-medication during pregnancy in a low- and middle-income country
Source: PLOS Glob Public Health. 2025 Sep 12;5(9):e0004794. doi: 10.1371/journal.pgph.0004794 (PMC12431270; doi:10.1371/journal.pgph.0004794)
Supplement: S1 File — Transcript 4 (CODES & THEMES by KU).pdf. Transcript 6 (CODES & THEMES by KU).pdf. Transcript 7 (CODES & THEMES, by KU).pdf. Transcript 8 (CODES & THEMES by KU).pdf. Transcript 9 (CODES & THEMES by KU).pdf. Transcript 10 (CODES & THEMES by KU).pdf. Transcript 11 (CODES & THEMES, by KU).pdf. Transcript 12 (CODES & THEMES by KU).pdf. Transcript 13 (CODES & THEMES by KU).pdf. Transcript 14 (CODED & THEMES by KU).pdf. Transcript 15_b (CODED & THEMES by KU). pdf. Transcript 16 (CODES & THEMES by KU).pdf. Transcript 17 (CODES & THEMES by KU).pdf. Transcript 18 (CODES & THEMES by KU).pdf. Transcript 19 (CODES & THEMES by HK).pdf. Transcript 20 (CODES & THEMES by HK).pdf. Transcript 21_b (CODES & THEMES by HK).pdfTranscript 22 (CODES & THEMES by HK).pdf. Transcript 25 (CODES & THEMES by HK).pdf. Transcript 27 (CODES & THEMES by HK).pdf. Transcript Sn1 (CODES & THEMES by RS).pdf Transcript Sn6 (pt3) (CODES & THEMES by RS).pdf. Transcript Sn15_a (CODES & THEMES by RS).pdf. Transcript SN17 (pt3) (CODES & THEMES by RS).pd. Transcript Sn21_a (CODES & THEMES by RS).pdf. (ZIP) [file pgph.0004794.s001.zip › Transcript 9 (CODES & THEMES by KU).pdf]

Text	Initial coding	Themes
**Transcription interview 9**<br>**Interviewee: XXX**<br>**SN-12**<br>**Interviewer: (MS), Research Assistant**<br>**Number of speakers :2**<br>**Time: 2.59pm**<br>**Length of interview recording: 20minutes 9 seconds**<br>**Date: 31/3/23**<br><br>**1. Interviewer [MS]: Perfect, so I've started recording. So the first thing I need to do is just go through a consent form with you. Did you have a look at the information sheet that I sent you? This one *shares information sheet on screen***<br>2. Interviewee [XXX]: Yes I did<br>**3. Interviewer [MS]: Have you had a read through it?**<br>4. Interviewee [XXX]: I read yeah *broken up speech*<br>5. *overlapping speech*<br>**6. Interviewer [MS]: Did you manage to have a read through it yeah?**<br>7. Interviewee [XXX]: Yes I did *broken up speech*<br>**8. Interviewer [MS]: Okay *overlapping speech*. Perfect and did you have a look through the consent form as well?**<br>9. Interviewee [XXX]: *broken up speech* not really<br>10. **Interviewer [MS]: You've not had you've not read through the consent form?**<br>11. Interviewee [XXX]: the consent okay I read about that yes I did *mumbled speech*<br>**12. Interviewer [MS]: Did you read the consent form yeah?**<br>13. Interviewee [XXX]: Yes I did I read *mumbled speech*<br>14. **Interviewer [MS]: Okay amazing *overlapping speech***<br>**15. Interviewer [MS]: Do you, are you happy do you consent to taking part in the study?**<br>16. Interviewee [XXX]: Yes its okay *broken up speech*<br>17. *overlapping speech*<br>18. **Interviewer [MS]: Great, so what I need to do is urm just because you can't physically it ,is it okay for me to put your initials in all of these boxes just to show that you consent to take part?**<br>19. Interviewee [XXX]: okay<br>20. **Interviewer [MS]: Is that okay? I can put your initials yeah?**<br>21. Interviewee [XXX]: yes you can		

22. **Interviewer [MS]: okay so what's is it *confirming initials* your initials is that right?**
23. Interviewee [XXX]: Yes *confirmed initials*
24. **Interviewer [MS]: Okay**
25. ***overlapping speech participant confirming initials***
26. **Interviewer [MS]: Like this?**
27. Interviewee [XXX]: *participant confirming initials*
28. **Interviewer [MS]: Is that correct yeah?**
29. Interviewee [XXX]: Yes *broken up speech*
30. **Interviewer [MS]: Yes and you're happy for me to um audio and video record this call?**
31. ***Silence for a few seconds***
32. **Interviewer [MS]: Yes? You're happy for me to**
33. Interviewee [XXX]: *overlapping speech* yes yes
34. **Interviewer [MS]: Yeah perfect**
35. Interviewee [XXX]: It's okay okay *mumbled speech*
36. **Interviewer [MS]: *cough* um if you want to not take part at anytime or want to stop the interview then just let me know um but overall you agree to take part so its *confirming name* yes? Can you see that on the screen? And today is the *confirming date* and then just because you can't sign it I need to put your intials in here as well is that okay?**
37. Interviewee [XXX]: Yes *broken up speech*
38. **Interviewer [MS]: yeah perfect so that's the consent form so you said you're happy to take part and I will send that to you um aft like later on ill be able to send that to you, just so you've got it**
39. Interviewee [XXX]: ***overlapping broken up speech by participant***   its okay
40. **Interviewer [MS]: email it you *broken up speech* okay?**
41. ***no response***
42. **Interviewer [MS]: perfect. Okay. Fab. Right so I just wanted to ask *broken up speech* are you using an airtime card for this call?**
43. Interviewee [XXX]: okay go ahead please go ahead
44. **Interviewer [MS]: *overlapping speech* are you using airtime, have you got an airtime card for this call?**
45. Interviewee [XXX]: sorry *unclear speech*
46. **Interviewer [MS]: are you using an airtime card for the call or just wifi? *cough***
47. Interviewee [XXX]: Im using myyy, im using my phone im using my phone
48. **Interviewer [MS]: okay not air, you've not got an airtime card?**
49. Interviewee [XXX]: no I not use it, I have a card

50. **Interviewer [MS]: mhmm, so so basically if you were using this an airtime card for this call you can submit it to *name of dr* for a refund but only if you're using an airtime card**		
51. Interviewee [XXX]: okay		
**52. Interviewer [MS]: okay?**		
53. Interviewee [XXX]: okay *line sounds broken up*		
**54. Interviewer [MS]: okay okay so im going to start the interview now. Em so its just some questions, just answer them the best that you can. If you've got any questions then just ask me okay?**		
55. Interviewee [XXX]: okay good *unclear speech*		
56. **Interviewer [MS]: okay so do you prescribe antibiotics to pregnant women?**		
57. Interviewee [XXX]: yes I do	**57. Prescribing antibiotics**	
**58. Interviewer [MS]: yes, how often do you prescribe them?**		
59. Interviewee [XXX]: *unclear speech* as the need arises. *unclear speech* not routine, I don't prescribe *unclear work* routinely	**59. Prescribe when needed**	**[1] PRESCRIBE ANTIBIOTICS**
60. *overlapping speech*		
**61. Interviewer [MS]: okay, how many times a week would you say that you prescribe antibiotics?**		
62. Interviewee [XXX]: sorry?		
**63. Interviewer [MS]: how many times a week do you think you prescribe antibiotics for?**		
**64. *computer background noise***		
65. Interviewee [IM}: mm antibiotics prescription I don't prescribe *unclear speech* as the need arises	**65. When needed**	
**66. Interviewer [MS]: okay *overlapping speech***		
67. Interviewee [XXX]: okay for example *unclear speech* prescribe antibiotics okay *unclear speech* once to a patient that needed this right?	**67. When needed**	
**68. Interviewer [MS]: mhmm okay how long have you been prescribing antibiotics for? How many years?**		
69. Interviewee [XXX]: mmm *unclear speech* how many years now *unclear speech* ermmm let me say for 18 years 17 to 18 years	**69. History of prescribing**	
70. **Interviewer [MS]: Okay great. Erm what are the 3 most common medical problems that you prescribe antibiotics for?**		
71. Interviewee [XXX]: okay the common urinary tract infection okay and lower urinary tract infection, so those are two commonest	**71. Medical conditions prescribed for**	
**72. Interviewer [MS]: Okay and do you use any guidelines when you've got to prescribe antibiotics?**		
73. Interviewee [XXX]: *unclear speech* guidelines yes. *unclear speech* prescription antibiotics we use	**73. Guidelines on prescribing (yes)**	

guidelines also based on imperial yeah baseline medical knowledge then guidelines yeah *unclear speech*		
**74. Interviewer [MS]: Great and em so where do find that pregnant women generally get their antibiotics from?**		
75. Interviewee [XXX]: sorry		
**76. Interviewer [MS]: where do you find that pregnant women generally get antibiotics from?**		
77. Interviewee [XXX]: where they get it from the hospital pharma if I where im working they would get it from the pharmacy	**77. Obtaining antibiotics (pharmacy)**	**[2] OBTAINING ANTIBIOTICS**
78. **Interviewer [MS]: Okay okay and do you ever find that pregnant women take antibiotics that havent been prescribed for them?**		
79. Interviewee [XXX]: sorry I didn't get you		
**80. Interviewer [MS]: do you ever know of any pregnant who have taken antibiotics that havent been prescribed for them?**		
81. Interviewee [XXX]: as in deh whether they take antibiotics that been prescribed to them? Is what you mean?		
**82. Interviewer [MS]: without without prescription yeah**	**83. Self-medication (ie., without prescription) is rare**	**[3] SELF-MEDICATION**
83. Interviewee [XXX]: mm okay some of them they took but its not common *speech broken up* I don't think its common that they take antibiotic without prescription its possible yes its possible		
**84. Interviewer [MS]: Have you seen that in your practice?**	**85/86. Direct observation (of self-medication)**	
85. Interviewee [XXX]: yes I have seen *unclear speech*		
**86. Interviewer [MS]: Okay *overlapping speech***		
87. Interviewee [XXX]: *unclear speech* yes yes I have seen		
**88. Interviewer [MS]: Okay, urm and do you know like pregnant women who have taken like herbal preparations or alternative medications instead of antibiotics?**	**89. Herbal self-medication (not common)**	**[4] HERBAL SELF-MEDICATION**
89. Interviewee [XXX]:mm that one is not common, that one not very common *mumbled speech* I say they do antibiotic self medication but herbal incase of		
**90. Interviewer [MS]: *cough***		
91. Interviewee [XXX]: errr not sure im not sure about that one		
92. **Interviewer [MS]: Okay. Dya not see it dya see it in your clinical practice? Or dya see It at work?**	**93. Herbal self-medication (uncommon in pregnancy)**	
93. Interviewee [XXX]: *unclear speech* taking herbal eh stuff in place of no I don't think ive had much experience with that *unclear speech* taking eh herbal *unclear speech* no its not common okay no ive seen that one outside pregnant but for pregnancy im not *unclear speech*		

94. **Interviewer [MS]: Okay**<br>95. Interviewee [XXX]: *overlapping speech*<br>**96. Interviewer [MS]: Okay. Em so you know we were saying about self-medication of antibiotics, so when the antibiotics haven't been prescribed. Em do you know of any methods that can identify when pregnant women self-medicate with antibiotics that haven't been prescribed? So any ways of identifying or detecting**<br>97. Interviewee [XXX]: *overlapping speech*<br>98. **Interviewer [MS]: that's happened**<br>99. Interviewee [XXX]: *unclear speech* yes, *unclear speech* when they're not close to the hospital, the *unclear word* nurse *unclear speech* they are not close to the hospital that's one, two sometimes buy antibiotics may be cheaper than going to the hospital paying for consultation and<br>100. *sound went off*<br>**101. Interviewer [MS]: hello? Hello? Hello? *name of dr* I can't hear you**<br>102. Interviewee [XXX]: *broken up speech*<br>**103. Interviewer [MS]: oh I can hear you now. Can you hear me? Hello?**<br>104. Interviewee [XXX]: hello?<br>**105. Interviewer [MS]: Hi can you hear me?**<br>**106. *no reply***<br>**107. Interviewer [MS]: Can you hear me? you might need to exit and then reenter the call. The signals, oh there we go. He has gone**<br>**108. Interviewer [MS]: Hello?**<br>109. Interviewee [XXX]: Hello *unclear speech* sorry<br>**110. Interviewer [MS]: *overlapping speech* You're back, don't worry don't worry don't worry um so it missed out some of what you were saying, so you were saying sometimes people cant afford antibiotics so they'll go somewhere or they've not got a prescription urm so dya wna carry on what you were saying. Sorry**<br>111. Interviewee [XXX]: okay I said 2 reasons why *unclear speech* when they see that they are not close to a hospital not be accessible so they don't have option than to than maybe go to a pharmacy shop a chemist or a nurse. Two they do not have enough money to come to hospital they feel that will be a cheaper alternative going to buy drugs meeting a non medical person<br>**112. Interviewer [MS]: mhmm**<br>113. Interviewee [XXX]: kay	**99. SM/cheaper than going to hospital. Proximity an issue**<br><br>**111. SM & lack of proximity to hospital**	**[1]**<br><br>**[1]**

**114. Interviewer [MS]: so if someone came into the hospital that was taking that was self medicating antibiotics that hadnt been prescribed. Do you know of any ways that you would identify that? How would you know?**<br>115. Interviewee [XXX]: *unclear speech* routinely we don't look for it. *unclear speech* interaction based on history and interaction with the patient. I know you can do some other pharmalogical to detect, for now we don't do it routinely in our centre. *unclear speech* we rely on our interaction with the patient<br>**116. Interviewer [MS]: Mhmm, Dya think it could be useful to have like a simple rapid test or questionnaire or tool that would help identify pregnant women who may be misusing antibiotics that we don't know about?**<br>117. Interviewee [XXX]: Yes that that would be good, that would be good okay that would be good but its also to other option would be also look at the the prescription of antibiotics the *unclear speech* that would also be very good if you can *unclear speech* rules to prescribe, who has assessed *unclear speech* going to help<br>118. **Interviewer [MS]: mhhm dya have any ways of how you would think maybe a tool or a test could work, dya have any ideas? About what might be a good idea?**<br>119. Interviewee [XXX]: yeah a tool can work we can have a standard tool eh *unclear speech* questionnaires *unclear speech* with the patients the pregnant women okay?<br>**120. Interviewer [MS]: mhmm**<br>121. Interviewee [XXX]: you know those people who have self medication antibiotics<br>**122. Interviewer [MS]: mhm**<br>123. Interviewee [XXX]: find out why they are doing it and trying to calculate that we also reduce *unclear speech*<br>**124. Interviewer [MS]: okay, so if such a test or a tool was available, would you be interested in using it?**<br>125. Interviewee [XXX]: yeah of course yes<br>**126. Interviewer [MS]: okay and would such a tool or a test or a questionnaire where do you think it would be best put in use? Like in antenatal care settings, or routine appointments, or A&E? Where dya think it would be good to have it?**<br>127. Interviewee [XXX]: *unclear speech* antenatal class, also be used during one of doctors consultations<br>**128. Interviewer [MS]: Mhmm, mhmm okay. Urm and then do you think that such a test would need to be**	**115. Detecting SM (they don't do it)**<br><br>**117. Detection (focus on prescription guidelines)**<br><br>**119. Using a questionnaire**<br><br>**121. to identify people SM**<br><br>**123. Why they are SM**<br><br>**127. Detection location (antenatal class)**	**[5] DETECTING SELF-MEDICATION (methods, tech, access..)**

**like mobile like easy to carry around not have to use electricity or dya not think it matters?**<br>129. Interviewee [XXX]: I don't get you<br>**130. Interviewer [MS]: so if we had a test or a tool, dya think it would be better if it was yahno easy to carry around and didn't need electricity or dya not think it really would matter?**<br>131. Interviewee [XXX]: * mumbled speech* don't think it would matter, don't think would matter carrying around no think its something you can drop at hospital<br>**132. Interviewer [MS]: mhmm**<br>133. Interviewee [XXX]: *unclear speech*<br>**134. Interviewer [MS]: What about needing to use electricity?**<br>135. Interviewee [XXX]: okay electricity, you can use electricity<br>136. *overlapping speech*<br>**137. Interviewer [MS]: Or internet**<br>138. Interviewee [XXX]: yeah you can use internet<br>139. *overlapping speech*<br>**140. Interviewer [MS]: right you don't**<br>141. Interviewee [XXX]: Its an option yes but *unclear speech* you make it accessible to all of them yes all the patients okay. If you have internet you have to make it accessible not only the doctor the health care professional *unclear speech* important that the woman have access *unclear speech* if not *unclear speech*<br>**142. Interviewer [MS]: mhmm mhmm okay em have you ever come across any methods or guidelines that have looked at identifying the side effects of antibiotic self-medication in pregnant women?**<br>143. Interviewee [XXX]: *mumbling speech* there is a lot of guidelines okay. They have a lot of guidelines okay there are some okay<br>**144. Interviewer [MS]: mhmm**<br>145. Interviewee [XXX]: sooo some that they try adverse effects of antibiotics *unclear speech*<br>**146. Interviewer [MS]: mhmm**<br>147. Interviewee [XXX]: *unclear speech* mainly ive not really seen much *unclear word* because *unclear speech* adverse reactions<br>**148. Interviewer [MS]: mhmm**<br>149. Interviewee [XXX]:anti antibiotics okay its not something we prescribe routinely<br>**150. Interviewer [MS]: mhmm**<br>151. Interviewee [XXX]: okay, my practice what we prescribe routinely *unclear speech* eye drops mhmm<br>**152. Interviewer [MS]: mhmm mhmm**	**131. Portability, electricity**<br>**135. Electricity**<br>**138. Using internet**<br>**141. Accessibility to all (patient, doctor etc).**<br>**143. Side effects of SM & guidelines**<br>**147. Side effects (not seen)**	**[7] DETECTING SELF-MEDICATION (CLINICAL, side effects)**

153. Interviewee [XXX]: that’s all we prescribe routine		
154. **Interviewer [MS]: Okay so we know sometimes antibiotics can cause side effects em dya think the presence of side effects is clear when someones taking antibiotics? Dya think its clear when someones got side effects?**		
155. Interviewee [XXX]: sorry I don’t get you		
156. **Interviewer [MS]: so we know that antibiotics can cause side effects like stomach upset**		
157. Interviewee [XXX]: yes *overlapping speech*		
**158. Interviewer [MS]: and things like that, dya think its clear when that’s from antibiotics?**		
159. Interviewee [XXX]: I don’t get the question its not clear I don’t get it *mumbling*		
**160. Interviewer [MS]: so just say when someone has anti em when someone has side effects from antibiotics**		
161. Interviewee [XXX]: mm	**163. Side effects (patient complaints)**	
**162. Interviewer [MS]: Is that clear? Is it obvious to you?**		
163. Interviewee [XXX]: yess if someone come and complain okay for example there is a lady *unclear speech* and some other anti yess it can be okay *unclear speech* antibiotics if they have reactions *unclear speech* you can always say for example when you have complains you can *unclear word* about antibiotics, other drugs not for the antibiotics		
**164. Interviewer [MS]: mhmm**		
165. Interviewee [XXX]: *unclear speech*	**167. Side effects (unclear from antibiotics)**	
**166. Interviewer [MS]: mhmm but when its from antibiotics is it quite clear?**		
167. Interviewee [XXX]:sometimes its clear sometimes its not clear depending on the antibiotics		
**168. Interviewer [MS]: okay *overlapping***		
169. Interviewee [XXX]: Patient is taking *overlapping*		
**170. Interviewer [MS]: yeah that’s fine. Em and do you know have you ever seen any pregnant women develop side effects from self medicating with antibiotics?**	**171. Side effects from SM (can’t remember)**	
171. Interviewee [XXX]: antibiotics *broken up speech* try to remember cant remember any body cant recall anyone		
172. **Interviewer [MS]: Okay that’s okay and do you know its similar to other questions but do you know of any methods or guidelines or protocols to manage em antibiotic self medication in pregnant women?**	**173. Guidelines on SM with antibiotics (not sure)**	**[6] GUIDELINES**

173. Interviewee [XXX]: guideline? Not sure that one any protocol *unclear speech* antibiotic self medication not sure *unclear speech* common one mmm<br>**174. Interviewer [MS]: okay**<br>175. Interviewee [XXX]: *overlap* yes I read about it I really don’t know because its not the case ive not seen quite a lot them *unclear* self medication so im not really *unclear speech* ive not used that much mm<br>**176. Interviewer [MS]: Okay that’s fine and theres a specific area where pregnant women who self medicate with antibiotics and may develop memory loss, or forgetfullness, do you know any treatment options or management options about what you would do if that happened to someone?**<br>177. Interviewee [XXX]: *unclear speech* first thing I would do is find antibiotics stop it<br>**178. Interviewer [MS]: mhmm**<br>179. Interviewee [XXX]: okay and then when you stop it then *unclear speech* some of them may be reversible *unclear speech* once they stop *unclear speech* may have to give *unclear speech* refer to clinical pharmacologist okay who will manage them *mumbled speech*<br>**180. Interviewer [MS]: okay great great I mean that’s all my questions so thank you that was very helpful. Do you have any questions?**<br>181. Interviewee [XXX]: okay no not really<br>**182. Interviewer [MS]: No okay that’s perfect, thank you so much for taking the time to take part um we really really appreciate It and appreciate you answering all the questions um so that’s everything with me so id already emailed you the before the consent form and participant information sheet ill send you the completed consent form ill email it you. Um and that’s everything, as we said before if you did use airtime you could of sent urm submitted it to *name of dr* for a refund but you said that you’ve just you’ve not used airtime**<br>183. Interviewee [XXX]: no<br>**184. Interviewer [MS]: so so that’s okay, um but yeah thank you that’s everything if you’ve got any questions you can email me or you’ve got my number you can message me**<br>185. Interviewee [XXX]: okay okay<br>**186. Interviewer [MS]: is that okay?**<br>187. Interviewee [XXX]:its okay *mumbling unclear speech* okay	**175. Guidelines on SM (not seen, not used)**<br><br>**177. Neurological effects (stop antibiotic [misuse])**<br>**179. Reversing symptoms. Referring to pharmacologist**	**[7]**

**188. Interviewer [MS]: thank you so much for your time I really really appreciate it**<br>189. Interviewee [XXX]: youre welcome<br>**190. Interviewer [MS]: thank you. Have a good rest of the day**<br>191. Interviewee [XXX]:you too bye<br>**192. Interviewer [MS]: bye**		
