# Supplementary material for: Advancing the safe motherhood initiative: A qualitative and sentiment analysis of local physician’s perspectives on antibiotic self-medication during pregnancy in a low- and middle-income country
Source: PLOS Glob Public Health. 2025 Sep 12;5(9):e0004794. doi: 10.1371/journal.pgph.0004794 (PMC12431270; doi:10.1371/journal.pgph.0004794)
Supplement: S1 File — Transcript 4 (CODES & THEMES by KU).pdf. Transcript 6 (CODES & THEMES by KU).pdf. Transcript 7 (CODES & THEMES, by KU).pdf. Transcript 8 (CODES & THEMES by KU).pdf. Transcript 9 (CODES & THEMES by KU).pdf. Transcript 10 (CODES & THEMES by KU).pdf. Transcript 11 (CODES & THEMES, by KU).pdf. Transcript 12 (CODES & THEMES by KU).pdf. Transcript 13 (CODES & THEMES by KU).pdf. Transcript 14 (CODED & THEMES by KU).pdf. Transcript 15_b (CODED & THEMES by KU). pdf. Transcript 16 (CODES & THEMES by KU).pdf. Transcript 17 (CODES & THEMES by KU).pdf. Transcript 18 (CODES & THEMES by KU).pdf. Transcript 19 (CODES & THEMES by HK).pdf. Transcript 20 (CODES & THEMES by HK).pdf. Transcript 21_b (CODES & THEMES by HK).pdfTranscript 22 (CODES & THEMES by HK).pdf. Transcript 25 (CODES & THEMES by HK).pdf. Transcript 27 (CODES & THEMES by HK).pdf. Transcript Sn1 (CODES & THEMES by RS).pdf Transcript Sn6 (pt3) (CODES & THEMES by RS).pdf. Transcript Sn15_a (CODES & THEMES by RS).pdf. Transcript SN17 (pt3) (CODES & THEMES by RS).pd. Transcript Sn21_a (CODES & THEMES by RS).pdf. (ZIP) [file pgph.0004794.s001.zip › Transcript 10 (CODES & THEMES by KU).pdf]

Text (Interview Transcript)	Initial Codes	Themes/Categories
**Transcription interview 10**<br>**Interviewee: XXX**<br>**SN-41**<br>**Interviewer: (MS), Research Assistant**<br>**Number of speakers : 2**<br>**Other Attendees: Dr Kanayo Umeh (KU), Principal Investigator**<br>**Time: 2.31pm**<br>**Length of interview recording: 21 min 15 seconds**<br>**Date: 6.4.23**<br><br>1. **Interviewer [MS]: okay *clears throat*, the first thing I need to do is ive sent you a consent form and information sheet before, have you had a chance to have a read through it?**<br>2. Interviewee [XXX]: Yes, ive gone through it<br>3. **Interviewer [MS]: okay let me just *talking to self*, so so this is the information sheet, so you've had a read through this yeah?**<br>4. Interviewee [XXX]: okay<br>5. **Interviewer [MS]: did you have a read through it? The information sheet**<br>6. Interviewee [XXX]: *overlapping speech* yes I have done that<br>7. **Interviewer [MS]: perfect and then this is the sorry the consent form I just need to go through this *mumbling to self* sorry about that *talking to self* right. So did you have a read through the consent form as well?**<br>8. Interviewee [XXX]: yes<br>9. **Interviewer [MS]: yeah so do you consent to all of the points? Are you happy to take part in the interview?**<br>10. Interviewee [XXX]: im happy to take part<br>11. **Interviewer [MS]: perfect so what we do is you said you already had a read through all this yes**<br>12. Interviewee [XXX]: yeah *slightly unclear, broken up*<br>13. **Interviewer [MS]: em so what we do is I just because you cant sign it were just putting your initials in every box is that okay? *background noise* So ill do that for you is that okay?**		

14. Interviewee [XXX]: okay okay by me<br>**15. Interviewer [MS]: whats your initials?**<br>16. Interviewee [XXX]: *says initials*<br>**17. Interviewer [MS]: Like that?**<br>18. Interviewee [XXX]: *repeats initials*<br>**19. Interviewer [MS]: like that? Is that right? *confirms initials* like that yeah?**<br>20. Interviewee [XXX]: yeah<br>**21. Interviewer [MS]: are you happy for it to be audio and video recorded? Yeah?**<br>22. Interviewee [XXX]: yeah<br>**23. Interviewer [MS]: perfect, so you can turn off your camera or anything at anytime em its completely up to you and we can stop the interview at anytime you want, so you agree to take part yeah?**<br>24. Interviewee [XXX]: yeah<br>**25. Interviewer [MS]: perfect and then your last is your last name whats your last name? Is it *asks to confirm name* is that your first name?**<br>26. Interviewee [XXX]: *confirms first name*<br>**27. Interviewer [MS]: and your last name?**<br>28. Interviewee [XXX]: *confirms last name*<br>**29. Interviewer [MS]: *confirms last name* like that yeah?**<br>30. Interviewee [XXX]: *confirms spelling of last name*<br>**31. Interviewer [MS]: that? Is that right? Ive put it on the form**<br>32. Interviewee [XXX]: *reading name*<br>**33. Interviewer [MS]: Is that right?**<br>34. Interviewee [XXX]: Yeah<br>**35. Interviewer [MS]: and then in the box just as you cant physically sign the form, we just put your initials which is *talking to self* so we put your intials which you said was *says initials* yeah?**<br>36. Interviewee [XXX]: yeah<br>**37. Interviewer [MS]: perfect, so at a later date I will send you this form just so you've got it for your records okay?**<br>38. Interviewee [XXX]: okay<br>**39. Interviewer [MS]: um but yeah that's perfect so we can start the interview now um and if you have any questions then let me know em but yeah thank you for reading the consent form we started its very helpful perfect. So now im just gno ask some questions okay?**<br>40. Interviewee [XXX]: *background noise noted*		

**41. Interviewer [MS]: so answer them to the best of your ability and if you've got any questions just let me know so first avail are you using airtime for this call?**<br>42. Interviewee [XXX]: pardon?<br>**43. Interviewer [MS]: are you using airtime for this call?**<br>44. Interviewee [XXX]: okay<br>**45. Interviewer [MS]: are you using airtime or wifi?**<br>46. Interviewee [XXX]: yeah wifi<br>**47. Interviewer [MS]: okay so that's fine if you're using airtime then we say you can forward the um like receipt to *name of a dr* but youre using wifi so its fine but yeah so im gno start, so do you prescribe antibiotics to pregnant women?**		
48. Interviewee [XXX]: occasionally pending the illness im treating	**48. Antibiotics prescribing (occasionally)**	[1] PRESCRIBING
**49. Interviewer [MS]: mhmm. how often long have you been a prescriber for?**		
50. Interviewee [XXX]: ee it depends on what we are treating but most times *unclear speech*	**50. Depends on treatment**	
**51. Interviewer [MS]: okay. How long have you been prescribing antibiotics for?**		
52. Interviewee [XXX]: for more than 10 years now	**52. Prescribing (years)**	
**53. Interviewer [MS]: perfect. What are the 3 most common medical problems that you prescribe antibiotics for?**		
54. Interviewee [XXX]: most times its urinary tract infection	**54. Prescribed (UTI)**	
**55. Interviewer [MS]: mhmm**		
56. Interviewee [XXX]: and upper respiratory tract infection	**56. Prescribed (RTI)**	
**57. Interviewer [MS]: mhmm**		
**58.** Interviewee [XXX]:mm that's that's the two most common problem that come from for which antibiotics are given most times. Then other things are dependent on what is present with but most times its urinary tract infection and upper respiratory tract infection	**58. Prescribed (UTI & RTI common illnesses)**	
59. **Interviewer [MS]: Okay. So how many times a week would you say you prescribe antibiotics? For pregnant women**		
60. Interviewee [XXX]: mmm in a way maybe three times	**60. Prescribe (frequency)**	
**61. Interviewer [MS]: mhmm okay. Do you have any guidelines that you use when prescribing antibiotics?**		

62. Interviewee [XXX]: actually, where I practice there is no stipulationed guidelines. We look at the based on the safety of the antibiotics and the duration of the pregnancy.	**62. Prescribe (guidelines)**	
**63. Interviewer [MS]: Okay**		
64. Interviewee [XXX]: yeah so for instance in first treimester at the first 13 weeks of pregnancy we try as much as possible to avoid the use of antibiotics okay? If we must use we use the ones that have good safety in pregnancy, most times the penicillin	**64. Prescribing (stage of pregnancy)**	
**65. Interviewer [MS]: mhmm okay**		
66. Interviewee [XXX]: *overlapping speech* Quinalonce are never used. *unclear speech* in the first trimester *unclear speech* tetrogenic features *unclear speech* usually used when there is need to prescribe antibiotics	**66. Prescribing (stage of pregnancy)**	
**67. Interviewer [MS]: okay. Where do you find that women generally get antibiotics from, like hospital pharmacy or health centres or clinics, where do they normally get it from?**		
68. Interviewee [XXX]: yeah most times they get it from pharmacy or hospital I work with, but occasionally they may request that the prescription is given to them to purchase it in any of the other pharmacies especially when they look at the cost because it looks more expensive within the hospital than if they purchase it outside	68. Obtaining antibiotics (pharmacy, hospital, elsewhere)	[2] OBTAINING
**69. Interviewer [MS]: mhmm *overlapping***		
70. Interviewee [XXX]: or they get it from the hospital pharmacy where we are sure of the potency of the antibiotics we are giving	70. Obtaining antibiotics (hospital pharmacy)	
**71. Interviewer [MS]: okay, so are you aware of any pregnant women who take antibiotics that havent been prescribed for them?**		
72. Interviewee [XXX]: pardon?		
**73. Interviewer [MS]: are you aware of sometimes pregnant women, do they ever take antibiotics that havent been prescribed for them?**		
74. Interviewee [XXX]: yeah yeah sometimes they they do self prescription, especially where we work I work in Nigeria. Most times some patients do self prescription *unclear word* before coming to the hospital to show you the antibiotics they are taking, but we try as much as possible to discourage them from doing self prescription because of its harmful effects	**74. Self-medication (occurs b4 coming to hospital) (discouraged)**	[3] SELF-MEDICATION

**75. Interviewer [MS]: mhmm. Do you see that it happens a lot?**<br>76. Interviewee [XXX]: yeah they do they do they do<br>**77. Interviewer [MS]: okay where do they normally get it from?**<br>78. Interviewee [XXX]: they get it from site pharmacy<br>**79. Interviewer [MS]: okay**<br>80. Interviewee [XXX]: or *unclear word* partent dealers<br>**81. Interviewer [MS]: okay**<br>82. Interviewee [XXX]: *unclear* partent dealers<br>**83. Interviewer [MS]: so are you aware of pregnant women who might take like herbal preparations or alternative medications that could work like antibiotics?**<br>84. Interviewee [XXX]: yeah! They do they do they take local concoctions some of them take local concoctions<br>85. *overlapping speech*<br>**86. Interviewer [MS]: do you have any examples of any that you know of?**<br>87. Interviewee [XXX]: eeerrrr I don't know if youre able to know the names because the names are mentioned in the local language okay? Like eh habaeaters, mmm err goguru a lot of names they are they are been mentioned based on the local dialect of the people, but the routine there are the *unclear word* herbs<br>**88. Interviewer [MS]: mhmm**<br>89. Interviewee [XXX]: roots and herbs<br>**90. Interviewer [MS]: mhmm**<br>91. Interviewee [XXX]: sometimes we *unclear speech* but we try to discourage, and we know it's quite difficult to discourage people of what they've practised over a long period of time especially when they don't see the danger that is coming before them so we try as much as possible to let them understand that they are not supposed to take unorthodox medicine especially when they are pregnant<br>92. **Interviewer [MS]: yeah Yeah definitely. Do you know of any methods that can like detect self-medication of antibiotics in pregnant women?**<br>93. Interviewee [XXX]: pardon? I didn't get it<br>94. **Interviewer [MS]: Do you know of any methods that might detect when a woman self-medicating in pregnancy?**	**76. SM (common)**<br>**78. SM (local pharmacy)**<br>**84. Herbal self-medication (yes, local concoctions)**<br>**87. Herbal self-medication (examples, local culture)**<br>**91. Herbal SM (discouraged, difficult problem)**	[4] HERBAL SELF-MEDICATION

95. Interviewee [XXX]: no no no no I don't we don't normally do that<br>**96. Interviewer [MS]: No so you don't know any methods that might detect, so how would you know that someone was self medicating with antibiotics?**<br>97. Interviewee [XXX]: we rely on history<br>**98. Interviewer [MS]: mhmm**<br>99. Interviewee [XXX]: yeah when you tell them when you get their confidence or ask them they will tell you whether they've been taking something not prescribed by *unclear speech* by a doctor. Some of them are openly tell you especially when you don't look judgemental<br>**100. Interviewer [MS]: mmm**<br>101. Interviewee [XXX]: and we rely so much on the history of the patient presenting to us<br>**102. Interviewer [MS]: mmmm**<br>103. Interviewee [XXX]: in terms of physicology we don't normally run physicology tests here because sometimes they the instrumental *unclear speech* are not avaliable, unless in rare cases especially when the person has come down with a lot of complications so *unclear speech* tests, but most times in general we don't we rely on what is provided to us<br>**104. Interviewer [MS]: fine**<br>105. Interviewee [XXX]: *overlapping speech* history taking<br>**106. Interviewer [MS]: fine. Do you think it could be useful to have a like a rapid test or lab test or questionnaire that might help identify pregnant women who are misusing antibiotics?**<br>107. Interviewee [XXX]: it will be very very helpful especially if it is less invasive<br>**108. Interviewer [MS]: mhmm**<br>109. Interviewee [XXX]: you can get one maybe one that you can use your *unclear word* or use less invasive procedure that can detect if someone *broken up speech*<br><br>110. **Interviewer [MS]: Mhmm, so if such a tool was available, would you be interested in using it?**<br>111. Interviewee [XXX]: oh I will I will<br>**112. Interviewer [MS]: *quite laugh***	**93. Detecting antibiotic SM (not done)**<br><br>**97. Detection (use patient history)**<br>**99. Detection (direct questioning, responses)**<br><br>**101. Detection (patient history)**<br><br>**103. Detection (physiological [clinical] testing)**<br><br>**107. Detection (utility of rapid test or questionnaire)**<br>**109. Detection (non-invasive)**<br><br>**111. Detection tool (using it)**	[5] DETECTING SELF-MEDICATION (METHODS…)

113. Interviewee [XXX]: I will and some of my some of my other colleagues will also will cause sometimes you sometimes some of the patients will tell you the truth *unclear speech*	**113. Detection tool (using it, colleagues)**	
**114. Interviewer [MS]: mm**		
115. Interviewee [XXX]: eh so have something less invasive can do within few minutes to get the result. That will be very very helpful	**115. Detection (non-invasive, quick results)**	
**116. Interviewer [MS]: Fine so do you think such a tool would be useful in like antenatal care settings, or during routine appointments, or in A&E? Like where do you think it will be most useful?**		
117. Interviewee [XXX]: yeah during antenatal visits especially at the booking visit	**117. Detection tool (utility, antenatal settings)**	
**118. Interviewer [MS]: mhmm**		
119. Interviewee [XXX]: Okay the first time you are seeing them I think its good, because you know some of them have to take some local concoction in order to get pregnant	**119. Detection (on first visit for antenatal care)**	
**120. Interviewer [MS]: mhmm**		
121. Interviewee [XXX]: so if you test them at the point of booking, the first time they are meeting them in the hospital that would be wonderful and then you may do that at a regular interval maybe every 3 months or so and until they *unclear speech*	**121. Detection (first visit, regular intervals)**	
**122. Interviewer [MS]: mhmm. Do you think it would be useful for like such a test to be mobile or remote or easy to use without having to use like electricity or internet?**		
123. Interviewee [XXX]: exactly exactly *overlap* because of the *unclear speech* of our supplies especially in this part of the world in Africa don't have steady light as in Europe okay *unclear speech*	**123. Detection tool (ease of use important)**	
**124. Interviewer [MS]: Do you have any ideas of how that would work?**		
125. Interviewee [XXX]: yeah what what happen if such if there is something like that what would you subjected to a local test or local research to invalidate it within the community you want to use it on and then and then try to get the hospital management to approve this use within the patient they see within the hospital and especially if there is no untoward effect to the patient less invasive I think they will	**125. Detection tool (need for community feedback, approval)**	

background noise interviewer side approval to use on their patient		
**126. Interviewer [MS]: mhmm, sorry my em dog is just choking on something *laughs* I don’t know what it is. Anyway sorry its just my dog**		
127. Interviewee [XXX]: whats the name		
**128. Interviewer [MS]: *says name of dog***		
129. Interviewee [XXX]: oh that’s good		
**130. Interviewer [MS]: sorry shes just choking on something so em have you come across any methods or guidelines which could help detect side effects of antibiotic self-medication in pregnant women? *background noise***		
131. Interviewee [XXX]: Im not quite sure if I have seen one unless you look at the some clear side effect of some antibiotic, there are some antibiotic with specific side effect when you see it like a skin reaction or *unclear speech* when you see it you may prove whether they have taken such an antibiotic *unclear speech* come down with such reaction but in terms of guidelines we follow in our country we don’t really practice based on the guidelines we follow but I feel it will be helpful if there are guidelines and it is now integrated into the practice of the people	**131. Guidelines on detecting SM via side effects (no guidelines)**	[6] GUIDELINES
**132. Interviewer [MS]: mmm *background noise***		
133. Interviewee [XXX]: I feel		
**134. Interviewer [MS]: so**		
135. Interviewee [XXX]: some clear clinical *unclear speech*		
**136. Interviewer [MS]: mhmm mhmm definitely. So you know antibiotics can sometimes cause side effects like stomach upset or a rash, *overlapping speech participant*, do you think the presence of such side effects in a patient is obvious you know that someones taking antibiotics?**		
137. Interviewee [XXX]: yeah	**137. Side effects, evidence of SM**	[7] DETECTING SELF-MEDICATION (SIDE EFFECTS)
**138. Interviewer [MS]: do you think that’s obvious? Like what kind of examples have you seen?**		
139. Interviewee [XXX]: yeah for instance let me give you an example, some some people react to penicillins	**139. Yes, side effects of penicillin, evidence of SM**	
**140. Interviewer [MS]: mhmm**		
141. Interviewee [XXX]: okay like *unclear speech* and em *unclear word* Johnsons		

disease, *unclear speech* syndrome or some of these skin reactions mucosal reaction of some antibiotics. When we will see this, it is also a pointer that this person may have taken something not *unclear speech* prescribe or even if its not *unclear speech* prescribe it can *unclear speech* reaction	**141. Side effects of penicillin, evidence of SM**	
**142. Interviewer [MS]: mmm**		
143. Interviewee [XXX]: *unclear speech* so when we see that we try to *unclear speech*		
**144. Interviewer [MS]: mhmm mhmm exactly and have you ever seen any pregnant women that have had side effects of antibiotic self-medication?**		
145. Interviewee [XXX]: yeah yes ive seen a lot of *unclear speech*	**145. Side effects (observed in patients)**	
**146. Interviewer [MS]: so when they've taken it without a prescription? They've had side effects?**		
147. Interviewee [XXX]: *unclear speech* let me just tell you let me just shock you most times our people optimise the outside pharmacy before coming to the hospital	**147. SM/pharmacy optimised, prior to hospital visit**	[3] SELF-MEDICATION (source, motivation…)
**148. Interviewer [MS]: mhmm**		
149. Interviewee [XXX]: it's a *unclear speech*		
**150. Interviewer [MS]: mhmm mhmm**		
151. Interviewee [XXX]: its not difficult to understand why you know here there is no universal health coverage	**151. Cost of medication (reason for SM)**	
**152. Interviewer [MS]: mhmm mhmm**		
153. Interviewee [XXX]: people pay out of pocket	**153. Cost of medication, OOP expenses (reason for SM)**	
**154. Interviewer [MS]: mhmm**		
155. Interviewee [XXX]: okay and as a result they tend to go where it will be cheaper for them	**155. Cost of medication (go where it is cheaper)**	
**156. Interviewer [MS]: yeah**		
157. Interviewee [XXX]: okay unless the signs and symptoms the problem they have are not that big thing		
**158. Interviewer [MS]: mhmm**		
159. Interviewee [XXX]: they will *unclear speech* go to the hospital		
**160. Interviewer [MS]: mhmm mhmm yeah**		
161. Interviewee [XXX]: *overlapping speech* they tend to *unclear speech* I can assure you that		
**162. Interviewer [MS]: mmm**		

163. Interviewee [XXX]: *unclear speech* at the end of it		
**164. Interviewer [MS]: yeah yeah that's good erm do you know any methods or guidelines or protocols that look at antibiotic self medication in pregnant women?**		
165. Interviewee [XXX]: There is no local one within where I work	**165. Guidelines on SM in preg women (none)**	[6] GUIDELINES (b)
**166. Interviewer [MS]: mhmm**		
167. Interviewee [XXX]: *unclear speech*		
**168. Interviewer [MS]: mhmm so its just kind of from experience**		
**169. *overlapping speech***		
170. Interviewee [XXX]: the guide we normally use is the FGA FGA drug *unclear words* in pregnancy okay	**170. Guidelines used**	
**171. Interviewer [MS]: mhm**		
172. Interviewee [XXX]: so *unclear speech* so we look at it *unclear speech*		
**173. Interviewer [MS]: mhmm**		
174. Interviewee [XXX]:okay, which one the benefit outweights the side effect okay?	**174. Cost-benefit analysis (as guideline?)**	
**175. Interviewer [MS]: mhmm**		
176. Interviewee [XXX]: so that's what guides us most times when we prescribe	**176. Cost-benefit (as guidance, for prescribing [not SM]?)**	
**177. Interviewer [MS]: mhmm mhmm yeah**		
178. Interviewee [XXX]: *overlapping speech* but for indetail written up guidelines		
**179. Interviewer [MS]: mhmm**		
180. Interviewee [XXX]: for antibiotic prescription in pregnancy, when It comes to my centre we don't have that	**180. No guidelines (on prescribing)**	
181. **Interviewer [MS]: mmm mmm yeah**		
182. Interviewee [XXX]: but *broken up speech* have workable guideline that can be a pride within our people	**182. Guidelines (as source of pride)**	
**183. Interviewer [MS]: definitely, definitely. So this question is more specific to a certain area. So sometimes you have pregnant women who self medicate with antibiotics, who might develop like signs of memory loss, or forgetfullness, do you know of any management signs oh options, and what would you do in those situations?**		
184. Interviewee [XXX]: yeah I generally when we have that the first thing *clears throat* the first thing we do to check and find out the medicating antibiotics	**184. Neurological effects from SM (check antibiotic use)**	[7] SIDE EFFECTS

**185. Interviewer [MS]: mhmm**		
186. Interviewee [XXX]: and when we discover that the first thing is taking any drug that have such side effects what we normally do is to discontinue its use	**186. Neurological effects (discontinue medication)**	
**187. Interviewer [MS]: mhmm**		
188. Interviewee [XXX]: we ask the patient to discontinue its use of the drug	**188. Neurological effects (discontinue)**	
**189. Interviewer [MS]: mhmm**		
190. Interviewee [XXX]: and then treat and then this treat this specific side effect they are presenting with, for instance having *unclear word*	**190. Neurological effects (treatment)**	
**191. Interviewer [MS]: mhmm**		
192. Interviewee [XXX]: we give them anti puretic agent	**192. Neurological effects (treatment)**	
**193. Interviewer [MS]: mhmm**		
194. Interviewee [XXX]: if theyre having vomiting we give them anti emetic	**194. Neurological effects (treatment)**	
**195. Interviewer [MS]: mhmm**		
196. Interviewee [XXX]: if they are having skin reaction we may give them something like a low dose steriods to stabilise the membrane *unclear speech* most times discontinue the offending agent they get	**196. Neurological effects (treatment)**	
**197. Interviewer [MS]: mhmm**		
198. Interviewee [XXX]: the whole thing here is to discontinue the offending agent and then treat symptomatically	**198. Neurological effects (discontinue, symptoms)**	
199. **Interviewer [MS]: mhmm mhmm so if they were having memory loss you would treat it symptomatically? Based on *unclear speech***		
200. Interviewee [XXX]: most the times memory loss is transient, normally *unclear speech* especially when they *unclear speech* problem	**188. Neurological effects (transient)**	
**201. Interviewer [MS]: mhmm mhmm perfect that's fine that's all my questions so thank you so much it was really interesting and thank you for taking the time I really really appreciate it. Dya have any questions about anything?**		
202. Interviewee [XXX]: no I don't have questions but I feel that em these these interviews is really revealing and eh I I will be pleased to participate in the research and see how it can help our people, especially the local protocol and guidelines *unclear speech*		

**203. Interviewer [MS]: definitely definitely and when weve finished the study we should be able to share the findings so when weve finished everything so we can keep you updated and yeah I really really appreciate you taking part and yeah if you've got any questions you've got my number you've got my email um but yeah thank you so much I really appreciate it**<br>204. Interviewee [XXX]: thank you very much<br>**205. Interviewer [MS]: and sorry for the sound disruption**<br>206. Interviewee [XXX]: *overlapping speech*<br>**207. Interviewer [MS]: normally shes fine but shes being weird**<br>208. Interviewee [XXX]: *laughs*<br>**209. Interviewer [MS]: so yeah thank you so much *laughs***<br>210. Interviewee [XXX]: okay bye bye<br>**211. Interviewer [MS]: thank you have a good rest of the day**<br>212. Interviewee [XXX]: thank you<br>**213. Interviewer [MS]: thanks bye**		
