# Supplementary material for: Advancing the safe motherhood initiative: A qualitative and sentiment analysis of local physician’s perspectives on antibiotic self-medication during pregnancy in a low- and middle-income country
Source: PLOS Glob Public Health. 2025 Sep 12;5(9):e0004794. doi: 10.1371/journal.pgph.0004794 (PMC12431270; doi:10.1371/journal.pgph.0004794)
Supplement: S1 File — Transcript 4 (CODES & THEMES by KU).pdf. Transcript 6 (CODES & THEMES by KU).pdf. Transcript 7 (CODES & THEMES, by KU).pdf. Transcript 8 (CODES & THEMES by KU).pdf. Transcript 9 (CODES & THEMES by KU).pdf. Transcript 10 (CODES & THEMES by KU).pdf. Transcript 11 (CODES & THEMES, by KU).pdf. Transcript 12 (CODES & THEMES by KU).pdf. Transcript 13 (CODES & THEMES by KU).pdf. Transcript 14 (CODED & THEMES by KU).pdf. Transcript 15_b (CODED & THEMES by KU). pdf. Transcript 16 (CODES & THEMES by KU).pdf. Transcript 17 (CODES & THEMES by KU).pdf. Transcript 18 (CODES & THEMES by KU).pdf. Transcript 19 (CODES & THEMES by HK).pdf. Transcript 20 (CODES & THEMES by HK).pdf. Transcript 21_b (CODES & THEMES by HK).pdfTranscript 22 (CODES & THEMES by HK).pdf. Transcript 25 (CODES & THEMES by HK).pdf. Transcript 27 (CODES & THEMES by HK).pdf. Transcript Sn1 (CODES & THEMES by RS).pdf Transcript Sn6 (pt3) (CODES & THEMES by RS).pdf. Transcript Sn15_a (CODES & THEMES by RS).pdf. Transcript SN17 (pt3) (CODES & THEMES by RS).pd. Transcript Sn21_a (CODES & THEMES by RS).pdf. (ZIP) [file pgph.0004794.s001.zip › Transcript 11 (CODES & THEMES, by KU).pdf]

Text	Initial codes	Themes
**Transcription interview 11**<br>**Interviewee: XXX**<br>**SN- 40**<br>**Interviewer: (MS), Research Assistant**<br>**Number of speakers : 2**<br>**Time: 7.02pm London time**<br>**Length of interview recording: 27 minutes 51 seconds**<br>**Date: April 6th 2023**<br><br>1. **Interviewer [MS]: *background noise* perfect, so I'm *name of interviewer* nice to meet you. Thank you so much for taking the time to join us *sound breaking up* interview um we really appreciate it *confirms name of interviewee pronounced correct***<br>2. Interviewee [XXX]: *laughs* its *confirms how to pronounce name*<br>3. **Interviewer [MS]: *confirms name of interviewee* okay, perfect thank you so much so the first thing I need to do with you is just go through *sound breaking up* a consent form with you, is that okay? It's the one that I sent to you urm had you**<br>4. Interviewee [XXX]: *overlapping speech* yes<br>5. **Interviewer [MS]: had a chance to read through it already or no**<br>6. Interviewee [XXX]: yeah I did I did I read through it *speech quite quiet*<br>7. **Interviewer [MS]:perfect so actually first of all, um *clears throat, speaks quietly to self* so I sent you as well a participant information sheet did you have a chance *slight pause silence* *speaking to self* *broken up speech***<br>8. Interviewee [XXX]: Sorry I didn't hear that<br>9. **Interviewer [MS]: Did you have a chance to read through the participant information sheet as well?**<br>10. Interviewee [XXX]: yeah uh I read the pages *mumbled speech*<br>11. **Interviewer [MS]: fine, so you've had a read through that perfect *overlapping speech noted* um and then that's great that's really helpful um and then the consent form**<br>12. Interviewee [XXX]: mm		

13. **Interviewer [MS]: *background noise* right so you've had a read through this consent form yeah?**
14. Interviewee [XXX]: Yeah I did
15. **Interviewer [MS]: perfect, are you happy with it all, do you say yes to all of the points?**
16. Interviewee [XXX]: mmm yes I *background noise*
17. **Interviewer [MS]: Perfect**
18. Interviewee [XXX]: I do *overlapping speech*
19. **Interviewer [MS]: Do you consent to take part in the interview?**
20. Interviewee [XXX]: Yes I do *speech quite quiet*
21. **Interviewer [MS]: perfect so what I need to do is because you cant actually initial or sign it, I just need to put your initials in the boxes, is that okay?**
22. Interviewee [XXX]: Yes *mumbled speech* *not very clear speech*
23. **Interviewer [MS]: yep *background noise* so can I just confirm what your initials are, is it *confirms initials* ?**
24. Interviewee [XXX]: Um *confirms initials* is fine
25. **Interviewer [MS]: *confirms initials* so like this, can you see?**
26. Interviewee [XXX]: yeah its okay
27. **Interviewer [MS]: that's fine, so you consent to all of these and you're happy for us to audio and video record you. You can turn off your camera any point and any questions you don't wanna answer that's also fine *note speech is slightly broken up***
28. Interviewee [XXX]: Okay *quiet speech*
29. **Interviewer [MS]: um perfect and then *speaking to self quietly*, this is how you**
30. ***video seems paused for a few seconds***
31. **Interviewer [MS]: *unclear speech* is the spelling of your name okay?**
32. Interviewee [XXX]: *very quiet unclear speech*
33. **Interviewer [MS]: hello?**
34. Interviewee [XXX]: *very quiet unclear speech*
35. **Interviewer [MS]: *speaking to self* one second let me just take this out *removes airpod***
36. Interviewee [XXX]: *very quiet unclear speech*
37. **Interviewer [MS]: um is that right? The spelling?**
38. Interviewee [XXX]: yeah *speech quiet*
39. **Interviewer [MS]: yeah perfect um sorry the signal the volume is not that clear and then just gonna put your initials in here as well *clicking of mouse* perfect, that's fine so that's all done um and then once weve done the interview once ive gone**

**through everything um well end up being able to send the consent form back to you so you have a copy. Is that okay?**		
40. Interviewee [XXX]: okay *overlapping* *mumbled speech, hard to hear, nods head*		
**41. Interviewer [MS]: um I also just want to check that are you using airtime for this, dya have an airtime card that you're using for this call? Or you just using wifi?**		
42. Interviewee [XXX]: *mumbled unclear speech*		
**43. Interviewer [MS]: Okay fine so if you were using er airtime card wed say you could ret give it to *name of dr* for a refund but you're just using wifi so that's so if were ever gno do a research project again just so you know you might be able to in the future but just for this *unclear word* using wifi so yeah that would only be the case if you had airtime. Okay so im gno ask you some questions eh you don't have to answer them to the best of your ability if you don't want to answer any that's fine if anything not sure then just ask again if you can't hear properly is that okay?**		
44. Interviewee [XXX]: yeh *quiet speech*		
45. **Interviewer [MS]: perfect. Um so do you prescribe antibiotics to pregnant women?**		
46. Interviewee [XXX]: Yes I do *sound very quiet, hard to hear*		
47. **Interviewer [MS]: Perfect, does the volume I don't know, I can't hear you that clearly.**		
48. Interviewee [XXX]: Ill try and speak *unclear work*		
49. **Interviewer [MS]: Oh yeah, that's abit better it was okay then it goes quiet then loud. So you do prescribe antibiotics to pregnant women? How many times a week do you prescribe them?**		
50. Interviewee [XXX]: ah it depends on my volume of work *sound very quiet*	**50. Prescribe antibiotics (volume of work)**	
• interviewer tries to increase volume*		
51. Interviewee [XXX]: *sound very quiet, hard to hear* maybe that will help *sounds louder*		**1. Prescribing antibiotics**
**52. Interviewer [MS]: mhmm mhmm so**		
53. Interviewee [XXX]: *overlapping speech* it depends		
**54. Interviewer [MS]: okay *unclear speech* How long have you been prescribing them to women for?**		
55. Interviewee [XXX]:o err say over 10 years	**55. Prescribing (years, duration)**	
**56. Interviewer [MS]: Okay, amazing and how many times a week did you say, it depends?**		
57. Interviewee [XXX]: It depends on my volume of work for the week	**57. Prescribing (volume)**	

58. **Interviewer [MS]: Okay, so what are the 3 most common medical problems that you prescribe antibiotics for?**		
59. Interviewee [XXX]: For pregnant women ur suspected urinary infection, um eh suspected upper respiratory tract infections and uh urm well they are the 2 common ones we tend to see um then sometimes yeah food poisoning.	**59. Prescribing (medical conditions)**	
60. *pause*		
61. Interviewee [XXX]: Sorry I didn’t hear that		
62. Interviewee [XXX]: Somethings off with the audio I can’t quite hear you		
63. Interviewee [XXX]: Still not hearing you		
64. *background noise*		
65. Interviewee [XXX]: I really can’t hear you. Are you hearing me? I can’t hear you at all now. You sure your microphone didn’t go off? Im sorry I really can’t hear anything youre saying. Okay		
**66. Interviewer [MS]: Can you hear me now?**		
67. Interviewee [XXX]: Yeah yeah I can		
**68. Interviewer [MS]: I don’t know whats going on, sorry so you said I said are there any guidelines that you use when you prescribe antibiotics?**		
69. Interviewee [XXX]: um well yes sometimes it depends on the antibiotic sensitivity of either the urine culture or *unclear word* analysis, uh sometimes its more empirical	**69. Prescribing (guidelines)**	
**70. Interviewer [MS]: mhmm, okay. Where dya find that pregnant women generally get their antibiotics from?**		
71. Interviewee [XXX]: where do I		
**72. Interviewer [MS]: Where do women, pregnant women generally get their antibiotics from?**	**73. Obtaining antibiotics (sources, vendors)**	**2. Obtaining antibiotics, (from different sources)**
73. Interviewee [XXX]: mostly over the counter drug em pharmacies um small business um drug vendors *unclear word*, I would say um its not as restricted so they tend to get It abit more freely than you think		
**74. Interviewer [MS]: mhm**	**75. Obtaining antibiotics (sources, hospitals)**	
75. Interviewee [XXX]: then of course from the hospitals but on their own quite a number of times they do get it *unclear speech*		
76. **Interviewer [MS]: Okay so are you aware of any pregnant women that might take antibiotics that havent been prescribed for them?**	**77. Self-medication/SM (awareness of)**	**3. Self-medication (awareness & prevalence)**
77. Interviewee [XXX]: yeah sure does happen quite often	**81. SM (common)**	
**78. Interviewer [MS]: mhmm, dya have any**		
79. Interviewee [XXX]: *overlapping speech* Do I what?		

80. **Interviewer [MS]: dya see it a lot?**<br>81. Interviewee [XXX]: yeah quite a number of them do do that you know either the *unclear speech* or they know what they've been given before they just try to get that maybe somebody has said I think this would be good for whatever they complain of and then you know they just get some of those medication<br>**82. Interviewer [MS]: Okay, Do you like are you aware of any pregnant women that might take like herbal preparations or alternative medications that work like antibiotics?**<br>83. Interviewee [XXX]: yes I do, urm some have taken that even *unclear speech* umm *unclear speech* not as much as maybe used to but here we tend to have some um we call them traditional prepem preparations some home remedies or cultural you know things so sometimes depends on tribe too you know we have um we have multi ethnic here so a lot of different cultures have their own preparations and so tend to see some people having taking you know something of that nature<br>**84. Interviewer [MS]: mhmm**<br>85. Interviewee [XXX]: *overlapping speech*<br>**86. Interviewer [MS]: dya have any examples**<br>87. *overlapping speech* *unclear speech*<br>88. Interviewee [XXX]: Do I have an example?<br>**89. Interviewer [MS]: yes**<br>90. Interviewee [XXX]: Of the particular thing they took? Orrr, *sighs* it may be difficult to say what exactly because personally I I don't quite know so much details about these sorts of *unclear speech* *unclear quiet speech* some plants or some trees *muffled speech* some leaves *speech muffled* things like that *speech muffled* *unclear speech*<br>**91. Interviewer [MS]: Okay, the signal its gone quiet again, it was okay and now its gone quiet again, the volume**<br>92. Interviewee [XXX]: Okay *quiet speech*<br>**93. Interviewer [MS]: I can hear you abit but its quite quiet**<br>94. Interviewee [XXX]: alright *quiet speech*<br>**95. Interviewer [MS]: what were you doing before, you el you had sometimes I can hear really clear sometimes not, I don't know if it's the headphones**<br>96. Interviewee [XXX]: *muffled speech* I don't know if it's the headphones ill try *takes headphones off* audio, I don't know if that's better	**83. Herbal SM (yes, home remedies, cultural factors)**<br><br>**90. Herbal SM (examples)**	**4. Herbal self-medication, cultural factors, names, sources)**

**97. Interviewer [MS]: that's better, that's much better yeah**<br>98. Interviewee [XXX]: oh okay<br>**99. Interviewer [MS]: so sorry you were saying that did you say there were any names or you said you're not sure**		
100. Interviewee [XXX]: im im not quite sure of the names you know some of them are not most of them are not english names anyway so that is in a local language or something so it may be difficult to translate whatever it is in english but the usually either roots from a plant or a tree or a you know something	100. Herbal SM (names of herbs, not sure)	5. **Detection of self-medication (direct questioning [TBAs?], rapid testing, in antenatal setting, ease of use)**
**101. Interviewer [MS]: mhm**<br>102. Interviewee [XXX]: usually from supposedly natural sources	102. Herbal SM (natural resources)	
**103. Interviewer [MS]: Okay. Amazing *muffled* , urm *clears throat* do you know of any methods that might identify when a woman is self-medicating with antibiotics?**<br>104. Interviewee [XXX]: any methods urm ahhh it depends, in terms of methods most of the time its all about asking	104. Detecting SM (ways to, direct questioning)	
**105. Interviewer [MS]: mhmm**<br>106. Interviewee [XXX]: because because of how<br>**107. Interviewer [MS]: *increases volume***<br>108. Interviewee [XXX]: liberal we are with medications here you know a lot of people don't tend to be that secretive about taking some antibiotics or something most of the time if you ask have you taken anything for this they say yes I took either this antibiotic or something like that *background noise horn* so it terms of methods I cant quite say unless maybe notice any particular or obvious reaction that may be related to some drug	108. Detecting SM (direct questioning, patients not secretive)	
**109. Interviewer [MS]: mhmm**<br>110. Interviewee [XXX]: but most times its about asking	110. Detecting SM (direct questioning)	
**111. Interviewer [MS]: mhmm. Do you think it could be useful to have a like a simple rapid test or lab test or tool or questionnaire that could help identify pregnant women who might be misusing antibiotics without us knowing?**<br>112. Interviewee [XXX]: well yeah if there was a tool like that I guess it would be useful for those who are not forthcoming with the information, it gives you a way of figuring out what it is without	112. detecting SM (rapid test, useful)	

you know even if they don't say what it is, yes I guess something like that would be useful<br>**113. Interviewer [MS]: Would we interested in using a tool like that?**<br>114. Interviewee [XXX]: yeah I would be interested in using such a tool urm where the uh yeah I would be I would be<br>**115. Interviewer [MS]: Do you think it would be useful to have such a tool just say just hypothetically used in like antenatal care settings, or routine appointments, or like busy A&E departments? Like where do you think it would be really useful to use such a tool?**<br>116. Interviewee [XXX]: sorry could you repeat that?<br>**117. Interviewer [MS]: where do you think it would be useful to use such a tool, like in antenatal appointments or routine appointments or antenatal settings or A&E like where do you think it would be a good place to use such a tool?**<br>118. Interviewee [XXX]: okay well urm pff I guess I would say antenatal appointments because quite a number of times you have that overlapping with other appointments so often you know women around here tend to come to the hospital more often at their antenatal appointments you know its unless they are having some other issues then they end up coming at other times so I would say antenatal appointments<br>**119. Interviewer [MS]: okay and do you feel it needs to be a tool that's like mobile, easy to carry around , you don't need to use electricity or internet, you know something that's just like to be used anywhere, anytime**<br>120. Interviewee [XXX]: yes of course if for for a proper urm universally accepted tool like that it should be something that should be easily applicable at any point in time so I would say yes something that doesn't require electricity doesn't require any complicated process to administer it that would be ideal<br>121. **Interviewer [MS]: Do you have any ideas of what that could be, like what an do you know what I mean any ideas of how it could work?**<br>122. Interviewee [XXX]: well ive not really thought about it before now, so it may be difficult for me to come up with something for the moment but eheh I cant quite say	**114. Detecting SM (rapid test, interesting)**<br><br>**118. Detecting SM (rapid test, setting [antenatal clinic])**<br><br>**120. Detecting SM (ease of use)**<br><br>**122. Detecting SM (no ideas)**	**6. Detecting self-medication (guidelines, need for, 124, 134, 136)**

**123. Interviewer [MS]: mmm its okay its just to wonder, to think about it. Urm so have you come across any guidelines that might help detect side effects of antibiotic self-medication in pregnant women?**<br>124. Interviewee [XXX]: guidelines. Urm not quite I wont say ive um had ur an indepth er read or purused any guidelines per say<br>**125. Interviewer [MS]: or any methods, how would you detect antibiotic self medication in a pregnant woman? Side effects, if she was having side effect?**<br>126. Interviewee [XXX]: mmm umm well, say we me want to detect usually boil down to asking the right questions and then doing a physical examination to look out for sighs certain features that might suggest that<br>**127. Interviewer [MS]: mhmm yeah so similarly we know antibiotics can cause side effects so like stomach upset, or rash, do you think the presence of side effects in a patient is like clear evidence that the patient is taking antibiotics? So if someone has a rash or an upset stomach do you think its clear that that is from antibiotics?**<br>128. Interviewee [XXX]: no no I wouldn't think so actually eh depending on what it is antibiotics may not you know the first thing that comes to mind because of the variety of eh food the people eat and all the things outside those antibiotics so yeah um it wouldn't be the first thing that comes to mind unless its obvious<br>**129. Interviewer [MS]: mhm**<br>130. Interviewee [XXX]: and also if possibly the person has a history of drug allergy or reactions then yeah maybe that may suggest it<br>**131. Interviewer [MS]: mhm. Do you know any pregnant women that have had side effects from antibiotic self-medication?**<br>132. Interviewee [XXX]: mmm not of recent not of recent um but I've had eh I've had 1 or 2 cases of those that they realise earlier that they had either uh allergy to some form of penicillin or something and then end up reacting but not not recently<br>133. **Interviewer [MS]: Okay *background noise ?overlap speech* do you know of any methods or guideline or protocols to manage antibiotic self-medication in women?**	**124. Detecting SM (no guidelines)**<br>**126. Detecting SM (direct questioning, physical exam)**<br>**128. Detecting SM (side effects not indicative)**<br>**130. Detecting SM (side effects, patient history)**<br>**132. Side effects (seen in patients?)**	**7. Detection of self-medication (side effects as indicators)**<br>**Detection of self-**

134. Interviewee [XXX]: not particular for antibiotic self-medication<br>**135. Interviewer [MS]: okay**<br>136. Interviewee [XXX]: not particular for it yes eh maybe particular to one or two non-medications but not in the category of self-medication<br>**137. Interviewer [MS]: fine so its more like from experience really**<br>138. Interviewee [XXX]: yes yeh *overlapping speech*<br>**139. Interviewer [MS]: so this is quite a specific question, last question, so in regards to an area where so some pregnant women if they self-medicate with antibiotics they might get side effects such as memory loss, or forgetfullness, do you know of any management plans if that happened like what would you do in a situation like that?**<br>140. Interviewee [XXX]: particular to memory loss<br>**141. Interviewer [MS]: mhmm memory loss or forgetfulness as like a side effect from self medicating with antibiotics**<br>142. Interviewee [XXX]: mmmm um management plan urm probably not urr an *unclear word said outline* protocol per say in terms of memory loss ur I wouldn't say I am aware of properly stated guideline for that<br>**143. Interviewer [MS]: mhmm okay, and just say if that happened in a clinical area like what do you think would be the management plan or have you never come across it really?**<br>144. Interviewee [XXX]: well urm I try to um figure out if there is any other reason why she should be having memory loss uh before we narrow it down to anti antibiotics um ur of course try to figure out what exactly she took also other option then it also depends on the additional um issue she may be having asides the memory loss as if shes just having what seems like urm simple if I may use the word simple case of amnesia and she doesn't seem to have any other threats to life or to herself<br>**145. Interviewer [MS]: mhmm *quietly***<br>146. Interviewee [XXX]: it would be more of a conservative approach to see what the cause of the um condition will be like and *distorted sound* probably keep her in for a while and observe her closely monitor her vital signs um you know keep a	**134. Guidelines on SM (none)**<br><br>**136. Guidelines on SM (none)**<br><br>**142. Neurological side effects from SM (not aware of)**<br><br>**144. Neurological side effects of SM (other reasons, explored)**<br><br>**146. Neurological sice effects of SM (need for further observation)**	

check on the baby to be sure there isnt any other *unclear word*, were talking particularly for memory loss im just trying to figure out you know the thought would be okay asides memory loss what else could be happening with her and with the pregnancy you understand<br>**147. Interviewer [MS]: yeah**<br>148. Interviewee [XXX]: mm *overlapping speech* quite a number of times some of situations like that I I feel that um an aggressive approach may not necessarily be what is needed<br>**149. Interviewer [MS]: mhmm**<br>150. Interviewee [XXX]: is more being able to gradually figure out what is happening including reviewing the doses and how long and how much she has been taking the actual medication, there is a difference in the fact that maybe she has been taking it antenatally for weeks and then she just took it for a day or two and is having these symptoms so its these are some of the things that guide em what maybe done eventually<br>**151. Interviewer [MS]: yeah**<br>152. Interviewee [XXX]: *overlap* you know yeah so in this scenario basically would eh um guide what happens ill put it that way kay<br>**153. Interviewer [MS]: yeah yeah that's great thank you so much for that insight perfect so that is all my questions um thank you very much do you have any questions?**<br>154. Interviewee [XXX]: um yeah probably ur I should have asked at the beginning but just im just curious urm how how do I put it urm how did I don't know my other colleagues that may be part of this, im just wondering how I ended up in your um list of people to call on<br>**155. Interviewer [MS]: yeah**<br>156. Interviewee [XXX]: yeah<br>**157. Interviewer [MS]: so um were basically doing a joint project with urm its like a collaboration between your hospitals and Liverpool John Moore University, so *name of dr* um**<br>158. Interviewee [XXX]: *overlapping speech*<br>**159. Interviewer [MS]: yeah he basically provided everyones details for us to get in contact with you to take part in the research because it's like a collaboration between the two so that's how**	**150. Neurological side effects of SM (need for careful assessment)**<br><br>Coding ends	

160. Interviewee [XXX]: yeah *overlap* um I was actually going to ask him because I thought it was from him but I kind of forgot you sent me that message until you sent it today again and you know I hadnt got in touch with him but I just forgot that how so I said okay ill ask

161. Interviewer [MS]: yeah *overlap*

162. Interviewee [XXX]: Just to be sure

163. Interviewer [MS]: yeah yeah yeah I mean on the information sheet um *talk to self* I think it does say oh no it just says why we ask you to take part but um

164. Interviewee [XXX]: mmm *overlap*

165. Interviewer [MS]: like the study looking at antenatal care in a Nigerian University Teaching hospital so

166. Interviewee [XXX]: yeah yeah *overlap* I saw I saw all that

167. *overlapping speech*

168. Interviewee [XXX]: um you know I had to *unclear word* look through it to know okay why is this coming from and then I saw certain information I was like okay fine um this is what it is um saw *name of PI* mentioned there but I don't quite know who he is ur but mm let me put it this way his name is actually Ebo same tribe as me

169. Interviewer [MS]: yeah *overlap*

170. Interviewee [XXX]: so it made it more familiar and I was like okay fine

171. Interviewer [MS]: mhmm

172. Interviewee [XXX]: right it sounds interesting enough to want to be a part of yep

173. Interviewer [MS]: yeah no thank you I mean if you've got any other questions so I emailed you so I obviously text you the everything but also I emailed you the consent form and information sheet so you've got it on your emails so you've got my email and you've got my number so ive you've got any questions then you can kind of message me on either um yeah and if you've got any other questions then youre more than welcome to let us know but thank you so much for taking part and for taking time out of your day I know it's the end of the day now but

174. Interviewee [XXX]: *overlap*

175. Interviewer [MS]: Thank you so much

176. Interviewee [XXX]: its fine your welcome

**177. Interviewer [MS]: thank you very much and um yeh have a good rest of the day. Ill send you a copy of the consent form signed like probably in a few weeks time um**<br>178. Interviewee [XXX]: *overlap speech*<br>**179. Interviewer [MS]: if that's okay but it will just be on your emails**<br>180. Interviewee [XXX]: its okay<br>**181. Interviewer [MS]: okay perfect thank you so much have a great evening**<br>182. Interviewee [XXX]: *unclear word* you too<br>**183. Interviewer [MS]: thank you bye**<br>184. Interviewee [XXX]: *unclear word*		
