# Supplementary material for: Advancing the safe motherhood initiative: A qualitative and sentiment analysis of local physician’s perspectives on antibiotic self-medication during pregnancy in a low- and middle-income country
Source: PLOS Glob Public Health. 2025 Sep 12;5(9):e0004794. doi: 10.1371/journal.pgph.0004794 (PMC12431270; doi:10.1371/journal.pgph.0004794)
Supplement: S1 File — Transcript 4 (CODES & THEMES by KU).pdf. Transcript 6 (CODES & THEMES by KU).pdf. Transcript 7 (CODES & THEMES, by KU).pdf. Transcript 8 (CODES & THEMES by KU).pdf. Transcript 9 (CODES & THEMES by KU).pdf. Transcript 10 (CODES & THEMES by KU).pdf. Transcript 11 (CODES & THEMES, by KU).pdf. Transcript 12 (CODES & THEMES by KU).pdf. Transcript 13 (CODES & THEMES by KU).pdf. Transcript 14 (CODED & THEMES by KU).pdf. Transcript 15_b (CODED & THEMES by KU). pdf. Transcript 16 (CODES & THEMES by KU).pdf. Transcript 17 (CODES & THEMES by KU).pdf. Transcript 18 (CODES & THEMES by KU).pdf. Transcript 19 (CODES & THEMES by HK).pdf. Transcript 20 (CODES & THEMES by HK).pdf. Transcript 21_b (CODES & THEMES by HK).pdfTranscript 22 (CODES & THEMES by HK).pdf. Transcript 25 (CODES & THEMES by HK).pdf. Transcript 27 (CODES & THEMES by HK).pdf. Transcript Sn1 (CODES & THEMES by RS).pdf Transcript Sn6 (pt3) (CODES & THEMES by RS).pdf. Transcript Sn15_a (CODES & THEMES by RS).pdf. Transcript SN17 (pt3) (CODES & THEMES by RS).pd. Transcript Sn21_a (CODES & THEMES by RS).pdf. (ZIP) [file pgph.0004794.s001.zip › Transcript 12 (CODES & THEMES by KU).pdf]

Text	Initial Codes	Themes
**Transcription interview 12**<br>**Interviewee: XXX**<br>**SN-11**<br>**Interviewer: (MS), Research Assistant**<br>**Number of speakers : 2**<br>**Time: 5:16pm London time**<br>**Length of interview recording: 16 Minutes 18 seconds**<br>**Date: 27th April 2023**<br><br>**1. Interviewer [MS]: Okay perfect so sorry about that so obviously we just tried to do it on whatsapp vid whatsapp audio call but for some reason the phone isnt recording it properly urm so were going to have to go through the consent again just so ive got a video like a recording of it is that okay?**<br>2. Interviewee [XXX]: That's okay<br>**3. Interviewer [MS]: perfect so as we discussed just before urm you I sent you the information sheet and the consent form before im just gonna put it on the screen one second just gonna put it on the screen**<br>4. Interviewee [XXX]: I went through them I went through every line of It<br>**5. Interviewer [MS]: perfect so as we just said before, can you hear me still?**<br>6. Interviewee [XXX]: yes yes I can hear you<br>**7. Interviewer [MS]: perfect so we went through the you've already gone through the participant information sheet and then**<br>8. Interviewee [XXX]: *overlapping speech*<br>9. **Interviewer [MS]: before so on the phone urm we went you said you read all the consent form when I asked you and you were happy you consent to take part in the interview**<br>10. Interviewee [XXX]:yes I do<br>**11. Interviewer [MS]: and you read all the points on the consent form already before our conversation and you consent to all of them yeah?**<br>12. Interviewee [XXX]: yes yes I do<br>13. **Interviewer [MS]: perfect and you're happy for the recording to be for our conversation to be recorded**<br>14. Interviewee [XXX]: yes I do		

**15. Interviewer [MS]: perfect and um as we discussed before on the phone sorry just for the recording so we have it recorded you're happy for me to put your initials in and weve put your name and the date and im gonna send it back to you after the interview probably next week**		
16. Interviewee [XXX]: that's okay		
**17. Interviewer [MS]: perfect fine so you consent to taking part in the interview yeah**		
18. Interviewee [XXX]: yes I do		
**19. Interviewer [MS]: great okay so we are gonna start now so im *name of interviewer* im the research assistant on this project urm if you have any questions during the interview then just let me know urm if you wanna stop at any point let me know its fine to keep your camera off I know its probably better with the signal urm do you are you using airtime for this call?**		
20. Interviewee [XXX]: *mumbled speech* prescribed data		
**21. Interviewer [MS]: okay fine so that fine so if you use airtime for like for interview you could of asked *name of dr* for a refund but are you okay to use your data**		
22. Interviewee [XXX]: yes im okay with that		
**23. Interviewer [MS]: okay perfect so just gonna ask you some questions just answer the best you can urm if you don't want to answer any that's fine um if you wanna stop just let me know okay?**		
24. Interviewee [XXX]:okay that's part of the consent form		
**25. Interviewer [MS]: pardon?**		
26. Interviewee [XXX]: I said what you just said to me is part of the consent form so *mumbled speech*		
27. **Interviewer [MS]: yeah yeah im just reiterating okay so im gonna start the interview now erm so do you prescribe antibiotics to pregnant women?**		
28. Interviewee [XXX]: yes I do when I think its necessary	**28. Prescribe antibiotics (yes)**	
**29. Interviewer [MS]: okay, How long have you been prescribing antibiotics to pregnant women?**		**[1] PRESCRIBING**
30. Interviewee [XXX]:urm atleast 6 years	**30. Prescribe antibiotics (years)**	
**31. Interviewer [MS]: okay how often how many times a week do you think you prescribe antibiotics to women?**		
32. Interviewee [XXX]: not very often	**32. Prescribe (frequency)**	
33. **Interviewer [MS]: not very often**		
34. Interviewee [XXX]:I would say its *unclear speech* urm maybe once in *distorted speech* 2 weeks 3 weeks *mumbled speech*	**34. Prescribe (frequency)**	
35. **Interviewer [MS]: Okay okay, What are the 3 most common medical problems that you prescribe antibiotics for?**		
36. Interviewee [XXX]:urinary tract infection	**36. Prescribe (med conditions)**	

**37. Interviewer [MS]: mhmm, anything else?**		
38. Interviewee [XXX]: yes occasionally when they have urm *unclear word* vaginal discharge *unclear speech* vaginal discharge and so prescribe antibiotics for *unclear word*	38. Prescribe (medical conditions)	
**39. Interviewer [MS]: okay do you use**		
40. Interviewee [XXX]: *overlapping speech* *unclear speech*		
41. **Interviewer [MS]: pardon?**		
42. Interviewee [XXX]:mostly in the form of pessaries for the vaginal discharge, for the uti mostly *unclear word* antiseptics	42. Prescribe (med conditions)	
**43. Interviewer [MS]: mhmm fine okay. Do you use any guidelines when you prescribe antibiotics?**		
44. Interviewee [XXX]: *unclear speech* urm where I practice we don't have a clear *unclear speech* as for prescription	44. Prescribing (guidelines, none)	
45. **Interviewer [MS]: mhmm**		
46. Interviewee [XXX]: so I wouldn't say I use any *unclear word* guideline	46. Prescribe (guidelines, don't use)	
**47. Interviewer [MS]: okay so where do find that pregnant women normally get their antibiotics from?**		
48. Interviewee [XXX]: from *unclear speech* prescriptions from their O B G Y N	48. Obtain antibiotics (fr. OB-GYN)	[2] OBTAINING
49. **Interviewer [MS]: okay so do they go to their pharmacy or like in the hospital or outside of the hospital?**		
50. Interviewee [XXX]: mostly within the hospital	50. Obtain (fr. hospital)	
51. **Interviewer [MS]: okay**		
52. Interviewee [XXX]: *unclear speech* particular prescription is not available but mostly within the hospital	52. Obtain (fr. Hospital)	
53. **Interviewer [MS]: mhmm do you know of any pregnant women who might have antibiotics that havent been prescribed for them?**		
54. Interviewee [XXX]: occasionally that happens so we try to consult them give them more information and let them know that because in pregnancy there other concentrations that will um look at before making a prescription so let them know that it is not an ideal practice occasionally lets say 1 in 10 or 2 in 10 they do that	54. SM (evidence of, occasionally)	[3] SELF-MEDICATION (freq…)
55. **Interviewer [MS]: okay okay so you don't see it a lot? Its like 1 in 10 women 2 in 10 women**		
56. **Interviewer [MS]: hello?**		
57. Interviewee [XXX]: hi sorry I had a little disruption but im back		

58. **Interviewer [MS]: its okay don't worry *overlap* so you were saying you don't see it that often its 1 1 to 2 in 10 women that you see it?**<br>59. Interviewee [XXX]: yes	59. SM (not often)	
**60. Interviewer [MS]: Okay. Do you know of any pregnant women who take like herbal preparations or alternative medications that like work like antibiotics?**<br>61. Interviewee [XXX]: no<br>62. **Interviewer [MS]: and you've never seen that?**<br>63. Interviewee [XXX]: no ive not	61. Herbal SM (no)<br>63. Herbal SM (no)	[4] HERBAL SELF-MEDICATION
**64. Interviewer [MS]: Okay. So do you know of any ways so just say if you have someone that comes in and there taking antibiotics that havent been prescribed for them as a pregnant women dya know of any ways that you can detect or identify that?**<br>65. Interviewee [XXX]: yes urm occasionally during the history taking sessions *unclear word* history also look at the med drug history *mumbled unclear speech* any medication currently and its through those lines um so because in our environment there is some people *unclear speech* to get the medication down whats prescribed and self prescribed<br>**66. Interviewer [MS]: mhmm**<br>**67.** Interviewee [XXX]: that that's what we want to know and I think that's what we normally do	65. Detecting SM (using patient history)<br>67. Detecting SM (history)	[5] DETECTING SELF-MEDICATION (Patient history…)
**68. Interviewer [MS]: okay. Do you think it would be useful to have like a simple rapid test or lab test or a tool or a questionnaire that would help identify pregnant women who are misusing antibiotics without us knowing?**<br>69. Interviewee [XXX]:yeah that will help if its an easy to *unclear speech* less than a page questionnaire I think that will be good	69. Detecting SM (need for rapid test, easy to use)	
70. **Interviewer [MS]:mhmm If such a test or a tool or a questionnaire was available, would you be interested in using it?**<br>71. Interviewee [XXX]: sure that will help the practice<br>72. **Interviewer [MS]: mhmm.**<br>**73.** Interviewee [XXX]: *overlap speech*	71. Detecting SM (interested in rapid test)	
**74. Interviewer [MS]: where do you think it would be best to use it?**<br>75. Interviewee [XXX]: What did you say can you repeat the question<br>76. **Interviewer [MS]: where dya think it would be best to use it?**<br>77. Interviewee [XXX]: okay because it gives you an additional opportunity to get information from the	77. Detecting SM (usefulness of rapid test)	

patient and it asks more clarity and is less ambiguous and assume that it is going to work its going to be less time consuming urr its something that can be done in the clinics		
**78. Interviewer [MS]: Mhmm so do you think such a tool could be used also within like A&E or just really you know within antenatal care settings or antenatal clinics or do you think it could be used like in other settings?**		
79. Interviewee [XXX]: I didn’t *unclear speech* sorry		
80. **Interviewer [MS]: pardon?**		
81. Interviewee [XXX]: I didn’t get the last thing you said	**83. Detecting SM (settings for rapid test)**	
82. **Interviewer [MS]: um do you think it could be used in other environments like busier environments like A&E or labour ward?**		
83. Interviewee [XXX]: okay I think it can be used everywhere its is about sensitisation letting the practioners understand the usefulness of the tool and then making it simple less time consuming less ambiguous I think the update would be good and I think it can be used everywhere	**85. Detecting SM (settings for rapid test, wards, medical emergencies)**	
84. **Interviewer [MS]: mhmm**		
85. Interviewee [XXX]:because even at the labour wards you have some of these patients who come in as emergencies so if you don’t have *unclear speech* missed that opportunity so I think *unclear speech* point of care	**87. Detecting SM (paper-based questionnaire)**	
**86. Interviewer [MS]: mhmm dya think it would be useful for such a test to be like mobile and not have to use electricity or internet?**		
87. Interviewee [XXX]:I think *unclear speech* that is to say like in *unclear speech* in Nigeria *unclear speech* I think what most suit us is a simple paper based questionnaire which will likely be *unclear word* document right uh must be an opportunity for all of the patients so im thinking *unclear speech* administered by a healthcare worker as soon as the patient comes and *unclear speech* labour ward	**89. Detecting SM (work place)**	
88. **Interviewer [MS]: mhmm so would it be something on a computer or just something that would be like easy to use?**		
89. Interviewee [XXX]: urm if you were to adapt it in the place where we work because with elec elec electronic medical records its not in all of the stations		
90. **Interviewer [MS]: mhmm**		
91. Interviewee [XXX]: *unclear speech* paper based *unclear speech*	**93. Guidelines on SM (no)**	
92. **Interviewer [MS]: Mhmm. Okay. Have you come across any methods or guidelines which help detect side**		**[6] GUIDELINES**

**effects of antibiotic self-medication in pregnant women?**<br>93. Interviewee [XXX]: no havent<br>94. **Interviewer [MS]: okay so if someone came in and they were having a reaction or side effects you wouldn't specifically know that was because of self medicating with antibiotics?**<br>95. Interviewee [XXX]: if someone were to come in with er with what looks like adverse drug reaction<br>96. **Interviewer [MS]: mhmm**<br>97. Interviewee [XXX]: errr ill take a quick history<br>98. **Interviewer [MS]: mhmm**<br>99. Interviewee [XXX]: *overlap* because we do that a lot of people here react to um sulphur containing drugs<br>100. **Interviewer [MS]: mhmm**<br>101. Interviewee [XXX]: and because of that *unclear speech* we take history along with *unclear speech* find out we know the common drugs that *unclear speech* some of the SPs that we use in pregnancy urm for malaria prophylaxis because the tropics<br>102. **Interviewer [MS]: mhmm**<br>103. Interviewee [XXX]: they contain sulphur some of the antibiotics we use too *name of antibiotic unclear* has some sulphur in it so yes through history taking *unclear speech*<br>104. **Interviewer [MS]: Okay**<br>105. Interviewee [XXX]: find out<br>**106. Interviewer [MS]: okay so similarly urm we know antibiotics can cause side effects like rashes, stomach upset, vomiting, things like that do you think the presence of side effects is clear when a person is taking antibiotics? Like is it clear that its from the antibiotics?**<br>107. Interviewee [XXX]: wow it will not always be clear to be honest *unclear speech* urm because some of those reactions depend on the persons genetic make up too and so most persons will not react *unclear word * to antibiotics so I think it will be and that means that even if cancelling before the prescription *unclear speech* sitting down and say ah this drug will likely cause this and this and this. Usually the *unclear words* I know in practice that counselling's usually not done so I would think that urr it may be difficult for people to remember that its coming from the antibiotic so maybe from the *unclear speech* or maybe theres something else there<br>108. **Interviewer [MS]: mhmm**	**95/96/97. Sides effects from SM (patient history)**<br><br>**99/101. Side effects, patient history**<br><br>**103. Side effects, patient history**<br><br>**107. Side effects from SM (not always clear, depends)**	**[7] SIDE EFFECTS**

109. Interviewee [XXX]: and then of course the practitioner when he gets that complaint from the patient but *unclear speech* it may be difficult for people to easily access *unclear speech* the antibiotics *unclear speech*<br>**110. Interviewer [MS]: mhmm yeah youre right. Do you know any pregnant women that have been suspected to have side effects of antibiotic self-medication? Have you ever seen that?**<br>111. Interviewee [XXX]: self medication no, but ive seen someone *unclear word* patient that had reaction to prescribed medication<br>**112. Interviewer [MS]:mhmm, mhmm okay but you've never seen it for self medication?**<br>113. Interviewee [XXX]: no ive not I cannot remember any such persons	**111/113. Side effects from SM (not seen such patients).., but seen for prescribed meds**	
114. **Interviewer [MS]: okay *overlap* that's fine. Urm and do you know of any methods or guidelines or protocols that look at managing antibiotic self medication in pregnant women?**<br>115. Interviewee [XXX]: no I do not	**115. Guidelines on SM (no/none)**	**[6] GUIDELINES**
**116. Interviewer [MS]: okay and then this is very specific to this area but sometimes there might be pregnant women who have self medicated with antibiotics and then they might develop signs of memory loss, or forgetfullness, um do you know of any management options if that happened as a side effect?**<br>117. Interviewee [XXX]: ive not been urm *unclear speech*<br>118. **Interviewer [MS]: mhmm**<br>119. Interviewee [XXX]: urr at the moment no<br>**120. Interviewer [MS]: okay okay that's fine, I know its quite a difficult question but its just really kind of if you've got any experience of that or kind of know what to do so its okay if you know youre not sure as well urm okay**	**119. Neurological effects of (none)**	**[7] SIDE EFFECTS (neuro…)**
121. Interviewee [XXX]: *overlapping speech* should I encounter that kind of thing *unclear speech*<br>**122. Interviewer [MS]: *overlapping speech* yeah no its just *overlapping speech* go on sorry**<br>**123.** *overlapping speech*<br>124. Interviewee [XXX]: *unclear speech* are more likely involved in urm clinical pharmacologists and um in our centre *unclear speech* microbiologists medical microbiologists *unclear speech* if we think its an adverse reaction then certainly we will involve the clinical pharmacologists and<br>**125. Interviewer [MS]: mm**	**124/126. Neurological effects (if adverse reactions, will seek other clinical experts)…**<br>*[NOTE: no ref to SM]*	

126. Interviewee [XXX]: and where it is possible to if its *unclear speech* therapeutic drug margin we may be able to *unclear speech* still call for help and *unclear speech*<br>127. **Interviewer [MS]: mhmm**<br>128. Interviewee [XXX]: know what to do<br>129. **Interviewer [MS]: yeah yeah no definitely, so that is all my questions so thank you very much. Um do you have any questions at all?**<br>130. Interviewee [XXX]: *unclear speech*<br>**131. Interviewer [MS]: no okay so thank you very much for taking part, im sorry about the distruption and the difficulty at the beginning you know whatsapp call and everything so thank you for coming on zoom. Um I will send you back the consent form next week or the week after um complete ill send it back to your email and if you have any questions in the mean time then you've got my email you've got my number just send me a message if you've got any questions but yeah that's all on my behalf and thank you so much for taking part**<br>132. Interviewee [XXX]: your very welcome the pleasure is all mine<br>133. **Interviewer [MS]: okay**<br>134. Interviewee [XXX]: *unclear speech*<br>**135. Interviewer [MS]: thank you so much have a great rest of the day**<br>136. Interviewee [XXX]: you too<br>137. **Interviewer [MS]: thank you bye**		
