# Supplementary material for: Advancing the safe motherhood initiative: A qualitative and sentiment analysis of local physician’s perspectives on antibiotic self-medication during pregnancy in a low- and middle-income country
Source: PLOS Glob Public Health. 2025 Sep 12;5(9):e0004794. doi: 10.1371/journal.pgph.0004794 (PMC12431270; doi:10.1371/journal.pgph.0004794)
Supplement: S1 File — Transcript 4 (CODES & THEMES by KU).pdf. Transcript 6 (CODES & THEMES by KU).pdf. Transcript 7 (CODES & THEMES, by KU).pdf. Transcript 8 (CODES & THEMES by KU).pdf. Transcript 9 (CODES & THEMES by KU).pdf. Transcript 10 (CODES & THEMES by KU).pdf. Transcript 11 (CODES & THEMES, by KU).pdf. Transcript 12 (CODES & THEMES by KU).pdf. Transcript 13 (CODES & THEMES by KU).pdf. Transcript 14 (CODED & THEMES by KU).pdf. Transcript 15_b (CODED & THEMES by KU). pdf. Transcript 16 (CODES & THEMES by KU).pdf. Transcript 17 (CODES & THEMES by KU).pdf. Transcript 18 (CODES & THEMES by KU).pdf. Transcript 19 (CODES & THEMES by HK).pdf. Transcript 20 (CODES & THEMES by HK).pdf. Transcript 21_b (CODES & THEMES by HK).pdfTranscript 22 (CODES & THEMES by HK).pdf. Transcript 25 (CODES & THEMES by HK).pdf. Transcript 27 (CODES & THEMES by HK).pdf. Transcript Sn1 (CODES & THEMES by RS).pdf Transcript Sn6 (pt3) (CODES & THEMES by RS).pdf. Transcript Sn15_a (CODES & THEMES by RS).pdf. Transcript SN17 (pt3) (CODES & THEMES by RS).pd. Transcript Sn21_a (CODES & THEMES by RS).pdf. (ZIP) [file pgph.0004794.s001.zip › Transcript 13 (CODES & THEMES by KU).pdf]

Text	Initial codes	Themes
**Transcription interview 13**<br>**Interviewee: XXX**<br>**SN-16**<br>**Interviewer: (MS), Research Assistant**<br>**Number of speakers :2**<br>**Time: 6pm**<br>**Length of interview recording: 18 minutes 52 seconds**<br>**Date: 27th April 2023**<br><br>**1. *background noise***<br>**2. Interviewer [MS]: Okay so I'm *name of interviewer* nice to meet you. Urm I am the research assistant on this project emm so thank you so much for taking the time to take part in the project, we really appreciate it.**<br>3. Interviewee [XXX]: okay<br>**4. Interviewer [MS]: urm did you read the information sheet and the consent form that I sent to you?**<br>5. Interviewee [XXX]: Yeah yeah I did<br>**6. Interviewer [MS]: yes perfect. so did you manage to look through, I'm just going to share it on my screen, one second urm *shares on screen*. So you've already had a look through this part this information sheet correct?**<br>**7.** Interviewee [XXX]: yea yea<br>**8. Interviewer [MS]: perfect and then also so this is the consent form *shares consent form on screen* fine so you had a read through this yeah?**<br>9. Interviewee [XXX]: yea yea *unclear word*<br>**10. Interviewer [MS]: can you see, um did you agree to all of the points that you read?**<br>11. Interviewee [XXX]: yeah I did<br>**12. Interviewer [MS]: did you consent to everything yeh? Dya consent to take part and you consent to all the points on the information sheet? On the consent form sorry**<br>13. Interviewee [XXX]: ah yeah its okay<br>**14. Interviewer [MS]: okay so what were gonna do is cause we need to fill it out, urm is it okay if I just put your initials in the boxes and then once weve done the interview probably next week or the week after ill send you a copy of the consent form so you've got it for your records**<br>15. Interviewee [XXX]: okay<br>**16. Interviewer [MS]: Is that okay? So whats your intials sorry?**<br>17. Interviewee [XXX]: my name amm		

18. **Interviewer [MS]: yes *overlap***
19. Interviewee [XXX]: *mumbled speech* *background noise* hello?
20. **Interviewer [MS]: hi sorry your intials**
21. Interviewee [XXX]: my initials okay *unclear word* *says initials* *repeats initials*
22. **Interviewer [MS]: like this? like that on the screen?**
23. ***silence***
24. **Interviewer [MS]: Is that correct? On the screen *repeats initials***
25. ***silence***
26. Interviewee [XXX]: *broken up speech*
27. **Interviewer [MS]: sorry the signals really bad. *pause* Can you hear me? *pause* Can you hear me?**
28. Interviewee [XXX]: hello I can hear you
29. **Interviewer [MS]: oh sorry the signal was really bad, urm**
30. Interviewee [XXX]: *overlapping speech**unclear word*
31. **Interviewer [MS]: *confirming initials***
32. Interviewee [XXX]: *says initial* is good yeah
33. **Interviewer [MS]: like that? Like ive done it on the screen?**
34. Interviewee [XXX]: yeah yeah yeah
35. **Interviewer [MS]: right so im just gonna put that in all of the boxes and then you're happy for me to record it? Yeah?**
36. Interviewee [XXX]: okay
37. **Interviewer [MS]: you can keep your urm camera off cause it probably helps the signal I understand um but if you decide you want to turn it on that's also fine its completely up to you urm and then yo we can stop the interview at any point if you don't want to take part anymore or you can withdraw at any point um you don't have to answer all the questions its completely up to you but and im gonna write your name on here**
38. Interviewee [XXX]: *unclear speech*
39. **Interviewer [MS]: *speaking to self* and todays date is the 27th erm and then just because you cant sign your signature**
40. Interviewee [XXX]: *background noise*
41. **Interviewer [MS]: just gonna put *puts initials in box* okay so that's fine *background noises* . ive completed this form so once urm**
42. ***background noises***
43. **Interviewer [MS]: can you hear me sorry?**
44. ***background noises***
45. **Interviewer [MS]: hello?**

46. Interviewee [XXX]: *background noise* hello?<br>**47. Interviewer [MS]: hey sorry theres a lot of background noise**<br>48. Interviewee [XXX]: *background noise* yeah let me get into somewhere more *unclear speech and background noise*<br>**49. Interviewer [MS]: *unclear speech***<br>50. Interviewee [XXX]: *background noise and mumbled speech*<br>**51. Interviewer [MS]: hello?**<br>**52. *background noise then silence***<br><br>**53. Interviewer [MS]: *after a few seconds silence* Hello?**<br>**54. Interviewer [MS]: *no response* hello? And he has gone off for now *speaks to self***<br><br>55. Interviewee [XXX]: *background noise* hello?<br>**56. Interviewer [MS]: hi can you hear me now?**<br>57. Interviewee [XXX]: yeh *background noise*<br>**58. Interviewer [MS]: okay so**<br>**59. *background noise***<br>**60. Interviewer [MS]: one second urm okay so obviously weve gone through the consent form urm ive signed ive wrote your intials on it so youre happy to take part in the study and you've read it all yeah?**<br>61. Interviewee [XXX]: okay<br>**62. Interviewer [MS]: that correct *overlap***<br>63. Interviewee [XXX]: *unclear speech*<br>**64. Interviewer [MS]: okay perfect so ill send you that once weve finished the interview *background noise* okay so ive just got a few questions urm answer them to the best of your ability if you're not able to answer any then that's okay urm its just some questions is that okay?**<br>65. Interviewee [XXX]: okay<br>**66. Interviewer [MS]: Okay so do you prescribe antibiotics to pregnant women?**		
67. Interviewee [XXX]: yeahh depends on indication<br>**68. Interviewer [MS]: pardon?**<br>69. Interviewee [XXX]: on indication on indication depends on<br>**70. Interviewer [MS]: *overlapping speech***<br>71. Interviewee [XXX]: *unclear speech*prescribe antibiotics to pregnant women depends on the indication on indication yeah I do	**67/71. Prescribe antibiotics (yes, depends)**	**[1] PRESCRIBING**
**72. Interviewer [MS]: Okay, How long have you prescribed antibiotics to women for?**		
73. Interviewee [XXX]: how long?<br>**74. Interviewer [MS]: mhmm**<br>75. Interviewee [XXX]: urm how long, since I started my since ive been a doctor	**73/75/77. Prescribe antibiotics (duration)**	

**76. Interviewer [MS]: mhmm *overlapping speech*** <br>77. Interviewee [XXX]: been a doctor have like um *unclear speech* 15 years **78. Interviewer [MS]: Okay How many times a week do you think you prescribe antibiotics to pregnant women?** 79. Interviewee [XXX]: urm ah rarely rarely **80. Interviewer [MS]: Rarely?** 81. Interviewee [XXX]: yeah rarely they rarely need it **82. Interviewer [MS]: Okay, urm what are the 3 most common medical problems that you prescribe antibiotics to pregnant women for?** 83. Interviewee [XXX]: okay eh like eh uti **84. Interviewer [MS]: mhm** 85. Interviewee [XXX]: *mumbled unclear speech* if there if there is *unclear speech* common others are rare *unclear quiet mumbled speech* **86. Interviewer [MS]: mhm** 87. Interviewee [XXX]: *unclear mumbled speech* **88. Interviewer [MS]: mhmm. Do you what are the 3 most common so so** 89. Interviewee [XXX]: *overlap of speech* *unclear speech* *mumbling* what did you say sorry? **90. Interviewer [MS]: pardon?** 91. Interviewee [XXX]: what you say, what did you say? **92. Interviewer [MS]: no no sorry the signal is just not great so I just couldn't hear properly** **93. Interviewer [MS]: Urm Do you use any guidelines when you're prescribing antibiotics?** 94. Interviewee [XXX]: guidelines no *unclear speech* guideline **95. Interviewer [MS]: okay** 96. Interviewee [XXX]: *unclear mumbled speech* **97. Interviewer [MS]: okay and where do find that pregnant women generally get antibiotics from?** 98. Interviewee [XXX]: ah? **99. Interviewer [MS]: where do you find that pregnant women generally get their antibiotics from?** 100. Interviewee [XXX]: where they get it from? **101. Interviewer [MS]: yeah where do they get it from?** 102. Interviewee [XXX]: where? Where do they get it from **103. Interviewer [MS]: *overlap* yeah** 104. Interviewee [XXX]: the pharmacy **105. Interviewer [MS]: in the hospital or outside of the hospital?** 106. Interviewee [XXX]: in the hospital	**79/81. Prescribe (freq)** <br>**94. Prescribe (guidelines, no)** <br>**100 to 106. Obtaining antibiotics (fr. Pharmacy, hospital)**	**[2] OBTAINING antibiotics. (100, 117, 121..)**

107. **Interviewer [MS]: Okay. So do you know of any pregnant women who have taken antibiotics that havent been prescribed for them?**		
108. Interviewee [XXX]: yeah *unclear speech* yeah yeah couple of some go	108/110. SM in pregnant women (yes)	[3] SELF-MEDICATION
**109. Interviewer [MS]: yeah**		
110. Interviewee [XXX]: some ahh might get it self and self self self medicating		
111. *overlapping speech*		
**112. Interviewer [MS]: dya have examples?**		
113. Interviewee [XXX]: do I have any what?		
**114. Interviewer [MS]: like examples or dya see it often?**		
115. Interviewee [XXX]: examples *overlap* no particular example but generally I know *unclear speech* antibiotics over the counter		
**116. Interviewer [MS]: mhm**		
117. Interviewee [XXX]: in the pharmacy in the private pharmacy *unclear speech* you can get it in the hospital pharmacy you can get in private pharmacy too, you can eh obviously eh I know some of them can *unclear word* self prescribe antibiotics for them *unclear speech*	117. SM… obtain antibiotics from private pharmacy	
**118. Interviewer [MS]: mhmm are you aware of any pregnant women who might take like herbal preparations or alternative medications that work like antibiotics?**		
119. Interviewee [XXX]: I know I know *unclear speech*		
**120. Interviewer [MS]: mhmm**		
121. Interviewee [XXX]: *unclear speech*I don't know where they get it from but some have *broken up speech* you know opened up and said yeah there taking some herbal *unclear word* so ehhhh either to make the baby smaller *unclear speech* vaginal delivery. I don't know what the herbs contain I don't know if they have eh any *unclear speech* act like antibiotics I don't know what the herbs I don't know how they are *unclear speech* so ah *unclear speech* some take it because they feel they want to cleanse their system, you know theres a lot of misinformation too	121. SM… obtain antibiotic [herbs] (not clear where from)	
**122. Interviewer [MS]: mhmm**		
123. Interviewee [XXX]: *overlapping speech* *unclear mumbled speech*		
**124. Interviewer [MS]: mhm okay urm do you know of any methods that detect or identify self-medication of antibiotics in pregnant women?**		
125. Interviewee [XXX]: any what?		
**126. Interviewer [MS]: any ways to detect or identify self medication of antibiotics in pregnant women**		

127. Interviewee [XXX]: *mumbled speech* detect or identify nah you just have to ask *unclear word* if they taking *unclear speech*<br>**128. Interviewer [MS]: Mhmm**<br>129. Interviewee [XXX]: that could help<br>**130. Interviewer [MS]: mhm**<br>131. Interviewee [XXX]: urm im not aware of any other ways to any other way to assess or identify<br>**132. Interviewer [MS]: mhmm**<br>133. Interviewee [XXX]: *overlapping speech* just ask and they will tell you<br>**134. Interviewer [MS]: mhm, dya think it would be useful to have like a simple rapid test or lab tool or questionnaire that would help identify pregnant women who might be misusing antibiotics without us knowing about it?**<br>135. Interviewee [XXX]: yeah yeah *unclear speech*<br>**136. Interviewer [MS]: mhm. Would you be**<br>137. Interviewee [XXX]: *overlapping speech*<br>**138. Interviewer [MS]: sorry go**<br>139. Interviewee [XXX]: *unclear speech*<br>**140. Interviewer [MS]: Mhm**<br>141. Interviewee [XXX]: *unclear speech* lab test<br>**142. Interviewer [MS]: mhm**<br>143. Interviewee [XXX]: *unclear speech*<br>**144. Interviewer [MS]: would you be interested in using such a tool?**<br>145. Interviewee [XXX]: urmm yeh yeh *unclear mumbled speech*<br>**146. Interviewer [MS]: Okay and Do you think such a tool could be used in like antenatal settings or during routine appointments, or in A&E?**<br>147. Interviewee [XXX]: mmm<br>**148. Interviewer [MS]: *cough***<br>149. Interviewee [XXX]: *mumbled* antenatal setting yea yea it could be used in antenatal settings<br>**150. Interviewer [MS]: mhmm and Dya think it would be useful for such a test or a tool if it was around urm would be better to be like remote and not have to use internet for it?**<br>151. Interviewee [XXX]: come again<br>**152. Interviewer [MS]: just say if we had a tool dya think it would be better or useful if it was easy to use and not having to use electricity or internet or things like that**<br>153. Interviewee [XXX]: *overlap speech* *unclear speech* definitely<br>**154. Interviewer [MS]: pardon?**	**127. Detecting SM… (asking)**<br><br>**131/133. Detecting SM…(ask/no other way)**<br><br>**135/141. Detecting SM (rapid test/yes, lab test)**<br><br>**149. Detecting SM (setting, antenatal care)**	**[5] DETECTING SELF-MEDICATION (‘METHODS’)**

155. Interviewee [XXX]: definitely definitely its better if it doesn't have	155. Detection tool (not rely on electricity)	
**156. Interviewer [MS]: *overlapping speech***		
157. Interviewee [XXX]: yeah definitely yeah		
**158. Interviewer [MS]: okay and have you come across any guidelines or methods that help detect side effects of antibiotic self-medication in pregnant women?**		[6] GUIDELINES
159. Interviewee [XXX]: no	159. Side effects of SM (no guidelines)	
**160. Interviewer [MS]: No okay**		
161. Interviewee [XXX]: no		
**162. Interviewer [MS]: so generally we know antibiotics can cause side effects like a rash or stomach upset, do you think the presence of such side effects is clear that its from antibiotics?**		
163. Interviewee [XXX]: nah *mumbled speech*		
**164. Interviewer [MS]: pardon?**		
165. Interviewee [XXX]:*unclear speech* side effects		[7] SIDE EFFECTS
**166. Interviewer [MS]: mhm**		
167. Interviewee [XXX]: have specifically *unclear speech* side effects doesn't necessarily mean that due to antibiotics	167/169. Sides effects (ambiguous, not necessarily antibiotics)	
**168. Interviewer [MS]: mhm**		
169. Interviewee [XXX]: other things could be influence		
**170. Interviewer [MS]: mhm. Okay do you know any pregnant women that have developed side effects after self medicating with antibiotics?**		
171. Interviewee [XXX]: No no		
**172. Interviewer [MS]: no**		
173. Interviewee [XXX]: *overlap* no		
174. **Interviewer [MS]: Okay. And are there any methods or guidelines or protocols to manage antibiotic self medication in pregnant women?**		[6] GUIDELINES
175. Interviewee [XXX]: no *background noise*		
**176. Interviewer [MS]: no nothing okay. This is a bit separate but its related urm so sometimes pregnant women who self medicate with antibiotics sometimes like signs of memory loss or forgetfulness. do you know of any management options or how you would treat this if that happened?**	175. SM (guidelines, none)	[7] SIDE EFFECTS
177. Interviewee [XXX]: no	177. Neurological effects of SM (management, no)	
**178. Interviewer [MS]: no?**		
179. Interviewee [XXX]: no		
**180. Interviewer [MS]: okay okay that's great. That's all my questions thank you very much I know its difficult cuse the signal isn't great urm but do you have any questions?**		

181. Interviewee [XXX]: no Im good

182. Interviewer [MS]:*overlapping speech*

183. Interviewee [XXX]: *unclear speech* the only thing I want to know is what is the goal of the study

184. Interviewer [MS]: so were looking at antibiotic err misuse in pregnancy basic like in the antenatal period so just doing some interviews and then you know hopefully well get more of an insight into use of antibiotics or misuse of antibiotics in pregnancy like on the information sheet it gives you a lot more detail about it though urm but that's kind of the goal so once weve done all the interviews urm then we should be able to you know make a report and then well be able to share that with you if you would like to

185. Interviewee [XXX]: okay okay

186. Interviewer [MS]: is that okay?

187. Interviewee [XXX]: that's okay that's okay

188. Interviewer [MS]: perfect also you know the information sheet is really good because it goes into detail and you know also looking at ways that we can like look at diagnosing and finding antibiotic misuse in pregnancy so we can send you you know then final report when its done if you would like it

189. Interviewee [XXX]: that will be that will be nice

190. Interviewer [MS]: okay perfect so you've got my email and you've also got my phone number so if you need anything urm then just let me know

191. Interviewee [XXX]: *overlapping mumbled speech*

192. Interviewer [MS]: If you've got any other questions, also are you using airtime card for this?

193. Interviewee [XXX]: using what?

194. Interviewer [MS]: Are you using an airtime card for this interview?

195. Interviewee [XXX]: yeah using my using my phone

196. Interviewer [MS]: is it airtime?

197. Interviewee [XXX]: yes airtime

198. Interviewer [MS]: okay so if its airtime then if you've got a card you can submit it to *name of dr* for a refund

199. Interviewee [XXX]: oh

200. Interviewer [MS]: for taking part

201. Interviewee [XXX]: ok *mumbled*

202. Interviewer [MS]: okay?

203. Interviewee [XXX]: okay

204. Interviewer [MS]: okay perfect thank you so much and I will send you the complete consent form in the next few weeks as well to your email okay?

205. Interviewee [XXX]: no problem *overlap*

**206. Interviewer [MS]: okay thank you so much for taking part we really appreciate it and yeah have a nice evening**<br>207. Interviewee [XXX]: you too dear<br>**208. Interviewer [MS]: thank you**<br>209. Interviewee [XXX]: thank you dear bye<br>**210. Interviewer [MS]: bye**<br>211. Interviewee [XXX]: bye *background noise*		
