# Supplementary material for: Advancing the safe motherhood initiative: A qualitative and sentiment analysis of local physician’s perspectives on antibiotic self-medication during pregnancy in a low- and middle-income country
Source: PLOS Glob Public Health. 2025 Sep 12;5(9):e0004794. doi: 10.1371/journal.pgph.0004794 (PMC12431270; doi:10.1371/journal.pgph.0004794)
Supplement: S1 File — Transcript 4 (CODES & THEMES by KU).pdf. Transcript 6 (CODES & THEMES by KU).pdf. Transcript 7 (CODES & THEMES, by KU).pdf. Transcript 8 (CODES & THEMES by KU).pdf. Transcript 9 (CODES & THEMES by KU).pdf. Transcript 10 (CODES & THEMES by KU).pdf. Transcript 11 (CODES & THEMES, by KU).pdf. Transcript 12 (CODES & THEMES by KU).pdf. Transcript 13 (CODES & THEMES by KU).pdf. Transcript 14 (CODED & THEMES by KU).pdf. Transcript 15_b (CODED & THEMES by KU). pdf. Transcript 16 (CODES & THEMES by KU).pdf. Transcript 17 (CODES & THEMES by KU).pdf. Transcript 18 (CODES & THEMES by KU).pdf. Transcript 19 (CODES & THEMES by HK).pdf. Transcript 20 (CODES & THEMES by HK).pdf. Transcript 21_b (CODES & THEMES by HK).pdfTranscript 22 (CODES & THEMES by HK).pdf. Transcript 25 (CODES & THEMES by HK).pdf. Transcript 27 (CODES & THEMES by HK).pdf. Transcript Sn1 (CODES & THEMES by RS).pdf Transcript Sn6 (pt3) (CODES & THEMES by RS).pdf. Transcript Sn15_a (CODES & THEMES by RS).pdf. Transcript SN17 (pt3) (CODES & THEMES by RS).pd. Transcript Sn21_a (CODES & THEMES by RS).pdf. (ZIP) [file pgph.0004794.s001.zip › Transcript 14 (CODED & THEMES by KU).pdf]

Text/transcript	**Initial codes**	**Themes**
1) **Transcription interview 14**		
2) **Interviewee: XXX**		
3) **SN- 1**		
4) **Interviewer: MS, Research Assistant**		
5) **Number of speakers: 2**		
6) **Time: 6.02pm London time**		
7) **Length of interview recording: 23 minutes 05 seconds**		
8) **Date: 4th May 2023**		
9) **Note-** Consent obtained at the start of the interview. Participant consented to take part in the study, recorded on zoom recording. Participant advised that they were taking part to be able to advise hospital about the study.		
10) **Interviewer [MS]: Do you prescribe antibiotics to pregnant women?**		
11) Interviewee [XXX]: yes	**11.Prescribe antibiotics (yes)**	
12) **Interviewer [MS]: How long have you been prescribing them for?**		
13) Interviewee [XXX]: *laughs* its more than 30 years	**13. Prescribe antibiotics (duration/years)**	**[1] Prescribing antibiotics**
14) **Interviewer [MS]: Mhmm okay so how many times a week do you think you prescribe them to pregnant women?**		
15) Interviewee [XXX]: When I started, when I was a younger doctor prescribe it on daily basis	**15. Prescribe antibiotics (freq)**	
16) **Interviewer [MS]: mhmm**		
17) Interviewee [XXX]: yes but now as a secior doctor maybe twice in a week	**17. Prescribe antibiotics (freq)**	
18) **Interviewer [MS]: Okay Okay and what are the 3 most common medical problems that you prescribe them for?**		
19) Interviewee [XXX]: umm pelvic inflammatory disease		
20) **Interviewer [MS]: mhm**		
21) Interviewee [XXX]: UTI in pregnancy	**19/21/23. Prescribe antibiotics (condition)**	
22) **Interviewer [MS]: yeah**		
23) Interviewee [XXX]: and ahh those are the common the most common causes		
24) **Interviewer [MS]: Okay and do you use any guidelines when you're prescribing antibiotics?**		
25) Interviewee [XXX]: uh except we have a guideline the hospital		

**26) Interviewer [MS]: mhmm**<br>27) Interviewee [XXX]: occasionally its em reviewed<br>**28) Interviewer [MS]: mhmm**<br>29) Interviewee [XXX]: so anytime they have a review they send it to us and we use it but not frequent<br>**30) Interviewer [MS]: Okay, so where do you find that pregnant women generally obtain their antibiotics from?**	**25/27. Prescribe antibiotics (guidelines)**	
**31)** Interviewee [XXX]: urm from a hospital pharmacy?<br>**32) Interviewer [MS]: Mhm**<br>33) Interviewee [XXX]: Yeah from a hospital pharmacy sometimes that's if the drugs are available and if the patients have the money with them<br>**34) Interviewer [MS]: mhmm**	**31/33. Obtaining antibiotics (hosp pharmacy)**	**[2] Obtaining antibiotics (sources)**
35) Interviewee [XXX]: If they don't have the money with them they might have to go home and ask their husbands to give them the money and in that case we don't know where they get the drugs from<br>**36) Interviewer [MS]: Okay, so are you aware of any pregnant women who take antibiotics that havent been prescribed for them?**<br>**37)** Interviewee [XXX]: Can you come again?<br>**38) Interviewer [MS]: Are you aware of any pregnant women who take antibiotics that haven't been prescribed for them?**	**35. Obtaining antibiotics (unknown source)**	
39) Interviewee [XXX]: urmm yess yes<br>**40) Interviewer [MS]: Dya have more details? Like when would that happen?**	**39. SM (yes, observed it)**	**[3] Self-medication (awareness of, source, motivation [symptoms, social pressure])**
41) Interviewee [XXX]: yes. Some of them erm they go to the chemist<br>**42) Interviewer [MS]: Mhmm**	**41. SM (they go to chemist)**	
43) Interviewee [XXX]: and complain that their having either vaginal vuval itching<br>**44) Interviewer [MS]: mhmm**<br>**45)** Interviewee [XXX]: and erm sometimes especially in pregnancy you are talking about pregnancy isnt it?<br>**46) Interviewer [MS]: mhmm**	**43. SM (complain to chemist)**	
47) Interviewee [XXX]: so it's the vulval itching that is common in pregnancy that they normally go to the hospital erm to the chemist or somewhere and they prescribe some antibiotics for them<br>**48) Interviewer [MS]: *clears throat* okay so is there ever a time when women take antibiotics that haven't been prescribed for them**<br>49) Interviewee [XXX]: How many times?<br>**50) Interviewer [MS]: Does it happen that women take antibiotics that haven't been prescribed for them?**	**47. SM (complain to chemist, symptoms)**	

51) Interviewee [XXX]: sorry I don't understand your question		
**52) Interviewer [MS]: It's okay so sometimes the pregnant women, have you ever seen pregnant women take antibiotics that have not been prescribed for them by a doctor**		
53) Interviewee [XXX]: Yes yes yes that's what im telling you yes	**53. SM (yes, observed)**	
**54) Interviewer [MS]: or even in the pharmacy the ones like you know that they've got from different places not just its not just got a prescription they've just kinda been given them**		
55) Interviewee [XXX]:yes yes	**55. SM (yes)**	
**56) Interviewer [MS]: Does that where does that happen? Like not just the pharmacy does it happen anywhere else?**		
57) Interviewee [XXX]: urm it happens maybe urm with friends might have taken some antibiotics and urm will tell the other ones that okay I had similar problem this what I took	**57. SM (encouraged by friends)**	
**58) Interviewer [MS]: mhmm**		
59) Interviewee [XXX]: and I still have some can you take will you don't mind you can have some of the antibiotics	**59. SM (encouraged by friends)**	
**60) Interviewer [MS]: mhmm okay so are you aware of any pregnant women who might take like**		
61) **herbal preparations or alternative medications that work like antibiotics?**		
**62)** Interviewee [XXX]: yes	**62. Herbal SM (yes)**	
**63) Interviewer [MS]: Dya have any examples?**		**[4] Herbal self-medication (observed, cultural aspects, motivation)**
64) Interviewee [XXX]: mmmm they take some em you want me the names of the local	**64. Herbal SM (yes, local herbs)**	
**65) Interviewer [MS]: yeah *overlap***		
66) Interviewee [XXX]: some of them take some local herbs	**66. Herbal SM (yes, local herbs)**	
**67) Interviewer [MS]: ahhh ha**		
68) Interviewee [XXX]: local herbs and then some of them take erm erm drugs I call it like Pika you know Pika	**68. Herbal SM (local herbs, e.g., 'Pika')**	
**69) Interviewer [MS]: mhmm**		
70) Interviewee [XXX]: yeah some of them take erm drugs in form of maybe they have pika some of them take drugs for the mmm what do you call this epigastric erm	**70. Herbal SM ('Pika', other)**	
71) *silence*		
**72) Interviewer [MS]: Hello?**		
73) Interviewee [XXX]: maybe for *unclear word* symptoms *unclear word* sorry there's a call coming inside		

74) *overlapping speech*<br>75) Interviewee [XXX]: Are you hearing me?<br>**76) Interviewer [MS]: yes yes the signal**<br>77) Interviewee [XXX]: *overlapping speech* have diarrhoea<br>**78) Interviewer [MS]: yeah**<br>79) Interviewee [XXX]: will have diarrhoea and take antibiotics for diarrhoea. Some of them will take em drugs for um acidi hyperacidity in pregnancy, they know what to take some of them already maybe before the pregnancy they already having em epigastric pain instead of them to come to the hospital they continue with the drug that might be having some different um aliment, but they will think its because of pain peptic ulcer<br>**80) Interviewer [MS]: mhmm**<br>81) Interviewee [XXX]: that is why they want to take the drug for the pain thinking that it's a peptic ulcer.<br>**82) Interviewer [MS]: mhm okay *unclear speech* okay do you know of any methods that can identify self-medication of antibiotics in pregnant women? So when a pregnant woman takes antibiotics without you knowing**<br>**83)** Interviewee [XXX]: eh there is no its not possible<br>84) **Interviewer [MS]: Mhmm okay**<br>85) Interviewee [XXX]: I don't know any I don't of any<br>**86) Interviewer [MS]: Okay. Do you think it could be useful to have like a simple rapid test or tool that could help identify pregnant women who may be misusing antibiotics without us knowing?**<br>87) Interviewee [XXX]: yes it would be very useful yes<br>**88) Interviewer [MS]: okay dya have any idea of how that might work?**<br>89) Interviewee [XXX]: yes it could work through the theeeee we call them *unclear word ?issues* those are the community health extension workers<br>**90) Interviewer [MS]: mhmm**<br>91) Interviewee [XXX]: They are the ones that are very close to these women the communities. They can reach out to these women and then ask them if they are have taken antibiotics or guide them, we can get them we can get to those pregnant women through those people in the community<br>**92) Interviewer [MS]: mhmm**<br>93) Interviewee [XXX]: Those are the healt community health extension workers<br>**94) Interviewer [MS]: mhmm**	**79. Herbal SM (motivation, condition, previous herbal treatment)**<br><br>**81. Herbal SM (motivation, condition)**<br><br>**83. Detecting SM (not possible, not aware of any method)**<br><br>**87. Detecting SM (rapid test useful)**<br><br>**89. Detecting SM (rapid test, CHEWs)**<br>**91. Detecting SM (direct questioning, by CHEWs)**	**[5] Detecting SM (methods, tools, questionnaire, HCWs) – also see 101**

95) Interviewee [XXX]: and then even the patent erm drug sellers<br>**96) Interviewer [MS]: mhmm**<br>97) Interviewee [XXX]: The patent drug sellers that are very close to the community, there drugs are very very cheap, then there are some organisations that ermm help women that are sick there not hospital based not health care workers those ones they have drugs that they get when they get when they have drugs that when you go there for assistant they can just give you drugs without any without prescription<br>**98) Interviewer [MS]: mhmm**<br>99) Interviewee [XXX]: because they have the money maybe from an organisation from an NGO who gave them money to buy this drug, so when you go there and say you are not well they have they have any drug they give it you so you can go through those channels to be able to get eh whatever want to do<br>**100) Interviewer [MS]: okay so were talking about as well if they do you think it would be useful if they or you or the hospital had like some kind of test or questionnaire that would help aid with identifying like pregnant women who are taking antibiotics without us knowing**<br>101) Interviewee [XXX]: Its possible yes<br>**102) Interviewer [MS]: Okay would you be interested in using such a tool?**<br>**103)** Interviewee [XXX]: **If mm its poss yes**<br>**104) Interviewer [MS]: yeah *overlap***<br>105) Interviewee [XXX]: No problem<br>106) **Interviewer [MS]: mm were not saying like you know weve not got an exact example of one were just saying if theres like a tool or questionnaire or rapid test or a lab test that could be used, would you be interested in using it? Its just kind of**<br>107) Interviewee [XXX]: *overlapping speech* yeah yeah<br>**108) Interviewer [MS]: discuss that mmm so just say if we had such a tool do you think it would be useful to be used within antenatal care settings like you were saying or during routine appointments, or like during in A&E and things like that?**<br>**109)** Interviewee [XXX]: yes<br>**110) Interviewer [MS]: Okay and dya think it would be useful for such a tool to be like mobile or easy to use without internet?**<br>**111)** Interviewee [XXX]: Maybe mobile but not internet	**93. Detecting SM (CHEWs)**<br><br>**97. SM (patent drug sellers, other non-medical organisations)**<br><br>**99. SM (non-medical organisations, provide drugs)**<br><br>**101. Detecting SM (questionnaire, yes)**	**[9] SM (role of non-medical entities, organisations)**

**112) Interviewer [MS]: okay**	**109. Detecting SM (setting, antenatal)**	
**113) *overlapping speech***		
114) Interviewee [XXX]: *unclear speech* those people might not have internet service and they might not be educated enough		
**115) Interviewer [MS]: mhmm mhmm so dya have any ideas of how that might work? Like what kind of tool we could use..for that**	**111. Detecting SM (mobile device, no internet)**	**[10] Detecting SM (technological issues)**
116) Interviewee [XXX]: mm urmm you can use the mobile maybe through whatsapp		
**117) Interviewer [MS]: mhmm**		
118) Interviewee [XXX]: If the whatsapp group or whatsapp and we can also get through the community leaders the community leaders the religious leaders traditional leaders	**114. Detecting SM (no internet, lack of educ)**	
**119) Interviewer [MS]: mhmm okay okay that could work urm have you come across any methods or guidelines which might help detect side effects of antibiotic self-medication in pregnant women?**	**116. Detecting SM (mobile, WhatsApp)**	
**120)** Interviewee [XXX]: No	**118. **Detecting SM (WhatsApp, community & religious leaders)**	
**121) Interviewer [MS]: No okay**		
122) Interviewee [XXX]: no		
123) **Interviewer [MS]: So as we know antibiotics can cause side effects like upset stomach, or you know feeling very unwell do you think the presence of such side effects in a woman is clear that their taking antibiotics?**		
124) Interviewee [XXX]: yes yes definitely		
**125) Interviewer [MS]: mm do you have any examples? Or like how would you know? What kind of side effects you might see**		
126) Interviewee [XXX]: eh sometimes they come with em *unclear word ?gaitas* symptoms		
**127) Interviewer [MS]: mhm**		
128) Interviewee [XXX]: they come with gaitas symptoms that you know they must have taken some antibiotics although most of the time if its not the doctor that prescribed they are very silent about it they will not come out and say this is where I got this drug from or ive taken antibiotics except if you dig deep	**124. Detecting SM (side effects, yes)**	**[7] Detecting SM (clinical issues – side effects…) – also see 142-148**
**129) Interviewer [MS]: mhm**		
130) Interviewee [XXX]: and they have the confidence in you for they can come out and tell you this what they have taken. Usually they silent and they will not even let you know that this is what they had taken	**128. Detecting SM (side effects, indicator)**	

**131) Interviewer [MS]: mhm mhm okay so it just depends on the person and the relationship that**<br>132) Interviewee [XXX]: yeah *overlapping*<br>**133) Interviewer [MS]: you have with them *overlapping***<br>134) Interviewee [XXX]: yes yes<br>**135) Interviewer [MS]: So do you know any pregnant women who have developed side effects of antibiotic self-medication?**<br>136) Interviewee [XXX]: emmm no no no<br>**137) Interviewer [MS]: okay**<br>138) Interviewee [XXX]: mmm<br>139) **Interviewer [MS]: That's fine and do you know any methods or guidelines or protocols whicto manage antibiotic self medication in pregnant women?**<br>140) Interviewee [XXX]: No<br>**141) Interviewer [MS]: Okay, this is the last question so its very specific to so theres a specific area of where pregnant women who have self medicated with antibiotics might develop like signs of memory loss, or forgetfullness, do you know of any management plans or options that would happen if that happened, if someone as a side effect had memory loss or forgetfullness?**<br>142) Interviewee [XXX]: no because we don't have any specific method to detect<br>**143) Interviewer [MS]: mhmm**<br>144) Interviewee [XXX]: the side effects of the antibiotics<br>**145) Interviewer [MS]: mhm**<br>146) Interviewee [XXX]: only find out what I said with personal communication<br>**147) Interviewer [MS]: mhmm**<br>148) Interviewee [XXX]: there is no way or method available for us to detect this even taking the antibiotic let alone that it has side effects no<br>**149) Interviewer [MS]: mhm**<br>150) Interviewee [XXX]: it doesn't we don't have that<br>**151) Interviewer [MS]: mhm okay that's great, thank you so much for answering all the questions that's really great and really helpful and we appreciate you taking part, urm do you have any questions about anything?**<br>152) Interviewee [XXX]: mmm I don't know does em in your place do you have something like that do	**130. Detecting SM (side effects, *patient silent on medication use)**<br><br>**136. Side effects from SM (don't know any patients)**<br><br>**140. SM guidelines (no)**<br><br>**142. Neurological side effects from SM (no, can't detect)**<br><br>**146/148. Detecting SM difficult (no available method, need for personal communication) [direct quest]**	**[6] Detecting SM (GUIDELINES)**

you have a guideline to detect if a pregnant woman has taken antibiotics unprescribed?

153) Interviewer [MS]: It varies, everything varies

154) Interviewee [XXX]: *laughs* *overlap*

155) Interviewer [MS]: I can't say specifically I cant say specifically urm but as you know this research is kind of based more like find out kind of whats going on in like within Nigeria

156) Interviewee [XXX]: mhmm *overlap*

157) Interviewer [MS]: *unclear speech* urm it kind of varies and its different so I can't say specifically cuse everything varies and I wouldn't really know urm but yeah dya have any other questions about anything?

158) Interviewee [XXX]:no no no really thank you

159) End of interview asked if using an airtime card for the call. Interviewee confirms using wifi for call.

160) Advised will send consent form back to interviewee and had brief discussion about next steps for participants colleagues taking part in the research.

161) End of interview.
