# Supplementary material for: Advancing the safe motherhood initiative: A qualitative and sentiment analysis of local physician’s perspectives on antibiotic self-medication during pregnancy in a low- and middle-income country
Source: PLOS Glob Public Health. 2025 Sep 12;5(9):e0004794. doi: 10.1371/journal.pgph.0004794 (PMC12431270; doi:10.1371/journal.pgph.0004794)
Supplement: S1 File — Transcript 4 (CODES & THEMES by KU).pdf. Transcript 6 (CODES & THEMES by KU).pdf. Transcript 7 (CODES & THEMES, by KU).pdf. Transcript 8 (CODES & THEMES by KU).pdf. Transcript 9 (CODES & THEMES by KU).pdf. Transcript 10 (CODES & THEMES by KU).pdf. Transcript 11 (CODES & THEMES, by KU).pdf. Transcript 12 (CODES & THEMES by KU).pdf. Transcript 13 (CODES & THEMES by KU).pdf. Transcript 14 (CODED & THEMES by KU).pdf. Transcript 15_b (CODED & THEMES by KU). pdf. Transcript 16 (CODES & THEMES by KU).pdf. Transcript 17 (CODES & THEMES by KU).pdf. Transcript 18 (CODES & THEMES by KU).pdf. Transcript 19 (CODES & THEMES by HK).pdf. Transcript 20 (CODES & THEMES by HK).pdf. Transcript 21_b (CODES & THEMES by HK).pdfTranscript 22 (CODES & THEMES by HK).pdf. Transcript 25 (CODES & THEMES by HK).pdf. Transcript 27 (CODES & THEMES by HK).pdf. Transcript Sn1 (CODES & THEMES by RS).pdf Transcript Sn6 (pt3) (CODES & THEMES by RS).pdf. Transcript Sn15_a (CODES & THEMES by RS).pdf. Transcript SN17 (pt3) (CODES & THEMES by RS).pd. Transcript Sn21_a (CODES & THEMES by RS).pdf. (ZIP) [file pgph.0004794.s001.zip › Transcript 15_b (CODED & THEMES by KU).pdf]

Text	Initial codes	Themes
**Transcription interview 15**<br>**Interviewee: XXX**<br><br>**Interviewer: (MS), Research Assistant**<br>**Number of speakers :2**<br>**Time: 6.34pm London time**<br>**Length of interview recording: 16 minutes 19 seconds**<br>**Date: 4th May 2023**<br><br>**Note: MS went through PIS and Consent form. Consent obtained on Zoom recording, participant consented to take part in the study. Confirmed participant using Wi-Fi for call.**		
1) **Interviewer [MS]: Do you prescribe antibiotics to pregnant women?**	**2. Prescribe antibiotics (yes)**	**[1] PRESCRIBING ANTIBIOTICS**
**2)** Interviewee [XXX]: Yes I do		
3) **Interviewer [MS]: Okay how often do you prescribe them to pregnant women, how many times a week?**		
**4)** Interviewee [XXX]: mmm 4-5 times not frequently	**4. Prescribe antibiotics (frequency)**	
**5) Interviewer [MS]: Okay. How long have you been prescribing them to women for?**		
6) Interviewee [XXX]: Up to 12 years now		
7) **Interviewer [MS]: Okay and what are the 3 most common medical problems that you prescribe antibiotics for?**	**6. Prescribe antibiotics (duration, years)**	
**8)** Interviewee [XXX]: Upper respiratory tract infections	**8. Prescribe antibiotics (conditions)**	
9) **Interviewer [MS]: Mhmm**		
**10)** Interviewee [XXX]: Urinary tract infections commonly these are the ones commonly encounter in pregnant women	**10. Prescribe antibiotics (conditions, UTI)**	
**11) Interviewer [MS]: Mhmm. Do you use any guidelines when you're prescribing antibiotics?**		
12) Interviewee [XXX]: mm not frequently uh generally we look at the eh sensitivity pattern in our environment common pathogens you know and then sometimes we *unclear speech* some eh microbiological investigations before we carry *unclear speech* for them	**12. Prescribe antibiotics (guidelines, not usually)**	

**13) Interviewer [MS]: Okay and where do find that pregnant women generally get their antibiotics from?**		
14) Interviewee [XXX]: yeah in my place in my country some of them before they see you they will *unclear words* some of them access these drugs from pharmacy shops buy on their own because even the prescription medications here is easily accessible across the counter even without prescription so most times theyy self prescribe before they come to the hospital	**14. Obtaining antibiotics (pharmacy, OTC)**	**[2] OBTAINING ANTIBIOTICS**
15) **Interviewer [MS]: mhm**		
**16)** Interviewee [XXX]: and then sometimes those who are more enlightened among them they do come to the hospital	**16, 18. Obtaining antibiotics (hospital, hospital pharmacy)**	
17) **Interviewer [MS]: mhmm**		
**18)** Interviewee [XXX]: and then access it from the hospital pharmacy		
**19) Interviewer [MS]: Mhmm, so are you aware of pregnant women who have take antibiotics that arent prescribed for them? Like you said, do you see that a lot?**		
20) Interviewee [XXX]: yeah of course I do	**20. SM (aware of)**	
21) **Interviewer [MS]: dya have any examples of when that happens**		**[3] SM (AWARENESS, MOTIVATION**
22) Interviewee [XXX]: yeah for instance when they have a when they have urm upper respiratory tract infection they commonly use eh *unclear words* antibiotics that gives a *unclear words* also sometimes have a general gastrointestinal problems they usually go for metronidazole	**22. SM (examples, medical conditions)**	
23) **Interviewer [MS]: mhm**		
24) Interviewee [XXX]: on their own before they come to see you	**24. SM (before visiting doctor)**	
25) **Interviewer [MS]: mhm *overlapping speech***		
26) Interviewee [XXX]: is when the problem persists sometimes or when they have a chance to see you at the antenatal clinic	**26. SM occurs, unless problem persists, able to see a doctor**	
27) **Interviewer [MS]: mhm**		
**28)** Interviewee [XXX]: and they may have to report and when you poke further you find that they put themselves on these antibiotics	**28. SM (doctor probes, queries)**	
**29) Interviewer [MS]: mhm mhm so do you find are you aware of pregnant women take like herbal medications or alternative medications that work like antibiotics?**		
30) Interviewee [XXX]: well that is not a common finding usually for even the uneducated women	**30. Herbal SM (not common, patients**	

here they they are usually careful about most of them are careful about what taking herbal medications during pregnancy a few that do take it do not take it for infection they usually do take it as um some of them are advised by their friends that em usually for some reasons they don't want to have babys that have large birthweights	careful, social pressure)	**[4] HERBAL SM (rarity, motivation)**
31) **Interviewer [MS]: mhm**		
32) Interviewee [XXX]: because some of them sometimes they encounter difficulties shoulder dystocia or some other problems during delivery so sometimes they used to buy medication just to cause uterine growth restriction for their babies okay so that the babies do not get to have large birth weights	**32. Herbal SM (for birth, delivery problems)**	
33) **Interviewer [MS]: mhm**		
34) Interviewee [XXX]: so that is the major reason they use for the few that use herbal medications in pregnancy they don't usually use it for infections	**34. Herbal SM (not for infection)**	
35) **Interviewer [MS]: Mhmm okay. Do you know any methods that can identify or detect self-medication of antibiotics in pregnant women?**		
36) Interviewee [XXX]: I don't Ive not come across any	**36. Detecting SM (no methods)**	
**37) Interviewer [MS]: Okay so how do you normally find out that someone is self medicating? With antibiotics**		
38) Interviewee [XXX]: usually do not have a protocol in place ahhh to identify those who do that usually its when usually on general eh interaction with their patients *unclear speech* with us not that you go out to look for it usually find when they come with complaint I want to know eh which measure they have taken	**38. Detecting SM (no existing protocols, doctor may query patient)**	**[5] DETECTING SM (methods, setting)**
39) **Interviewer [MS]: mhm**		
**40)** Interviewee [XXX]:on their own *unclear speech* find that out		
**41) Interviewer [MS]: Mhm. Dya think it could be useful to have like a simple rapid test or a lab tool or questionnaire that could help identify pregnant women who might be misusing antibiotics without us knowing?**		
42) Interviewee [XXX]: yeah a questionnaire yes a questionnaire I don't know if any rapid test that does that	**42. Detecting SM (rapid test, questionnaire, yes)**	
43) **Interviewer [MS]: Mhm. So If there was a questionnaire or something avaliable would you be interested in using it?**		

44) Interviewee [XXX]: sure sure	44. Detecting SM (questionnaire, will use it)	
**45) Interviewer [MS]: Okay and do you think such a tool could be used within antenatal care settings, or during routine appointments, or like even in A&E?**		
46) Interviewee [XXX]: yeah a good number of them will well some will	46. Detecting SM (settings)	
47) **Interviewer [MS]: mhm**		
**48)** Interviewee [XXX]: *mumbled speech* *unclear speech* many of them will respond		
49) **Interviewer [MS]: Mhmm**		
**50)** Interviewee [XXX]: *overlapping speech*		
**51) Interviewer [MS]: and dya think**		
**52)** Interviewee [XXX]: *overlapping speech* sure sure		
**53) Interviewer [MS]: okay, dya think it would be useful for like a questionnaire or a tool or a test to be kind of mobile or remote easy to use, like not having to use electricity or internet things like that**		
54) Interviewee [XXX]: yeah most of the women do have smartphones	54. Detecting SM (smartphones useful, common)	[10] DETECTING SM (TECH ISSUES)
55) **Interviewer [MS]: mhmm**		
56) Interviewee [XXX]: I would say 50 60 percent will have smart phones otherwise *slightly mumbled speech* you have phones that may not be smart phones	56. Detecting SM (smartphones useful, common)	
57) **Interviewer [MS]: mhm**		
58) Interviewee [XXX]: *mumbled speech unclear* rare practice a good percentage were able to because I know some parts like eh those of them that are diabetic we got we have them record their sugars monitoring and all that simple *unclear word* help them record them then they come to the hospital *unclear speech* to follow them up so yes	58. Detecting SM (smartphones already being used in care provision)	
**59) Interviewer [MS]: mhmm, okay so like in terms of using a potential test or questionnaire for use in the hospital dya think it would need to be something that's quite easy to use by like the staff aswell?**		
**60)** Interviewee [XXX]: *overlapping speech*		
61) **Interviewer [MS]: and within the hospital**		
**62)** Interviewee [XXX]: yeah easy to use that is key yeah	62. Detection (test must be easy to use)	
63) **Interviewer [MS]: okay so dya have any ideas of how that could work?**		
64) Interviewee [XXX]: ah well um the women are if by the time that eh well motivated patients who will		

agree to take part in the study I have a good number of them that get responses for us do that *mumbled speech* they get these records that are *mumbled speech* so likewise I believe that uh if they are *mumbled unclear speech* such as that they will be able to cooperate	**64. Detecting SM (patients will cooperate)**	
65) **Interviewer [MS]: mhm**		
**66)** Interviewee [XXX]: and do the monitoring themselves at home especially if the app is not so complex for use	**66. Detecting SM (app, self-monitoring, at home)**	
**67) Interviewer [MS]: Mhmm okay. So have you come across any methods or guidelines that could help detect side effects of antibiotic self-medication in pregnant women?**		
68) Interviewee [XXX]: No I have not come across that	**68. Side effects of SM (no guidelines)**	**[6] DETECTING SM (GUIDELINES) – also see 80**
**69) Interviewer [MS]: Okay, so we know that like antibiotics can cause side effects *unclear word* like stomach upset or feeling sick or nausea that kind of thing, do you think the presence of such antibiotics in a patient is clear evidence that the patient is taking antibiotics when theyre having side effects like that**		
70) Interviewee [XXX]: ahh most of the anside effects are not specific so you may not really if the patient doesn't admit to using antibiotics *unclear speech* side effects may not be really just enough for you to	**70. Side effects of SM (ambiguous, not reliable indicator of SM)**	**[7] DETECTING SM (CLINICAL ISSUES, side effects..] – also see 82, 84**
71) **Interviewer [MS]: mhmm**		
**72)** Interviewee [XXX]: say the patient is *unclear word* self medication		
**73) Interviewer [MS]: mhmm mhmm, so do you know any pregnant women that have suspected to developed, like have side effects of antibiotic self-medication have you ever seen that?**		
74) Interviewee [XXX]: yeah sure we do see them sometimes you youu they don't really understand whats happening to them actually maybe the side effect that they will report to you and when you probe further you now ask what they have been taking on their own and find out they've been self medicating	**74. Side effects of SM (observed in patients, queried by doctor)**	
75) **Interviewer [MS]: mhmm mhmm**		
**76)** Interviewee [XXX]: *overlapping speech unclear* the side effects that brings them that is the complaint they give you at the clinic	**76. Side effects of SM (reason for clinic visit)**	
77) **Interviewer [MS]: mhmm**		
78) Interviewee [XXX]: yes		

79) **Interviewer [MS]:Okay. So again do you know of kind of similarly do you know any methods or guidelines or protocols that manage antibiotic self medication in pregnant women?**		
80) Interviewee [XXX]: no	**80. SM guidelines (none)**	
**81) Interviewer [MS]: no okay that's fine so this is like a specific area looking at sometimes women that have self medicated with antibiotics might develop side effects such as like memory loss, or forgetfulness have you ever seen that?**		
**82)** Interviewee [XXX]: noo	**82. Side effects, neurological (no, none)**	
**83) Interviewer [MS]: okay dya know like any management options so if someone came to you and they've self medicated with antibiotics and they developed memory loss or forgetfullness dya know any kind of management options that would take place?**		
84) Interviewee [XXX]: no ive not encountered that and ive not had reason to probe and then make some research in that area	**84. Side effects, neurological (no, none)**	
85) **Interviewer [MS]: Okay *overlapping speech***		
**86)** Interviewee [XXX]: *overlapping speech* I have not		
**87) Interviewer [MS]: okay okay that's fine that's absolutely fine thank you, so that's all my questions thank you very much do you have any questions?**		
88) Interviewee [XXX]: ah okay I would like to know is there is there a design for a work in this area that incorporates um people from um maybe *unclear speech* okay I looked at the title I was wondering whether that was such a research going on and eh the centres you are using for the work		
**89) Interviewer [MS]: so I cant we cant say the other centres so obviously weve got your details from like *name of dr* and things like that but we cant kind of disclose the other centres that are involved at the moment um but at the end of the study a paper will be released cuse cuse confidentiality and**		
90) Interviewee [XXX]: okay *overlapping speech*		
**91) Interviewer [MS]: things like that so at the end of the when the pa when everythings finished um well probably gonna write a paper and well be able to kind of release the paper so then you can see the findings through that**		
92) Interviewee [XXX]: its alright		
93) **Interviewer [MS]: is that okay?**		

**94)** Interviewee [XXX]: yes no problem<br><br>95) No other questions asked. Advised consent form will be sent to participant and participant advised can email or message RA, if got any other questions. Thanked for taking part in the study.		
