# Supplementary material for: Advancing the safe motherhood initiative: A qualitative and sentiment analysis of local physician’s perspectives on antibiotic self-medication during pregnancy in a low- and middle-income country
Source: PLOS Glob Public Health. 2025 Sep 12;5(9):e0004794. doi: 10.1371/journal.pgph.0004794 (PMC12431270; doi:10.1371/journal.pgph.0004794)
Supplement: S1 File — Transcript 4 (CODES & THEMES by KU).pdf. Transcript 6 (CODES & THEMES by KU).pdf. Transcript 7 (CODES & THEMES, by KU).pdf. Transcript 8 (CODES & THEMES by KU).pdf. Transcript 9 (CODES & THEMES by KU).pdf. Transcript 10 (CODES & THEMES by KU).pdf. Transcript 11 (CODES & THEMES, by KU).pdf. Transcript 12 (CODES & THEMES by KU).pdf. Transcript 13 (CODES & THEMES by KU).pdf. Transcript 14 (CODED & THEMES by KU).pdf. Transcript 15_b (CODED & THEMES by KU). pdf. Transcript 16 (CODES & THEMES by KU).pdf. Transcript 17 (CODES & THEMES by KU).pdf. Transcript 18 (CODES & THEMES by KU).pdf. Transcript 19 (CODES & THEMES by HK).pdf. Transcript 20 (CODES & THEMES by HK).pdf. Transcript 21_b (CODES & THEMES by HK).pdfTranscript 22 (CODES & THEMES by HK).pdf. Transcript 25 (CODES & THEMES by HK).pdf. Transcript 27 (CODES & THEMES by HK).pdf. Transcript Sn1 (CODES & THEMES by RS).pdf Transcript Sn6 (pt3) (CODES & THEMES by RS).pdf. Transcript Sn15_a (CODES & THEMES by RS).pdf. Transcript SN17 (pt3) (CODES & THEMES by RS).pd. Transcript Sn21_a (CODES & THEMES by RS).pdf. (ZIP) [file pgph.0004794.s001.zip › Transcript 16 (CODES & THEMES by KU).pdf]

Transcript	Initial codes	Themes
**Transcription interview 16**<br>**Interviewee: XXX**<br>**SN-21**<br>**Interviewer: (MS), Research Assistant**<br>**Number of speakers :2**<br>**Time: 7.24pm**<br>**Length of interview recording: 21 minutes 52 seconds**<br>**Date: 4th May 2023**<br>Note: Consent obtained on zoom call prior to commencing interview. Consent obtained to audio & video record the call. Participant advised using Wifi for call.		
1) **Interviewer [MS]: Do you prescribe antibiotics to pregnant women?**<br>2) Interviewee [XXX]: we do, when indicated<br>**3) Interviewer [MS]: Okay and how long have you been a prescriber for?**<br>4) Interviewee [XXX]: said?<br>**5) Interviewer [MS]: How long**<br>6) Interviewee [XXX]: How many *overlapping speech*<br>**7) Interviewer [MS]: How long have you been prescribing antibiotics for?**	**2. Prescribe antibiotics (when needed)**	**[1] PRESCRIBING**
8) Interviewee [XXX]: ah since ive been practising *unclear speech* over 10 years<br>9) **Interviewer [MS]: Okay and how many times a week do you prescribe antibiotic?**<br>10) Interviewee [XXX]: *unclear word* times a week<br>**11) Interviewer [MS]: mhmm**<br>**12) *Silence for a few seconds***	**8. Prescribe antibiotics (years/duration)**	
13) Interviewee [XXX]: maybe once or twice<br>14) **Interviewer [MS]: Okay. What are the 3 most common medical problems that you prescribe antibiotics for?**<br>15) Interviewee [XXX]: I didn't get it<br>**16) Interviewer [MS]: What are the 3 most common medical problems that you prescribe antibiotics for?**	**13. Prescribe antibiotics (freq)**	
17) Interviewee [XXX]: okay the main reason for antibiotics is in pregnancy<br>**18) Interviewer [MS]: mhm**<br>19) Interviewee [XXX]: In pregnancy when they prescribe antibiotics for women that come down with fever<br>**20) Interviewer [MS]: mhmm**<br>21) Interviewee [XXX]: *unclear speech* pick up infection in pregnancy commonly or those who come in with vaginal discharge in pregnancy<br>**22) Interviewer [MS]: mhm okay and do you use any guidelines when you prescribe antibiotics?**	**17/18/21. Prescribe antibiotics (reasons, conditions)**	

23) Interviewee [XXX]: guidelines guidelines we had you know in my centre *name of hospital* we have a labour ward protocol<br>**24) Interviewer [MS]: mhm**	**23. Prescribe antibiotics (guidelines, <u>yes</u>).**	
25) Interviewee [XXX]: we have a labour ward protocol but it does not actually have antibiotic guideline we have a protocol that shows that tells us what to do *unclear word* we don't have a clear cut antibiotic guideline that that's *unclear mumbled speech*<br>**26) Interviewer [MS]: okay so where do you find**<br>27) Interviewee [XXX]: *overlapping speech*<br>**28) Interviewer [MS]: Go on sorry**	**25. Prescribe antibiotics (guidelines, protocol, <u>yes</u>).**	
29) Interviewee [XXX]: sometimes we have to go go with the British National Formarly<br>**30) Interviewer [MS]: Mhm *overlapping speech***<br>31) Interviewee [XXX]: *unclear speech overlapping*<br>**32) Interviewer [MS]: Okay, so where do find that pregnant women generally get their antibiotics from?**<br>33) Interviewee [XXX]: *unclear mumbled speech* where?<br>**34) Interviewer [MS]: Where? Yeah**<br>35) Interviewee [XXX]: so where do I get the *unclear word*<br>**36) Interviewer [MS]: Where do you pregnant women generally get their antibiotics from?**<br>37) Interviewee [XXX]: sorry im not clear about the question<br>**38) Interviewer [MS]: where where do pregnant women generally get their antibiotics from?**	**29. Prescribe antibiotics (guidelines, source)**	
39) Interviewee [XXX]: where do they get antibiotics from?<br>**40) Interviewer [MS]: mhm**<br>41) Interviewee [XXX]: where do they get the antibiotics from<br>**42) Interviewer [MS]: yeah**<br>43) Interviewee [XXX]: from the hospital store<br>**44) Interviewer [MS]: okay**<br>45) Interviewee [XXX]: *overlapping unclear speech*<br>**46) Interviewer [MS]: uhuh, okay so**	**39/41/43. Obtaining antibiotics (from hospital)**	**[2] OBTAINING**
47) Interviewee [XXX]: *overlapping speech* and from outside pharmacist too<br>48) **Interviewer [MS]: Okay, so are you aware of any pregnant women that sometimes take antibiotics that havent been prescribed for them?**	**47. Obtain antibiotics (from external pharmacist)**	
49) Interviewee [XXX]: ah in this clim in this part of the world many women take antibiotics that are not prescribed in this part of the world especially my area *unclear speech* of nigeria many *unclear words* take antibiotics that not prescribed for them<br>**50) Interviewer [MS]: mhm do you have any examples like where do people get them from then**<br>51) Interviewee [XXX]: example of antibiotic they take?	**49. SM with ATB (yes, many women)**	**[3] SELF-MEDICATION**

**52) Interviewer [MS]: *overlapping speech* or like where where do they get them from if there not getting them prescribed**		
53) Interviewee [XXX]: okay *speaking fast unclear speech*		
**54) Interviewer [MS]: mhm**		
55) Interviewee [XXX]: okay they take antibiotics when they when they when they feel that they have an aliment	**55. SM with ATB (motivation)**	
**56) Interviewer [MS]: mhm**		
57) Interviewee [XXX]: they come down with headache they take antibiotics	**57. SM with ATB (motivation)**	
**58) Interviewer [MS]: mhm**		
59) Interviewee [XXX]: when they come down with weakness they take antibiotics theres this local theres this common place *unclear word* that when someone feels headache mostly likely its an infection and because of that most of them take unprescribed antibiotics	**59. SM with ATB (motivation)**	
**60) Interviewer [MS]: Okay okay. Urm do you know of any pregnant women who might take like herbal preparations or alternative medications that work like antibiotics?**		
61) Interviewee [XXX]: I didn't get that		
**62) Interviewer [MS]: Have you ever seen any pregnant women who might take like herbal preparations or alternative medications that could work like antibiotics**		
63) Interviewee [XXX]: Just like I said earlier in this part of the world to herbal medication is a common place	**63. Herbal SM (common**) *[note: relevant to TBAs)*	**[4] HERBAL SELF-MEDICATION**
**64) Interviewer [MS]: mhm**		
65) Interviewee [XXX]: not all of them herbal medications is a common place they take them on their own	**65. Herbal SM (common, not all patients)**	
**66) Interviewer [MS]: mhm**		
67) Interviewee [XXX]: *unclear word ?un or not?* prescribed		
**68) Interviewer [MS]: Dya have any examples of herbal medications that they take**		
69) Interviewee [XXX]: the herbal medication doesn't have names	**69. Herbal SM (identification)**	
**70) Interviewer [MS]: okay**		
71) Interviewee [XXX]: *overlapping speech* they are special they are special they are special herbs made from the leaves *unclear words* for various reasons some will tell them the herbal medications will help them control or reduce the size of the baby	**71. Herbal SM (type, content, motivation)**	
**72) Interviewer [MS]: mhm**		
**73)** Interviewee [XXX]: some will give them the medications and tell them the medications will help them condense some infection in pregnancy	**73. Herbal SM (motivation)**	
**74) Interviewer [MS]: mhm**	**75. Herbal SM (type, content)**	

75) Interviewee [XXX]: but really you don’t know the content of the medications		
**76) Interviewer [MS]: mhm**		
77) Interviewee [XXX]: noone knows the content	79. Herbal SM (side effects)	
**78) Interviewer [MS]: mhm**		
79) Interviewee [XXX]: and the effect side effect *unclear quick speech* of those herbal medications are not known		
**80) Interviewer [MS]: mhm mhm. Do you know of any methods that detect or identify self-medication of antibiotic in pregnant women?**		
81) Interviewee [XXX]: Do I know		
**82) Interviewer [MS]: any methods that might detect or identify self-medication of antibiotics in pregnant women?**	83/85. Detecting SM (difficult)	[5] DETECTING SELF-MEDICATION
83) Interviewee [XXX]: It is it is quite difficult		
**84) Interviewer [MS]: mhm**		
85) Interviewee [XXX]: mm quite difficult		
**86) Interviewer [MS]: okay**		
87) Interviewee [XXX]: *overlapping speech* I don’t let me let me get it clear identifying		
**88) Interviewer [MS]: so if someones self medicating with antibiotics**		
89) Interviewee [XXX]: okay *overlapping speech*	91/93. Detecting SM (method, direct questioning)	
**90) Interviewer [MS]: do you have methods that might identify that**		
91) Interviewee [XXX]: the its only by questioning		
**92) Interviewer [MS]: mhm**	95. Detecting SM (questioning, while taking histories)	
93) Interviewee [XXX]: *overlapping speech* questioning them		
**94) Interviewer [MS]: mhm**		
95) Interviewee [XXX]: so when someone presents with symptoms *fast speech unclear speech* have you been on antibiotics have you been on have you been on its only during during the time we are doing histories from them that will try to *unclear word* whether there is anyone been on antibiotics		
**96) Interviewer [MS]: mhm**	99. Detecting SM (method, questioning)	
97) Interviewee [XXX]:mmm		
**98) Interviewer [MS]: *started to speak then overlapping speech***		
99) Interviewee [XXX]: its only from questioning that we can get that from them mm		
**100) Interviewer [MS]: yeah. do you think it could be useful to have like a simple rapid test or tool or questionnaire that could help identify pregnant women who might be misusing antibiotics without us knowing?**	101. Detecting SM (need for a tool)	

101) Interviewee [XXX]: it will it will be nice to have such a tool	103. Detecting SM (need for a tool/ unavailable)	
**102) Interviewer [MS]: okay**		
103) Interviewee [XXX]: *overlapping speech* *unclear speech* to identify those who have who might have been engaging such antibiotic self medication it will be nice but we don't have in our centre		
**104) Interviewer [MS]: mhm**		
105) Interviewee [XXX]: *overlapping speech*		
106) **Interviewer [MS]: Okay, so if there was such a tool available, would you be interested in using it?**	109/111. Detecting SM (tool unavailable)	
107) Interviewee [XXX]: is there what?		
**108) Interviewer [MS]: If there was such a tool or a proforma or questionnaire or a test available would you be interested in using it?**		
109) Interviewee [XXX]: we don't have any proforma questionnaire or such tools available in our centre		
**110) Interviewer [MS]:mhm**		
111) Interviewee [XXX]: mm *unclear speech* centre we don't have	115. Detecting SM (interested in using tool)	
**112) Interviewer [MS]: okay if you**		
113) Interviewee [XXX]: *overlapping speech*		
**114) Interviewer [MS]: did, would you be interested in using it?**		
115) Interviewee [XXX]: no if eh of course		
**116) Interviewer [MS]: okay**		
117) Interviewee [XXX]: *overlapping speech* *unclear speech* if it is avaliable it would be interesting	119. Detecting SM (setting)	
**118) Interviewer [MS]: okay. Dya think such a tool could be used within like antenatal care settings, or in routine appointments, or in A&E? Where dya think it would be best used?**	121/123. Detecting SM (setting, domestication, training)	
119) Interviewee [XXX]: *background noise* *unclear speech* such a tool incorporating such a tool in antenatal patients in antenatal care		
**120) Interviewer [MS]: mhm**		
121) Interviewee [XXX]: yes maybe helpful but obviously eh domesticating such may take some time	119/121. Detecting SM (tool, level of acceptance)	
**122) Interviewer [MS]: mhm**		
123) Interviewee [XXX]: domesticating such erm to in antenatal care may take some time		
**124) Interviewer [MS]: mhm, dya think**	127. Detecting SM (tool a good idea)	
125) Interviewee [XXX]: *overlapping unclear speech* acceptance maybe some consultants may not accept it whilst some may do		
**126) Interviewer [MS]: mhm**		
127) Interviewee [XXX]: but I think its generally a very nice thing it is		

**128) Interviewer [MS]: mhm**<br>129) Interviewee [XXX]: *unclear speech*<br>**130) Interviewer [MS]: Yeah. Dya think it would be useful for such a test or a tool to be like mobile or remote or easy to use without like internet or electricity? Kind of thing**<br>131) Interviewee [XXX]: I didn't I didn't get that<br>**132) Interviewer [MS]: Dya think that such a tool could be should be mobile or remote or easy to use without electricity or internet**<br>133) Interviewee [XXX]: yeahh I think by mail by mail maybe I think I would prefer it if it can come by mail<br>**134) Interviewer [MS]:Okay Okay. Dya have any idea how such a test could work?**<br>135) Interviewee [XXX]: how it could work<br>**136) Interviewer [MS]: yeah**<br>**137) *pause***<br>138) Interviewee [XXX]: I didn't get it how such test could work<br>**139) Interviewer [MS]: yeah like dya have an idea of what what kind of tool are you thinking of?**<br>140) Interviewee [XXX]: well for now I don't think I have a clear idea<br>**141) Interviewer [MS]: okay okay that's okay. Urm have you ever come across any methods or guidelines which can help detect side effects of antibiotic self-medication in pregnant women?**<br>142) Interviewee [XXX]: have I come across any guideline<br>**143) Interviewer [MS]: which can help**<br>144) Interviewee [XXX]: *overlapping speech*<br>**145) Interviewer [MS]: detect side effects of antibiotic**<br>146) Interviewee [XXX]: *overlapping speech* side effect<br>**147) Interviewer [MS]: medication**<br>148) Interviewee [XXX]: I don't really think so no<br>**149) Interviewer [MS]: Okay okay and as we know antibiotics can cause like side effects like stomach upset or not feeling well, do you think the presence of such side effects is clear when the patients taking antibiotics?**<br>150) Interviewee [XXX]: lets come again<br>**151) Interviewer [MS]: so some we know antibiotics can cause side effects do you think the presence of such side effects is clear when someones taking antibiotics**<br>152) Interviewee [XXX]: if if side effect is clear<br>**153) Interviewer [MS]: mhm from antibiotics**	**133. Detecting SM (access)**<br><br>**140. Detecting SM (type of tool, unclear)**<br><br>**142, 146, 148. Side effects of SM (guidelines, no)**	**[6] GUIDELINES (1/2)**

154) Interviewee [XXX]: from antibiotics		
**155) Interviewer [MS]: mhm yep**		
156) Interviewee [XXX]: so I don't know what to answer		
157) *overlapping speech*		
**158) Interviewer [MS]: its just say if you see a pregnant woman that's like having side effects from antibiotics**	**162, 164, 166, 168, 170. Side effects (patients complaints, ATB not working, ATB resistance)** [NOTE: NOT LINKED TO SM]	
159) Interviewee [XXX]: okay yeah		
160) *overlapping speech*		
**161) Interviewer [MS]: yeah is it obvious that its from the antibiotics?**		**[7] SIDE EFFECTS (1/2)**
162) Interviewee [XXX]: mmm just a few complain of urm *unclear word* complain those *unclear speech*		
**163) Interviewer [MS]: mhm**		
164) Interviewee [XXX]: *unclear speech* they complain of rashes all over their body		
**165) Interviewer [MS]: mhm mhm**		
166) Interviewee [XXX]: and some others I have when they complain of rashes some of complain of tingling sensations		
**167) Interviewer [MS]: mhm**		
168) Interviewee [XXX]: and some have urm complain that the antibiotics they are using are no longer working		
**169) Interviewer [MS]: mhm**		
170) Interviewee [XXX]: when they want to use this for a treatment or an aliment same antibiotics no longer working		
**171) Interviewer [MS]: okay**	**74. Side effects (patient symptoms) [NOTE: link to SM UNCLEAR]**	
172) Interviewee [XXX]: *overlapping speech* resistance		
**173) Interviewer [MS]: Okay. So do you know any pregnant women who have developed side effects from self medicating with antibiotics?**		
**174)** Interviewee [XXX]: any woman *unclear speech* that's just that's exactly what I *unclear fast speech* someone that developed rash and using *unclear word ?said* cream and developed urm someone developed rash using said cream	**74. Side effects (Yes, from Herbal SM) [LINKABLE TO SM?]**	
**175) Interviewer [MS]: from self medication?**		
176) Interviewee [XXX]: self medication yeah		
**177) Interviewer [MS]: Okay Okay**		
178) Interviewee [XXX]:and someone developed eh abdominal pain from using herbal medications	**180. SM Guidelines (no, don't know)**	**[6] GUIDELINES (2/2)**
**179) Interviewer [MS]: mhm okay. Urm and do you know any methods or guidelines or protocols that would manage antibiotic self medication in pregnant women?**		

180) Interviewee [XXX]: I don’t know I don’t know any yet<br>**181) Interviewer [MS]: Okay so this is very specific to sometimes some pregnant women develop urm like signs of memory loss or forgetfulness after self medicating with antibiotics. Have you ever seen that?**<br>182) Interviewee [XXX]: Ive not seen that<br>**183) Interviewer [MS]: dya know of any management options or kind of what would you do if someone had side effects such as like memory loss or forgetfulness?**<br>184) Interviewee [XXX]: urm when someone comes with like the one that comes with the rash<br>**185) Interviewer [MS]: mhm**<br>**186) *background noise***<br>187) Interviewee [XXX]: she will manage in the emergency room<br>**188) Interviewer [MS]: mhm**<br>189) Interviewee [XXX]: er er hydrocortisone given then<br>**190) Interviewer [MS]: mhm**<br>191) Interviewee [XXX]: and um hydrocortisone usually given them	**184, 187, 189, 191, 193, 195. Side effects (management of)**	**[7] SIDE EFFECTS (2/2)**
**192) Interviewer [MS]: mhm**<br>193) Interviewee [XXX]: treatment given to them usually symptomatic those that came *unclear speech*<br>**194) Interviewer [MS]: mhm**<br>195) Interviewee [XXX]: *unclear speech* they were given hydrocortisone and monitored closely<br>**196) Interviewer [MS]: mhm. So if someone had memory loss or forgetfulness as a side effect is there any management plans that you would do?**<br>197) Interviewee [XXX]: I don’t think ive had anyone that had memory loss forgetfulness taking self medication antibiotics<br>**198) Interviewer [MS]: okay that’s fine so that’s all my questions thank you very much for answering them that was really helpful**<br><br>199) End of interview questions<br>200) Participant asked if had any questions. Participant advised that antibiotics not routinely prescribed for pregnant women, given on indication. Advised that people can go to chemist for self medications, both pregnant and non-pregnant and occasionally when it doesn’t work they come to the clinic or further care. Further participant advised women with premature rupture of membranes, preterm women given	**197. Side effects (neurological, no, none) [NOTE: LINK TO SM UNCLEAR]**	

antibiotics in pregnancy and even after labour/postpartum continue antibiotics for 72 hours according to their protocol. Spoke about postnatal sepsis by nature of the environment. Confirmed study mainly about antenatal period/antenatal care at present.<br>201) Advised if participant has any other questions they have my details and consent form will be sent to them.		
