# Supplementary material for: Advancing the safe motherhood initiative: A qualitative and sentiment analysis of local physician’s perspectives on antibiotic self-medication during pregnancy in a low- and middle-income country
Source: PLOS Glob Public Health. 2025 Sep 12;5(9):e0004794. doi: 10.1371/journal.pgph.0004794 (PMC12431270; doi:10.1371/journal.pgph.0004794)
Supplement: S1 File — Transcript 4 (CODES & THEMES by KU).pdf. Transcript 6 (CODES & THEMES by KU).pdf. Transcript 7 (CODES & THEMES, by KU).pdf. Transcript 8 (CODES & THEMES by KU).pdf. Transcript 9 (CODES & THEMES by KU).pdf. Transcript 10 (CODES & THEMES by KU).pdf. Transcript 11 (CODES & THEMES, by KU).pdf. Transcript 12 (CODES & THEMES by KU).pdf. Transcript 13 (CODES & THEMES by KU).pdf. Transcript 14 (CODED & THEMES by KU).pdf. Transcript 15_b (CODED & THEMES by KU). pdf. Transcript 16 (CODES & THEMES by KU).pdf. Transcript 17 (CODES & THEMES by KU).pdf. Transcript 18 (CODES & THEMES by KU).pdf. Transcript 19 (CODES & THEMES by HK).pdf. Transcript 20 (CODES & THEMES by HK).pdf. Transcript 21_b (CODES & THEMES by HK).pdfTranscript 22 (CODES & THEMES by HK).pdf. Transcript 25 (CODES & THEMES by HK).pdf. Transcript 27 (CODES & THEMES by HK).pdf. Transcript Sn1 (CODES & THEMES by RS).pdf Transcript Sn6 (pt3) (CODES & THEMES by RS).pdf. Transcript Sn15_a (CODES & THEMES by RS).pdf. Transcript SN17 (pt3) (CODES & THEMES by RS).pd. Transcript Sn21_a (CODES & THEMES by RS).pdf. (ZIP) [file pgph.0004794.s001.zip › Transcript 17 (CODES & THEMES by KU).pdf]

Transcript	**Initial Codes**	**Themes**
**Transcription interview 17**<br>**Interviewee: XXX**<br>**SN-42**<br>**Interviewer: [MS], Research Assistant**<br>**Number of speakers : 2**<br>**Time: 1.51pm**<br>**Length of interview recording: 25minutes 57 seconds**<br>**Date: 11th May 2023**		
1) Consent obtained to take part in the study at the start of the zoom call, prior to interview questions being asked. Consented to video & audio recording. Participant using airtime for call, discussed refund.		
2) **Interviewer [MS]: Do you prescribe antibiotics to pregnant women?**		
3) Interviewee [XXX]: Yes I do	**2). Prescribe ATB (yes)**	
**4) Interviewer [MS]: Ahha, How long have you been prescribing them to women for?**		**[1] PRESCRIBING (1/1)**
5) Interviewee [XXX]: okay urm uhhh that should be from urm 2000 and urrr 2010 to 8 2010 to 8	**5). Prescribe ATB (years)**	
6) **Interviewer [MS]: Okay. How many times a week**		
7) Interviewee [XXX]: Hello? *overlap*		
**8) Interviewer [MS]: hi can you hear me? Hello yeah I can hear you can you hear me?**		
9) Interviewee [XXX]: *overlapping speech*		
10) **Interviewer [MS]: Okay how many times a week do you prescribe antibiotics to pregnant women?**		
11) Interviewee [XXX]: *background noise* you said what I didn't get		
**12) Interviewer [MS]: hello? *overlapping speech* How many times a week do you prescribe antibiotics to pregnant women?**		
13) Interviewee [XXX]: urm usually I don't have a fixed number it depends on it depends on the situation or the condition of the patients	**13. Prescribe ATB (frequency)**	
**14) Interviewer [MS]: mhm**		
15) Interviewee [XXX]: like for example during the antenatal clinic	**15/17. Prescribe ATB (when)**	
**16) Interviewer [MS]: mhm *overlapping speech***		
17) Interviewee [XXX]: *overlapping speech* when weve got our pregnant women		
**18) Interviewer [MS]: mhm**		
19) Interviewee [XXX]: usually most of the times we don't prescribe antibiotics we wait for to have result of culture and sensitivity	**19. Prescribe ATB (clinical circumstances)**	
**20) Interviewer [MS]: mhm**		

21) Interviewee [XXX]: but in some selected cases you know some of our women selected cases of severe infection<br>**22) Interviewer [MS]: mhm**<br>23) Interviewee [XXX]: you can usually start what we call *unclear speech* antibiotics while we wait for the result of the culture and sensitivity<br>**24) Interviewer [MS]: mhm**<br>25) Interviewee [XXX]: it's a routine prescription per say but prescribe based on the need<br>**26) Interviewer [MS]: mhm**<br>27) Interviewee [XXX]: and sometimes result of culture and sensitivity<br>28) **Interviewer [MS]: Okay. What are the 3 most common medical problems that you prescribe antibiotics for?**<br>29) Interviewee [XXX]: okay is it one of the common problems that we<br>**30) Interviewer [MS]: yeah *overlapping speech***<br>31) Interviewee [XXX]: *unclear speech*<br>**32) Interviewer [MS]: yeah**<br>33) Interviewee [XXX]: okay yeah urm sometimes we may have urm a women upper respiratory tract infection<br>**34) Interviewer [MS]: mhm**<br>35) Interviewee [XXX]: sometimes some of them maybe you know usually *unclear speech* those ones we don't prescribe antibiotics but when we have<br>**36) Interviewer [MS]: mhm *overlapping speech***<br>37) Interviewee [XXX]: *unclear speech*<br>**38) Interviewer [MS]: mhm**<br>39) Interviewee [XXX]: *unclear speech* we may want to add antibiotics while we wait for result of culture and sensitivity's<br>**40) Interviewer [MS]: mhm *overlap***<br>41) Interviewee [XXX]: sometimes you also in cases of um severe urinary tract infections<br>**42) Interviewer [MS]: mhm**<br>43) Interviewee [XXX]: you also tend to prescribe antibiotics orr these are the you know common conditions that we prescribe on outpatient basis<br>**44) Interviewer [MS]: okay**<br>45) Interviewee [XXX]: *unclear speech* usually our post op patients usually place them on prophylactic em<br>**46) Interviewer [MS]: mhm**<br>47) Interviewee [XXX]:antibiotics against infection every post op patient especially caesarean section you know urm *unclear speech* place them on antibiotics but for our obstetric patients usually post op<br>**48) Interviewer [MS]: mhm**	**21, 23, 25, 27. Prescribe ATB (clinical circumstances, requirements)**<br><br>**29, 33. Prescribe ATB (common conditions, UTI)**<br><br>**39. Prescribe ATB (clinical circumstances)**<br><br>**41. Prescribe ATB (conditions, UTI)**<br>**43, 45. Prescribe ATB (common conditions, clinical circum)**<br><br>**47. Prescribe ATB (clinical requirements)**	

49) Interviewee [XXX]: inpatient for outpatient prescribe based on selected conditions and histories	**49, 51. Prescribe ATB (clinical requirements)**	
**50) Interviewer [MS]: mhm**		
51) Interviewee [XXX]: or you know pending the result of culture and sensitivity		
**52) Interviewer [MS]: Mhm, urm do you use any guidelines when you're prescribing antibiotics?**		
53) Interviewee [XXX]: okay yes yes we have our routine we have our routine protocol what we call the labour ward protocol our obstetric protocol we have a manual	**53, 55. Prescribe ATB (guidelines, yes)**	**[6] GUIDELINES (1/3)**
**54) Interviewer [MS]: mhm**		
55) Interviewee [XXX]: *unclear speech* conditions where we prescribe antibiotics		
**56) Interviewer [MS]: okay**		
57) Interviewee [XXX]: okay let me give example for example when you have a pregnant women with a prom you know pre mature rupture of *unclear word* membranes	**57, 59, 61. Prescribe ATB (guidelines, clinical examples)**	
**58) Interviewer [MS]: mhm**		
59) Interviewee [XXX]: and she is draining liqour you may have to place on prophylactic antibiotics		
**60) Interviewer [MS]: mhm yep okay**		
61) Interviewee [XXX]: *overlapping speech* *unclear speech* some cases like preterm labour where there is obvious *unclear words* of infection we may also want to place we have a we have a labour ward protocol that guides our prescription		
**62) Interviewer [MS]: Okay great so where do you find that pregnant women generally get their antibiotics from? In the antenatal period**		
63) Interviewee [XXX]: you say?		
**64) Interviewer [MS]: where do pregnant women generally get their antibiotics from?**		
65) Interviewee [XXX]: where do they get it from?	**65, 67. Obtaining antibiotics (hospital)**	
**66) Interviewer [MS]: yeah**		
67) Interviewee [XXX]: okay well they have the hospital pharmacy like I work in the teaching hospital		
**68) Interviewer [MS]: mhm**		**[2] OBTAINING**
69) Interviewee [XXX]: so if there is need for prescription we make prescription and then the patient will get it from the hospital pharmacy here	**69. Obtaining ATB (hospital pharmacy)**	
**70) Interviewer [MS]: mhm mhm**		
71) Interviewee [XXX]:but like I said usually most of the prescriptions are based on result of microscopic culture and sensitivity however there are some selected cases where we prescribe what you call a verical antibiotics	**71, 73. Prescribe ATB (clinical requirements)**	
**72) Interviewer [MS]: mhm**		**[1] PRESCRIBING (1/2)**
73) Interviewee [XXX]: while waiting for the result of culture and sensitivity *background noise*		

74) **Interviewer [MS]: Okay, so are you aware of any pregnant women who ever take antibiotics that havent been prescribed for them?**<br>75) Interviewee [XXX]: pardon?<br>**76) Interviewer [MS]: have you ever are you aware of any pregnant women who take antibiotics that havent been prescribed for them?**		
77) Interviewee [XXX]: okay urm ah yes there are some you know some cases you know we are in Nigeria developing country we are sometimes prescription urm we don't have this restricted urm you know laws in terms of prescription. Suppose they go over the counter<br>**78) Interviewer [MS]: mhm**	**77. SM with ATB (yes, OTC, lack of regulation)**	**[3] SELF-MEDICATION**
79) Interviewee [XXX]: to you know go to the pharmacy over the counter and get some antibiotics some selected antibiotic<br>**80) Interviewer [MS]: mhm**	**79. SM with ATB) (yes, OTC)**	
81) Interviewee [XXX]: so sometimes they can *unclear word* them even without your own prescriptions<br>**82) Interviewer [MS]: mhm**	**81. SM with ATB (without prescription)**	
83) Interviewee [XXX]: so there are few of them but majority of our patient get their prescription based on doctors prescription from the hospital<br>**84) Interviewer [MS]: okay**	**83. SM with ATB (a minority)**<br>**[NOTE: CHECK AGAINST LIT]**	
85) Interviewee [XXX]: *overlapping speech* have some pockets of places that source it on their own outside<br>**86) Interviewer [MS]: mhm mhm okay so are you aware of any pregnant women who might take like herbal preparations or alternative medications that could work like antibiotics?**	**85. SM with ATB (outside sources)**	
87) Interviewee [XXX]: urm no im not aware of that we don't advocate for that we don't prescribe that no body has ever come to me with such a urm you know complain or prescription however there are some pregnant women who take herbal concoction they are few anyway<br>**88) Interviewer [MS]: mhm**	**87. Herbal SM (yes, a few patients)**	
89) Interviewee [XXX]: some of them can *unclear speech* the herbal medicine dealer, especially those in remote villages<br>**90) Interviewer [MS]: mhm mhm**	**89. Herbal SM (yes, remote villages)**	**[4] HERBAL SELF-MEDICATION**
91) Interviewee [XXX]: its quite rare its quite rare anyway but<br>**92) Interviewer [MS]: mhm**<br>93) Interviewee [XXX]: *unclear speech* but those that come to see us *unclear speech* usually they don't do that<br>**94) Interviewer [MS]: okay**<br>95) Interviewee [XXX]: *unclear speech*<br>**96) Interviewer [MS]: Okay. Urm do you know of any methods that could detect self-medication of antibiotics in pregnant women? So when they take it themselves without being prescribed**	**91, 93. Herbal SM (quite rare, especially in hospital patients)**	

97) Interviewee [XXX]: the method of detection of antibiotics		
**98) Interviewer [MS]: self medication**		
99) Interviewee [XXX]: self medication		
**100) Interviewer [MS]: yeah**		
101) Interviewee [XXX]: mmmm you can only know about that from history when you try to interact with the patient	**101, 103, 105. Detecting SM (patient histories, direct questioning)**	**[5] DETECTING**
**102) Interviewer [MS]: mhm**		
103) Interviewee [XXX]: so you can get that history outside history *unclear speech* to find out		
**104) Interviewer [MS]: okay**		
105) Interviewee [XXX]: however from the history you get a drug history of what the patient are taking		
**106) Interviewer [MS]: mhm *overlap* mhm do you think**		
107) Interviewee [XXX]: *overlapping speech* and sometimes		
**108) Interviewer [MS]: yeah**		
109) Interviewee [XXX]: and sometimes cases you know ah as a result of abuse of antibiotics there might be you know might be resistance when you do antimicrobial culture and sensitivity sometimes	**109, 111, 113. Detecting SM (from signs of ATB Resistance) {NOTE THIS POINT IN MANUSCRIPT]**	
**110) Interviewer [MS]: mhm**		
111) Interviewee [XXX]: that may be a pointer for possible abuse		
**112) Interviewer [MS]: mhm**		
113) Interviewee [XXX]: but it is not definitely *unclear speech*		
**114) Interviewer [MS]: mhm mhm. Do you think it could be useful to have a simple rapid test or tool or questionnaire that could help identify pregnant women who might misuse antibiotics without us knowing?**		
115) Interviewee [XXX]: that's that that would be very good that would be very good	**115. Detecting SM (test/tool useful)**	
**116) Interviewer [MS]: mhm**		
117) Interviewee [XXX]: very good		
**118) Interviewer [MS]: Dya have any idea of how that might work or what could work If you know there was such a tool?**		
119) Interviewee [XXX]: oh I mmm but I know from history and this *mumbled speech* some responses that may help you that guide you but ive not used any and ive not applied it yet	**119. Detecting SM (patient histories, but not sure)**	
**120) Interviewer [MS]: mhm. If such a tool was available, would you be interested in using it?**		
121) Interviewee [XXX]: why not. We are *unclear speech* I think its to be avaliable to be *unclear speech*		
**122) Interviewer [MS]: mhm mhm**		

123) Interviewee [XXX]: *mumbled unclear speech*<br>**124) Interviewer [MS]: Okay and do you think such a tool could be used within antenatal care settings, or during routine appointments, or in A&E or labour ward like where dya think it would be most useful to use such a tool?**<br>125) Interviewee [XXX]: urm I think it will be useful in all these areas you mentioned<br>**126) Interviewer [MS]: mhm**<br>127) Interviewee [XXX]: in all these areas you mentioned *mumbled unclear speech*<br>**128) Interviewer [MS]: mhm**<br>129) Interviewee [XXX]: so if you have cases that require that in antenatal clinic of course you make use of it<br>**130) Interviewer [MS]: mhm**<br>131) Interviewee [XXX]: if you have them in the labour ward you can also do that and the postnatal ward<br>**132) Interviewer [MS]: mhm**<br>133) Interviewee [XXX]: I think it will be very useful in alll those areas<br>**134) Interviewer [MS]: Do you think it would be useful for such a test to be like easy to use, remote, not needing you know electricity or internet? Dya think that would be useful?**<br>135) Interviewee [XXX]: why not yeah we *unclear speech*<br>**136) Interviewer [MS]: mhm mhm**<br>137) Interviewee [XXX]: *unclear mumbled speech* cant rely much on this<br>**138) Interviewer [MS]: mhm**<br>139) Interviewee [XXX]: and with lot of interuptions and this so if have something that try to by pass all the you know<br>**140) Interviewer [MS]: mhm**<br>141) Interviewee [XXX]:urm all those eh *unclear word* then it will be good<br>**142) Interviewer [MS]: mhm, have you come across any methods or guidelines that could help self like the side effects of antibiotic self-medication in pregnant women?**<br>143) Interviewee [XXX]: guidelines or methods<br>**144) Interviewer [MS]: yeah that kind of look at the self side effects of antibiotic self medication in women pregnant women**<br>145) Interviewee [XXX]: uh what we usually do if we have if we have a suspected case of antibiotic *unclear word* of course we take history from the history and from the presentation of the patient we can be able to say this is	**125, 127, 129, 131, 133. Detecting SM (settings)**<br><br>**142, 145, 147. Sides effects of SM (guidelines, what actually happens in practice, but NO SPECIFIC METHOD, GUIDELINE)**	**[7] SIDE EFFECTS (1/3)**<br><br>**[6] GUIDELINES (2/3)**

likely be the antibiotics the culprit but we don’t have a specific method<br>**146) Interviewer [MS]: mhm**<br>147) Interviewee [XXX]: that will help or guide you or a guideline that will help and guide you to know exactly what particular antibiotic<br>**148) Interviewer [MS]: mhm**<br>149) Interviewee [XXX]: with our knowledge of antibiotics *mumbled unclear speech*<br>**150) Interviewer [MS]: mhm *overlapping speech***<br>151) Interviewee [XXX]: *unclear speech* likely culprit you know *mumbled unclear words* antibiotics<br>**152) Interviewer [MS]: yeah, um so obviously we know that you know side effects can cause antibiotics can cause side effects and you can get side effects from antibiotics like stomach upset or not feeling well, do you think that the presence of side effects from antibiotics is clear that the patient is taking antibiotics? you know someone has side effects is it clear that its from the antibiotics?**		
153) Interviewee [XXX]: urr usually some of them may be non-specific theres other conditions that can cause same side effects that antibiotics are having<br>**154) Interviewer [MS]: mhm**<br>155) Interviewee [XXX]: so sometimes it may be difficult to draw you know a thin line between antibiotic side effect<br>**156) Interviewer [MS]: mhm**<br>157) Interviewee [XXX]: and symptoms from particular illnesses so<br>**158) Interviewer [MS]: mhm**<br>159) Interviewee [XXX]: based on history you can be able to you know um you know *unclear speech* likely due to antibiotics or other aliments<br>**160) Interviewer [MS]: mhm *overlap speech***<br>161) Interviewee [XXX]: but of course it will be difficult may be difficult to differentiate usually some of the side effects are usually non specific<br>**162) Interviewer [MS]: mhm**<br>163) Interviewee [XXX]: which other conditions can cause<br>**164) Interviewer [MS]: mhm**<br>165) Interviewee [XXX]: but *unclear speech* those conditions you know you can be able to say that this likely be due to antibiotics<br>**166) Interviewer [MS]: mhm**<br>167) Interviewee [XXX]: side effects<br>**168) Interviewer [MS]: mhm. Do you know any pregnant women who have developed side effects of antibiotic use like self-medication?**	**153, 155, 157, 159, 161, 163, 165, Side effects, as evidence of SM (side effects are non-specific, not necessarily due to ATB, difficult…, other factors, requires patient history)**	**[7] SIDE EFFECTS (2/3)**

169) Interviewee [XXX]: ummmm ummm ummumum um can I remember can I remember ummm umm yes I think ive seen some urr though not quite often ive seen some you know allergic you know rashes and *unclear speech* from ummmm trimethoprim<br>**170) Interviewer [MS]: mhm**<br>171) Interviewee [XXX]: and *another name of a medication* and all these things ive seen some yeah<br>**172) Interviewer [MS]: from self medication? *overlapping speech***<br>173) Interviewee [XXX]: self medications yes<br>**174) Interviewer [MS]: okay okay *overlapping speech*, what kind of things did you see?**<br>175) Interviewee [XXX]: you have this widespread eh skin reactions from sulphonomide so have symptom<br>**176) Interviewer [MS]: mhm okay do you know of any like methods or guidelines or protocols that manage antibiotic self medication in pregnant women?**<br>177) Interviewee [XXX]: protocol<br>**178) Interviewer [MS]: mhm**<br>179) Interviewee [XXX]: there are some guidelines but im not able to remember the particular *mumbled speech*. I know of some where you can be able to know have such *laughing in background* side effect what to do<br>**180) Interviewer [MS]: mhm mhm *overlapping speech***<br>181) Interviewee [XXX]: how to manage yes there are I cant remember *mumbled speech* for now<br>**182) Interviewer [MS]: okay**<br>183) Interviewee [XXX]: *overlapping unclear speech*<br>**184) Interviewer [MS]: okay yeah or even I mean like are there any guidelines that are written specifically about antibiotic self medication in pregnant women not just the side effects just you know is there a guideline that just looks at antibiotic self medication in pregnant women**<br>185) Interviewee [XXX]: *mumbled speech*<br>**186) Interviewer [MS]: sorry?**<br>187) Interviewee [XXX]: yeah yeah I said of course if whenever there is you know allergic reaction or any side effect usually we go to look at that and see how we can manage the patient<br>**188) Interviewer [MS]: mhm**<br>189) Interviewee [XXX]: we have that<br>**190) Interviewer [MS]: okay okay so you have a guideline about side effects so is that how you would kind of manage or have a method at managing antibiotic self medication mainly through if the woman had side effects**	**169, 171. Side effects fr. SM (possibly, not certain**<br><br>**173, 175. Side effects fr. SM (yes, skin reactions)**<br><br>**177, 179, 181. SM guidelines (possibly, can't remember)**<br><br>**187, 189. SM guidelines (yes, will investigate allergic reactions).**<br><br>**191, 193, 195. SM guidelines (none in hospital, use**	[6] GUIDELINES (3/3)

191) Interviewee [XXX]: yeah what im saying is that we don't have a protocol in the hospital<br>**192) Interviewer [MS]: mhm**<br>193) Interviewee [XXX]: but of course we make use of other guidelines<br>**194) Interviewer [MS]: mhm mhm**<br>195) Interviewee [XXX]: to guide us on managing but we don't have hospital protocol per say or guideline<br>196) **Interviewer [MS]: okay okay that's great thank you. So this is my last question dya feel like you need a break or anything? Or are you okay to carry on, ive just got one more question.**<br>197) Interviewee [XXX]: okay you have one more<br>**198) Interviewer [MS]: is that okay? *overlapping speech* yeah is that okay sorry**<br>199) Interviewee [XXX]: *overlapping speech*<br>**200) Interviewer [MS]: I know you said you've not got a lot of time either so I wanted to try and urm get things done for you you know like to have the interview with you so yeah**<br>201) Interviewee [XXX]:*overlapping unclear speech*<br>**202) Interviewer [MS]: yeah no thank you. Umm so theres a specific area with pregnant women who have self medicated with antibiotics and they develop like signs of memory loss, or forgetfullness, do you know any management options if that happened?**<br>203) Interviewee [XXX]: of memory loss?<br>**204) Interviewer [MS]: mhm or forgetfullness**<br>205) Interviewee [XXX]: mmmm *no reply for a few seconds* not really not really<br>**206) Interviewer [MS]: okay okay that's fine that's its just really think of any management plans or options of what you might do if someone had memory loss or forgetfullness from self medicating with antibiotics so its just kinda to have a discussion if you did have any insight**<br>207) Interviewee [XXX]:yes ive not had a patient not had a case *mumbled speech* so<br>**208) Interviewer [MS]: okay that's that's fine urm thank you so that's all my questions**<br><br>**209) End of interview questions. Participant asked if they had any questions, and they said no. Thanked for taking part in the interview, advised can contact us if they have any questions. Advised consent form completed will be returned to them. Mentioned airtime card again.**	**other guidelines) [NOTE: RESPONSES IMPLY SOME CONFUSION RE. GUIDELINES]**<br><br>**203, 205, 207. Side effects (neurological, no, not seen such a patient).**	**[7] SIDE EFFECTS (3/3)**
