# Supplementary material for: Advancing the safe motherhood initiative: A qualitative and sentiment analysis of local physician’s perspectives on antibiotic self-medication during pregnancy in a low- and middle-income country
Source: PLOS Glob Public Health. 2025 Sep 12;5(9):e0004794. doi: 10.1371/journal.pgph.0004794 (PMC12431270; doi:10.1371/journal.pgph.0004794)
Supplement: S1 File — Transcript 4 (CODES & THEMES by KU).pdf. Transcript 6 (CODES & THEMES by KU).pdf. Transcript 7 (CODES & THEMES, by KU).pdf. Transcript 8 (CODES & THEMES by KU).pdf. Transcript 9 (CODES & THEMES by KU).pdf. Transcript 10 (CODES & THEMES by KU).pdf. Transcript 11 (CODES & THEMES, by KU).pdf. Transcript 12 (CODES & THEMES by KU).pdf. Transcript 13 (CODES & THEMES by KU).pdf. Transcript 14 (CODED & THEMES by KU).pdf. Transcript 15_b (CODED & THEMES by KU). pdf. Transcript 16 (CODES & THEMES by KU).pdf. Transcript 17 (CODES & THEMES by KU).pdf. Transcript 18 (CODES & THEMES by KU).pdf. Transcript 19 (CODES & THEMES by HK).pdf. Transcript 20 (CODES & THEMES by HK).pdf. Transcript 21_b (CODES & THEMES by HK).pdfTranscript 22 (CODES & THEMES by HK).pdf. Transcript 25 (CODES & THEMES by HK).pdf. Transcript 27 (CODES & THEMES by HK).pdf. Transcript Sn1 (CODES & THEMES by RS).pdf Transcript Sn6 (pt3) (CODES & THEMES by RS).pdf. Transcript Sn15_a (CODES & THEMES by RS).pdf. Transcript SN17 (pt3) (CODES & THEMES by RS).pd. Transcript Sn21_a (CODES & THEMES by RS).pdf. (ZIP) [file pgph.0004794.s001.zip › Transcript 18 (CODES & THEMES by KU).pdf]

Transcript	Initial Codes	Themes
**Transcription interview 18**<br>**Interviewee: XXX**<br>**SN-29**<br>**Interviewer: (MS), Research Assistant**<br>**Other attendees: [RS] Student Volunteer**<br>**Number of people on call: 3**<br>**Time: 3:51pm**<br>**Length of interview recording: 34 minutes 19 seconds**<br>**Date: 26th May 2023**<br><br>Student volunteer for project also attended the call, participant happy for this.<br><br>Note participant advised had forgot to read participant information sheet or consent form that was sent to him. Asked if wanted to read prior to interview, but participant wanted to start the interview due to limited time. Signal problems at the start of the call. Consent obtained prior to commencing interview questions, via zoom. Participant was using airtime for call, discussed.		
1) **Interviewer [MS]: So do you prescribe antibiotics to pregnant women?**		
2) Interviewee [XXX]: yes if they need when they need It I do	**2.Prescribing ATB**	
**3) Interviewer [MS]: okay urm how long have you been prescribing antibiotics to pregnant women for?**		**[1] PRESCRIBNG ATB**
4) Interviewee [XXX]: mmmm throughout my practice I practice for many years and within the practice I have been prescribing antibiotics to those that need it	**4.Prescribing ATB (duration)**	
5) **Interviewer [MS]: okay how many years have you been qualified for?**		
6) Interviewee [XXX]: mmmm more than 20 *slightly unclear* years now	**6/7. Prescribing (years)**	
7) **Interviewer [MS]: fine, so you've been prescribing antibiotics since you qualified as a doctor?**		
8) Interviewee [XXX]: yes		
**9) Interviewer [MS]: so now how many times a week do you prescribe antibiotics.. to pregnant women?**		
10) Interviewee [XXX]: I didn't get that		
**11) Interviewer [MS]: How many times a week do you prescribe antibiotics to pregnant women?**		
12) Interviewee [XXX]: Is it how many times		
**13) Interviewer [MS]: yeah a week yeah**	**12/14. Prescribing frequency**	

14) Interviewee [XXX]: I do it weekly during my clinic there is almost be one or two people that may need antibiotics	16. Prescribing frequency	
**15) Interviewer [MS]: mhm mhm okay**		
16) Interviewee [XXX]: *overlapping speech* soo lets just say my my antenatal days once a week and during the antenatal they prescribe for people that need it that's never been a week I did not prescribe antibiotics		
**17) Interviewer [MS]: okay okay so a lot**		
18) Interviewee [XXX]: yeah	20, 22. Prescribing (illnesses, diseases)	
**19) Interviewer [MS]: urm what are the 3 most common medical problems that you prescribe antibiotics for?**		
20) Interviewee [XXX]: okay um maybe respiratory tract infections what we do is put in an mcs and we see if we can for culture organism normally I prescribe sensitive antibiotics some that also come with maybe genital tract infections er maybe after a wahh mcs also prescribe for them that's the two major areas where we prescribe antibiotics we also prescribe for those that have premature rupture of membrane		
**21) Interviewer [MS]: mhm**		
22) Interviewee [XXX]: especially if its more than 12 hours after the rupture of membrane	24. Prescribing (illnesses, diseases)	
**23) Interviewer [MS]: mhm**		
24) Interviewee [XXX]: we also prescribe antibiotics but the major area is those who have these upper respiratory tract infections and genital tract infections then once in a while the premature membranes of also prescribe antibiotics for them		
**25) Interviewer [MS]: okay so do you use any guidelines when you prescribe antibiotics?**	28. Guidelines	[6] GUIDELINES_ 1/3
26) Interviewee [XXX]: Do I use any?		
**27) Interviewer [MS]: Guidelines**	30. Guidelines (none)	
28) Interviewee [XXX]: Guideline		
**29) Interviewer [MS]: yeah**		
30) Interviewee [XXX]: mmmm we don't really have any specific guideline what guide us *unclear speech* sensitivity test the culture the sensitivity test that's what guides us but in some *signal disruption*		
31) *signal disruption unclear speech*		
**32) Interviewer [MS]: sorry the signal cut out**		
33) Interviewee [XXX]: *overlap speech*		
**34) Interviewer [MS]: I don't know if it might be better signal if you turn off your video it might be better, signal**		
35) Interviewee [XXX]: Hello?		
**36) Interviewer [MS]: hi can you hear me?**		
37) Interviewee [XXX]: I didn't get you it *unclear speech*		

**38) Interviewer [MS]: it might the signal might be better if you turn off your video it might work better**<br>39) Interviewee [XXX]: okay<br>**40) Interviewer [MS]: It might give you more signal**<br>41) Interviewee [XXX]: *no speech*<br>**42) Interviewer [MS]: or improve the quality of the call**<br>43) Interviewee [XXX]: okay<br>**44) Interviewer [MS]: you can try if not it might sometimes the video makes it**<br>45) Interviewee [XXX]: *overlap speech* turn off the video<br>**46) Interviewer [MS]: yeah we'll we will try and then it might make the call better**<br>47) Interviewee [XXX]: okay<br>**48) Interviewer [MS]: right should we try**<br>49) Interviewee [XXX]: *overlapping speech* is it okay now?<br>**50) Interviewer [MS]: I can hear you, if you're happy to do it this way its fine but sometimes the video when your signals not good makes it harder so if you turn off the video it might make the signal better**<br>51) Interviewee [XXX]: mm its alright lets try it and see		
**52) Interviewer [MS]: okay so you were saying that you don't it cut off so you were saying you don't really have guidelines?**	**53. Guidelines (none)**	
53) Interviewee [XXX]: yes I just say we don't actually have guideline we depend on the sensitivity test<br>**54) Interviewer [MS]: mmhm mhm**<br>55) Interviewee [XXX]: the ahh the culture and sensitivity test that's what will base our decisions but in some cases we use we just go ahead and use broad spectrum antibiotics<br>**56) Interviewer [MS]: mhm**	**55. Guidelines (none, so prescribe based on cultural and clinical assessments)**	
57) Interviewee [XXX]: especially when we are waiting for the sensitivity test<br>**58) Interviewer [MS]: mhm mhm**<br>59) Interviewee [XXX]: but there no specific guideline that is available to us<br>**60) Interviewer [MS]: Okay okay so where do pregnant women generally get their antibiotics from?**	**59. Guidelines (none)**	
61) Interviewee [XXX]: mm I didn't get that<br>**62) Interviewer [MS]: where do find that pregnant women generally get their antibiotics from?**<br>63) Interviewee [XXX]: that's where will source the antibiotics	**63. Obtaining ATB**	**[2] OBTAINING_1**
**64) Interviewer [MS]: mhm yeah where do the women get them from**<br>65) Interviewee [XXX]: okay we give them in our hospital<br>**66) Interviewer [MS]: mhm**	**65. Obtaining ATB (from hospital)**<br>**67. Obtaining ATB (from hospital pharmacy)**	

67) Interviewee [XXX]: will normally get from the drug companies store in our hospital pharmacy so they source it from the hospital pharmacy<br>**68) Interviewer [MS]: okay do they ever get it from anywhere else?**<br>69) Interviewee [XXX]: mmm?<br>**70) Interviewer [MS]: do the women ever get antibiotics from anywhere else?**<br>71) Interviewee [XXX]: ahh sometimes they also go out and buy from those pharmacies selling drugs around but most of the time we give them from the hospital pharmacy store<br>72) **Interviewer [MS]: mhm okay do you know of any pregnant women that sometimes take antibiotics that havent been prescribed for them?**<br>73) Interviewee [XXX]: any?<br>74) **Interviewer [MS]: do you know have you ever seen when a women has taken antibiotics that havent been prescribed for them?**<br>75) Interviewee [XXX]: of course a lot of them do that<br>**76) Interviewer [MS]: okay *overlapping speech***<br>77) Interviewee [XXX]: they come to hospital they'll come the hospital and say they have taken certain antibiotics but they are still having the symptoms<br>**78) Interviewer [MS]: mhm**<br>79) Interviewee [XXX]: so they normally tell us Im having certain symptoms and ive taken antibiotics and they will mentioned ones have taken but that the symptom is still there<br>**80) Interviewer [MS]: okay**<br>81) Interviewee [XXX]: and that's why they now came to hospital yeah<br>**82) Interviewer [MS]: okay and where have they where do they get those antibiotics from if they havent been prescribed?**<br>83) Interviewee [XXX]: they get it from the pharmacists or and chemists around along the road those *unclear speech* stores along the road<br>**84) Interviewer [MS]: okay and they don't need a prescription for that?**<br>85) Interviewee [XXX]: mm?<br>**86) Interviewer [MS]: they don't need a prescription for that?**<br>87) Interviewee [XXX]: mmmm actually by law they should they should dispense it<br>**88) Interviewer [MS]: mhm**<br>89) Interviewee [XXX]: *signal disruption again unclear speech*	**71. Obtaining ATB (other sources)**<br><br>**75. SM (very common)**<br><br>**77. SM (confirmed by patients themselves)**<br><br>**79. SM (confirmed by patients themselves, symptoms)**<br><br>**83. Obtaining ATB (from pharmacists, in community)**<br><br>**87. Obtaining ATB (legal perspective)**	**[3] SELF-MEDICATION**<br><br>**[2] OBTAINING_2**

**90) Interviewer [MS]: hello?**<br>91) Interviewee [XXX]: hello sorry about the interuption<br>**92) Interviewer [MS]: okay its okay *overlapping speech***<br>93) Interviewee [XXX]: I was going to say that by law by law<br>**94) Interviewer [MS]: mhm *overlapping speech***<br>95) Interviewee [XXX]: the antibiotics should be sold on prescription but most of those *unclear speech* the pharmacists they don't obey the law they just go there and they'll give them antibiotics and make their money<br>**96) Interviewer [MS]: okay**<br>97) Interviewee [XXX]: actually not the law<br>**98) Interviewer [MS]: okay okay urm okay so are you aware of any pregnant women who might take like herbal preparations or alternative medications that could work like antibiotics?**<br>99) Interviewee [XXX]: hello?<br>**100) Interviewer [MS]: hi can you hear**<br>101) Interviewee [XXX]: I didn't get that I didn't get that<br>**102) Interviewer [MS]: Have you ever known any pregnant women to take like herbal preparations or like alternative medications that might work in the same way as antibiotics?**<br>103) Interviewee [XXX]: any pregnant women taking medication like antibiotics<br>**104) Interviewer [MS]: *overlapping speech* like dya know any pregnant women take like herbal preparations so like herbs or leaves or soil**<br>105) Interviewee [XXX]: *overlapping speech* okay okay okay mmmm no ive not im not come across those taking herbal preparations<br>**106) Interviewer [MS]: mhm**<br>107) Interviewee [XXX]: pregnant women the ones I know they are not pregnant but im aware that some of them take that but ive not come across it my person have not mentioned to me mhm<br>**108) Interviewer [MS]: okay okay**<br>109) Interviewee [XXX]: the herbal preparation they take is not really for antibiotic ive seen the ones taking herbal preparation maybe to make their child not to be big things like that<br>**110) Interviewer [MS]: Okay *overlapping speech***<br>111) Interviewee [XXX]: That's one they do commonly they will tell you that taking so and so so that the child will not be big<br>**112) Interviewer [MS]: mmm okay**	**95. Obtaining ATB (legal perspective)**<br><br>**105. Herbal SM (not aware of)**<br><br>**107. Herbal SM (happens, but had no personal experience of it)**<br><br>**109. Herbal SM (not necessarily ATB)**<br><br>**109/111. Herbal SM (motivation - baby)**<br><br>**113. Herbal SM (motivation - delivery)**	**[4] HERBAL SELF-MEDICATION**

113) Interviewee [XXX]: *overlapping speech* another *unclear speech* vaginal delivery<br>**114) Interviewer [MS]: mhm fine fine so there not doing it they don't take it necessarily for antibiotics but they take it for other reasons in the pregnancy?**<br>115) Interviewee [XXX]: *mumbled*<br>**116) Interviewer [MS]: okay**<br>117) Interviewee [XXX]: hello?<br>**118) Interviewer [MS]: Hi could you hear me?**<br>119) Interviewee [XXX]: no I didn't hear you sorry<br>**120) Interviewer [MS]: okay so im saying so they'll take it they don't take them herbal preparations for antibiotics they take it for other reasons in the pregnancy sometimes?**<br>121) Interviewee [XXX]: *muffled noise in background*<br>**122) Interviewer [MS]: hello?**<br>123) Interviewee [XXX]: you talked about ee hello?<br>**124) Interviewer [MS]: hi its okay I was just clarifying what you were saying**<br>125) Interviewee [XXX]: im sorry you have to really have to have to have patience with me because the calls or something not really *unclear word ?clear*<br>**126) Interviewer [MS]: its okay no I appreciate you taking the time so eh its fine its completely fine urm**<br>127) Interviewee [XXX]: *overlapping speech* so so im very sorry you may need to repeat *unclear speech* before I get it because its not all that clear<br>**128) Interviewer [MS]: that's fine**<br>129) Interviewee [XXX]: mm *overlapping*<br>**130) Interviewer [MS]: completely mm fine do you know of any methods that detect self-medication of antibiotics in pregnant women? So how would you know that someone was self medicating with antibiotics?**<br>131) Interviewee [XXX]: *background noise* are you talking about the adverse effect of pregnant women<br>**132) Interviewer [MS]: no no im just asking you know how would you know if you had a woman in your clinic that was self medicating with antibiotics in the pregnancy, how would you know that she was?**<br>133) Interviewee [XXX]: that how I know that shes taking antibiotics something in pregnancy<br>**134) Interviewer [MS]: mhm**<br>135) Interviewee [XXX]: is that question<br>**136) Interviewer [MS]: yeah**	**131. Detecting SM (patient history, patient feedback)**	**[[5] DETECTING_1**

137) Interviewee [XXX]: okay eh from what I said earlier, it from history normally when they come to the hospital they tell you what they been taking so those taking antibiotics will usually tell us ive taken certain antibiotics but Im still having my symptoms so we hear from them<br>**138) Interviewer [MS]: mhm okay dya think**<br>139) Interviewee [XXX]: mm *overlapping speech*<br><br>**140) Interviewer [MS]: it could be useful to have like a simple rapid test or a tool or questionnaire that could help identify pregnant women who might be misusing antibiotics?**<br>141) Interviewee [XXX]: *background noise* mm mm<br>**142) Interviewer [MS]: dya think it could be useful to have a test or a questionnaire or a tool that would help identify pregnant women who might be misusing antibiotics?**<br>143) Interviewee [XXX]: test that will help the misuse of antibiotics<br>**144) Interviewer [MS]: yeah dya think**<br>145) Interviewee [XXX]: *overlapping speech* yeah trying to get the question is it you mean things that can help the misuse of antibiotics<br>**146) Interviewer [MS]: so im asking do you think it would be helpful to have like a simple test or a tool or a questionnaire that might help you know find pregnant women who are taking antibiotics without a prescription**<br>147) Interviewee [XXX]: ee okay ee I don't think it will be necessary<br>**148) Interviewer [MS]: mhm**<br>149) Interviewee [XXX]: because I learn from many time of practice we always know when to give antibiotics and those that will need It so I don't think we will need any like questionaires<br>**150) Interviewer [MS]: okay so its not really about people who that need it its more about you know you were saying that sometimes its women who are taking them without a prescription like they may have got it from a pharmacy or you know without seeing a doctor. Do you think it might be useful to have a test or a questionnaire or some kind of tool that would identify these people? You know not just from history taking might be from a test or a tool or a more complex questionnaire dya think it would be helpful to have that?**	**143. Detecting SM (tool needed)**<br><br>**147. Detecting SM (tool NOT needed [but see 143])**<br><br>**149. Detecting SM (using clinical experience)**<br><br>**151. Detecting SM (tool might be useful [but see 147 & 143})**<br>**153. Detecting SM (tool efficacy**	**[5] DETECTING_2**

151) Interviewee [XXX]: eh wa I think it may althought I cannot really say about it may actually be helpful<br>**152) Interviewer [MS]: *overlapping speech***<br>153) Interviewee [XXX]: its only when you put it in use you can *unclear speech* whether its actually helpful in my opinion it may be<br>**154) Interviewer [MS]: *overlapping speech***<br>155) Interviewee [XXX]: but if you have such a *unclear speech* you can now definitely see whether its helpful or not.<br>**156) Interviewer [MS]: okay**<br>157) Interviewee [XXX]: but *unclear speech* actually be helpful<br>**158) Interviewer [MS]: Okay would you be interested in using such a tool, if it was available?**<br>159) Interviewee [XXX]: if its available why not you can use it atleast know how helpful it is<br>**160) Interviewer [MS]: Okay so if there was such a tool would dya think it could be used in antenatal care settings, or routine appointments, or in A&E? Like where do you think it would be best used?**<br>161) Interviewee [XXX]: hello?<br>**162) Interviewer [MS]: Hi did you hear me?**<br>163) Interviewee [XXX]: no I didn't get this<br>164) **Interviewer [MS]: okay so if there was a tool that you could use, urm dya think it would be best within you know antenatal care settings, or routine appointments, or in A&E like where dya think it would be best used?**<br>165) Interviewee [XXX]: sorry I<br>**166) Interviewer [MS]: did you hear me or no?**<br>**167)** Interviewee [XXX]: still don't get I don't know you my network is bad I still don't get you sorry about that **Interviewer [MS]: okay fine**<br>168) Interviewee [XXX]: *overlapping speech* I wish *unclear speech*<br>**169) Interviewer [MS]: its okay. So if there was a tool like a questionnaire or a tool or something where dya think it would best be used in the hospital?**<br>170) Interviewee [XXX]: okay if theres gno be a questionnaire<br>**171) Interviewer [MS]: yeah**<br>172) Interviewee [XXX]: you know we'll u'll use it in the hospital<br>**173) Interviewer [MS]: yeah where in the hospital dya think it could be used**	**unclear, needs to be tried out first)**<br>**155. Detecting SM (tool needs to be tried out first)**<br>**159. Detecting SM (tool: will use if available)**<br>**172. Detecting SM (setting.., hospital)**<br>**174. Detecting SM (setting.., hospital)**<br>**178. Detecting SM (tool: will use if available)**	

174) Interviewee [XXX]: if we have such we'll use it in the hospital<br>**175) Interviewer [MS]: mhm**<br>176) Interviewee [XXX]: such is avaliable say we don't have such in our area<br>**177) Interviewer [MS]: mhm**<br>178) Interviewee [XXX]: if its avaliable we will actually definitely use it<br>**179) Interviewer [MS]: what departments dya think it would be most useful in?**<br>180) Interviewee [XXX]:*unclear words*<br>**181) Interviewer [MS]: what departments what areas dya think it could be useful you know like labour ward or in routine appointments or A&E**<br>182) Interviewee [XXX]: *high pitched words* I think it will be in areas it will be useful maybe in such area research area selection maybe selection of the right antibiotics to use as *unclear speech* be useful ummmm woah woah woah I think it will only be useful if selection of the right antibiotics for the right maybe for the right symptoms<br>**183) Interviewer [MS]: okay okay so just say if we had a test tor a tool that was looking at antibiotic its all about identifying um antibiotic misuse in pregnancy so if someone has got antibiotics without a prescription without seeing a doctor or their not the right antibiotics if we had a tool to test that dya think it would need to be easy to use remote would we need to use electricity for it?**<br>**184) *background noise***<br>**185) Interviewer [MS]: hello?**<br>186) Interviewee [XXX]: hello?<br>**187) Interviewer [MS]: did you hear me?**<br>188) Interviewee [XXX]: no<br>**189) Interviewer [MS]: okay so ill try again so if you had if we had such a test urm**<br>190) Interviewee [XXX]: *overlapping unclear speech*<br>**191) Interviewer [MS]: a test or a tool**<br>192) Interviewee [XXX]: okay<br>**193) Interviewer [MS]: a tool to identify antibiotic misuse in pregnancy do you think it should be like easy to use or remote or use without internet how dya think it would best be used?**<br>194) Interviewee [XXX]: okay lemme see whether I got the question you said if you have maybe a tag or how t select antibiotics in pregnancy that what youre saying?	**182. Detecting SM (setting.., research context)**	

195) Interviewer [MS]: no so its not the whole the whole concept isnt about finding the right antibiotic in pregnancy

196) Interviewee [XXX]: yeah *overlapping speech*

197) Interviewer [MS]: *unclear speech* is about

198) Interviewee [XXX]: *overlapping speech*

199) Interviewer [MS]: if you've got if you've got a pregnant woman that comes and she is misusing antibiotics so shes using antibiotics that havent been prescribed for her um its about detecting that so if you wanted to detect that and you're using a test or a tool or a questionnaire that was developed dya think that would need to be easy to use like with electricity or internet or not like you know that kind of thing

200) Interviewee [XXX]: *background noise breathing* *laughing*

201) Interviewer [MS]: is the connection not good enough?

202) Interviewee [XXX]: *background noise*

203) Interviewer [MS]: can you hear me?

204) Interviewee [XXX]:mmmm just hearing half of what youre saying

205) Interviewer [MS]: okay

206) Interviewee [XXX]: *overlapping speech* *unclear speech* I will read it and get you

207) Interviewer [MS]: so if the signals not good enough then and you don't understand the questions we cant really continue with the interview cuse obviously you don't you're not you know able to hear the whole question urm are you able to go somewhere where theres wifi or at home dya have wifi or dya have better signal at home and we can try and do it next week?

208) Interviewee [XXX]: its actually difficult to get connected to wifi normally I chat with me *unclear word* *unclear speech* with my phone

209) Interviewer [MS]: yeah

210) Interviewee [XXX]: you know sometimes we don't have wifi in my area

211) Interviewer [MS]: mhm

212) Interviewee [XXX]: so what I do is use the data and change and do *unclear speech* maybe that's why the *unclear word* stuff is here sometimes but that's as much we can get that is what is available to me, so *unclear words* what is avaliable. Wifi not readily avaliable in my area

213) Interviewer [MS]: okay so where is there somewhere where theres better signal can you go

anywhere we can try I normally do the interviews on thursdays urm but if you want we can try and continue with other questions if the signal gets better but if the signals not good and you're not understanding the questions then its hard to do it *unclear speech*

214) Interviewee [XXX]: its okay if possible to write down the question then I will be I can *unclear word* answer them that one is it possible at all *unclear speech* can we finish then in something written

215) Interviewer [MS]: so weve not done that before urm I can just ask my manager and see if we can do that or we can skip that question and know and try and do the other questions and see if the signals better or is it all the time that my speech is cutting out?

216) Interviewee [XXX]: mmm its okay

217) Interviewer [MS]: are you understanding the full sentence of what im saying or is it cutting up?

218) Interviewee [XXX]: what im saying is that if I can have the questionnaire it wil be easy answered

219) Interviewer [MS]: yes yes the thing is there not yeah ill it's a talking interview were not normally you know writing the questions down it's a discussion so ill I can ask my manager if we can do that and if we can do that and if we can look at doing that next week if that's a possibility

220) Interviewee [XXX]: its alright

221) Interviewer [MS]: is that okay

222) Interviewee [XXX]: that's no problem

223) Interviewer [MS]: dya want to continue with the rest of the questions or is the signal not good enough are you not understanding the questions

224) Interviewee [XXX]: its not that im understanding the question but sometimes I don't hear it very well maybe because of poor network and stuff and that's why I said the questionnaire will be better and be answering

225) Interviewer [MS]: mhm mhm okay okay

226) Interviewee [XXX]: so if you can mmm arrange and maybe give me a questionnaire it will be better

227) Interviewer [MS]: mmm mmm okay ill speak to my manager

228) Interviewee [XXX]: *overlapping speech* maybe next one will be *unclear speech*

229) Interviewer [MS]: okay that's okay I will speak to him and see what kind of the options are cuse with this it is an interview you know were

**talking urm would you like me to ask you some of the other questions and see if you can answer them if the signals okay or dya want to stop now**<br>230) Interviewee [XXX]: well you can ask my problem is whether it will be clear or not the network be clear enough for me to hear them lets give it a try *unclear speech*<br>**231) Interviewer [MS]: okay okay**<br>232) Interviewee [XXX]: *overlapping speech*<br>**233) Interviewer [MS]: *unclear speech* abit complicated urm. Have you ever come across any guidelines which can help detect side effects of antibiotic self-medication in pregnant women?**<br>234) Interviewee [XXX]: no<br>**235) Interviewer [MS]: okay**<br>236) Interviewee [XXX]: *unclear speech* guidelines<br>**237) Interviewer [MS]: Okay okay, so we know antibiotics can cause side effects urm do you think side effects in a patient is clear evidence that their taking antibiotics?**<br>238) Interviewee [XXX]: mmmm ive not seen any of the side effect in my own patients ive not seen any side effects my patient<br>239) **Interviewer [MS]: mhm okay. When they've taken antibiotics?**<br>240) Interviewee [XXX]: mmm<br>**241) Interviewer [MS]: when they've taken antibiotics you've never seen side effects when someone has taken antibiotics**<br>242) Interviewee [XXX]: yes ive not witnessed any side effects so far<br>**243) Interviewer [MS]: okay that's fine. Do you know any pregnant women who have had side effects when they've self medicated with antibiotics?**<br>244) Interviewee [XXX]: no<br>**245) Interviewer [MS]: *unclear speech***<br>246) Interviewee [XXX]: none<br>247) **Interviewer [MS]: okay okay Are there any guidelines to manage antibiotic self medication in pregnant women?**<br>248) Interviewee [XXX]: we don't have such guideline<br>**249) Interviewer [MS]: okay okay so this is a longer question. So sometimes pregnant women that self medicate with antibiotics can develop signs of memory loss or forgetfullness is there any management plans when that happens?what would you do if that was a side effect that you saw?**	**234. Guidelines on SM side effects (no)**<br><br>**238. Side effects fr. SM (not seen any)**<br><br>**242, 244. Side effects (not witnessed any)**<br><br>**248. GUIDELINES on SM (none)**<br><br>**250. Guidelines (no management plan, use own judgement)**<br><br>**252. Guidelines (none, use own judgement)**	**[6] GUIDELINES_ 2/3**<br><br>**[7] SIDE EFFECTS**<br><br>**[6] GUIDELINES_ 3/3**

250) Interviewee [XXX]: ehh well theres no management theres no standard management plan but when we see such we use our own clinical *unclear word* and make decisions<br>**251) Interviewer [MS]: mhm mhm**<br>252) Interviewee [XXX]: sometimes you do symptomatic management some of these days but depends on you as a physician to make *unclear word* but theres no standard or generalised guideline that is avaliable so we do on our own on their own *unclear speech* based on symptom *unclear word*<br>**253) Interviewer [MS]: okay amazing so that's all my questions thank you**<br><br>**254) Participant had no further questions. End of interview. Advised we could only do interviews on zoom not whatsapp due to ability to record. Advised did answer most of the questions, but would speak to manager. Signal issues noted during call.**		
