# Supplementary material for: Advancing the safe motherhood initiative: A qualitative and sentiment analysis of local physician’s perspectives on antibiotic self-medication during pregnancy in a low- and middle-income country
Source: PLOS Glob Public Health. 2025 Sep 12;5(9):e0004794. doi: 10.1371/journal.pgph.0004794 (PMC12431270; doi:10.1371/journal.pgph.0004794)
Supplement: S1 File — Transcript 4 (CODES & THEMES by KU).pdf. Transcript 6 (CODES & THEMES by KU).pdf. Transcript 7 (CODES & THEMES, by KU).pdf. Transcript 8 (CODES & THEMES by KU).pdf. Transcript 9 (CODES & THEMES by KU).pdf. Transcript 10 (CODES & THEMES by KU).pdf. Transcript 11 (CODES & THEMES, by KU).pdf. Transcript 12 (CODES & THEMES by KU).pdf. Transcript 13 (CODES & THEMES by KU).pdf. Transcript 14 (CODED & THEMES by KU).pdf. Transcript 15_b (CODED & THEMES by KU). pdf. Transcript 16 (CODES & THEMES by KU).pdf. Transcript 17 (CODES & THEMES by KU).pdf. Transcript 18 (CODES & THEMES by KU).pdf. Transcript 19 (CODES & THEMES by HK).pdf. Transcript 20 (CODES & THEMES by HK).pdf. Transcript 21_b (CODES & THEMES by HK).pdfTranscript 22 (CODES & THEMES by HK).pdf. Transcript 25 (CODES & THEMES by HK).pdf. Transcript 27 (CODES & THEMES by HK).pdf. Transcript Sn1 (CODES & THEMES by RS).pdf Transcript Sn6 (pt3) (CODES & THEMES by RS).pdf. Transcript Sn15_a (CODES & THEMES by RS).pdf. Transcript SN17 (pt3) (CODES & THEMES by RS).pd. Transcript Sn21_a (CODES & THEMES by RS).pdf. (ZIP) [file pgph.0004794.s001.zip › Transcript 19 (CODES & THEMES by HK).pdf]

Transcript	Initial Codes	Themes
**Transcription interview 19**<br>**Interviewee: XXX**<br>**SN- 35**<br>**Interviewer: (MS), Research Assistant**<br>**Number of speakers :3**<br>**Other Attendees: (RS) Research Volunteer/Student**<br>**Time: 16:06 UK time**<br>**Length of interview recording: 18 minutes 15 seconds**<br>**Date: 15th June 2023**<br><br>1) Participant wanted to read Participant Information Sheet after the interview, Interviewer gave brief description of study. Participant didn't have any questions and was okay with volunteer (RS) being present. Consent obtained on zoom call prior to interview questions proceeding, including consenting to recording. Participant using airtime, discussed reimbursement process.		
**2) Interviewer [MS]: So do you prescribe antibiotics to pregnant women?**<br>3) Interviewee [XXX]: yes but not very often<br>**4) Interviewer [MS]: Okay, how long have you been a prescriber for?**<br>5) Interviewee [XXX]: I I prescribe only when its needed<br>**6) Interviewer [MS]: okay how long have you been prescribing antibiotics to women for?**<br>7) Interviewee [XXX]: Ive urm since since eh like like 10 years now<br>**8) Interviewer [MS]: Okay okay. How many times a week do you think you prescribe them to pregnant women?**<br>9) Interviewee [XXX]: maybe maybe once in a week	**3-5) Gives a pre-assumed answer to highlight he/she has not prescriped more antibiotics and gives a wrong answer to justify previous question**	.<br>**[1] PRESCRIBING (Professional Ethics)**
**10) Interviewer [MS]: okay okay and urmm what are the 3 most common medical problems that you prescribe antibiotics for?**<br>11) Interviewee [XXX]: um like sepsis<br>**12) Interviewer [MS]: mhm**<br>13) Interviewee [XXX]: sometimes for a as a prophylaxis	**11 & 13) He/She is experienced**	
**14) Interviewer [MS]: mhm okay anything else?**<br>15) Interviewee [XXX]: nooo<br>**16) Interviewer [MS]: Okay. Do you use any guidelines when prescribing you're prescribing antibiotics?**<br>17) Interviewee [XXX]: Yes I not really<br>**18) Interviewer [MS]: N Dya not have any guidelines that you use?**	**17) Seems he/she prescribes based on his/her experience so,**	**[6] GUIDELINES**

19) Interviewee [XXX]: well I use eh only guidelines *unclear word* guidelines<br>20) *overlapping speech unclear*<br>21) Interviewee [XXX]: *unclear speech* in pregnancy<br>22) *overlapping speech*<br>23) Interviewee [XXX]: I use yes I use MBACE<br>**24) Interviewer [MS]: okay**<br>25) Interviewee [XXX]: *overlapping unclear speech*<br>**26) Interviewer [MS]: LBASE?**<br>27) Interviewee [XXX]: NBASE<br>**28) Interviewer [MS]: NBASE, whats that?**<br>**29) *no answer***<br>**30) Interviewer [MS]: what is that sorry?**<br>31) Interviewee [XXX]: Its just a book, a book from<br>32) *overlapping speech*<br>33) Interviewee [XXX]: *unclear speech*<br>**34) Interviewer [MS]: okay**<br>35) Interviewee [XXX]: yeah<br>**36) Interviewer [MS]: Okay. Where do you find that pregnant women generally get their antibiotics from?**<br>37) Interviewee [XXX]: The the pregnant women comes to the clinic *unclear speech*<br>**38) Interviewer [MS]: mhm, d'they get antibiotics from anywhere else? D'they get it from like their pharmacy or y'know without a prescription ever? Or d'they always just get it from the hospital?**<br>39) Interviewee [XXX]: they *unclear word* from the pharma the hospital pharmacy<br>**40) Interviewer [MS]: mhm without a prescription? They can just go?**<br>41) Interviewee [XXX]: yes with a prescription *unclear mumbled speech* hospital pharmacy<br>**42) Interviewer [MS]: so what sorry they I didn't hear that sorry so they can just go they have to have a prescription you were saying**<br>43) Interviewee [XXX]: yes<br>**44) Interviewer [MS]: always**<br>45) Interviewee [XXX]: yes<br>**46) Interviewer [MS]: Okay urm are you aware of any pregnant women who might take antibiotics that haven't been prescribed for them?**<br>47) Interviewee [XXX]: the ive not seen any that from my encounter with them ive not seen like that took antibiotic that was not prescribed<br>**48) Interviewer [MS]: Okay Okay and do you know of any pregnant women who might take herbal**	**does not use guidelines**<br><br>**~~19 - 27) Doesn't use guidelines but there is one. Seems that he/she is uncomfortable and confused about his/her ethics.~~**<br><br>**~~29 - 35) He/ She seems stuck with the previous question due to contrasting answers (17 & 27) and feels uncomfortable to give more details about his/her methodology (book)~~**<br><br>[37] Obtain antibiotics from clinic<br>[39] Obtain from hosp pharmacy<br><br>**~~39-45) He/She seems very conscious of the questions and gives suggested answers~~**<br>**[47] Self-medication: not seen any evidence**	[2] OBTAINING<br><br>[3] SELF-MEDICATION (1/2)<br><br>[4] HERBAL SELF-MEDICATION

**preparations or alternative medications that could work like antibiotics?**	[49] Herbal self-medication (no)	
49) Interviewee [XXX]: no ive not ive not come across them	~~47)He/she gives more detail here compared to the rest of the questions. Seems the answer is pre-assumed.~~	
**50) Interviewer [MS]: Okay that's fine. Urm do you know any methods that might detect or identify self-medication of antibiotics in pregnant women?**		
51) Interviewee [XXX]: pardon?		[3] SELF-MEDICATION (2/2)
**52) Interviewer [MS]: do you know of any methods or ways that you could detect or identify urm self-medication of antibiotics in pregnant women? So when they take it without a prescription**		
53) Interviewee [XXX]: yes I eh uh ah I know that theres an antibiotic that apart from pregnant women other women other men have used it that is metronidazole	[53] Self-medication: Type of antibiotic used [interpretation*: without prescription?]*	
**54) Interviewer [MS]: mhm**		
55) Interviewee [XXX]: then ur another antibiotic is like *unclear word*		
**56) Interviewer [MS]: mhm *overlapping***		
57) Interviewee [XXX]: that is *unclear speech* our pregnant women		
**58) Interviewer [MS]: mhm *overlapping***		
59) Interviewee [XXX]: *unclear speech* something like gastroenteritis		
**60) Interviewer [MS]: mhm okay**	???	
61) Interviewee [XXX]: that's when *overlapping* *unclear speech* medications	~~53-61) Did not understand the question and gives a pre-assumed answer~~	
**62) Interviewer [MS]: fine so if someone was taking antibiotics without you knowing that hadn't been prescribed by a doctor or you how would you know**		
63) Interviewee [XXX]: the *stuttered speech* the reports at the clinic		
**64) Interviewer [MS]: mhm**		
65) Interviewee [XXX]: one of the *unclear speech* reports that they took something like that	???	
**66) Interviewer [MS]: Okay. Do you think it could be useful to have like a simple rapid test or tool or questionnaire that could help identify pregnant women who might be misusing antibiotics without us knowing?**	???	[5] DETECTING 1/2
67) Interviewee [XXX]: urr I don't really *unclear word* this one		
**68) Interviewer [MS]: pardon?**		
69) Interviewee [XXX]: I don't know how to detect no		
**70) Interviewer [MS]: okay okay**		
71) Interviewee [XXX]: *overlapping speech* *unclear speech* divulge the information		

**72) Interviewer [MS]: mhm. So if there was like a questionnaire that maybe had more information on it and then you could ask more questions dya think that would be helpful to kinda detect the women who are taking antibiotics without them being prescribed?**<br>73) Interviewee [XXX]: okay probably *unclear speech* may detect person yeah<br>**74) Interviewer [MS]: mhm so just say there**<br>75) Interviewee [XXX]: *started speaking*<br>**76) Interviewer [MS]: go on go on sorry what were you gonna say**<br>77) Interviewee [XXX]: yes<br>**78) Interviewer [MS]: Okay so if such a tool or questionnaire was available, dya think you would be interested in using it?**<br>79) Interviewee [XXX]: I will I will try<br>**80) Interviewer [MS]: okay**<br>81) Interviewee [XXX]: I will try<br>**82) Interviewer [MS]: and dya think such a tool could be used in like antenatal care settings, or in routine appointments, or like A&E? Where dya think it would be best used?**<br>83) Interviewee [XXX]: yes<br>**84) Interviewer [MS]: where dya think it would be used best? Or in all of them?**<br>85) Interviewee [XXX]: *unclear start of sentence* I think so<br>**86) Interviewer [MS]: In all of them?**<br>87) Interviewee [XXX]: yes<br>**88) Interviewer [MS]: Okay, dya think it would work better somewhere else like somewhere you know, where do you normally work? What area are you working in?**<br>89) Interviewee [XXX]: I work in teaching hospital<br>**90) Interviewer [MS]: okay so dya think it would be best kind of in an antenatal clinic or like when they come in in labour when its an emergency like where dya think it would maybe be best used?**<br>91) Interviewee [XXX]: *stuttered start of sentence* It can be used in antenatal clinic<br>**92) Interviewer [MS]: mhm mhm**<br>93) Interviewee [XXX]: *unclear mumbled speech* antenatal clinic<br>**94) Interviewer [MS]: okay okay that's fine. And just say if we had a test do you think it would need to be like remote easy to use dya think we would be able to**	**[69] Detecting SM (don't know how to)**<br>**???**<br>**???**<br>**73)After suggestion she/ he thinks that a tool might detect AB without prescription**<br>**79-81) He/She is willing to use such tools as suggested**<br>**83-93) He/she is not very thoughtful of the application of the tool in practice and gives suggested answers**<br>**95-134) He/She lost patience and is inflexible to the questions regarding the tools that**	**[6] DETECTING (Attitude towards medical evolution/novelty)**

**use it with internet or is it better for it to not have to need internet?**<br>95) Interviewee [XXX]: uuuurr I don't really get you<br>**96) Interviewer [MS]: so just say if we had a test like were saying like a questionnaire or a tool or someway to detect antibiotic misuse in pregnant women, dya think it would be best for such a test to be kind of mobile easy to carry around not using electricity or internet that kind of thing very accessible**<br>97) Interviewee [XXX]: yes<br>**98) Interviewer [MS]: okay dya have any ideas how that might work?**<br>99) Interviewee [XXX]: no I don't have *mumbled word*<br>**100) Interviewer [MS]: okay that's okay and have you ever come across any methods or guidelines that look at detecting the side effects of antibiotic self-medication in pregnant women?**<br>101) Interviewee [XXX]: ive not really done done come across it<br>**102) Interviewer [MS]: Okay okay that's okay. So as we know antibiotics might cause side effects like stomach upset or rashes or feeling sick, do you think that the presence of such side effects is clear that a patient has taken antibiotics? *stuttered* does it seem that its always from antibiotics when someone has those kind of side effects**<br>103) *background noises noted*<br>104) Interviewee [XXX]: urrr I don't think so<br>**105) Interviewer [MS]: okay that's fine and so when dya kind of know its side effects from antibiotics how can you tell?**<br>106) Interviewee [XXX]: I don't get you<br>**107) Interviewer [MS]: how can you tell when someones having side effects from antibiotics?**<br>108) Interviewee [XXX]: okay urmmm ah it may may take 2 2 or 3 days or even after 1 week to know<br>**109) Interviewer [MS]: mhm *overlapping***<br>110) Interviewee [XXX]: about that<br>**111) Interviewer [MS]: mhm mhm how would you know though what kind of symptoms might they have**<br>112) Interviewee [XXX]: *mumbled unclear speech at start * If they report something like urm no sleeping or some rashes on their body<br>**113) Interviewer [MS]: mhm *overlapping***	**detect self-medication misuse and therefore gives suggested answers.**<br><br>**[104] Side effects… (doesn't think so)**<br><br>**[108] Side effects: how long to know…**<br><br>**[112, 118] Side effects (of antibiotics)**	[7] SIDE EFFECTS

114) Interviewee [XXX]: ill know that there is eh some side effects<br>**115) Interviewer [MS]: from antibiotics?**<br>116) Interviewee [XXX]: yes<br>**117) Interviewer [MS]: okay**<br>118) Interviewee [XXX]: *overlapping* then sometimes sometimes eh the side effects of antibiotics may result to eh gastroenteritis<br>**119) Interviewer [MS]: mhm**<br>120) Interviewee [XXX]: *unclear speech*<br>**121) Interviewer [MS]: mhm**<br>122) Interviewee [XXX]: that's something *unclear speech*<br>**123) Interviewer [MS]: okay and do you know any pregnant women that have developed side effects from antibiotic self-medication?**<br>124) Interviewee [XXX]: I not know<br>**125) Interviewer [MS]: Okay and do you know of any methods or guidelines or protocols that look at managing antibiotic self-medication in pregnant women?**<br>126) Interviewee [XXX]: no<br>**127) Interviewer [MS]: no okay. So this is the last question. Thanks sorry I know its a lot of questions urm so regards so sometimes when pregnant women have side effects from self-medication of antibiotics they might sometimes develop like memory loss or forgetfulness have you ever seen that? Would you know how to XXXage that if that happened?**<br>128) Interviewee [XXX]: ive not seen such<br>**129) Interviewer [MS]: okay so you've never seen anyone kind of have memory loss or forgetfulness as a side effect of antibiotics**<br>130) Interviewee [XXX]: no ive not seen ive not come across such presence<br>**131) Interviewer [MS]: okay dya know what you would do if that happened? Dya have any ideas? Orr you not sure**<br>132) Interviewee [XXX]: *mumbled stuttered speech* no no im not really sure not seen any<br>**133) Interviewer [MS]: okay that's fine that's fine so that's all the questions from me, urm thank you for taking part dya have any questions yourself?**<br>134) Interviewee [XXX]: I don't know I don't have any questions thank you very much	**[124] side effects (does not know any patients)**<br><br>**[126] side effects – no**<br><br>**[128] side effects – not seen,**<br><br>**[130, 132] side effects – not come across,**	

135) Advised if participant has any questions to let interviewer know. Reminded re airtime card. Advised would send copy of consent form to participant.<br>***136) Interview ended***<br><br>137)<br>138) Interviewee [XXX]:<br><br>**139) Interviewer [MS]:**<br>140) Interviewee [XXX]:		
