# Supplementary material for: Advancing the safe motherhood initiative: A qualitative and sentiment analysis of local physician’s perspectives on antibiotic self-medication during pregnancy in a low- and middle-income country
Source: PLOS Glob Public Health. 2025 Sep 12;5(9):e0004794. doi: 10.1371/journal.pgph.0004794 (PMC12431270; doi:10.1371/journal.pgph.0004794)
Supplement: S1 File — Transcript 4 (CODES & THEMES by KU).pdf. Transcript 6 (CODES & THEMES by KU).pdf. Transcript 7 (CODES & THEMES, by KU).pdf. Transcript 8 (CODES & THEMES by KU).pdf. Transcript 9 (CODES & THEMES by KU).pdf. Transcript 10 (CODES & THEMES by KU).pdf. Transcript 11 (CODES & THEMES, by KU).pdf. Transcript 12 (CODES & THEMES by KU).pdf. Transcript 13 (CODES & THEMES by KU).pdf. Transcript 14 (CODED & THEMES by KU).pdf. Transcript 15_b (CODED & THEMES by KU). pdf. Transcript 16 (CODES & THEMES by KU).pdf. Transcript 17 (CODES & THEMES by KU).pdf. Transcript 18 (CODES & THEMES by KU).pdf. Transcript 19 (CODES & THEMES by HK).pdf. Transcript 20 (CODES & THEMES by HK).pdf. Transcript 21_b (CODES & THEMES by HK).pdfTranscript 22 (CODES & THEMES by HK).pdf. Transcript 25 (CODES & THEMES by HK).pdf. Transcript 27 (CODES & THEMES by HK).pdf. Transcript Sn1 (CODES & THEMES by RS).pdf Transcript Sn6 (pt3) (CODES & THEMES by RS).pdf. Transcript Sn15_a (CODES & THEMES by RS).pdf. Transcript SN17 (pt3) (CODES & THEMES by RS).pd. Transcript Sn21_a (CODES & THEMES by RS).pdf. (ZIP) [file pgph.0004794.s001.zip › Transcript 20 (CODES & THEMES by HK).pdf]

Transcript	Codes	Themes
**Transcription interview 20**<br>**Interviewee: [XXX]**<br>**SN-29**<br>**Interviewer: (MS), Research Assistant**<br>**Number of speakers :3**<br>**Other Attendees: [RS], Research Volunteer**<br>**Time: 12.01pm London time**<br>**Length of interview recording: 21 minutes 52 seconds**<br>**Date: 30th June 2023**<br><br>1) Consent obtained for RS/Research Volunteer to be on the call. Participant confirmed went through information and consent form. Consent obtained on zoom call prior to commencing interview questions. Confirmed not using airtime card for call.		
**2) Interviewer [MS]: Do you prescribe antibiotics to pregnant women?**<br>3) Interviewee [XXX]: Yes we do<br>**4) Interviewer [MS]: okay how lon**<br>5) Interviewee [XXX]: *overlapping speech* we do prescribe urm<br>**6) Interviewer [MS]: *overlapping speech* How**<br>7) Interviewee [XXX]: *background noises* *overlapping speech* we prescribe urm we prescribe for a short course<br>**8) Interviewer [MS]: mhm**<br>9) Interviewee [XXX]: usually within 3 to 5 days	**3 to 9) prescribes antibiotics for a short course within 3 to 5 days**	**1)[PRESCRIBING] Systematic approach in handling antibiotics and its misuse**
**10) Interviewer [MS]: mhm How long have you been prescribing antibiotics for? to pregnant women?**<br>11) Interviewee [XXX]: okay urm since 20 2016<br>**12) Interviewer [MS]: okay**<br>13) Interviewee [XXX]: *unclear speech*<br>**14) Interviewer [MS]: Okay. How long have you been a prescriber for in general?**<br>15) Interviewee [XXX]: okay from 2015<br>**16) Interviewer [MS]: okay fine so how many week time a week dya think you prescribe antibiotics to pregnant women?**<br>**17)** Interviewee [XXX]: urm okay lets say 3 times a week<br>**18) Interviewer [MS]: Okay and what are the 3 most common medical problems that you prescribe them for?**		
19) Interviewee [XXX]: okay urm we usually prescribe antibiotics as prophylaxis for urm prophylaxis against	**19) Commonly prescribed as prophylaxis for**	

chorioamnionitis especially women who present urm *broken up speech* premature rupture of membrane also prescribe antibiotic to women with any secondary infection in pregnancy also prescribe antibiotics as prophylaxis before we conduct em caesarean section<br>**20) Interviewer [MS]: mhm mhm**<br>21) Interviewee [XXX]: or any surgical procedure we also give antibiotic prophylaxis	**chorioamnionitis, secondary infection or surgical procedure.**	
**22) Interviewer [MS]: mhm so do you use any guidelines when you prescribe antibiotics?**<br>23) Interviewee [XXX]: yes urm actually in Nigeria we use the urm society of obstetrics and gynaecology of Nigeria that's *unclear words* also make use of the NICE guideline also make use of the urm also make use of the NICE guideline sometimes we also use R C O G RCOG guideline depending on yep	**23)NICE and RCOG guidelines are used to prescribe antibiotics**	**[6] GUIDELINES 1/3**
**24) Interviewer [MS]: mhm okay amazing. So where do find that pregnant women generally get their antibiotics from d'they get it from hospital pharmacies or clinic pharmacies? Where do they normally get the antibiotics from?**<br>25) Interviewee [XXX]: okay they normally get it from the urm hospitals em pharmacy<br>**26) Interviewer [MS]: okay, d'they get it from anywhere else?**<br>27) Interviewee [XXX]: no the hospitals pharmacy the hospital has its own pharmacy so once you prescribe they get it at the pharmacy	**25-27) Obtain Antibiotics from hospital pharmacy**	**[2] OBTAINING**
**28) Interviewer [MS]: Mhm so are you aware of any pregnant women who might take antibiotics that havent been prescribed for them?**<br>29) Interviewee [XXX]: might I didn't get that?<br>**30) Interviewer [MS]: do you are you aware of any pregnant women that might take antibiotics that haven't been prescribed for them?**<br>31) Interviewee [XXX]: eh yes urm that's very rampant in Africa so women just take antibiotics on their own before meeting the doctor maybe on their *unclear mumbled speech* see the doctor, yes it's a normal occasion normal occurrence in Africa<br>**32) Interviewer [MS]: okay so where do they normally get them from if they've not been prescribed by them?**<br>33) Interviewee [XXX]: *breaths out* okay they get it from local pharmacies and local drug shops<br>**34) Interviewer [MS]: mhm mhm okay so are you aware of any pregnant women who might take**	**31)Self-medication of antibiotics before meeting the doctors is quite normal in Africa**<br><br>**33)Self-Medicated Antibiotics are obtained from a local pharmacy**	**[3] SELF-MEDICATION - Local practices and upgrade to guidelines in antibiotics misuse**

**herbal preparations or alternative medications that might work like antibiotics?**<br>35) Interviewee [XXX]: urrm mmm over here women take herbal medications though but I don't think they see that it work as an antibiotic they just take it for their illness or for their ailment<br>**36) Interviewer [MS]: mm**<br>37) Interviewee [XXX]: they just take herbal medications and alternative um medicines<br>**38) Interviewer [MS]: okay. Dya have any examples of any alternative medications that peop pregnant women**<br>39) Interviewee [XXX]: *overlapping speech*<br>**40) Interviewer [MS]: might use**<br>41) Interviewee [XXX]: okay urm okay pregnant women sometimes take um eh I don't know the names but they are just locally prepared herbs so they don't actually most of them don't actually have a name then some alternative medicine from china like all those um medicine containing *unclear word* and the rest<br>**42) Interviewer [MS]: mhm**<br>43) Interviewee [XXX]: so I don't actually know their name<br>**44) Interviewer [MS]: Okay, so do you know any methods that might detect or identify self-medication of antibiotics in pregnant women? Like how would you know that a pregnant woman was self medicating with antibiotics that aren't prescribed to them?**<br>45) Interviewee [XXX]: okay urm so some of the women can present with maybe some allergies to the drugs in the pres when they now present to the hospital with the allergies during your when you're trying to take a history and get the history of antibiotics previous antibiotics use urm then I think its just through history with em communicating with the patient that most times get the history of em taking abusing or taking antibiotics<br>**46) Interviewer [MS]: ok**<br>47) Interviewee [XXX]: that was not prescribed<br>**48) Interviewer [MS]: Okay. Dya think it could be useful to have like a simple rapid test or tool or questionnaire that could help identify pregnant women who might be misusing antibiotics without us knowing about it?**<br>49) Interviewee [XXX]: okay urm the use of a questionnaire	**or local drug shop**<br><br>**35-37) People take herbal and alternative medicines for illness but not as an antibiotic.**<br><br>**41)The alternative medicines are ambiguous and are prepared locally and some from China**<br><br>**45-47) Self-Medication is detected through allergic symptoms of the antibiotics and the case history of its prescription**<br><br>**53-57) Questionnaires can be useful to**	**[4] HERBAL SELFMEDICATION**<br><br>**[5] DETECTING SELF-MEDICATION ½**

**50) Interviewer [MS]: or a test *overlapping speech***<br>51) Interviewee [XXX]: *overlapping speech*<br>**52) Interviewer [MS]: tool**<br>53) Interviewee [XXX]: okay a questionnaire or a proforma can be designed though for that urm yes that can be done<br>**54) Interviewer [MS]: mhm**<br>55) Interviewee [XXX]: to maybe identify some yes that can be done a questionnaire proforma can be done<br>**56) Interviewer [MS]: Mhm, so if such a questionnaire or proforma was available, would you be interested in using it?**<br>57) Interviewee [XXX]: okay if the questionnaire is available I will I will use it<br>**58) Interviewer [MS]: mhm and do you think**<br>59) Interviewee [XXX]: *overlapping speech* yes I can use it<br>**60) Interviewer [MS]: Okay okay and do you think such a tool could be used within like antenatal care settings, or during routine appointments, or like in A&E?**<br>61) Interviewee [XXX]: okay urm urm those questionnaires can be used um anytime in pregnancy *unclear speech* can be true antenatal care during labour ward during emergency period I think questionnaires can be used anytime not just antenatal period<br>**62) Interviewer [MS]: Where dya think you would use it most?**<br>63) Interviewee [XXX]: okay maybe during antenatal period<br>**64) Interviewer [MS]: okay**<br>65) Interviewee [XXX]: because that's when pregnant women come<br>**66) Interviewer [MS]: mhm mhm. Do you think if there was such a questionnaire or proforma urm it would be useful for it to be like remote easy mobile easy to use without electricity or internet? Or dya not think that would matter?**<br>67) Interviewee [XXX]: ur yeh that would be problem especially in Nigeria because of availability of um power so I think um a paper based questionnaire would be better administered to patients paper based questionnaire can be *unclear speech*<br>**68) Interviewer [MS]: mhm dya have any ideas of how that might work?**<br>69) Interviewee [XXX]: okay urm urm a paper based questionnaire can be printed out and given to	**detect antibiotic misuse, so the doctor is interested in using it.**<br><br>**61-65) The questionnaire can be useful anytime during pregnancy but mostly during antenatal period.**<br><br>**67-73) Mixture of online and offline is preferred due to uncertainty of power supply in Nigeria.**	**5] DETECTING SELF-MEDICATION 2/2**

pregnant women maybe during the antenatal care for them to fill out before urm once they are done with antenatal care submitted but it can also be uploaded back to you<br>**70) Interviewer [MS]: mhm**<br>71) Interviewee [XXX]: or maybe then some antenatal women who er who er who er well knowledged can also able to fill questionnaire loaded on google forms<br>**72) Interviewer [MS]: okay okay**<br>73) Interviewee [XXX]: if you can use a google form<br>**74) Interviewer [MS]: mhm mhm mhm okay amazing. Urm have you come any methods across any methods or guidelines that might help detect side effects of antibiotic self-medication in pregnant women?**<br>75) Interviewee [XXX]: mm yes lots of guideline try to protect pregnant women against antibiotics use and also *unclear speech* antibiotics in pregnancy like<br>76) *overlapping speech*<br>77) Interviewee [XXX]: like the um okay R C Royal college of obstetrics and gynaecology guideline try's to protect women against the use of antibiotics especially when its not necessary<br>**78) Interviewer [MS]: mhm**<br>79) Interviewee [XXX]: *overlapping unclear speech*<br>**80) Interviewer [MS]: so is it do they specifically look at detecting side effects of antibiotic self-medication in pregnant women**<br>81) Interviewee [XXX]: yh yh that's part of the history we try to take from pregnancy *unclear word* that's part of the history we try to take *unclear speech*<br>**82) Interviewer [MS]: fine *overlapping***<br>83) Interviewee [XXX]: *unclear speech* allergy to drugs<br>**84) Interviewer [MS]: Fine. So its more the history or is there guidelines as well or is it more from history that you know kind of li**<br>85) Interviewee [XXX]: its more from history that we know<br>**86) Interviewer [MS]: okay**<br>87) Interviewee [XXX]: that weve tried to more from history<br>**88) Interviewer [MS]: Okay great. So as we know antibiotics can cause side effects like stomach upset, rashes, things like that. Do you think the presence of such side effects in a patient is clear that the patient is taking antibiotics?**	**75-87) RCOG guidelines instruct to detect the misuse of antibiotics in pregnant women through case history taking**<br><br>**89-103) The details of the self-medicated antibiotics can**	**[6] GUIDELINES 2/3**<br><br>**[7] SIDE EFFECTS ½**

89) Interviewee [XXX]: duh her the side effects are not very clear that the patient does um is taking antibiotics but it can be a guide to you<br>**90) Interviewer [MS]: mhm**<br>91) Interviewee [XXX]: to maybe *unclear word* that, so maybe when you *unclear speech* with the patient and don't get the true information if she has been on any antibiotics have to check if the antibiotics can give *unclear word* side effects<br>**92) Interviewer [MS]: mhm dya have any examples of this?**<br>93) Interviewee [XXX]: okay um okay lets say if a woman has been on um okay um ok lets start with um ummm um um um um umm okay this not a typical antibiotic though but maybe when a woman comes and presents with discolouration of her urine urm that can be a side effect *unclear speech* so but that's not the typical antibiotic *unclear speech* but that's the one that can come to my head right now<br>**94) Interviewer [MS]: okay**<br>95) Interviewee [XXX]: discolouration of urine *unclear word*<br>**96) Interviewer [MS]: okay okay that's fine and that would be a sign of maybe antibiotic use?**<br>97) Interviewee [XXX]: yes<br>**98) Interviewer [MS]: okay**<br>99) Interviewee [XXX]: or *unclear speech*<br>**100) Interviewer [MS]: Okay so D'know of any pregnant women who have had side effects of antibiotic self-medication? When its not been prescribed?**<br>101) Interviewee [XXX]: urm okay yes urm yes ive encountered some that present with rashes some dark spots some dark rashes mostly but yes ive met some<br>**102) Interviewer [MS]: okay when the antibiotics haven't been prescribed?**	**lead to verify if the side-effects are only due to consumption of those antibiotics**	
103) Interviewee [XXX]: yes when the antibiotics when they self medicating<br>**104) Interviewer [MS]: okay okay and what kind of happened when that when someone comes and they've got a rash and they've been self medicating with antibiotics, whats kind of what happens?**<br>105) Interviewee [XXX]: okay urm one they stop the medication then um we do some blood tests will check for toxicity check the liver function the kidney function um that's basically but most times when	**105.a) The side-effects are stopped after terminating the use of antibiotics.**<br><br>**105.b) There is a possibility of dysfunctions with the kidney**	**[7] SIDE EFFECTS 2/2**

they stop the medications the side effects usually go away<br>**106) Interviewer [MS]: mhm okay great. Urm do you know or you aware of any methods or guidelines that look at managing antibiotic self medication in pregnant women?**<br>107) Interviewee [XXX]: mmmm ummm no im not aware of any guideline<br>**108) Interviewer [MS]: okay**<br>109) Interviewee [XXX]: *overlapping speech*<br>**110) Interviewer [MS]: or protocols?**<br>111) Interviewee [XXX]: for managing antibiotic um side effects of um self medication<br>**112) Interviewer [MS]: no just not even just kind of not necessarily its just like really just looking at managing antibiotic self medication in pregnant women**<br>113) Interviewee [XXX]: okay ive not actually come across that<br>**114) Interviewer [MS]: Okay that's fine and um then this is the last question sorry I know weve kind of gone through things do you feel like you need a break or anything? Weve just got another question for you or are you happy to continue?**<br>115) Interviewee [XXX]: okay we can continue *unclear speech* last question<br>116) **Interviewer [MS]: That's fine so with regards specifically pregnant women who might have self medicated with antibiotics and developed like signs of memory loss, or forgetfullness, do you know any management options if this happened? If someone came and self medicated with antibiotics and then had memory loss or forgetfulness?**<br>117) Interviewee [XXX]: okay urm when a patient presents with that self medicating presents with memory loss urm okay basically what we do is we try to make sure actually the drug the patient is self medicating was the offending agent<br>**118) Interviewer [MS]: mmm**<br>119) Interviewee [XXX]: so we try to take a history after the history we try to examine the patient to make sure that there are no other causes that will be causing the um take the blood pressure maybe she has had a stoke or something or maybe *unclear speech* something causing the memory loss after weve ruled out every cause of urm memory loss then also try to stop the drug the patient is self medicating with then we try to take toxicity of the	**and liver due to the toxicity.**<br><br>**107-113) There is no guideline on managing self-medication of antibiotics in pregnant women**<br><br>**117)Ensures if the memory loss is due to self-medicated antibiotics**<br><br>**119.a) Careful and detailed history taking eliminates all the possible causes of memory loss and then checks the toxicity of antibiotics.**<br><br>**119.b) Multidisciplinary team is formed to provide intense care for the pregnant women with memory loss**	**[6] GUIDELINES (3/3)**<br><br>**[7] SIDE EFFECTS (neurological, need for clinical assessment, history taking)**

offending agent and check if there is any signs of toxicity urm all is just trying to rule out the make sure the offending self medicating antibiotics is cause of the urm effects then urm okay then due to we work in a teaching hospital environment after the examination we can do some investigations then we manage using the multidisciplinary team approach we try to invite the neurologist also try to invite the other *unclear word* neurologists other specialists to help us *mumbled speech* maybe intensive care unit<br>**120) Interviewer [MS]: mhm**<br>**121)** Interviewee [XXX]: help manage this patient<br>**122) Interviewer [MS]: mhm**<br>123) Interviewee [XXX]: so ah<br>**124) Interviewer [MS]: okay**<br>125) Interviewee [XXX**]:** *overlapping* *unclear mumbled speech*<br>**126) Interviewer [MS]: okay amazing great so that's all my questions do you have any questions about anything**<br><br>*127) Participant asked if there was any guideline on managing a woman who presents with self-medicating antibiotics & presents with side effects. Interviewer responded that research is about knowledge behind that, we are interviewing from Liverpool for research so not in position to advise/give information on that. Interviewer advised that participant should be able to get research report when study finished. Advised if participant has any other questions, participant can contact PI for study. Consent form will be returned to participant.*	**condition *[need for clinical assessment – KU]***	
