# Supplementary material for: Advancing the safe motherhood initiative: A qualitative and sentiment analysis of local physician’s perspectives on antibiotic self-medication during pregnancy in a low- and middle-income country
Source: PLOS Glob Public Health. 2025 Sep 12;5(9):e0004794. doi: 10.1371/journal.pgph.0004794 (PMC12431270; doi:10.1371/journal.pgph.0004794)
Supplement: S1 File — Transcript 4 (CODES & THEMES by KU).pdf. Transcript 6 (CODES & THEMES by KU).pdf. Transcript 7 (CODES & THEMES, by KU).pdf. Transcript 8 (CODES & THEMES by KU).pdf. Transcript 9 (CODES & THEMES by KU).pdf. Transcript 10 (CODES & THEMES by KU).pdf. Transcript 11 (CODES & THEMES, by KU).pdf. Transcript 12 (CODES & THEMES by KU).pdf. Transcript 13 (CODES & THEMES by KU).pdf. Transcript 14 (CODED & THEMES by KU).pdf. Transcript 15_b (CODED & THEMES by KU). pdf. Transcript 16 (CODES & THEMES by KU).pdf. Transcript 17 (CODES & THEMES by KU).pdf. Transcript 18 (CODES & THEMES by KU).pdf. Transcript 19 (CODES & THEMES by HK).pdf. Transcript 20 (CODES & THEMES by HK).pdf. Transcript 21_b (CODES & THEMES by HK).pdfTranscript 22 (CODES & THEMES by HK).pdf. Transcript 25 (CODES & THEMES by HK).pdf. Transcript 27 (CODES & THEMES by HK).pdf. Transcript Sn1 (CODES & THEMES by RS).pdf Transcript Sn6 (pt3) (CODES & THEMES by RS).pdf. Transcript Sn15_a (CODES & THEMES by RS).pdf. Transcript SN17 (pt3) (CODES & THEMES by RS).pd. Transcript Sn21_a (CODES & THEMES by RS).pdf. (ZIP) [file pgph.0004794.s001.zip › Transcript 21_b (CODES & THEMES by HK).pdf]

Transcription interview 21

Interviewee: XXX

SN- 14

Interviewer: (MS), Research Assistant

Number of speakers :3

Other Attendees: (RS), Research Intern

Time: 3.17pm

Length of interview recording: 17 minutes 5 seconds

Date: 30th June 2023

1) **Participant happy for RS to also be present on call. Shown Participant Information Sheet on call & participant read on call as advised not read prior, however ?only shown first page on call by accident (noted on rewatching interview for transcription, however participant had previously been sent Information Sheet & consent form prior to call). Had a question regarding how long session was going to ask, advised 20-30mins. Participant advised had already read consent form and Consent obtained on zoom call prior to commencing interview questions. Participant advised using airtime for call.**

2) **Interviewer [MS]: Do you prescribe antibiotics to pregnant women?**
3) Interviewee [XXX]: Yes I do
4) **Interviewer [MS]: Okay. How long have you been prescribing antibiotics for?**
5) Interviewee [XXX]: a a for more than 10 years
6) **Interviewer [MS]: Okay, great. Urm so how many times in a week do you prescribe antibiotics to pregnant women?**
7) Interviewee [XXX]: eh hu I cant I cant put a number to it but as long as I *unclear speech* and they have indication then I I I give them antibiotics
8) **Interviewer [MS]: so is it quite a lot?**
9) Interviewee [XXX]: pardon?
10) **Interviewer [MS]: Is it quite a lot?**
11) Interviewee [XXX]: Yes
12) **Interviewer [MS]: Okay great and what are the 3 most common medical problems that you prescribe antibiotics for?**
13) Interviewee [XXX]: well I I can't say specifically this is the commonest but the common ones are for respiratory tract infection
14) **Interviewer [MS]: mhm *overlapping***
15) Interviewee [XXX]: urinary tract infections
16) **Interviewer [MS]: Okay, anything else?**
17) Interviewee [XXX]: *pause* yeahh sometimes pre operatively
18) **Interviewer [MS]: Okay great urm and do you use any guidelines when you prescribe antibiotics?**
19) Interviewee [XXX]: occasionally

20) **Interviewer [MS]: okay do you have like whats the name of the guidelines that they that you use at your hospital?**
21) Interviewee [XXX]: we don't have a *unclear speech* guideline sometimes
22) **Interviewer [MS]: mhm *overlapping***
23) Interviewee [XXX]: we will *unclear speech* guidelines for antibiotic *unclear word*
24) **Interviewer [MS]: Okay great. Where do you find that pregnant women generally get their antibiotics from?**
25) Interviewee [XXX]: from the hospital
26) **Interviewer [MS]: okay do they get them from anywhere else they just get them from the hospital pharmacy or do they get them from anywhere else?**
27) Interviewee [XXX]: They are sometimes they also get from pharmacy shops and what we call eh *unclear word* medicine shops in Nigeria
28) **Interviewer [MS]: Okay so do you know any times that like pregnant women that take antibiotics haven't been prescribed the antibiotics they've got them at like you know taken them when they haven't been prescribed**
29) Interviewee [XXX]: yeah occasionally will encounter such situations where women on their own have taken antibiotics yes
30) **Interviewer [MS]: *overlapping speech* dya have any examples?**
31) **Interviewer [MS]: dya have any examples of that?**
32) Interviewee [XXX]: yeah [I***corrected] here, [***this/corrected] is a common practice here for women sometimes when they have eh cold and cattar they already on antibiotics from before coming to the hospital yes
33) Interviewer [MS]: Okay okay and Do you know of like pregnant women who might take like herbal preparations or alternative medications that could work like antibiotics?
34) Interviewee [XXX]: yes they do that a lot here
35) **Interviewer [MS]: okay dya have any examples of the name of like herbal preparations that they use?**
36) Interviewee [XXX]: *broken up unclear speech* most times they they grind garlic ginger and then steam it yeah they do
37) **Interviewer [MS]: mhm mhm, instead of antibiotics?**
38) Interviewee [XXX]: yes they do
39) **Interviewer [MS]: okay dya see that a lot?**
40) Interviewee [XXX]: not necessarily a lot but occasionally yeah
41) **Interviewer [MS]: Okay great. urm do you know of any methods that could identify or detect self-medication of antibiotics in pregnant women?**
42) Interviewee [XXX]: yeah direct question I think if you ask them majority of them will tell you yes
43) **Interviewer [MS]: Mhm Mhm and do you think it could be useful to maybe have like a simple rapid test or tool or questionnaire that might help identify pregnant women who might be taking antibiotics without us knowing?**
44) Interviewee [XXX]: yeah it should be necessary to do that yeah it will be necessary to do that
45) **Interviewer [MS]: okay dya have so dya have any idea how that could work? what kind of thing might be helpful?**
46) Interviewee [XXX]: if there is tool like that that can be administered to them
47) **Interviewer [MS]: mhm**
48) Interviewee [XXX]: by the while the *unclear speech*
49) **Interviewer [MS]: mhm**

50) Interviewee [XXX]: then first and *unclear speech* nurses before they make contact with the doctors
51) **Interviewer [MS]: mhm**
52) Interviewee [XXX]: the nurses can help them to go through the questionnaire and they will
53) **Interviewer [MS]: mhm**
54) Interviewee [XXX]: volunteer the answers and then at the end of the clinic you *unclear speech* you *unclear speech* their case files are moved and then
55) **Interviewer [MS]: mhm**
56) Interviewee [XXX]: that can be attached for the doctor to know
57) **Interviewer [MS]: mhm so if such a tool or a questionnaire or a test was available, would you be interested in using it?**
58) Interviewee [XXX]: sure sure
59) **Interviewer [MS]: Okay and dya think that such a tool or questionnaire could be used in like antenatal care settings, or routine appointments, or in A&E? Like where dya think it would be best used?**
60) Interviewee [XXX]: best used in the antenatal clinic
61) **Interviewer [MS]: okay okay are you happy to continue or dya want a break or anything between the questions?**
62) Interviewee [XXX]: no um im im *broken up speech* happy to continue
63) **Interviewer [MS]: Okay amazing. Urm so just say we had a test um that we were talking about do you think it would be useful for it to be mobile or remote like easy to use without internet or electricity? How dya think it would be best kind of used in practice?**
64) Interviewee [XXX]: repeat please
65) **Interviewer [MS]: so just say if we had a test or a tool or a questionnaire dya think it would need to be like mobile or remote or to be able to be used without internet or electricity like how dya think it would work best in like the environment?**
66) Interviewee [XXX]: okay it can be it can be a a it can be a printed document
67) **Interviewer [MS]: mhm**
68) Interviewee [XXX]: it can also be it can also be an electronic document that can be circulated either
69) **Interviewer [MS]: mhm**
70) Interviewee [XXX]: through the social media handles of the women
71) **Interviewer [MS]: mhm mhm okay and have you come across any methods or guidelines which could help detect side effects of antibiotic self-medication in pregnant women?**
72) Interviewee [XXX]: no
73) **Interviewer [MS]: okay you've never come across anything like that?**
74) Interviewee [XXX]: yeh
75) **Interviewer [MS]: Okay so as we know antibiotics can cause side effects like stomach upset, rashes urm do you think the presence of such side effects in a patient is clear evidence that the patients taking antibiotics?**
76) Interviewee [XXX]: no I don't think so *distorted speech*
77) **Interviewer [MS]: okay dya have any can you expand on that? How would you maybe know that someone whats clear evidence that someone's taking antibiotics? From side effects**
78) Interviewee [XXX]: well that's eh If you have index of suspicion then you corroborate with the womans information I think that's that's sure *end of sentence hard to interpret*

79) **Interviewer [MS]: Okay and urm do you know any pregnant women who have suspected to have developed side effects from antibiotic self-medication when its not been prescribed by a doctor?**
80) Interviewee [XXX]: I can't remember
81) **Interviewer [MS]: Okay okay you don't know if you've ever seen that?**
82) Interviewee [XXX]: no we've seen that but specifically I can't place my hand on a any at present
83) **Interviewer [MS]: okay okay. So do you know of any methods or guidelines or protocols that look at managing antibiotic self medication in pregnant women?**
84) Interviewee [XXX]: no
85) **Interviewer [MS]: Okay and then last question so with regards to the specific area of pregnant women who have self medicated with antibiotics and then developed like signs of memory loss, forgetfullness, dya know of any management options if this happened from self medication of antibiotics?**
86) Interviewee [XXX]: yeah are the management options for me would be based on what the patient is coming down with
87) **Interviewer [MS]: okay so if they had like memory loss or forgetfulness what would kind of be the management plan for that or options?**
88) Interviewee [XXX]: I would send send to neurology
89) **Interviewer [MS]: mhm have you seen anything like that before?**
90) Interviewee [XXX]: not at all
91) Interviewer [MS]: okay okay that's great thank you so that's all my questions
92) Thanked for taking part. Participant didn't have any questions. Interviewer brought up airtime card reimbursement again. Interviewer advised will send participant copy of consent form.
