# Supplementary material for: Advancing the safe motherhood initiative: A qualitative and sentiment analysis of local physician’s perspectives on antibiotic self-medication during pregnancy in a low- and middle-income country
Source: PLOS Glob Public Health. 2025 Sep 12;5(9):e0004794. doi: 10.1371/journal.pgph.0004794 (PMC12431270; doi:10.1371/journal.pgph.0004794)
Supplement: S1 File — Transcript 4 (CODES & THEMES by KU).pdf. Transcript 6 (CODES & THEMES by KU).pdf. Transcript 7 (CODES & THEMES, by KU).pdf. Transcript 8 (CODES & THEMES by KU).pdf. Transcript 9 (CODES & THEMES by KU).pdf. Transcript 10 (CODES & THEMES by KU).pdf. Transcript 11 (CODES & THEMES, by KU).pdf. Transcript 12 (CODES & THEMES by KU).pdf. Transcript 13 (CODES & THEMES by KU).pdf. Transcript 14 (CODED & THEMES by KU).pdf. Transcript 15_b (CODED & THEMES by KU). pdf. Transcript 16 (CODES & THEMES by KU).pdf. Transcript 17 (CODES & THEMES by KU).pdf. Transcript 18 (CODES & THEMES by KU).pdf. Transcript 19 (CODES & THEMES by HK).pdf. Transcript 20 (CODES & THEMES by HK).pdf. Transcript 21_b (CODES & THEMES by HK).pdfTranscript 22 (CODES & THEMES by HK).pdf. Transcript 25 (CODES & THEMES by HK).pdf. Transcript 27 (CODES & THEMES by HK).pdf. Transcript Sn1 (CODES & THEMES by RS).pdf Transcript Sn6 (pt3) (CODES & THEMES by RS).pdf. Transcript Sn15_a (CODES & THEMES by RS).pdf. Transcript SN17 (pt3) (CODES & THEMES by RS).pd. Transcript Sn21_a (CODES & THEMES by RS).pdf. (ZIP) [file pgph.0004794.s001.zip › Transcript 22 (CODES & THEMES by HK).pdf]

Transcription interview 22

Interviewee: XXX

SN- 39

Interviewer: (MS), Research Assistant

Number of speakers :3

Other Attendees: (RS) Research intern

Time: 3.59pm

Length of interview recording: 21 minutes 39 seconds

Date: 30th June 2023

Participant happy for RS research intern to be on call as well listening in. Participant advised has read participant information sheet and has no questions regarding information sheet. Consent form completed on zoom call prior to interview questions commencing, participant consented to take part. Confirmed participant is not driving as appears to be in a car, participant confirms is a passenger. Participant using headset and is using airtime. Interviewer informed regarding reimbursement for airtime card.

1. **Interviewer [MS]: So do you prescribe antibiotics to pregnant women?**
2. Interviewee [XXX]: yeh I do
3. **Interviewer [MS]: Okay. How long have you been a prescriber for?**
4. Interviewee [XXX]: uhhh 4 years
5. **Interviewer [MS]: Okay and how many times a week do you prescribe antibiotics to pregnant women?**
6. Interviewee [XXX]: eh on average of once a week
7. **Interviewer [MS]: Okay so what are the 3 most common medical problems that you prescribe antibiotics for?**
8. Interviewee [XXX]: urmmm premature rupture of membranes
9. **Interviewer [MS]: mhm**
10. Interviewee [XXX]: urrr urmm urinary tract infection
11. **Interviewer [MS]: mhm**
12. Interviewee [XXX]: urr what else *unclear word* sepsis
13. **Interviewer [MS]: Okay Okay so do you use any guidelines when you prescribe antibiotics?**
14. Interviewee [XXX]: urrrrr not really not really not really
15. **Interviewer [MS]: okay does the hospital have any guidelines that you use that you can use**
16. Interviewee [XXX]: errr our department has a guideline yeah
17. **Interviewer [MS]: mhm okay fine so theres guidelines but do people not generally dya kind of dependent on the situation orr**
18. Interviewee [XXX]: yeah

19. **Interviewer [MS]: things okay**
20. **Interviewer [MS]: Fine so where do you find that pregnant women generally get their antibiotics from?**
21. Interviewee [XXX]: sorry can you repeat that
22. **Interviewer [MS]: where do you find that pregnant women generally get their antibiotics from?**
23. Interviewee [XXX]: eh most of them get from eh over the counter
24. **Interviewer [MS]: mhm**
25. Interviewee [XXX]: from eh partent (note unclear word) medicine dealers
26. **Interviewer [MS]: mhm mhm okay**
27. Interviewee [XXX]: *overlapping speech* that's where most of them get you know because we are developing country and most pregnant women are not eh are not well informed about antibiotic resistance
28. **Interviewer [MS]: mhm**
29. Interviewee [XXX]: so they get antibiotics from over the counter and maybe when It doesn't work that's when they come to the hospital
30. **Interviewer [MS]: okay okay so similar question so do you know of that pregnant women kind of are taking antibiotics that havent been prescribed for them is that quite common for you to see?**
31. Interviewee [XXX]: yeah yeah its quite common
32. **Interviewer [MS]: okay dya have any examples of like where they get them or what kind dya know what I mean kind of what happens?**
33. Interviewee [XXX]: ur like I said no around here eh people some pregnant women or most pregnant women are not educated
34. **Interviewer [MS]: mhm**
35. Interviewee [XXX]: even the educated ones are not well informed
36. **Interviewer [MS]: mhm**
37. Interviewee [XXX]: so whenever they they have some symptoms of eh infection like fever headache the first thing that comes to their mind is to go to partent medicine dealer or over the counter drugs to get some eh antibiotics and eh when the antibiotics didn't work eh then they now present to the hospital also they also eh *unclear speech* antibiotics abuse where they where they don't complete the doseage of antibiotics the required days and the dosage some of them even combine antibiotics ehh irrationally you know because they don't know how supposed to be taken so when these things don't work that's when they present to the hospital
38. **Interviewer [MS]: Okay, so do you know of pregnant women who might take like herbal preparations or any kinds of alternative medications that might work like antibiotics?**
39. Interviewee [XXX]: eh yeah pregnant women take some herbal medications that they believe that work like antibiotics but eh because this herbal medication has not been certified by eh our countries ehh what we call the navtak so we don't know whether it works or not but most of them believe that it works however some of them still come down with eh some eh kidney issues from taking of these herbal medications
40. **Interviewer [MS]: d what dya have like examples of the herbal medications or alternative medications that they use?**
41. Interviewee [XXX]: urr honestly I don't have a name

42. **Interviewer [MS]: mhm**
43. Interviewee [XXX]: but I know that eh they they get some leaves
44. **Interviewer [MS]: mhm**
45. Interviewee [XXX]: I I don't know the botanical name of those leaves
46. **Interviewer [MS]: mhm**
47. Interviewee [XXX]: some of them also get some some eh roots of some trees
48. **Interviewer [MS]: mhm**
49. Interviewee [XXX]: which they boil
50. **Interviewer [MS]: mhm**
51. Interviewee [XXX]: after boiling it they now drink the water that is extracted from the root of those trees
52. **Interviewer [MS]: mhm**
53. Interviewee [XXX]: but im not sure I know the exact names of those roots or the leaves
54. **Interviewer [MS]: Okay that's fine, urm this is just about kind of the next questions are about identifying like methods that can detect self medication of antibiotics so do you know of any methods that can detect or identify self-medication of antibiotics in pregnant women?like how would you know that a pregnant woman was self medicating with antibiotics that hasn't been prescribed?**
55. Interviewee [XXX]: eh I think you know from asking the patient yeah if you ask the patient and then you try to provide them eh confidentiality and you make them comfortable they will be able to divulge that information that they've been taking over the counter antibiotic that have not been prescribed
56. **Interviewer [MS]: mhm and dya think it could be useful to have a like a simple rapid test or tool or questionnaire that might help us identify pregnant women who could be misusing antibiotics without us knowing?**
57. Interviewee [XXX]: yeah yeah yeah I think that would be very helpful because eh if we can *unclear word * the amount of pregnant women that are taking over the counter antibiotics we will be able to reduce antibiotic resistance and also improve the potence of antibiotics that we use around here
58. **Interviewer [MS]: mhm dya have any examples of how this could work?**
59. Interviewee [XXX]: uh like you mentioned a questionnaire I don't know if theres any tool that will be able to do that maybe I don't know im not aware theres anyone avaliable but I think if a questionnaire can be designed whereby a woman that requires antibiotics so a woman that presents with symptoms of infection will be able to go through the questionnaire *unclear words* will be able to to inform the health worker if this woman has been taking over the counter antibiotics
60. **Interviewer [MS]: mhm so if there was a tool or a questionnaire or proforma that was available, would you be interested in using it?**
61. Interviewee [XXX]: yeah yeah I will I will yeah
62. **Interviewer [MS]: Okay and do you think such a tool or questionnaire could be used in like antenatal care settings, or during routine consultations, or like A&E? Like where dya think it would be best used?**
63. Interviewee [XXX]: I think it can be used during antenatal even in routine visits it can be used
64. **Interviewer [MS]: ah ha *overlapping speech***

65. Interviewee [XXX]: in all those scenarios
66. **Interviewer [MS]: okay fine are you happy to continue or dya need a break or anything from the questions or your happy to continue?**
67. Interviewee [XXX]: yeah im happy to continue
68. **Interviewer [MS]: *overlapping speech*okay cool**
69. **Interviewer [MS]: So if there was a tool or questionnaire or proforma dya think it would be useful for it to be mobile easy like easy to use without internet or electricity? Or dya not think it would matter really**
70. Interviewee [XXX]: er well em I think it should be a combination of both because eh people that are eh tech savvy can be able to use the mobile questionnaire then the a lot of people that are not tech savvy around here a lot of women that not even own a mobile phone development do not have access to the internet I believe those kind of women can now use the manual questionnaire
71. **Interviewer [MS]: mhm**
72. Interviewee [XXX]: so I think it work both ways
73. **Interviewer [MS]: mhm okay and dya think like electricity might be an issue or internet?**
74. Interviewee [XXX]: eh I think the major issue is ehh the knowledge accessibility then internet is also an issue
75. **Interviewer [MS]: mhm mhm**
76. Interviewee [XXX]: electricity is an issue but I don't think it's a major issue cuse around here *unclear speech* don't have a stable power supply but at least an average pregnant woman will be able to access electricity so I think the major problem here should be the knowledge that okay that this can be accessed in the internet then secondly is the accessibility do they have a mobile phone that is eh internet enabled then thirdly is that even if they have a mobile phone that is internet enabled can they be able to surf the internet to be able to go through this questionnaire if its put into their mobile phone
77. **Interviewer [MS]: okay so dya think what you're thinking it would be something that women would fill out at home rather than or before rather than something that maybe a healthcare professional carry out in the hospital? For women?**
78. Interviewee [XXX]: ahhhh yes I think eh if eh *unclear speech* it will be better if the healthcare professional carries it out in the hospital
79. **Interviewer [MS]: mhm**
80. Interviewee [XXX]: it will be more effective and eh the healthcare professional will be able to ask directed questions
81. **Interviewer [MS]: mhm**
82. Interviewee [XXX]: that will help to know if this woman *unclear speech* pregnant women are eh using em eh eh an over the counter antibiotic so
83. **Interviewer [MS]: mhm**
84. Interviewee [XXX]: I think it should be too that the healthcare professional will get able to use for the pregnant women however eh for the women that are that ah eh ehhh tech savvy people that can access the internet I don't think it will hurt if they can also access it from the comfort of their homes
85. **Interviewer [MS]: mhm okay that's cool, so have you come across any methods or guidelines that help detect side effects of antibiotic self-medication in pregnant women?**

86. Interviewee [XXX]: ahhh ive come across a lot of them cant remember any particular one but ive come across a lot of eh documents that eh highlighted the side effects of antibiotics self medication in pregnancy
87. **Interviewer [MS]: okay dya have an example?**
88. Interviewee [XXX]: ehhhhh for example the side effects of the mother or the baby
89. **Interviewer [MS]: no its more like methods or guidelines about detecting the side effects of antibiotics**
90. Interviewee [XXX]: guideline for detecting the side effects
91. **Interviewer [MS]: mhm mhm**
92. Interviewee [XXX]: okay no ive not come across something like *mumbled speech*
93. **Interviewer [MS]: Okay okay, urm so as we know antibiotics can cause side effects like stomach upset, rash things like that dya think the presence of such side effects in a patient is clear evidence that the patients taking antibiotics?**
94. Interviewee [XXX]: errrr yeah yeah urm it might not be clear evidence because theres some other disease can present with those side effects so its eh it might be difficult to say if it's the antibiotics that causing it however when you get those symptoms and someone that is taking over the counter antibiotics especially when its not prescribed by a medical personal you can *unclear speech* this is most likely cause
95. **Interviewer [MS]: mhm**
96. Interviewee [XXX]: but never say never
97. **Interviewer [MS]: mhm**
98. Interviewee [XXX]: you cant say that you're 100% sure that this cause but atleast it will be the top of your list that this is most likely being caused by the antibiotics that being taken by the patient
99. **Interviewer [MS]: Okay so do you know any pregnant women that have like developed side effects from antibiotic self-medication? Have you ever seen that?**
100. Interviewee [XXX]: yeah yeah yeah ive seen
101. **Interviewer [MS]: can you expand? Like what kind of side effects have they had?**
102. Interviewee [XXX]: okay urm ive seen pregnant women that had multiple side effects like eh vomiting ehh tiredness urm skin rashes urm and eh some of them also come down with em em decreased urine output
103. **Interviewer [MS]: mhm**
104. Interviewee [XXX]: you know yeah so ive seen and the issue is that whenever they come they arent even aware that eh what is causing the issue is because of the antibiotics they've taken
105. **Interviewer [MS]: mhm okay**
106. Interviewee [XXX]: so ive seen *unclear speech* of those cases
107. **Interviewer [MS]: form antibiotic self medication?**
108. Interviewee [XXX]: yes yes yes
109. **Interviewer [MS]:Okay great thank you. And do you know any methods or guidelines or protocols that manage antibiotic self medication in pregnant women?**
110. Interviewee [XXX]: no I don't
111. **Interviewer [MS]: okay that's fine and final question, um regards to the specific area of pregnant women who have self medicated with antibiotics and maybe develop side effects**

like memory loss, or forgetfulness from that, do you know of any management options of what you would do if someone had self medicated with antibiotics and then had like memory loss or forgetfulness?

112. Interviewee [XXX]: eh yeah first of all you you you'd take a history and when you get the history of antibiotics intake you examine the patient eh you do a a a nervous system examination and I think that's where you will be able to detect the memory loss or forgetfulness then after that you can send for investigation where you try to eh assess the blood level of the antibiotic that the patient *unclear word* and you can also go ahead to do a liver function test or kidney function test and also do a urinalysis eh all this would try to tell you about an effect on those organs then eh this patient will be eh this patient will be good candidate for fluid therapy so that you able to dilute the antibiotics in the system then eh this is also a patient that you want to manage with a multidisciplinary team where you want to involve the neurologist eh and eh possibly even the infectious disease specialist eh even the clinical pharmacologist also also be involved in management of this patient and so this patient should be admitted be given *unclear word, ?fluid* ah possibly some medications possibly steriods

113. **Interviewer [MS]: mhm**

114. Interviewee [XXX]: and *unclear speech* eh sort out some of the issues that the patient is having so I believe when this patient is admitted given fluid after some hours when the antibiotics have cleared from the system I believe that those symptoms that the patient is having will subside so I think this the issue I don't know protocol off hand but I think this should be the *unclear word* of management

115. **Interviewer [MS]: okay great no thank you very much so that's all my questions**

116. Participant thanked for their time and asked if had any questions. Participant didn't have question but would like to be in future research if possible, to play a major part of them if they need any sample size from Africa. Advised would pass this information onto manager in charge of project. Advised once we have report we should be able to share that once its published. Reiterated about refund for airtime and advised will email consent form.
