# Supplementary material for: Advancing the safe motherhood initiative: A qualitative and sentiment analysis of local physician’s perspectives on antibiotic self-medication during pregnancy in a low- and middle-income country
Source: PLOS Glob Public Health. 2025 Sep 12;5(9):e0004794. doi: 10.1371/journal.pgph.0004794 (PMC12431270; doi:10.1371/journal.pgph.0004794)
Supplement: S1 File — Transcript 4 (CODES & THEMES by KU).pdf. Transcript 6 (CODES & THEMES by KU).pdf. Transcript 7 (CODES & THEMES, by KU).pdf. Transcript 8 (CODES & THEMES by KU).pdf. Transcript 9 (CODES & THEMES by KU).pdf. Transcript 10 (CODES & THEMES by KU).pdf. Transcript 11 (CODES & THEMES, by KU).pdf. Transcript 12 (CODES & THEMES by KU).pdf. Transcript 13 (CODES & THEMES by KU).pdf. Transcript 14 (CODED & THEMES by KU).pdf. Transcript 15_b (CODED & THEMES by KU). pdf. Transcript 16 (CODES & THEMES by KU).pdf. Transcript 17 (CODES & THEMES by KU).pdf. Transcript 18 (CODES & THEMES by KU).pdf. Transcript 19 (CODES & THEMES by HK).pdf. Transcript 20 (CODES & THEMES by HK).pdf. Transcript 21_b (CODES & THEMES by HK).pdfTranscript 22 (CODES & THEMES by HK).pdf. Transcript 25 (CODES & THEMES by HK).pdf. Transcript 27 (CODES & THEMES by HK).pdf. Transcript Sn1 (CODES & THEMES by RS).pdf Transcript Sn6 (pt3) (CODES & THEMES by RS).pdf. Transcript Sn15_a (CODES & THEMES by RS).pdf. Transcript SN17 (pt3) (CODES & THEMES by RS).pd. Transcript Sn21_a (CODES & THEMES by RS).pdf. (ZIP) [file pgph.0004794.s001.zip › Transcript 25 (CODES & THEMES by HK).pdf]

Transcription interview 25

Interviewee: XXX

SN- 7 Kaduna participant at start of discussions, but in private hospital at time of interview.

Interviewer: (MS), Research Assistant

Number of speakers : 2

Other Attendees:

Time: 4.01pm Uk time

Length of interview recording: 22 minutes 11 seconds

Date: 21st July 2023

Participant advised had read information sheet. Discussed consent form as participant had emailed consent form and consent obtained on zoom call prior to starting interview questions. Participant using wifi for call.

Participant advised that was in Kaduna but now at a private hospital in a different region. Advised I would check with manager if any issues. He advised he is going to be going back to Kaduna to work there soon.

1. **Interviewer [MS]: Do you prescribe antibiotics to pregnant women?**
2. Interviewee [XXX]: Yes
3. **Interviewer [MS]: How often do you do that?**
4. Interviewee [XXX]: It's a regular depending on patients presentation
5. **Interviewer [MS]: mhm mhm urm how long have you been**
6. Interviewee [XXX]: *overlap*
7. **Interviewer [MS]: go on sorry**
8. Interviewee [XXX]: when I have patients with err who are pregnant
9. **Interviewer [MS]: mhm (overlap)**
10. Interviewee [XXX]: and have (?urinary) tract infection
11. **Interviewer [MS]: mhm**
12. Interviewee [XXX]: *overlapping unclear speech*
13. **Interviewer [MS]: okay**
14. Interviewee [XXX]: patients with prom
15. **Interviewer [MS]: mhm**
16. Interviewee [XXX]: *unclear speech* usually postpartum
17. **Interviewer [MS]: mhm**
18. Interviewee [XXX]: following delivery sometimes we prescribe then following caesarean section
19. **Interviewer [MS]: mhm**
20. Interviewee [XXX]: we generally prescribe antibiotics

21. **Interviewer [MS]: Okay. How long have you been a prescriber for?**
22. Interviewee [XXX]: well throughout my er residency program *unclear word*
23. **Interviewer [MS]: mhm**
24. Interviewee [XXX]: we were looking at er 15 years
25. **Interviewer [MS]: oh wow 15?**
26. Interviewee [XXX]: 15 about 12 to 15
27. **Interviewer [MS]: 50? 50? Five zero? 15**
28. Interviewee [XXX]: *overlapping speech* 15 one five
29. **Interviewer [MS]: fifteen! I thought you didn't yeah *overlapping speech***
30. Interviewee [XXX]: *overlapping speech*
31. **Interviewer [MS]: I was gonna say *laughing* 15 not 50 I was like I didn't think you you know that's yeah *laughing* 12 to 15 perfect sorry the signals not that good. Urm How many times a week do you prescribe antibiotics for? Like women for?**
32. Interviewee [XXX]: okay we do have urm antenatal twice a week
33. **Interviewer [MS]: mhm**
34. Interviewee [XXX]: so during the antenatal clinic yes and urm during surgeries following surgeries surgeries we have a day for surgeries
35. **Interviewer [MS]: mhm**
36. Interviewee [XXX]: so following surgery *mumbling unclear words*
37. **Interviewer [MS]: okay**
38. Interviewee [XXX]: so lets say 3 to 4 times a week
39. **Interviewer [MS]: Okay. What are the 3 most common medical problems that you prescribe antibiotics for to pregnant women?**
40. Interviewee [XXX]: urinary tract infection in pregnancy
41. **Interviewer [MS]: mhm**
42. Interviewee [XXX]: premature rupture of membrane
43. **Interviewer [MS]: mhm**
44. Interviewee [XXX]: and then post caesarean section
45. **Interviewer [MS]: okay anything else whilst they're pregnant?**
46. Interviewee [XXX]: yes sometimes upper respiratory tract infection
47. **Interviewer [MS]: mhm**
48. Interviewee [XXX]: we do sometimes give antibiotics
49. **Interviewer [MS]: okay**
50. Interviewee [XXX]: pyelonephritis we see a lot of them
51. **Interviewer [MS]: mhm**
52. Interviewee [XXX]: so we prescribe antibiotics
53. **Interviewer [MS]: okay. Where do you do you use any guidelines when you're prescribing antibiotics?**
54. Interviewee [XXX]: yes! We do we use ur guidelines but most of them are *unclear speech* are not just like *unclear word*
55. **Interviewer [MS]: mhm**
56. Interviewee [XXX]: *unclear speech* some of them *unclear speech*
57. **Interviewer [MS]: Okay. Where do you find that pregnant women generally get their antibiotics from?**

58. Interviewee [XXX]: well from our local pharmacy within hospitals and then sometimes we have to write so that they can get them from outside the hospital pharmacy there are pharmacies outside
59. Interviewee [XXX]: *unclear overlapping speech*
60. **Interviewer [MS]: Okay *overlapping speech*. So as far as you aware do pregnant women ever take antibiotics that havent been prescribed for them?**
61. Interviewee [XXX]: yes
62. **Interviewer [MS]: dya have more details?**
63. Interviewee [XXX]: yes ur I know of a had situations with patients where they come and then
64. **Interviewer [MS]: *cough***
65. Interviewee [XXX]: *unclear speech* whilst taking history from them tell you they have taken antibiotics and when you dig a little further you find out that they actually got some of these antibiotics over the counter unprescribed by a medical practitioner
66. **Interviewer [MS]: mhm mhm okay. Urm and are you aware of pregnant women who might take herbal preparations or alternative medications that might work like antibiotics?**
67. Interviewee [XXX]: yes we have a very large urrr local traditional population that actually prefer herbal medications some of them don't prefer herbal medication but because of costs of *unclear word* medicines they *unclear word* go with urr they have urr some of those traditional preparations
68. **Interviewer [MS]: mhm mhm**
69. Interviewee [XXX]: and use them as antibiotics yes
70. **Interviewer [MS]: okay dya have any examples of any?**
71. Interviewee [XXX]: well when I was in Zaria during my residency I know I have I encountered *mumbled speech* such but not recently
72. **Interviewer [MS]: dya have any names or examples of any that you know of?**
73. Interviewee [XXX]: that use traditional medications?
74. **Interviewer [MS]: yeah**
75. Interviewee [XXX]: well that will be ill have to go back and check
76. **Interviewer [MS]: no I mean**
77. Interviewee [XXX]: *overlapping unclear speech*
78. **Interviewer [MS]: I mean like the names of the like the the names of the alternative or herbal medication d'they have any names?**
79. Interviewee [XXX]: okay no *mumbled unclear speech*
80. **Interviewer [MS]: you don't know**
81. Interviewee [XXX]: I don't know yeah *speech very quiet*
82. **Interviewer [MS]: Okay that's fine that's fine. So do you know of any methods that might detect or identify self-medication of antibiotics in pregnant women?**
83. Interviewee [XXX]: sorry I didn't get that *mumbled speech*
84. **Interviewer [MS]: d'know any methods that might detect or identify self medication of antibiotics in pregnant women?**
85. Interviewee [XXX]: No *very quiet speech*
86. **Interviewer [MS]: No. Dya think it could be useful to have like a simple rapid test or lab test or tool or questionnaire that could help identify pregnant women who may be misusing antibiotics without us knowing about it?**

87. Interviewee [XXX]: That would be excellent if there's any it will really help

88. **Interviewer [MS]: Okay so if there was such a test or a tool or questionnaire or proforma, would you be interested in using it?**

89. Interviewee [XXX]: yes I will

90. **Interviewer [MS]: okay and just say if there was dya think should a tool could be used within antenatal care settings, or during routine appointments, or like in A&E? Where dya think it would be best used?**

91. Interviewee [XXX]: antenatal settings

92. **Interviewer [MS]: okay dya have any examples of how that might work maybe?**

93. Interviewee [XXX]: yes *unclear speech* urr urr antenatal clinic

94. **Interviewer [MS]: mhm**

95. Interviewee [XXX]: consultations

96. **Interviewer [MS]: mhm**

97. Interviewee [XXX]: *unclear speech* ask questions few questions and then document or give an interview ah ahhhh administered questionnaire

98. **Interviewer [MS]: mhm *overlapping***

99. Interviewee [XXX]: that is very possible

100. **Interviewer [MS]: Okay urm do you think it would be useful for such a test to be like mobile or remote or easy to use without electricity or internet?**

101. Interviewee [XXX]: It should be easy to use without electricity or internet

102. **Interviewer [MS]: okay urm yeah that's dya have any ideas of again how that might work?**

103. Interviewee [XXX]: well that ur if printed

104. **Interviewer [MS]: mhm**

105. Interviewee [XXX]: is really printed yeah printed *unclear speech* printed forms

106. **Interviewer [MS]: mhm okay and have you come across any methods or guidelines which help detect the side effects of antibiotic self-medication in pregnant women?**

107. Interviewee [XXX]: yess ur pharmacovigilance

108. **Interviewer [MS]: mhm**

109. Interviewee [XXX]: *unclear speech* the hospital I was working kaduna

110. **Interviewer [MS]: mhm**

111. Interviewee [XXX]: but eh its rarely rarely used actually *mumbled speech*

112. **Interviewer [MS]: whats it called sorry?**

113. Interviewee [XXX]: the tool we use

114. **Interviewer [MS]: yeah**

115. Interviewee [XXX]: its just a reporting system if urr there is any suspected adverse effects

116. **Interviewer [MS]: fine *overlapping speech***

117. Interviewee [XXX]: *overlapping speech* of course one should report

118. **Interviewer [MS]: so its**

119. Interviewee [XXX]: *overlapping unclear speech*

120. **Interviewer [MS]: fine so its more of like a method urm rather than a guideline**

121. Interviewee [XXX]: yes it's a method rather than a guideline *end of sentence quiet*

122. **Interviewer [MS]: okay so dya not really have any guidelines about that?**

123. Interviewee [XXX]: correctly no

124. **Interviewer [MS]: okay dya want to take a break or anything or you happy to continue?**

125. Interviewee [XXX]: *unclear speech* discussion its okay

126. **Interviewer [MS]: pardon?**

127. Interviewee [XXX]: im okay with it lets *unclear word*

128. **Interviewer [MS]: okay *overlapping speech* okay so as we know antibiotics can cause side effects such as stomach upset like rashes not feeling well, do you think the presence of such side effects in a patient is clear evidence that the patient is taking antibiotics?**

129. Interviewee [XXX]: its not clear evidence actually

130. **Interviewer [MS]: okay dya wna expand abit on that?**

131. Interviewee [XXX]: yeah yes urm for us here we notice certain antibiotics especially ur *unclear word*

132. **Interviewer [MS]: mhm**

133. Interviewee [XXX]: *unclear word* not really in pregnancy per say

134. **Interviewer [MS]: mhm**

135. Interviewee [XXX]: generally urrr are really *unclear speech*

136. **Interviewer [MS]: mhm**

137. Interviewee [XXX]: where I work

138. **Interviewer [MS]: mhm *overlap***

139. Interviewee [XXX]: *mumbled unclear speech* and a lot of patients come with diarrhoea

140. **Interviewer [MS]: mhm**

141. Interviewee [XXX]:so when they come *unclear speech* ask them did you use any *unclear speech* say yes and sometimes for a very long time

142. **Interviewer [MS]: mhm**

143. Interviewee [XXX]: *quiet unclear speech* but not really in pregnancy I must clarify that yes

144. **Interviewer [MS]: mhm mhm okay**

145. Interviewee [XXX]: *overlapping speech* so that sutuation yes *unclear word*

146. **Interviewer [MS]: mhm**

147. Interviewee [XXX]: suspect *unclear speech*

148. **Interviewer [MS]: mhm okay. Do you know any pregnant women that have developed side effects of antibiotic self-medication?**

149. Interviewee [XXX]: not in this *unclear speech*

150. **Interviewer [MS]: Okay and are you aware or do you know of any methods or guidelines or protocols that look at managing antibiotic self medication in pregnant women?**

151. Interviewee [XXX]: no

152. **Interviewer [MS]: okay urm this is the last question. So in regards to the specific area of pregnant women who have self medicated with antibiotics and then may develop side effects such as memory loss, or forgetfulness, do you know of any management options if that happened?**

153. Interviewee [XXX]: following the use of antibiotics?

154. **Interviewer [MS]: so if they self medicated with antibiotics**
155. Interviewee [XXX]: yeah
156. **Interviewer [MS]: and then developed like signs of memory loss or forgetfulness what would kind of be the management plan of that**
157. Interviewee [XXX]: such situation is to quickly tell the patient to stop
158. **Interviewer [MS]: mhm**
159. Interviewee [XXX]: the use of such an antibiotic
160. **Interviewer [MS]: mhm**
161. Interviewee [XXX]: and depending on the severity
162. **Interviewer [MS]: yeah**
163. Interviewee [XXX]: of the toxicity one would want to intervene yes
164. **Interviewer [MS]: mhm mhm**
165. Interviewee [XXX]: in situations like the situation I mentioned *unclear word* patient *unclear speech* diarrhoea
166. **Interviewer [MS]: mhm**
167. Interviewee [XXX]: *unclear speech* we want to quickly stop the medication
168. **Interviewer [MS]: *cough***
169. Interviewee [XXX]: resuscitate using intravenous fluid and then closely monitor patients wellbeing
170. **Interviewer [MS]: mhm *overlapping speech***
171. Interviewee [XXX]: *unclear speech*
172. **Interviewer [MS]: mhm**
173. Interviewee [XXX]: *unclear speech*
174. **Interviewer [MS]: okay okay**
175. Interviewee [XXX]: *unclear speech overlapping*
176. **Interviewer [MS]: have you ever seen anything like that happen?**
177. Interviewee [XXX]: hello?
178. **Interviewer [MS]: hi, have you ever seen anything like memory loss memory loss**

background noise noted

179. **Interviewer [MS]: memory loss or forgetfulness no?**
180. Interviewee [XXX]: no no
181. **Interviewer [MS]: okay okay perfect well thank you that is all our questions urm do you have any questions about anything?**

Participant asked what would study be used for, asked if would be used to develop a guide for misuse or inappropriate use of antibiotics in the near future. Researcher advised at present carrying out interviews and then we are going to be able to collate information and write a research report. Advised once we have done that then we will be able to know the outcome. Advised once everything has been completed we should be able to share the study with participant and show the outcomes. Signposted back to information sheet that had been sent to participant. Advised PI name on information sheet, so if participant has any questions can contact PI. Participant interested in study findings. Advised if participant has any other questions to let researcher know.

Thanked for taking part. Completed Consent form will be sent to participant.

Note unsure if Research volunteer was present during interview, as not mentioned in recording of interview but seems that someone else may be in the room. However, from email records link was also sent to Research Volunteer for this zoom call

Also note that participant volume of speech was quite quiet and hard to hear at times.
