# Supplementary material for: Advancing the safe motherhood initiative: A qualitative and sentiment analysis of local physician’s perspectives on antibiotic self-medication during pregnancy in a low- and middle-income country
Source: PLOS Glob Public Health. 2025 Sep 12;5(9):e0004794. doi: 10.1371/journal.pgph.0004794 (PMC12431270; doi:10.1371/journal.pgph.0004794)
Supplement: S1 File — Transcript 4 (CODES & THEMES by KU).pdf. Transcript 6 (CODES & THEMES by KU).pdf. Transcript 7 (CODES & THEMES, by KU).pdf. Transcript 8 (CODES & THEMES by KU).pdf. Transcript 9 (CODES & THEMES by KU).pdf. Transcript 10 (CODES & THEMES by KU).pdf. Transcript 11 (CODES & THEMES, by KU).pdf. Transcript 12 (CODES & THEMES by KU).pdf. Transcript 13 (CODES & THEMES by KU).pdf. Transcript 14 (CODED & THEMES by KU).pdf. Transcript 15_b (CODED & THEMES by KU). pdf. Transcript 16 (CODES & THEMES by KU).pdf. Transcript 17 (CODES & THEMES by KU).pdf. Transcript 18 (CODES & THEMES by KU).pdf. Transcript 19 (CODES & THEMES by HK).pdf. Transcript 20 (CODES & THEMES by HK).pdf. Transcript 21_b (CODES & THEMES by HK).pdfTranscript 22 (CODES & THEMES by HK).pdf. Transcript 25 (CODES & THEMES by HK).pdf. Transcript 27 (CODES & THEMES by HK).pdf. Transcript Sn1 (CODES & THEMES by RS).pdf Transcript Sn6 (pt3) (CODES & THEMES by RS).pdf. Transcript Sn15_a (CODES & THEMES by RS).pdf. Transcript SN17 (pt3) (CODES & THEMES by RS).pd. Transcript Sn21_a (CODES & THEMES by RS).pdf. (ZIP) [file pgph.0004794.s001.zip › Transcript 27 (CODES & THEMES by HK).pdf]

Transcription interview 27

Interviewee: XXX

SN- 3

Interviewer: (MS), Research Assistant

Number of speakers: 2

Other Attendees: -

Time: 3pm UK time

Length of interview recording: 30 minutes 24 seconds

Date: 28th July 2023

Advised emailed participant information sheet and shown on screen. Signal with participant was not good at start of the call. Participant wanted to read information sheet later. Noted signal not good on call at start. Consent form discussed on zoom call and consent obtained on zoom call prior to starting interview questions.

1. **Interviewer [MS]: Do you prescribe antibiotics to pregnant women?**
2. Interviewee [XXX]: Hereeee in Nigeria doctors do the prescription
3. **Interviewer [MS]: mhm**
4. Interviewee [XXX]: im a midwife im a public health *unclear word* so I do yes I do
5. **Interviewer [MS]: *overlapping* mhm. Okay how long have you been prescribing them for?**
6. Interviewee [XXX]:urmmmm I became a midwife about 30 years now so for this 30 years once in a while I do but in my facilities doctors responsibility *unclear speech*
7. **Interviewer [MS]: okay so generally you don't prescribe the antibiotics?**
8. Interviewee [XXX]: doctors responsibility the hospital but when they are not there ill do the charge of the unit when they *unclear word* I can
9. **Interviewer [MS]: How many times a week dya say you prescribe them?**
10. Interviewee [XXX]: rarely
11. **Interviewer [MS]: okay**
12. Interviewee [XXX]: not common not often
13. **Interviewer [MS]: Okay. What are the 3 most common medical problems that you prescribe antibiotics for?**
14. Interviewee [XXX]: sorry repeat *unclear word*
15. **Interviewer [MS]: what are the 3 most common medical problems that women are prescribed antibiotics for?**
16. Interviewee [XXX]: In cases of things like infection
17. **Interviewer [MS]: mhm mhm**
18. Interviewee [XXX]: then yes severe infections like some they have that have these urr vaginal *unclear word* rashes they go for antibiotics so *mumbled speech* form of infection

19. **Interviewer [MS]: mhmm *overlapping***
20. Interviewee [XXX]: like when they complain they have something like severe fever and you can trace it back to *mumbled unclear speech*
21. **Interviewer [MS]: mhm okay do you use any guidelines when you're prescribing antibiotics? *cough***
22. Interviewee [XXX]: *broken up unclear speech* can you hear me?
23. **Interviewer [MS]: yeah I can hear you urm**
24. Interviewee [XXX]: if the person has an underlying infection you might give antibiotics
25. **Interviewer [MS]: okay do yo**
26. Interviewee [XXX]: *overlapping speech* *unclear speech*
27. **Interviewer [MS]: Okay do you use any guidelines when prescribing antibiotics? Or when the doctors prescribe antibiotics?**
28. Interviewee [XXX]: *mumbled speech* theres no guidelines
29. **Interviewer [MS]: Okay. Where do you find that pregnant women generally get their antibiotics from?**
30. Interviewee [XXX]: Pardon?
31. **Interviewer [MS]: where do women**
32. Interviewee [XXX]: repeat *overlapping*
33. **Interviewer [MS]: gen where do women generally get their antibiotics from?**
34. Interviewee [XXX]: okay here if its prescribed the facility has *unclear speech* they buy from the hospital pharmacy there are *unclear word* pharmacy open to the hospital *unclear word* which can they can get from them
35. **Interviewer [MS]: okay and do you know any time d'pregnant women sometimes take antibiotics that havent been prescribed for them?**
36. Interviewee [XXX]: *unclear speech* I can't remember prescribing antibiotics to pregnant mother its so rare
37. **Interviewer [MS]: yeah no I know but sometimes do you ever find d'women ever get antibiotics without a doctor prescribing them for them?**
38. Interviewee [XXX]: yes they do *unclear speech*
39. **Interviewer [MS]: dya have any like how does that happen? How where do they get them from?**
40. Interviewee [XXX]: yeah some people will be *unclear word* ehh somebody that has taken care of may now *unclear speech* my sister my brother *unclear speech* they not want to *unclear speech* don't prescribe always *unclear word* if I make prescription I like *unclear speech* out of the packet and give him the drug chart in the drug card so that you wont even know *unclear speech*
41. **Interviewer [MS]: mhm okay are you aware**
42. Interviewee [XXX]: *overlapping speech*
43. **Interviewer [MS]: go on sorry dya know any pregnant women who take herbal preparations or alternative medications that work like antibiotics?**
44. Interviewee [XXX]: sorry please you said do I know what?
45. **Interviewer [MS]: do women ever use herbal preparations or alternative medicine that works like antibiotics?**
46. Interviewee [XXX]: yeah many women here

47. **Interviewer [MS]: dya have *overlapping speech***
48. Interviewee [XXX]: *unclear speech* herbal concoction
49. **Interviewer [MS]: mhm**
50. Interviewee [XXX]: *unclear speech*
51. **Interviewer [MS]: dya have any examples?**
52. Interviewee [XXX]: yes but you know most of them will not *unclear speech* there are complications *unclear speech* like my younger sister *unclear speech* she was sick and her urrr some complications so when I was asking her she was telling me she was going to hospital *unclear speech* they will say no a doctor gave them
53. **Interviewer [MS]: mhm**
54. Interviewee [XXX]: but most *unclear speech* herbal concoction *cough*
55. **Interviewer [MS]: Okay. So do you know of any ways that can detect when women self-medicate with antibiotics in pregnancy?**
56. Interviewee [XXX]: *cough* well its cultural belief
57. **Interviewer [MS]: but dya know ho**
58. Interviewee [XXX]: *overlapping speech*
59. **Interviewer [MS]: how do you know if a woman is self medicating with antibiotics that havent been prescribed?**
60. Interviewee [XXX]: like when they *unclear speech* now you can see from the size of the child theres some things they take here to reduce the babys body mass to reduce the size *unclear speech* deliver so easy
61. **Interviewer [MS]: mmm**
62. Interviewee [XXX]: one so you can now know from seeing that child
63. **Interviewer [MS]: mmm**
64. Interviewee [XXX]: the size of the child *unclear speech*
65. **Interviewer [MS]: mhm**
66. Interviewee [XXX]: then from taking history *unclear speech*
67. **Interviewer [MS]: mhm**
68. Interviewee [XXX]: I don't know if this what you want im answering
69. **Interviewer [MS]: mhm mhm mhm. What about in what about antenatally? How would you know antenatally?**
70. Interviewee [XXX]: *unclear speech* you know from history taking
71. **Interviewer [MS]: mhm okay**
72. Interviewee [XXX]: *unclear speech* ask the mother what have you done
73. **Interviewer [MS]: yeah**
74. Interviewee [XXX]: where are you coming to the hospital and maybe most of them tend to come to us the end of their third or *unclear speech* pregnancy because *unclear speech* mother why are you just coming to the hospital *unclear speech* you understand
75. **Interviewer [MS]: mhm**
76. Interviewee [XXX]: you can probe more depending on the information but *unclear speech* people and their culture
77. **Interviewer [MS]: mhm**
78. Interviewee [XXX]: they can now open up

79. **Interviewer [MS]: mhm dya think it could be useful to have a simple test or tool or questionnaire that could help identify pregnant women who might be misusing antibiotics without us knowing?**
80. Interviewee [XXX]:its very very important *unclear word*
81. **Interviewer [MS]: how dya think that could work? Dya have an example of how that might work?**
82. Interviewee [XXX]: okay the kind of question you can ask them
83. **Interviewer [MS]: uhuh**
84. Interviewee [XXX]: an example of what *unclear word*
85. **Interviewer [MS]: yeah how d'you think what kind of test or tool or questionnaire might work to identify pregnant women who might be misusing antibiotics?**
86. Interviewee [XXX]: okay like what I do here?
87. **Interviewer [MS]: yeah**
88. Interviewee [XXX]: *unclear speech* *very hard to understand speech* I will ask what have you done some will tell you nothing some will tell you I have been taking *unclear word* I will now probe more to know how have you been taking *unclear speech* and having some irritation
89. Interviewer [MS]: mhm
90. *silence*
91. Interviewee [XXX]: *broken up speech* the person cannot be calm
92. **Interviewer [MS]: mhm mhm**
93. Interviewee [XXX]: *unclear speech* you have to probe more you have to probe more when you now probe more you will start getting the answers you give the person the highest *unclear word* confidentiality *unclear speech* *very hard to interpret speech*
94. **Interviewer [MS]: mhm**
95. Interviewee [XXX]: so the person will be *unclear speech* and you get the information *unclear speech* like people from the remote areas when you see those people *unclear speech* *unclear speech* they tell you no *unclear speech* what have you done *unclear speech* so its from questioning history taking
96. **Interviewer [MS]: mhm *overlapping***
97. Interviewee [XXX]: I will go through *unclear speech*
98. **Interviewer [MS]: Okay. So if if there was a questionnaire or a tool to look at identifying women pregnant that were misusing antibiotics would you be interested in using it?**
99. Interviewee [XXX]: yes I will I will
100. **Interviewer [MS]: okay. Where do you think such a tool would be best used? Dya think maybe in antenatal care settings or during routine appointments or like in A&E? Where dya think it would be best used?**
101. Interviewee [XXX]: Where I think it be okay to have the questionnaire on assessing what is the abuse antibiotic that's what you asking?
102. **Interviewer [MS]: yeah**
103. Interviewee [XXX]: antibiotics is not a common drug in pregnancy unless when *unclear word* prescription so *unclear words* pregnancy not a disease it's a *unclear word* condition *unclear speech* *unclear speech* detrimental to the pregnant woman even the child in the womb
104. **Interviewer [MS]: mhm**

105. Interviewee [XXX]: *unclear speech very hard to understand* it can be a killing condition

106. **Interviewer [MS]: mhm okay so if there was a test if there was like a tool or a questionnaire that was looking at antibiotic misuse in pregnancy dya think it would be useful for the test to be like remote easy to use urm have to use without electricity? How dya think you know would work?**

107. Interviewee [XXX]: repeat your question *unclear word* sorry

108. **Interviewer [MS]: if we had a tool like a questionnaire that was looking at antibiotic use in pregnancy dya think it would need to be like remote or easy to use or dya know have to use without electricity like how dya think it would be best used on the computer on paper?**

109. Interviewee [XXX]: use it on paper here

110. **Interviewer [MS]: mhm mhm elect**

111. Interviewee [XXX]: *overlapping speech*

112. **Interviewer [MS]: tricity**

113. Interviewee [XXX]: yeah it’s a questionnaire flyer in plain sheet

114. **Interviewer [MS]: mhm mhm okay and have you come across any guidelines that look at urm or methods that look at detecting the side effects of antibiotic self-medication in pregnant women?**

115. Interviewee [XXX]: I don’t have any but I know it depends on the drug *unclear speech* for the antibiotic because its not all the antibiotic *unclear speech* if it’s the product the pharmacy that’s producing it it depends *unclear speech*

116. **Interviewer [MS]: okay**

117. Interviewee [XXX]: *overlapping speech* but not all *unclear speech* group of antibiotics

118. **Interviewer [MS]: okay dya need dya want to take a break or anything or you happy to continue? Ive got a few more questions**

119. Interviewee [XXX]: continue because I *unclear speech*

120. **Interviewer [MS]: okay*overlapping speech***

121. **Interviewer [MS]: yeah so we know antibiotics can cause side effects**

122. Interviewee [XXX]: yeah *overlapping speech*

123. **Interviewer [MS]: do you think the presence of such side effects in a patient is clear that the patient is taking antibiotics? So when someone has stomach upset or rash or not feeling well is it clear that that’s from antibiotics?**

124. Interviewee [XXX]: it depends *unclear speech* it depends on the type of antibiotics like some people will have rash if drug reactions it depends some people will have gastro like me if I take drug that is not good for me

125. **Interviewer [MS]: mhm**

126. Interviewee [XXX]: I start having gastritis *unclear speech*

127. Interviewer [MS]: mhm okay

128. Interviewee [XXX]: *overlapping speech*

129. **Interviewer [MS]: pardon?**

130. Interviewee [XXX]: *unclear speech*

131. **Interviewer [MS]: okay**

132. Interviewee [XXX]: the system is reacting with the drug

133. **Interviewer [MS]: mhm**

134. Interviewee [XXX]: *unclear speech*

135. **Interviewer [MS]: Okay. Do you know of any pregnant women that have developed side effects of antibiotic self-medication? So when antibiotics havent been prescribed?**

136. Interviewee [XXX]: *unclear speech* not of current

137. **Interviewer [MS]:okay okay and do you know of any methods or guidelines or protocols that look at managing antibiotic self medication in pregnant women?**

138. Interviewee [XXX]: I don't know any guideline but I know that its not a common prescription in pregnancy

139. **Interviewer [MS]: okay and you know you said thered been some what side effects had you seen from antibiotic self medication?**

140. Interviewee [XXX]: *unclear word* side effects even in a normal person its itch skin and rashes is common

141. **Interviewer [MS]: mhm mhm**

142. Interviewee [XXX]: but it is *unclear speech*

143. **Interviewer [MS]: mhm**

144. Interviewee [XXX]: *unclear speech can't make out speech*

145. **Interviewer [MS]: mhm**

146. Interviewee [XXX]: *unable to hear speech on recording*

147. **Interviewer [MS]: mhm**

148. Interviewee [XXX]: *speech very broken up on recording*

149. **Interviewer [MS]: oh**

150. Interviewee [XXX]: so it depends on the form of *unclear speech*

151. **Interviewer [MS]: I didn't really hear what you were saying cuse you were walking around**

background noise

152. **Interviewer [MS]: I didn't really hear what you were saying**

153. Interviewee [XXX]: sorry are you hearing me?

154. **Interviewer [MS]: I can hear you now yeah**

155. Interviewee [XXX]: *distorted speech* hear me

156. **Interviewer [MS]: not really because you were walking around**

157. Interviewee [XXX]: * unclear speech* have to cut it

158. **Interviewer [MS]: its okay its okay**

159. Interviewee [XXX]: I said that *unclear speech* when they have antibiotic reaction they *unclear speech*

160. **Interviewer [MS]: okay okay so when someones had antibiotic reaction from self medication what kind of side effects do you see?**

161. Interviewee [XXX]: I said rashes forms of rashes

162. **Interviewer [MS]: Okay *overlapping***

163. Interviewee [XXX]: *unclear speech* complications vomiting diarrhoea hydration

164. **Interviewer [MS]: fine**

165. Interviewee [XXX]: *overlapping speech* *unclear speech*

166. **Interviewer [MS]: okay so theres no guidelines to manage when people have reactions from self medication of antibiotics?**

167. Interviewee [XXX]: you bring the person to hospital

168. **Interviewer [MS]: okay so this is the last question. So sometimes its seen that you know maybe that when people self medicate with antibiotics they might develop signs of memory loss or forgetfulness would you know of any management options of what they would do if someone came to the hospital and had signs of memory loss of forgetfulness And theyd self medicated with antibiotics what would you do or what would the hospital do?**

169. Interviewee [XXX]: in a pregnant *unclear word* not seen any pregnant memory loss

170. **Interviewer [MS]: uhu**

171. Interviewee [XXX]: any case of memory *unclear word* I think the neurological surgeon *unclear speech* so the neurologist will be invited

172. **Interviewer [MS]: mhm**

173. Interviewee [XXX]: *unclear speech* always invited *unclear speech*

End of interview questions

Thanked for taking part. Participant asked question about the study, advised study not about a particular drug, no duration of study, not about stage in pregnancy. Advised the study is making the participant confused. Related back to participant information sheet. Participant using airtime card, advised re reimbursement. Advised will send consent form back to participant. Advised to let us know if has more questions.
