# Supplementary material for: Advancing the safe motherhood initiative: A qualitative and sentiment analysis of local physician’s perspectives on antibiotic self-medication during pregnancy in a low- and middle-income country
Source: PLOS Glob Public Health. 2025 Sep 12;5(9):e0004794. doi: 10.1371/journal.pgph.0004794 (PMC12431270; doi:10.1371/journal.pgph.0004794)
Supplement: S1 File — Transcript 4 (CODES & THEMES by KU).pdf. Transcript 6 (CODES & THEMES by KU).pdf. Transcript 7 (CODES & THEMES, by KU).pdf. Transcript 8 (CODES & THEMES by KU).pdf. Transcript 9 (CODES & THEMES by KU).pdf. Transcript 10 (CODES & THEMES by KU).pdf. Transcript 11 (CODES & THEMES, by KU).pdf. Transcript 12 (CODES & THEMES by KU).pdf. Transcript 13 (CODES & THEMES by KU).pdf. Transcript 14 (CODED & THEMES by KU).pdf. Transcript 15_b (CODED & THEMES by KU). pdf. Transcript 16 (CODES & THEMES by KU).pdf. Transcript 17 (CODES & THEMES by KU).pdf. Transcript 18 (CODES & THEMES by KU).pdf. Transcript 19 (CODES & THEMES by HK).pdf. Transcript 20 (CODES & THEMES by HK).pdf. Transcript 21_b (CODES & THEMES by HK).pdfTranscript 22 (CODES & THEMES by HK).pdf. Transcript 25 (CODES & THEMES by HK).pdf. Transcript 27 (CODES & THEMES by HK).pdf. Transcript Sn1 (CODES & THEMES by RS).pdf Transcript Sn6 (pt3) (CODES & THEMES by RS).pdf. Transcript Sn15_a (CODES & THEMES by RS).pdf. Transcript SN17 (pt3) (CODES & THEMES by RS).pd. Transcript Sn21_a (CODES & THEMES by RS).pdf. (ZIP) [file pgph.0004794.s001.zip › Transcript Sn1 (C0DES & THEMES by RS).pdf]

Interview transcript sn1	Initial coding	Themes
1. Interviewer [MS]: I hadn't started recording, sorry		
2. I forgot to record it sorry		
3. So just for the recording, I am just going through the consent form (shows consent form), because it had been signed but we are just going through the boxes. Urm so I am just signing on his behalf with his consent. So, I consent voluntarily to be a participant in this study and understand that I can refuse to answer questions I can withdraw from the study at any time, without giving a reason and without any penalty or my legal rights being affected. Is that okay?		
4. Interviewee [XXX]: Yes		
5. Interviewer [MS]: perfect, sorry I know its quite long	Verbal consent given	
6. Interviewer [MS]: I have been advised about potential risks associated with taking part in this study and have taken these into consideration before consenting to participate (note background noise)		
7. Interviewee [XXX]: Yeah I will participate		
8. Interviewer [MS]: okay		
9. Interviewer [MS]: Urm you agree that the interview can be audio and video recorded, participants are free at any time to decline this if they wish		
10. Interviewee [XXX]: Yeah		
11. Interviewer [MS]: yeah		
12. Interviewer [MS]: Urm, you understand you'll have access to personal data provided, how the data will be stored and what will happen to the data at the end of the project		
13. Interviewee [XXX]: Yeah		
14. Interviewer [MS]: I understand that personal data will remain confidential and that all efforts will be made to ensure that you cannot be identified in reports		
15. Interviewee [XXX]: Yeah		
16. Interviewer [MS]: Yeah - I understand that interview recordings of me may		

be used in the final report and further outputs.		
17. Interviewer [MS]: Yeah, Yeah?		
18. Interviewee [XXX]: Yes, Yes I agree	Airtime Card	
19. Interviewer [MS]: Okay amazing (at same time)		
20. Interviewer [MS]: Perfect. I understand that parts of our conversation will be used urm in future publications or presentations and that all efforts will be made to ensure I cannot be identified in the future reports		
21. Interviewee [XXX]: yes		
22. Interviewer [MS]: and you agree to take part in this study, perfect		
23. Interviewer [MS]: So I have just put your initials in there, I know you said you read through it before didn't you as well when you signed the bottom	Technical Difficulty in zoom	
24. Interviewee [XXX]: Okay		
25. Interviewer [MS]: Okay, perfect , so ill I'll keep that, im I'm going to start the interview if that's okay, I just wanted to ask – do you have an airtime card? Yeah? Yeah You'll have an airtime card		
26. Interviewee [XXX]: I have airtime, go ahead		
27. Interviewer [MS]: Yeah okay amazing		
28. Interviewer [MS]: So urm Its just some questions that I will ask you, if you need me to repeat them just let me know		
29. Interviewer [MS]: Do you prescribe antibiotics to pregnant women?		
30. Interviewer [MS]: Hello? Hello? Oh he's gone on mute		
31. Interviewee [XXX]: Yeah I can hear you		
32. Interviewer [MS]: Oh		
33. Interviewee [XXX]: I can hear you again		

34. Interviewer [MS]: Oh right		
35. Interviewer [MS]: Do you prescribe antibiotics to pregnant women?<br>36. Interviewee [XXX]: Yes<br>37. Interviewer [MS]: Yes, perfect. If yes, how long have you been prescribing them for women?<br>38. Interviewee [XXX]: How long?<br>39. Interviewer [MS]: Yes<br>40. Interviewee [XXX]: Do you mean for each patient or for number of years of practice?<br>41. Interviewer [MS]: Years of practice<br>42. Interviewee [XXX]: 15 years<br>43. Interviewer [MS]: Wow<br>44. Interviewee [XXX]: 15 years	Years of practise as a qualified doctor	1_PRESCRIBING
45. Interviewer [MS]: Okay. How many times a week do you find that you prescribe them for women?<br>46. Interviewee [XXX]: Around once a week	No of times per week antibiotic prescribed	
47. Interviewer [MS]: Once a week okay and then what are the 3 most common problems that you prescribe antibiotics for?<br>48. Interviewee [XXX]: Okay urm, one of the common problems could be infection, like respiratory tract infection, respiratory tract infection	Most common health problem for which antibiotic is prescribed:<br>Infection	

49. then for vaginal discharge	Reproductive	
50. Interviewer [MS]: Mhmm<br>51. Interviewee [XXX]: and for prophylaxis for surgery<br>52. Interviewer [MS]: Perfect	Prophylaxis	
53. Interviewee [XXX]: and delivery for childbirth	Childbirth	
54. Interviewer [MS]: Mhm, great and do you have to follow some guidelines for this, when you're prescribing them, do you have specific guidelines that you use?<br>55. Interviewee [XXX]: No there is no specific guidelines	No guidelines followed when antibiotics prescribed.	6_GUIDELINES
56. Interviewer [MS]: Okay, that's interesting. Okay so generally where do you find that pregnant women obtain get their antibiotics from?<br>57. Interviewee [XXX]: Repeat	Participant asked to clarify/repeat the question	

58. Interviewer [MS]: Where do they get them from? Do they get them kind of the hospital? the pharmacy? health clinics?<br>59. Interviewee [XXX]: From the hospital and pharmacy	Primarily, prescription of antibiotics is given from Hospital and Pharmacy<br><br>Interruption during interview	2_OBTAINING
60. Interviewer [MS]: Do they get them from anywhere else?<br>61. Interviewee [XXX]: * disruption*		
62. Interviewer [MS]: Do they get them? Sorry?<br>63. Interviewee [XXX]: Get them from pharmacy or hospital	Legal prescription of antibiotics taken from hospital and pharmacy	2_OBTAINING
64. Interviewer [MS]: Okay, so as far as you are aware, are there any pregnant women taking antibiotics that haven't been prescribed from them, for them<br>65. Interviewee [XXX]: Repeat	Participant asked to clarify/repeat the question	
66. Interviewer [MS]: As far as you know, are there any women who take antibiotics who aren't prescribed to them<br>67. Interviewee [XXX]: Some take it on their own<br>68. Interviewer [MS]: Mhmm, when they've not been prescribed?	Few reported cases of women misusing antibiotics was reported.	3_SELF-MEDICATION

69. Interviewee [XXX]: Yes,Yes some take them on their own when there not prescribed		
70. Interviewer [MS]: Okay, and then do you find that any pregnant women are taking preparations or any alternative medications that may work like antibiotics but there not antibiotics<br>71. Interviewee [XXX]: Yes they took some other and some take alternative medicine.<br>72. Interviewer [MS]: Mhm	Alternative to antibiotics taken by pregnant women	
73. Interviewee [XXX]: Some USO	Name of an alternative medication: Uso	4_HERBAL SELF-MEDICATION (Uso)
74. Interviewer [MS]: Where do they get them from?<br>75. Interviewee [XXX]: Ur they get, some of them get them from pharmacy, shops	Alternative medication are found in pharmacy and shops	
76. Interviewer [MS]: Oh okay<br>77. Interviewer [MS]: That's very interesting. So what kind of examples are there?<br>78. Interviewer [MS]: Hello?	Unstable internet connection	

79. Interviewee [XXX] (←Shouldn't it be the interviewer or Investigator?): So we lost connectivity there, so this is not uncommon. I will just text him, just a minute<br>80. Interviewee [XXX]: *name of PI* please continue<br>81. Interviewer [MS]: Sorry, we thought we lost connection that's why. So what kind of examples do you have for alternative medicines that they might use?<br>82. Interviewee [XXX]: Continue<br>83. Interviewer [MS]: Can you hear me? Interviewee [XXX]: Okay, continue<br>84. Interviewer [MS]: Can you hear me okay? Interviewee [XXX]: I can hear you<br>85. Interviewer [MS]: I was just asking, what examples, do you have any examples of alternative medicines that women may use?<br>86. Interviewee [XXX]: Yeah, they can use prenata. There is a drug called prenatal<br>87. Interviewer [MS]: Now hes he's gone	Clarifying if the network is stable to continue with the interview<br>Name of Alternative medication: Prenatal used<br>Lost connection due to unstable network	4_HERBAL SELF-MEDICATION

88. Principal Investigator [KU]: Dr *name of participant* are you there? The wiifi wi-fi system can be quite intermittent. If this happens we just need to wait for them to rejoin urm in the last meeting I had this happened around 4 or 5 times<br>89. Interviewer [MS]: Okay<br>90. Principal Investigator [KU]: Its more the norm, the acception<br>91. Interviewer [MS]: Mhm<br>92. Principal Investigator [KU]: It may be desirable to aim for 40 minutes maximum<br>93. Interviewer [MS]: yeah<br>94. Principal Investigator [KU]: with these interviews and accept that there may be connectivity issues<br>95. Principal Investigator [KU]: His phone may die<br>96. Interviewer [MS]: Hi, thanks so much for coming back on, so you were saying prenata?<br>97. Interviewee [XXX]: Yeah they use prenata,<br>98. they also use some other concoction that doesn't have a name.	Issue with network connection is very frequent<br><br>Network connection is a frequent problem<br><br>Name of alternative medication: Prenata<br><br>Alternative medication is made of a mixture of unknown substance.<br><br>Practitioner does not know the name of the alternative medication	4_HERBAL SELF-MEDICATION unknown substance)

99. Interviewer [MS]: Whats prenata?<br>100. Interviewee [XXX]: prenata drug, containing multivitamins and all kinds of drugs. Ferrous sulphate, iron, vitamin c, all kinds of other drugs	Description of the components found in prenatal.	4_HERBAL SELF-MEDICATION (concoction)
101. Interviewer [MS]: Ah. So they use that for like antibiotics?<br>102. Interviewee [XXX]: No they don't use it as antibiotics. They will use it as form of care for their baby, not necessarily for antibiotics	Clarification by the participant that the alternative medication called Prenatal is not used as antibiotics.	
103. Interviewer [MS]: okay, that's fine. Perfect, We will move onto the next question<br>104. Interviewee [XXX]: They will always go to pharmacy for antibiotics, for prescription	Antibiotics available in pharmacy	2_OBTAINING
105. Interviewer [MS]: Fine, so do women sometimes use alternative medicines or therapies for antibiotics? Or? Generally they will go to the hospital?<br>106. Interviewee [XXX]: Yes, they use some alternative , use herbs,	Alternative medication are used instead of antibiotics	4_HERBAL SELF-MEDICATION

107. they don’t have a name		
	Name of alternative medication is unknown	
108. Interviewer [MS]: Okay, great thank you. So next question		
109. Interviewer [MS]: Are you aware of any methods to detect or identify the women that are taking the medication by themselves, like self medication, so women that maybe using herbs instead of antibiotics. Do you have anyway to identify those women?		
110. Interviewee [XXX]: No, its only by asking them when they come to hospital. Can only ask them through history or	History taking is vital when identifying women who have misused antibiotics	
	Identification of women who have misused antibiotics is when they come with complication	5_DETECTION (importance of patient history)
111. maybe something bad has happened in pregnancy, maybe baby die.		
	History is taken to identify women who have misused antibiotics	
112. We are asking medical history.		
	Positive response towards a use of an alternative test to identify women who are using an alternative medication or misusing antibiotics	5_DETECTION (attitude towards)
113. Interviewer [MS]: Okay great. Do you think it would be helpful to have a simple rapid test, or a lab test that would kind of help identify		

women that are having alternative use of antibiotics or misusing antibiotics without you knowing? Would it be helpful to have a kind of rapid test<br>114. Interviewee [XXX]: Yes, perfect, welcome	Positive response for the potential use of test to detect antibiotic misuse in hospital or clinics	5_DETECTION (attitude towards)
115. Interviewer [MS]: And maybe that could be used in the hospital or somewhere in the clinic? That would be useful would you think?<br>116. Interviewee [XXX]: Yes		
117. Interviewer [MS]: Perfect, and if there was such a thing that was possible, like a tool or a test or questionnaire that was available to find those women, would you be interested in using it?<br>118. Interviewee [XXX]: Yes		5_DETECTION (features of tool/test)
	Positive response towards using a tool/test that could identify misuses of antibiotics or alternative medications	
119. Interviewer [MS]: Where would you mainly use it?<br>120. Interviewee [XXX]: You would use it routinely. With those who have complaints, those who have complaints, or from the history that are not taking their drugs or prescriptions, or suspicious of taking	Best use for tests would be routinely to identify women who are misusing antibiotics	

it. The test could be used to identify such people<br><br>121. Interviewer [MS]: great. Do you think that such a test or a tool could be used in antenatal settings, during like routine appointments or busy hospital A&E departments? Where do you think it would be best used?<br>122. Interviewee [XXX]: Yes, the dangers are many so it would be good to have such a test. So that such women were identified they would be counselled and not have bad side effects or adverse effects, and reduce morbidity and mortality rates. So it would be good to have such tests	Features of the tool<br>Rapid tests would be a great tool as misuse of antibiotics is harmful (side effects potentially leading to mortality and morbidity). Therefore, it would help mitigate these negative consequences.	5_DETECTION tool (key characteristics)
123. Interviewer [MS]: In like different areas as well?<br>124. Interviewee [XXX]: Yes	Versatility of the tool (used in different areas) desirable	
125. Interviewer [MS]: Great, and do you think that if such a test would need to be mobile, like easy to carry around not having to use electrical equipment or internet for it, I know sometimes the connection can be bad, or there needs to be more power. Do you think it needs to be more mobile?<br>126. Interviewee [XXX]: Yes, something that is portable would be good, a point of care test		

127. Interviewer [MS]: MMM<br>128. Interviewee [XXX]: it would not require electricity, that would be good<br>129. Interviewer [MS]: mhmm Yeah yeah, I know, just easy to use, quick, great	Portability of the potential test is desirable.<br><br>The device that can run without the requirement of electricity.	
130. Interviewer [MS]: And do you know of any method, or guideline or protocol to detect side effects or antibiotic selfmedication In women, so for women that are taking the medication by themselves, like the herbs, are there are guidelines that tell you how to see the different side effects in them compared to women that may have taken prescribed antibiotics<br>131. Interviewee [XXX]: No, I cannot say for now. There are no guideline, I am aware there is a guideline on safety of drugs, categories of drugs used in pregnancy by the WRCDC, categories, category 1, 2, 3. That one I am aware, but guidelines no, I am not aware of any guideline	No known guidelines which can detect the side effects or misuse of antibiotics.	6_GUIDELINES
132. Interviewer [MS]: So its more like your experience as well , maybe<br>133. Interviewee [XXX]: Yes	Experience of the practitioner is important for being able to identify women who are potentially misusing antibiotics.	5_DETECTION (role of practitioner)
134. Interviewer [MS]: MM, okay so next question. Antibiotics can cause side effects as weve we've talked about, do you think the		

presence of such side effects in patients is clear? So if they have a side effect is it clear that its because of the antibiotics?<br>135. Interviewee [XXX]: If theres there's side effects it would be an indication to not to take that antibiotic or use an alternative antibiotic that would not have side effects<br><br>136. Interviewer [MS]: Have you had any like experiences of that?<br>137. Interviewee [XXX]: The side effects of antibiotics?<br>138. Interviewer [MS]: Yeah<br>139. Interviewee [XXX]: Yeah, I have had some. If we take antibiotics… For drugs that contain sulphur, sulphur drugs<br><br>140. Interviewer [MS]: Mhm, mhmm<br><br>141. Interviewee [XXX]: They will cause some adverse effects for the user.<br><br>142. So Sometimes they don't take the selective ones type of antibiotics they use, some don't take penicillin, they prefer erythromycin.	Presence of side effect would be an indication to use an alternative antibiotic<br><br>Encounter with patients who have had side effects of antibiotics<br><br>Antibiotic can cause adverse effect<br><br>Patients can prefer one antibiotic over the other.	7_SIDE EFFECTS<br><br>7_SIDE EFFECTS

143. So because of the effect of the penicillin group, some also have what we can adverse effects to some drugs like ceftriaxone	Side effect of antibiotic use.	7_SIDE EFFECTS
144. Interviewer [MS]: Mhmm<br>145. Interviewee [XXX]: They might have a brownish or greenish urm watery discharge from the anus as a side effect, which will also not make them want to take the antibiotics	Aversion from the use of prescribed antibiotics due to the fear of its side effects (Discharge from anus)	
146. Interviewer [MS]: Mhmm<br>147. Interviewee [XXX]: Nausea after taking drugs, they will stop taking it.	Aversion from the use of prescribed antibiotics due to the fear of its side effects	7_SIDE EFFECTS
148. They will prefer the one that is mild, that has less side effects		
149. Interviewer [MS]: Great that's really interesting<br>150. Interviewer [MS]: Um so do you know of any women who have been suspected to have developed any side effects from taking antibiotic selfmedication? So as we discussed, not prescribed or self-medicated, do you know of any pregnant women that that's happened to<br>151. Interviewee [XXX]: Yeah I know some of them	Preference over antibiotic which causes less adverse effect	

	Few known cases of women who developed side effects as a result of misuse were reported	
152. Interviewer [MS]: you've had experience? What kinds of side effects have they had?<br>153. Interviewee [XXX]: They have rashes, they have increased fetal movement, most will have reduced fetal movements.		7_SIDE EFFECTS
	Adversities of antibiotics misuse:<br>Concerning fetal movement<br>Allergic reaction	
154. Some will say they took the antibiotics and they have rupture of membranes, although that one is not clear.		
	Although pregnant women who misuse antibiotics are presented with ruptures membrane, there was no clear evidence for such adversity in misuse.	
155. Interviewer [MS]: Mhmm, Mhmm, Which I guess is hard because you don't know what they've taken if its not been prescribed or its abit more complicated isn't it?<br><br>156. Interviewee [XXX]: They took it if its not prescribed		
157. Interviewer [MS]: mm, its more complicated	[mild side effects] suggests pregnant women took unprescribed antibiotics.	

158. Interviewee [XXX]: Dosage, we will not know the dose, if they took low dose or they will take high dose.	Misuse of antibiotic is hard to identify.<br>Hard to identify the dosage intake for unprescribed antibiotics.	5_DETECTION (of dosage difficult)
159. And the route of administration also varies, some like to take injection, prefer to take injections,	Preference of intravenous method rather than oral intake of antibiotics in pregnant woman.	
160. but can be traumatic, it can cause abscess from having it	Intravenous antibiotic injections can result in skin trauma.	
161. Interviewer [MS]: mmm yeah no course<br>162. Interviewer [MS]: mm so are you aware or do you know any methods or guidelines or protocols to manage antibiotic self medication in pregnant women, I know weve we've asked a similar question but this is specific to<br>163. Interviewee [XXX]: I don't know any guidelines	No known existing guideline for managing antibiotic misuse.	6_GUIDELINES

164. Interviewer [MS]: No, perfect<br>165. Interviewer [MS]: and in regards to specifically pregnant women who have self medicated with antibiotics, urm have you seen any that have developed any signs of memory loss or forgetfulness?<br>166. Interviewee [XXX]: Repeat that<br>167. Interviewer [MS]: so with specifically to women, pregnant women who have self-medicated with antibiotics, have you ever noticed that they have displayed any signs of memory loss or forgetfulness?<br>168. Interviewee [XXX]: No no no no	Participant asked to clarify/repeat the question<br><br>No reported known cases of forgetfulness/memory loss in pregnant women who misuse antibiotics.	7_SIDE EFFECTS
169. Interviewer [MS]: and if that happened would there be any management options for that? Or not really?<br>170. Interviewee [XXX]: you mean memory loss?<br>171. Interviewer [MS]: like if they had memory loss or forgetfulness from self-medicating from antibiotics, is there any kind of management of that or process you might follow?<br>172. Interviewee [XXX]: would have to refer to neurologists who would manage that	Suggestion for referral to neurologist in case of memory Loss in pregnant women who misuse antibiotics.	7_SIDE EFFECTS (require referral to a specialist, neurologist)

173. Interviewer [MS]: Okay and you’ve not experienced that or anything?<br>174. Interviewee [XXX]: No ive I’ve not experienced that	No reported known cases of forgetfulness/memory loss in pregnant women who misuse antibiotics.	
175. Interviewer [MS]: Okay, perfect, well thank you very much that is all of the questions<br>176. Principal Investigator [KU]: Dr *name of participant*, sorry *name of RA*, I just interject there. Dr *name of participant*, , I just wanted to say thank you so much for all the effort you’ve made to facilitate this project. Urm you know we are very grateful, we know you are very busy and urr I, you know we do appreciate the effort that you’ve made to put things together. I mean do you know is there anyone around today that can be interviewed this afternoon? Er or this morning? Any of your colleagues?<br>177. Interviewee [XXX]: Urm *name included* is around<br>178. Principal Investigator [KU]: Okay, is there anyway we can can we is there someway for us to reach him? Is he nearby or is there someway for us to, should we just text him or whatsapp<br>179. Interviewee [XXX]: You can send him, Whatsapp him. You can call him. Hes He’s around, I don’t know whether he is around in the clinic now but I will check<br>180. Principal Investigator [KU]: Okay *name of potential participant*<br>181. Interviewee [XXX]: *name of potential participant* is around<br>182. Principal Investigator [KU]: *name of potential participant*okay<br>183. Interviewee [XXX]: *name of potential participant* I saw him today		

name of potential participant, he is on that list *name of potential participant*<br>184. Interviewer [MS]: Okay<br>185. Principal Investigator [KU]: Yes okay, name of potential participant* yes*name of potential participant*<br>186. Interviewer [MS]: I can email them<br>187. Interviewee [XXX]: Urm, contact them through telephone or whatsapp<br>188. Principal Investigator [KU]: Okay, okay<br>189. Interviewer [MS]: Okay<br>190. Interviewee [XXX]: They will respond<br>191. Principal Investigator [KU]: Okay<br>192. Interviewer [MS]: Great, thank you so much. Thank you so much for your time I really really appreciate it and for your patience with all the technical problems<br>193. Principal Investigator [KU]: *name of participant*, don't forget to give your airtime card. We will send you a refund urm at the end of the project<br>194. Interviewee [XXX]: Okay<br>195. Principal Investigator [KU]: Okay, god bless you, thank you very much<br>196. Interviewer [MS]: Thank you so much<br>197. Interviewer [MS]: Thank you, Bye<br>198. [GE now out of Zoom call]<br>199. Principal Investigator [KU]: Right urm<br>200. Interviewer [MS]: Ill stop recording<br>201. Principal Investigator [KU]: Stop recording<br><br>202. Recording stopped		
