# Supplementary material for: Advancing the safe motherhood initiative: A qualitative and sentiment analysis of local physician’s perspectives on antibiotic self-medication during pregnancy in a low- and middle-income country
Source: PLOS Glob Public Health. 2025 Sep 12;5(9):e0004794. doi: 10.1371/journal.pgph.0004794 (PMC12431270; doi:10.1371/journal.pgph.0004794)
Supplement: S1 File — Transcript 4 (CODES & THEMES by KU).pdf. Transcript 6 (CODES & THEMES by KU).pdf. Transcript 7 (CODES & THEMES, by KU).pdf. Transcript 8 (CODES & THEMES by KU).pdf. Transcript 9 (CODES & THEMES by KU).pdf. Transcript 10 (CODES & THEMES by KU).pdf. Transcript 11 (CODES & THEMES, by KU).pdf. Transcript 12 (CODES & THEMES by KU).pdf. Transcript 13 (CODES & THEMES by KU).pdf. Transcript 14 (CODED & THEMES by KU).pdf. Transcript 15_b (CODED & THEMES by KU). pdf. Transcript 16 (CODES & THEMES by KU).pdf. Transcript 17 (CODES & THEMES by KU).pdf. Transcript 18 (CODES & THEMES by KU).pdf. Transcript 19 (CODES & THEMES by HK).pdf. Transcript 20 (CODES & THEMES by HK).pdf. Transcript 21_b (CODES & THEMES by HK).pdfTranscript 22 (CODES & THEMES by HK).pdf. Transcript 25 (CODES & THEMES by HK).pdf. Transcript 27 (CODES & THEMES by HK).pdf. Transcript Sn1 (CODES & THEMES by RS).pdf Transcript Sn6 (pt3) (CODES & THEMES by RS).pdf. Transcript Sn15_a (CODES & THEMES by RS).pdf. Transcript SN17 (pt3) (CODES & THEMES by RS).pd. Transcript Sn21_a (CODES & THEMES by RS).pdf. (ZIP) [file pgph.0004794.s001.zip › Transcript Sn6 (pt3)(CODES & THEMES by RS).pdf]

Interview Transcript (SN 6)	Initial Coding	Open Codes	Axial Codes
2. Interviewee [XXX]: The consent form?<br>4. Interviewee [XXX]: I only received the consent form<br><br>8. Interviewer [MS]: Did you receive it?<br>9. Interviewee [XXX]: I'm not sure if I received it, I will check my emails later *unclear speech*<br><br>15. Interviewee [XXX]: mmm so I will check emails again but that's no problem, go on with the interview	Participant unsure about receiving consent form	**Proper consent could not be obtained**	Issues concerning receiving consent via virtually
5. Interviewer [MS]: Okay so I am just going to show you on here, this is just the participant information sheet, can you see this. I sent this on an email as well. So you can have a read of it quickly now or have a read of it later. It's just about the study and urm the kind	Meticulous process of receiving virtual consent	**Virtual Consent taking is a time-consuming process.**	

of questions we will be asking. Is that okay?<br>6.<br>Interviewee [XXX]: Yes okay<br><br><br>24.<br>Interviewer [MS]: So it says, I confirm that I have read the information sheet dated 17/12/2022 for the above study or it has been read to me. I had the opportunity to consider the information, ask questions and have had these answered satisfactorily<br>25.<br>Interviewee [XXX]: Its okay, no problem<br>26.<br>Interviewer [MS]: You happy? Yeah, urm you understand what taking part in the study involves so its some questions about antibiotic misuse in pregnancy<br>27.<br>Interviewee [XXX]: Yes<br>28.<br>Interviewer [MS]: Yes and you consent voluntarily to take part in this study			

and understand that you can refuse to answer questions and can withdraw from the study from anytime without giving any reason and without penalty or your legal rights being affected<br>29. Interviewee [XXX]: Yes<br>30. Interviewer [MS]: You have been advised about potential risks associated with taking part in the study and have taken these into consideration before consenting to take part?<br>31. Interviewee [XXX]: Yes<br>32. Interviewer [MS]: Um you agree that this interview can be audio and video recorded, you don't have to be audio or video recorded if you don't want to be, its up to you<br>33. Interviewee [XXX]: Yes<br>34. Interviewer [MS]: Perfect, um you understand that we will have access to your			

personal data and how your data will be stored and what will happen to your data at the end of the project. All that information is on the information sheet<br>35. Interviewee [XXX]: Yes<br>36. Interviewer [MS]: Err You understand that personal data will remain confidential and that all efforts will be made to ensure that you cannot be identified in any reports or further outputs<br>37. Interviewee [XXX]: Yes<br>38. Interviewer [MS]: Um you understand that interview recordings may be used in the final report or any further outputs<br>39. Interviewee [XXX]: Yes<br>40. Interviewer [MS]: Um and you understand that parts of our conversation will be used in future publications or presentations and that all efforts will			

be made to ensure that you cannot be identified in reports or any further outputs<br>41. Interviewee [XXX]: Yes<br>42. Interviewer [MS]: Um and you agree to take part in the study?<br>43. Interviewee [XXX]: Yes, okay<br>44. Interviewer [MS]: perfect and its Dr *start to say name*, how do I spell that, is it like that? *spelling confirmed with participant*<br>45. Principal Investigator [KU]: *confirming spelling*<br>46. Interviewee [XXX]: Yes correct<br>47. Interviewer [MS]: Okay perfect *confirms status*, sorry<br>48. Interviewee [XXX]: Yes *confirms status*<br>49. Interviewer [MS]: *laughter* and then ill just put your intials in here<br>50. Interviewee [XXX]: Okay			

7. *unclear speech*<br>16. *slightly unclear*<br>19. Interviewee [XXX]: *quiet unclear speech*<br>100. Interviewee [XXX]: Repeat, pardon<br>104. Interviewee [XXX]: *mumbles, unclear*<br>107. *unclear speech*<br>115. Interviewee [XXX]: *unclear speech*<br>128. Interviewee [XXX]: *unclear speech*<br>126. Interviewee [XXX]: Do I know of eh? repeat please<br>135. *unclear*<br>146. *unclear speech*<br>155. *unclear speech*<br>179. *unclear speech*<br>184. Interviewee [XXX]: *speech in background unclear*	Difficulty in interpretation due to unstable network connection		**Virtual Interview are challenging to conduct**
18. *muffling noises heard*	Disturbance during the interview process	**An ideal setting for conducting virtual**	

54. Interviewee [XXX]: *vibration of phone*<br>63. *unclear speech, phone vibrated*		**interview needs to be assessed**	
71. Interviewer [MS]: Okay, do you follow any guidance when you prescribe antibiotics or any guidelines that you follow? ……….Hello? Hello?<br>72. Interviewee [XXX]:*bad signal*<br>73. Interviewer [MS]: The signal gone abit<br>75. Interviewee [XXX]:*bad signal* helloooo *bad signal*<br><br>78. Principal Investigator [KU]: He might try to reconnect umm there are, there are im not sure if this has much to do with it. There are very bad tropical thunderstorms in that part of the world, so you get one of these storms and it could interfere with the	Network Issues leading to disruption during interview		

connection quiet easily			
61. Interviewee [XXX]: Medical problems, some kind of cough, mmm that's what they usually come with and we give them antibiotics that's all.	**Common health problem for which antibiotic are prescribed for: 1. Medical Problem**	Medical	**Antibiotics use for the most common health problems [[1] PRESCRIBING]**
62. When they come with upper respiratory tract infection, which is common here now that they have 64. dust everywhere, most of them come down with cough, katar 68. Interviewee [XXX]: Respiratory yes	**2. Respiratory Problem**	Respiratory	
66. Interviewee [XXX]: and you give some antibiotics for prophylaxis and also for treatment as well	**3. Prophylaxis**	Prevention	
70. Interviewee [XXX]: yes and occasionally urinary tract infections	**4. Urinary Problem**	Urology	
86. Interviewee [XXX]: Any guidelines ehh yes,	**No recognised guidelines available when**		

urm no particular guidance urr *unclear speech* guidance<br>87. Interviewer [MS]: Mhmm, fine so theres not like guidelines that you follow<br>88. Interviewee [XXX]: Yeah there is no strict guideline<br>91. Interviewee [XXX]: In terms of general use I don't use any particular guidelines<br><br>162. Interviewee [XXX]: umm not come across any protocol really<br><br>177. Interviewee [XXX]: no don't have any guidelines. I know when you take a lot of antibiotics,<br>95. but ehm people that use drugs easily here in Nigeria and they go over the counter and buy some of these antibiotics that should not be over the counter,	**prescribing antibiotics**	**A proper and systematic guideline for safe antibiotic use in pregnancy not implemented**	**Antibiotics has not been recognised as a priority in healthcare**<br><br>**[[6] GUIDELINES]**
89. but we use the guidelines	**Guidelines available only**		

when we have someone with the premature rupture of membranes, that time we use guidelines because we are treating, we are wanting to prevent chorioamnionitis and so forth and that one has its own guidelines	**when there is risk of infection/ potential risk that can endanger life**		**[3] SELF-MEDICATION_KU**
96. sometimes they take these antibiotics before coming to the hospital<br><br>97. Interviewer [MS]: Fine, so you find that that happens a lot? Like generally?<br>98. Interviewee [XXX]: yes this happens a lot<br><br>108. Interviewer [MS]: But sometimes they just go and get their own<br>109. Interviewee [XXX]: yes they buy on their own before coming to your clinic and that's an abuse really	**People take antibiotics on their own without consulting medical professions.**	**The harmful potency of the antibiotics misuse is still not widely established among pregnant women. (Not taken very seriously)**	
118. because most pregnant	**Awareness among most**		

women now they know that they have to consult doctors	**pregnant women that they must consult doctor before taking medication**		**[[2] OBTAINING]**
94. If they come to the hospital you prescribe and they get from the hospital pharmacy,<br><br>102. Interviewee [XXX]: Yes, so when they come to the hospital you prescribe, they go to the hospital pharmacy to pick the drugs<br><br>105. Interviewer [MS]: So when you prescribe them they will go to the hospital pharmacy?<br>106. Interviewee [XXX]: Yes they will go	**Prescribed antibiotics obtained from hospital's pharmacy**<br><br>**Cultural mindset?**	Juxtaposition of the medication distribution of antibiotics in healthcare<br><br>(Both prescribed and non-prescribed medication are given from the same place)	
93. Interviewee [XXX]: oh in this part of the world, In nigeria, I find that many people buy some of these drugs out of eh or just over the counter.<br><br>95. but ehm people that use	**Readily get non-prescribed medication over the counter.**		

drugs easily here in Nigeria and they go over the counter and buy some of these antibiotics that should not be over the counter,<br><br>109. Interviewee [XXX]: yes they buy on their own before coming to your clinic and that's an abuse really			
97. Interviewer [MS]: Fine, so you find that that happens a lot? Like generally?<br>98. Interviewee [XXX]: yes this happens a lot<br><br>117. some people eh take one antibiotic so the other saying that they have typhoid, this is more common among non-pregnant women,<br><br>136. have fever one or two days. They start with typhoid, some of those antibiotics augmention augmentation some drops for thyoid typhoid fever they take that without doing any tests	Pregnant women self-diagnose and misuse antibiotics when showing symptoms (Fever)	**Lack of awareness among pregnant women regarding the haphazard use of antibiotics (which can lead to antibiotic resistance.)**<br><br>Do pregnant women consider antibiotics to be the same as paracetamol? Are they not aware that they might develop antibiotic resistance if they do not complete the dose??	Awareness campaign among the pregnant women about the misuse of antibiotics and antibiotic resistance could be a potential idea to reduce misuse.<br><br>**[3] SELF-MEDICATION_KU**

169. Interviewee [XXX]: mmm yes some of the side effects may present, but normally you tell a patient about side effects and they will come back, when they come back for follow up you ask them about side effects, especially nausea and if they are taking a high dose of antibiotics, most of these patients will have nausea	Misuse of antibiotics causes side effects: Nausea	**Side effects of antibiotics misuse**	**[7] SIDE EFFECTS_KU**
171. Interviewee [XXX]: emm no, not that recently but I know sometimes that come with some kind of rashes and so forth<br>173. Interviewee [XXX]: Allergy, but yeh but you have to find out what they've taken.<br><br>175. I cant recall anyone recently	Misuse of antibiotic causes allergic reaction in pregnant women<br><br>No recent recount of any allergic reaction in pregnant women misusing antibiotics.		
178. some pregnant women come back with candida	Disruption of normal vaginal flora (discharge) examined in pregnant		

181. Interviewee [XXX]: when they come back they come with recurrent, when they have taken a lot of antibiotics its part of the discharge of the vaginal flora,	women who misuse antibiotics		
129. - history, history<br>130. Interviewer [MS]: mhmm<br>131. Interviewee [XXX]: History, asking them the drugs they're taking<br>133. Interviewee [XXX]:ehh when before coming into the hospital, they have taken one or two antibiotics<br>141. yes, from history<br>143. Interviewee [XXX]: They will tell you what they have taken<br>185. Interviewer [MS]: yeah a lot of it is from the history really is it.<br>186. Interviewee [XXX]: mhmm yes most of it<br>182. these are the things you get from the history<br>173. Interviewee [XXX]: Allergy,	History Taking is crucial for detecting antibiotic misuse	**A questionnaire that incorporates essential elements of history taking can help in detecting antibiotic misuse**	**[[5] DETECTING SELF-MEDICATION]**<br><br>A versatile questionnaire developed with the help of the experiences of health care professional could be beneficial for detecting antibiotics misuse.

but yeh but you have to find out what they've taken.<br>141. Interviewee [XXX]: *overlapping* yes a questionnaire, yes, from history			**[[5] DETECTING SELF-MEDICATION] questionnaire**
140. Interviewer [MS]: or like a questionnaire?<br>141. Interviewee [XXX]: *overlapping* yes a questionnaire, yes, from history<br>144. Interviewer [MS]: mhmm so if there was like a tool or a questionnaire or a test, would you be interested in using it?<br>145. Interviewee [XXX]: Yes yes	**Positive response towards a using a questionnaire that can detect misuse in pregnant woman.**	A questionnaire could be beneficial in clinical setting for detecting misuse	
159. Interviewee [XXX]: Yes yes if you have a method that doesn't require any electricity<br>160. maybe just a questionnaire so forth, that would be preferable	**Questionnaires are a great tool due to its versatile nature (doesn't require electricity, easy to use)**	**Features : Versatility of the questionnaires is desirable**	
159. Interviewee [XXX]: Yes yes if you have a method that doesn't	Preference over a test that would not require any electricity.		

require any electricity			
147. Interviewer [MS]: When do you think you would use it the most?<br>148. Interviewee [XXX]: When the patient is present and suggesting symptoms of em infection<br>149. Interviewer [MS]: mhmm<br>150. Interviewee [XXX]: Urinary infection, mastitus, chorioamintus, ehh maybe vaginal discharge, vaginitis *unclear speech*,all the common things<br>152. Interviewee [XXX]: That present within pregnancy<br>154. Interviewee [XXX]: better in the antenatal, antenatal clinics, when they come for consulting and present in the clinics<br>156. Interviewer [MS]: Rather than when its like an emergency?	**Tools would be most useful during antenatal setting when they come for consultation.**<br><br>**Prevention is better than cure. Tools would be better used before there is an emergency.**	When can a test be most useful?	

157. Interviewee [XXX]: yes, even emergency			**[[5] DETECTING SELF-MEDICATION] Skill/proficiency**
163. Interviewer [MS]: okay, so its just from experience really<br>164. Interviewee [XXX]:yes from history and examination, youll know, they will tell you what they've taken,<br><br>165. you may do some investigations, check their liver function, check their *unclear* function test and so on.<br>166. Interviewer [MS]: mhmm<br>167. Interviewee [XXX]: and do a culture, sensitivity of any particular system that's involved	**Experience and skill of the practitioner is vital in regards to detecting antibiotic misuse in pregnant women.**	**Experience and skill of the practitioner is vital in regard to detecting antibiotic misuse in pregnant women**	
110. Interviewer [MS]: Okay, are you aware of any pregnant women who take kind of herbal preparations or alternative medications in like in place of antibiotics?	Alternative Medication: Herbal Medication is used instead of antibiotics	Alternative Medication are substitute for antibiotics	Effects of Herbal Medication needs to be studied

111. Interviewee [XXX]: emm yes, they do take herbal medication on their own<br>112. Interviewer [MS]: In place of antibiotics?<br>113. Interviewee [XXX]: mmm Yes			[[4] HERBAL SELF-MEDICATION_KU
124. they go to their local herbalist there	Herbal medication is obtained from the local herbalist		
116. herbal medication in pregnancy for any reason eh some people for one reason or the other they might think talking about typhoid fever,<br><br>120. Interviewee [XXX]: People in the village still take medication or other 120. Interviewee [XXX]: People in the village still take medication or other things even mastitus mastitis they just take herbal medication	Herbal Medication universally taken for all types of health problem in pregnant woman	High popularity of alternative medication due to the belief that it can treat all health concerns	[[4] HERBAL SELF-MEDICATION_KU
122. Interviewee [XXX]: mmm a concoction, I don't know anything about it,	Ingredients of the herbal medications are unknown.	Alternative Medication is strongly discouraged as the effects has not been tested	

123. discourage them from that as you don't know the molecular,			
138. Interviewee [XXX]: mmm yes if it is possible. It will be difficult because the tests *unclear speech* take tests all day *unclear speech* antibiotics and<br><br>139. no one single test cannot detect all of them in terms of forensics and stuff like that	**Not keen about using Lab test/ rapid test, as they are time consuming. (Needs to be quick)**		Unsure about the use of Rapid Test<br><br>**[[5] DETECTING SELF-MEDICATION]**
188. Interviewee [XXX]: Forgetfullness Forgetfulness, em, oo not eh encountered patients with such eh in pregancy and linking it to antibiotics, ive not come across such complications	No reported side effect such as forgetfulness encountered in pregnant women taking antibiotics		No reported cases of Memory Loss<br><br>**[7] SIDE EFFECTS**
192. Interviewee [XXX]: No I used some data, some wifi or use cellular	Used data/Wi-Fi to connect		**[5] DETECTION SELF-MEDICATION_KU**_internet
198. Interviewee [XXX]: don't mind don't mind *unclear speech* im interested in the outcome not	Participant keen on the outcome of the project		

reinbursement *laughing*			
