# Supplementary material for: Advancing the safe motherhood initiative: A qualitative and sentiment analysis of local physician’s perspectives on antibiotic self-medication during pregnancy in a low- and middle-income country
Source: PLOS Glob Public Health. 2025 Sep 12;5(9):e0004794. doi: 10.1371/journal.pgph.0004794 (PMC12431270; doi:10.1371/journal.pgph.0004794)
Supplement: S1 File — Transcript 4 (CODES & THEMES by KU).pdf. Transcript 6 (CODES & THEMES by KU).pdf. Transcript 7 (CODES & THEMES, by KU).pdf. Transcript 8 (CODES & THEMES by KU).pdf. Transcript 9 (CODES & THEMES by KU).pdf. Transcript 10 (CODES & THEMES by KU).pdf. Transcript 11 (CODES & THEMES, by KU).pdf. Transcript 12 (CODES & THEMES by KU).pdf. Transcript 13 (CODES & THEMES by KU).pdf. Transcript 14 (CODED & THEMES by KU).pdf. Transcript 15_b (CODED & THEMES by KU). pdf. Transcript 16 (CODES & THEMES by KU).pdf. Transcript 17 (CODES & THEMES by KU).pdf. Transcript 18 (CODES & THEMES by KU).pdf. Transcript 19 (CODES & THEMES by HK).pdf. Transcript 20 (CODES & THEMES by HK).pdf. Transcript 21_b (CODES & THEMES by HK).pdfTranscript 22 (CODES & THEMES by HK).pdf. Transcript 25 (CODES & THEMES by HK).pdf. Transcript 27 (CODES & THEMES by HK).pdf. Transcript Sn1 (CODES & THEMES by RS).pdf Transcript Sn6 (pt3) (CODES & THEMES by RS).pdf. Transcript Sn15_a (CODES & THEMES by RS).pdf. Transcript SN17 (pt3) (CODES & THEMES by RS).pd. Transcript Sn21_a (CODES & THEMES by RS).pdf. (ZIP) [file pgph.0004794.s001.zip › Transcript Sn15_a (CODES & THEMES by RS).pdf]

Interview Transcript (Sn 15)	Initial Coding	Open Codes	Axial Codes
7. Interviewee [XXX]: *unclear speech* some logistic issues, some challenges with network<br>9. Interviewee [XXX]: Hello?<br>13. Interviewee [XXX]: Hello<br>22. Interviewer [MS]: So I need to go through this consent form can you see it on the screen? *no reply* Can you see this on the screen?<br>23. Principal Investigator [KU]: *participant name* Hello?<br>24. Interviewee [XXX]: Hello sorry there is poor network here I don't know<br>33. Interviewee [XXX]: Yes I can hear you now *unclear speech*<br>45. Interviewee [XXX]: ah ha let me *unclear speech<br>48. *line breaking up*	Disturbance during interview due to poor network connection	Network connection is unstable	**Virtual Interviews are challenging to conduct**

73. Principal Investigator [KU]: yes no carry on signal is so poor, so were quite lucky to get him eh so carry on with the questions<br>93. Interviewee [XXX]: Hello?<br>94. Interviewer [MS]: Hi<br>95. Interviewee [XXX]: Yes *overlap speech*<br>157. Interviewee [XXX]: if eh hello?<br>199. Interviewer [MS]: If someone had a reaction to antibiotics, is it clear? Is it obvious?<br>200. Interviewee [XXX]: Whether its clear *distorted speech* sorry I’m not following, I don’t understand the question.<br><br>214. Interviewee [XXX]: Can you come again with the question sorry *signal not very clear*<br>35. Interview			

ee [XXX]: I can see I wasn't hearing what you were saying before but I can hear you now			
107. Interviewee [XXX]: usually get it from the institution where I'm practising cause I work in the *unclear speech*, I work with the institution *unclear speech*, so when we prescribe and they have the antibiotics they get it from there<br>120. Interviewee [XXX]: *unclear speech* a good number of them. There are patients that go to *unclear speech* rural area.<br>121. Sometimes they will come tell you they<br>122. *unclear speech*<br>123. concoction<br>128. some of those herbal concoctions or leaves *unclear speech* have not been studied to know their pathological names or their	Accurate interpretation cannot be done due to unclear speech	**Misinterpretation / Inaccurate interpretation.**	

unclear speech names<br>134. Interviewee [XXX]: *unclear speech*, the only way I can identify that they are taking antibiotics on their own?<br>182. Interviewee [XXX]: urmm it depends it depends on yeah if yeah it will not likely be better because eh it might be it might be *unclear speech* for the pregnant woman, they may not be able to afford it just to screen them *unclear speech* general screening antibiotics not prescribed by doctors, the cost may be a lot for them and they might not be able to afford that *unclear speech*, theres not a lot in the villages *unclear speech*, it might not be cost effective general screening using			
21. Interviewee [XXX]: I didn't understand what you mean<br>54. then also the accent was a challenge	Participant finds it difficult to understand the question	**Language Barrier (tone, accent, interpretation)**	

65. Interviewee [XXX]: not quite clear with the accent *unclear speech* I didn't quite hear the last thing you said<br>66. Interviewer [MS]: pardon<br>67. Interviewee [XXX]: I didn't hear the last thing you said<br>109. Interviewer [MS]: Okay, do you know any pregnant women who have taken antibiotics that haven't been prescribed for them?<br>110. Interviewee [XXX]: Can I get the question clearer?<br>130. Interviewee [XXX]: Any way to detect?<br>131. Interviewer [MS]: If a woman has taken antibiotics without a prescription?<br>132. Interviewee [XXX]: If a woman is taking antibiotics, I didn't understand the question sorry			

159. Interviewer [MS]: Okay urm and then do you think that a questionnaire or a tool could be used in antenatal care, during appointments, or in A&E? that kind of environment?<br>160. Interviewee [XXX]: sorry can you come again<br>164. Interviewee [XXX]: Sorry can you come again<br>166. Interviewee [XXX]: Im not sure I understand what you mean, sorry<br><br>168. Interviewee [XXX]: *unclear speech*<br>187. Interviewee [XXX]:* unclear speech*<br>196. Interviewee [XXX]: *unclear speech*<br>220. Interviewee [XXX]: *unclear speech*<br>169. Interviewer [MS]: Mhmm<br>170. Interviewee [XXX]: I don’t understand the			

question, to detect how? Use of electricity?<br>174. Interviewee [XXX]: To do what sorry?<br>190. Interviewee [XXX]: Do I have any what sorry?<br>224. Interviewee [XXX]: I don't understand the question sorry			
11. Interviewee [XXX]: Okay I've not seen any information apart from the information asking me to join this meeting<br>15. Interviewee [XXX]: I said I have not seen any other information except for the one asking me to join this. Okay<br>35. Interviewee [XXX]: I can see I wasn't hearing what you were saying before but I can hear you now	Participant did not receive any information about the consent form beforehand	Technical Difficulties	
81. Interviewee [XXX]: *background noise*	Noise disturbance during the interview	Disturbance during interview	
17. Interviewee [XXX]: Okay I	Sense of urgency as the participant informs that they	**Busy Schedule**	Difficulty identifying if the

can go through it later<br>18. Interviewer [MS]: Okay, do you want to do it later? Yeah?<br>19. Interviewee [XXX]: Okay	will check the information sheet later.		participant is genuinely interested or just taking part in the study for the sake of it.
41. Interviewee [XXX]: the interview?<br>42. Principal Investigator [KU]: Yes, do you agree to take part?<br>43. Interviewee [XXX]: Okay this an interview now going on?<br>45. Interviewee [XXX]: ah ha let me *unclear speech*<br>46. of the interview, the aim<br><br>53. but then whats the focus of the interview, because I wasn't *unclear speech* when she was talking and<br><br>57. Interviewee [XXX]: About what<br>58. Interviewer [MS]: Antibiotics in antenatal pregnancy care	Unawareness of what the study is about	**Spontaneous acceptance to be a participant without knowing what the study is.**	

59. Interviewee [XXX]: Okay okay yeah antibiotics in antenatal patients<br><br>249. Interviewee [XXX]: okay yes the question I have, maam can I you said you sent me something which I wasn't able to read for this interview<br>250. Interviewer [MS]: mhmm<br>251. Interviewee [XXX]: so then I will still read it, I will still go through the message you sent me, was it send through whatsapp or through my mail, email<br>252. Interviewer [MS]: both it should be both, your whatsapp and your email<br>253. Interviewee [XXX]: okay so maybe when I go through it I will understand the whole essence of this exercise. Otherwise if you can just give me briefly the essence of the			

exercise and so on, if further things are expected from me in future<br><br>257. PI [KU]: *name of participant*, this is *name of Principal investigator*, eh so this project its just to gain a better understanding of the problem of antibiotic misuse eh during pregnancy and eh it's a collaboration between *name of university* and *name of hospital*<br>258. Interviewee [XXX]: yeah<br>259. PI [KU]: we've had liaisons with *name of doctor* and *name of doctor* and some other consultants at *name of hospital* and they've given permission for us to interview eh you and some of your colleagues on this topic<br>260. Interviewee [XXX]: okay			

50. Interviewee [XXX]: To take part in the interview?<br>52. Interviewee [XXX]: Yes<br><br>61. Interviewee [XXX]: yes Yes im ready<br>62. Interviewer [MS]: Yes okay and youre happy that im recording this<br>63. Interviewee [XXX]: Im okay<br><br>69. Interviewee [XXX]: I don't have to answer any of the questions<br>70. Interviewer [MS]: if you don't want to, if you want to stop the interview you can stop the interview you just have to let me know<br>71. Interviewee [XXX]: Okay	Verbal Consent received for the study		Verbal Consent
98. Interviewer [MS]: For pregnant women<br>99. Interviewee [XXX]: Okay ah most of them	Common Health problem for which antibiotic is prescribed for is for Infection	Infection	Antibiotic Prescription for **[1_PRESCRIBING]**

when they have infections<br>100. Interviewer [MS]: mhmm<br>101. Interviewee [XXX]: infections of the genital tracts<br><br>103. Interviewee [XXX]: or infections any other place okay? Outside the genital tracts that the man said that they should be given antibiotics<br>104. Interviewer [MS]: mhmm mhmm okay<br>105. Interviewee [XXX]: Yeah	Unable to recognise what the speech means.		
106. Interviewer [MS]: Where do you, where do pregnant women normally get their antibiotics from?<br>107. Interviewee [XXX]: usually get it from the institution where I'm practising cause I work in the *unclear speech*, I work with the institution *unclear speech*, so when we prescribe and they have the	Legalised prescription of antibiotics in hospital and pharmacy.	**Juxtaposition of healthcare system. Unregulated use of antibiotics**	**Antibiotics has not been recognised as a priority in healthcare [1_PRESCRIBING]**

antibiotics they get it from there.<br><br>108. If they don't have it within the facility where I am working, then they normally go they go across to nearby pharmacy shops and procure them			[3_SELF-MEDICATION/ MISUSE]
116. Interviewer [MS]: Where do they normally get them from?<br>117. Interviewee [XXX]: Usually they get it across the counter, from chemist shops, from pharmacy shops<br><br>118. some from traders in the market that stop and sell antibiotics	Un-prescribed medication are also found locally in pharmacy or market		
112. Interviewee [XXX]: Okay any women that has taken antibiotics not prescribed<br>113. Interviewer [MS]: Yeah<br>114. Interviewee [XXX]: Yes, there are several of them<br><br>115. and occasionally we	Reported cases of antibiotic misuse among pregnant women	**Antibiotic misuse is common among pregnant women.**	[6_GUIDELINES]

come reports that they take antibiotics *unclear speech*, antibiotics that were not prescribed by doctors, by any doctors			
134. Interviewee [XXX]: *unclear speech*, the only way I can identify that they are taking antibiotics on their own?<br>135. Interviewer [MS]: Mhmm<br>136. Interviewee [XXX]: Theres no way,<br><br>144. otherwise theres no other way we can find out *unclear speech*<br>191. Interviewer [MS]: Guidelines that kind of help detect the side effects of antibiotic self medication in pregnant women<br>192. Interviewee [XXX]: Okay not I don't have any for now<br><br>212. Interviewee [XXX]: no I	**No specific method of detecting self-medication of antibiotic among pregnant women identified**	**A proper and systematic guideline for safe antibiotic use in pregnancy not implemented**	

don't know any sorry			
119. Interviewer [MS]: mhmm okay. Do you know of any pregnant women who sometimes take herbal preparations or alternative medications instead of antibiotics?<br>120. Interviewee [XXX]: *unclear speech* a good number of them. There are patients that go to *unclear speech* rural area.	Larger population among rural community consume herbal medication instead of antibiotics	**Herbal Medication is popular in rural community**	**Effects of herbal medication needs to be studied [4_HERBAL SELF-MEDICATION]**
121. Sometimes they will come tell you they<br>122. *unclear speech*<br>123. concoction	History of consumption of herbal medication when patients come to the hospital.	**Consumption of herbal medication for infection**	
124. Interviewer [MS]: mhmm<br>125. Interviewee [XXX]: for possible infection, for suspected possible infections	Pregnant women consume herbal medication when they suspect infection		
127. Interviewee [XXX]: Um they just have some local herbs that	Ingredients of the herbal	**Potential risk of herbal medication has**	

do not have *unclear speech* biological, I mean urm how do you call it now, that we don't know the name but the local villages where they get the herbal concoction, they have their name,<br><br>128. some of those herbal concoctions or leaves *unclear speech* have not been studied to know their pathological names or their *unclear speech* names	medication are not known	**not been studied**	
138. if you ask them if they are taking any medication outside *unclear speech*<br><br>142. When we ask questions with regard to medications in the pregnancy sometimes they own up<br><br>144. otherwise theres no other way we can find out *unclear speech*<br>150. Interviewee [XXX]: Yeah I ask them when we ask them we find out	**History about antibiotic misuse can help detect potential women who take antibiotics without prescription.**	**History taking skills of the practitioner is important**	**Crucial role of the practitioner in order to identify pregnant woman who have misused antibiotics [5_DETECTING SELF-MEDICATION]**

155. Interviewee [XXX]: especially when they know youre not going to use that against them	Confidentiality must be maintained so that the women feel safe if they were to be honest about their antibiotic misuse.	A practitioner must have good rapport building skill for the patients to open up about the misuse of antibiotic	
153. a good number of them will tell you the truth	**Trust must be gain among the pregnant women who have misused antibiotics, inorder for them to tell the truth about the misuse,**		
139. and some of them will own up and say yeah they did, or<br><br>142. When we ask questions with regard to medications in the pregnancy sometimes they own up<br>143. and divulge information	Pregnant women who misuse antibiotics are reluctant to confess about their misuse of the medication.	Guilt is associated with misuse of antibiotic. Hence, it is important to build trust.	
201. Interviewer [MS]: Is it obvious if you see someone, a pregnant woman that's having side effects to antibiotics? Is it obvious that there having side effects?<br>202. Interviewee [XXX]: Okay its not always	Hard to specify which medication has caused side effects during pregnancy	**Adversities of antibiotics misuse are not easily identified**<br><br>**Hence, the role of a physician is important**	

obvious *unclear speech* its not very obvious as such eh some of them may have the antibiotics *unclear speech* after delivery some may not be very obvious<br><br>208. Interviewee [XXX]: em theres some women actually that we know that use drugs in early pregnancy and they have malformed fetuses babies delivered preterm or sometimes they have miscarriage and then some obvious abnormality<br>209. Interviewer [MS]: Mhmm *overlap*<br>210. Interviewee [XXX]: but then its difficult to pin down on the real ~~course~~ **[CAUSE]** of this is result of antibiotics		**inorder to identify potential people who have misused antibiotics**	**[7_SIDE EFFECTS]**
264. Interviewee [XXX]: okay thank you very much and will there be a	**Participant is interested in the project and would like to be part of it**		

publication on this?<br>268. Interviewee [XXX]: okay, its alright, thank you very much for the opportunity and em we hope we'll be part of the project of *unclear speech*			
137. maybe when they if they if they come and they are complaining of infection *unclear speech*,<br><br>141. Interviewee [XXX]: maybe when they have problems or some complications in their pregnancy we begin to wonder why.	**Pregnant women who misuse antibiotics complain of infection when presented**<br><br>**Antibiotic misuse can be detected when doctors must find differential diagnosis for the complications presented in pregnant women**	**Potential side effect which can be detected by the practitioner**	
206. Interviewee [XXX]: ummmm, eh none that I can remember now because they isn't aware of specifically em *unclear speech*, the antibiotics that have caused the problem	**No recognised cases of side effects as a result of antibiotic misuse among pregnant woman**	**Side effects are rarely noted in pregnant women who misuse antibiotics**	**[7_SIDE EFFECTS]**

152. Interviewee [XXX]: yeah we can have a questionnaire ask them,<br>158. Interviewer [MS]: Would you be interested in using such a questionnaire if it was available? Interviewee [XXX]: Yes I would<br>162. Interviewee [XXX]: Yeah I think I can be useful, can be useful *unclear speech* , subsequently *unclear speech*	Positive response towards using a questionnaire that can help identify women who misuse antibiotics.	**A questionnaire could be beneficial in clinical setting for detecting misuse**	**Affordability needs to be considered when analysing potential tests that can be used to detect antibiotic misuse**<br><br>**[5_DETECTING SELF-MEDICATION]**
180. Interviewee [XXX]: Chemical analysis to detect them<br>181. Interviewer [MS]: Mhm<br>182. Interviewee [XXX]: urmm it depends it depends on yeah if yeah it will not likely be better because eh it might be it might be *unclear speech* for the pregnant woman, they may not be able to afford it just to screen them *unclear speech* general screening antibiotics not	Test (Screening Test) to detect antibiotic misuse is not convenient because it will be expensive for the patients.	Screening Test is not suitable	

prescribed by doctors, the cost may be a lot for them and they might not be able to afford that *unclear speech*, theres not a lot in the villages *unclear speech*, it might not be cost effective general screening using			
183. maybe a chemical analysis<br>184. Interviewer [MS]: okay<br>185. Interviewee [XXX]: from their blood or urine<br>186. Interviewer [MS]: okay thank you	Test using samples from blood or urine can be more cost effective instead of screening test for detecting antibiotic misuse in pregnant women		
215. Interviewer [MS]: so so if some pregnant women if they take like their antibiotics without them being prescribed, sometimes they might get memory loss or forgetfullness, have you ever come across that, do you know how to manage that, have you ever seen that?<br>216. Interviewee [XXX]: eh not	**No cases of memory loss associated with antibiotic use during pregnancy.**		Memory Loss<br>**[7_SIDE EFFECTS]**

come across that in my practice			
83. Interviewee [XXX]: Yes I do and *unclear speech* I give yes<br>84. Interviewer [MS]: Okay. How long have you been prescribing antibiotics for?<br>85. Interviewee [XXX]: emm as long as I can remember, since I graduated *unclear speech*, over over 15 years now	Years qualified to prescribe antibiotics.		**[1_PRESCRIBING]**
87. Interviewee [XXX]: yeah<br>88. Interviewer [MS]: How many times a week do you prescribe antibiotics….. to pregnant women?<br>89. Interviewee [XXX]: ummm on average be about around 3 times in a week<br>90. Interviewer [MS]: Okay<br>91. Interviewee [XXX]: On average emm	Times per week when antibiotic prescribed		
97. Interviewee [XXX]: For	Clarification of the question asked		

women? Generally? Or for pregnant women?			
205. Interviewer [MS]: Okay. Do you know any pregnant women who have had side effects of self-medication of antibiotics? So if they've taken it themselves without a prescription?<br>206. Interviewee [XXX]: ummmm, eh none that I can remember now because they isn't aware of specifically em *unclear speech*, the antibiotics that have caused the problem	**No recognised cases of side effects as a result of antibiotic misuse among pregnant woman**		**[7_SIDE EFFECTS]**
225. Interviewer [MS]: have you bought an airtime card?<br>226. Interviewee [XXX]: I have got one<br>227. Interviewer [MS]: yeah so you can submit *name of a doctor* for a refund for taking part after the interview	Use of airtime for call		Airtime

228. Interviewee [XXX]: okay *name of a doctor*<br>229. Interviewer [MS]: yeah<br>230. Interviewee [XXX]: *unclear overlapping speech*<br>231. Interviewer [MS]: so you can submit forward the airtime card to him and he will be able to give you a refund<br>232. Interviewee [XXX]: okay so I can send some airtime to *name of a doctor*<br>233. Interviewer [MS]: * started to speak then stopped*<br>234. PI [KU]: So *name of participant* shes referring to the airtime eh that you use for this interview. Did you buy a card for this interview?<br>235. Interviewee [XXX]: okay yes I did<br>236. PI [KU]: okay so you could send that card to *name of a doctor*			

237. Interviewee [XXX]: okay the card?<br>238. Interviewer [MS]: yeah<br>239. PI [KU]: yeah, the airtime card			
