# Supplementary material for: Advancing the safe motherhood initiative: A qualitative and sentiment analysis of local physician’s perspectives on antibiotic self-medication during pregnancy in a low- and middle-income country
Source: PLOS Glob Public Health. 2025 Sep 12;5(9):e0004794. doi: 10.1371/journal.pgph.0004794 (PMC12431270; doi:10.1371/journal.pgph.0004794)
Supplement: S1 File — Transcript 4 (CODES & THEMES by KU).pdf. Transcript 6 (CODES & THEMES by KU).pdf. Transcript 7 (CODES & THEMES, by KU).pdf. Transcript 8 (CODES & THEMES by KU).pdf. Transcript 9 (CODES & THEMES by KU).pdf. Transcript 10 (CODES & THEMES by KU).pdf. Transcript 11 (CODES & THEMES, by KU).pdf. Transcript 12 (CODES & THEMES by KU).pdf. Transcript 13 (CODES & THEMES by KU).pdf. Transcript 14 (CODED & THEMES by KU).pdf. Transcript 15_b (CODED & THEMES by KU). pdf. Transcript 16 (CODES & THEMES by KU).pdf. Transcript 17 (CODES & THEMES by KU).pdf. Transcript 18 (CODES & THEMES by KU).pdf. Transcript 19 (CODES & THEMES by HK).pdf. Transcript 20 (CODES & THEMES by HK).pdf. Transcript 21_b (CODES & THEMES by HK).pdfTranscript 22 (CODES & THEMES by HK).pdf. Transcript 25 (CODES & THEMES by HK).pdf. Transcript 27 (CODES & THEMES by HK).pdf. Transcript Sn1 (CODES & THEMES by RS).pdf Transcript Sn6 (pt3) (CODES & THEMES by RS).pdf. Transcript Sn15_a (CODES & THEMES by RS).pdf. Transcript SN17 (pt3) (CODES & THEMES by RS).pd. Transcript Sn21_a (CODES & THEMES by RS).pdf. (ZIP) [file pgph.0004794.s001.zip › Transcript SN17 (pt3) (CODES & THEMES by RS).pdf]

Interview Transcript (Sn17)	Initial Coding	Open Codes	Axial Codes
5. Interviewee [XXX]: okay you can go on (hard to make out end of sentence)<br><br>74. *unclear sounds like 75*<br><br>81. *hard to make out rest of sentence*<br><br>85. Interviewee [XXX]: * said something, unclear*<br><br>96. *unclear speech*,<br><br>116. *hard to make out ?<br><br>137. Interviewee [XXX]: Please repeat yourself please	- Difficulty interpretating/ understanding what the interviewee is saying	Language Barrier (intonation, pronunciations , accents)	Virtual Interviews are difficult to perform
146. Interviewer [MS]: Yeah okay urm and then do you know I mean similar to a question we talked about before, but do you know of any guidelines or protocols that kind of look at antibiotic self-medication in pregnant women, the side effects of that?<br>147. Interviewee [XXX]: Come again please	Interviewee finding it difficult to understand the question	Lengthier questions are harder to understand	

60. Interviewee [XXX]: Maybe I can't understand what you mean by that *unable to make out next part of sentence/overlapping speaking*			
7. Interviewee [XXX]: Hello?<br>9. Interviewee [XXX]: Yes I can see, no screen sharing already what I am seeing is just *name mentioned*, I no screen yet<br>14. Principal Investigator [KU]: Right yes its visible now<br>21. Interviewer [MS]: Can you see this consent form?<br>22. Interviewee [XXX]: Its no longer showing, okay now yes	Difficultly to navigate in zoom.	Technological Difficulties	
19. Interviewee [XXX]: okay<br>20. Principle Investigator [KU]: Okay. *name of RA* I think you can go through the consent form er now because I think *name mentioned* has limited time erm	Rushing through the consent form	Busy Schedule, participant might want to rush through it	
76. *sound not as loud, hard to hear*<br><br>79. *hard to make out, sound unclear*	Disturbance due to poor network connection	Internet Connection Issues	

154. *unable to hear properly*<br><br>160. *background noise/muffled noise noted*<br><br>161. Interviewee [XXX]: *unclear speech*			
16. Principal Investigator [KU]: So *name of participant*, this is just information about the study err just to make you aware err of the study. I mean *name of RA* would have sent this to you via email erm<br>17. Interviewee [XXX]: Yeah<br><br>25. Interviewee [XXX]: I've gone through all that<br>26. Interviewer [MS]: Pardon?<br>27. Interviewee [XXX]: I've gone through that, through my mail<br>28. Interviewer [MS]: Okay so have you had a read of it? Have you read the consent form?<br>29. Interviewee [XXX]: Okay, you say I should read it?<br>30. Interviewer [MS]: Or you can have, im just going to if you just have a quick read of the statements and then I can just put your initials urm and then that's fine	A proper consent cannot be established	Virtual consent taking is time consuming	

31. Interviewee [XXX]: I say I have read through this<br>32. Interviewer [MS]: Okay<br>33. Interviewee [XXX]: I’ve read through this, I've read through<br>34. Interviewer [MS]: Okay<br>35. Interviewee [XXX]: Yes, just to appoint my signature<br>42. Interviewer [MS]: So you’re happy for me to sign this? Yeah?<br>43. Interviewee [XXX]: Yes<br>46. Principal Investigator [KU]: So *initials of participant* all the way through this<br>47. Interviewee [XXX]:Yes			
69. Interviewee [XXX]: umm pre surgery post surgery ur<br><br>83. Interviewee [XXX]: mainly post surgical, post caesarean delivery and all that	Common health problem for which antibiotic are prescribed for	Surgical	Common health problems for which Antibiotics are prescription<br><br>**[1_PRESCRIBING]**
71. infections urm sexually transmitted um infections and um rest of it	Common health problem for which antibiotic are prescribed for	Reproductive	
89. Interviewee [XXX]: Well urinary tract infections in pregnancy, yes<br>90. Interviewer [MS]: Okay<br>91. Interviewee [XXX]: Mainly	Common disease/health problem for which antibiotics are prescribed for?	Urology	

93. Interviewee [XXX]: mmmmm I wouldn’t say there are really existing guidelines per say um so yes<br>163. Interviewee [XXX]: No no we are not used to guidelines	No official guidelines followed when antibiotics prescribed.	A proper and systematic guideline for safe antibiotic use in pregnancy not implemented.	Antibiotics has not been recognised as a priority in healthcare<br>**[1_PRESCRIBING]**
103. Interviewee [XXX]: Well yess, eh because of eh none availability of guidelines	Unrestricted access to antibiotics due to no proper guidelines	Unregulated antibiotic distribution and use	**[2_OBTAINING]**
104. some of them can over the counter eh can get from chemist without prescription	Availability of non-prescribed antibiotics through chemist and over the counter.		
121. Interviewee [XXX]: How do they identity them?<br>123. Interviewer [MS]: Yeah, do you have something like that?<br>124. Interviewee [XXX]: No no im not aware of anything<br><br>126. no not necessarily from any known tests<br><br>148. Interviewer [MS]: Do you know of any guidelines or urm side effects of antibiotic misuse in pregnant women?	No known recognised protocols which can identify antibiotic misuse in pregnant woman.	Detection of antibiotic misuse not implemented	**[5_DETECTING MISUSE]**

Interviewee [XXX]: mmm not that I'm aware of			[1_OBTAINING]
94. Interviewer [MS]: Okay, so where do pregnant women normally get antibiotics from? From the pharmacy or health centres?<br>95. Interviewee [XXX]: Well often from pharmacy, pharmacy within the hospital , pharmacy<br>100. Interviewer [MS]: That's generally where they go when they have a prescription? They go to the pharmacy or somewhere inside or outside the hospital<br>101. Interviewee [XXX]: yes yes	Primarily, prescription of antibiotics is given from Hospital and Pharmacy	Duality of health care facilities providing antibiotics	
104. some of them can over the counter eh can get from chemist without prescription	Availability of non-prescribed antibiotics through chemist and over the counter.		
106. Interviewee [XXX]: whether I know if any herbal medications that work as much as antibiotics?	No known positive effects of alternative medication in comparison to antibiotics.		Antibiotics are not readily available? / not

107. Well I don’t know<br><br>but I know that pregnant women consume herbal medications and em my collegue did a study on that and found it is really common<br><br>115. Interviewee [XXX]: some of them do but if I use the word abuse	Herbal medications are commonly used as alternative source to antibiotics	Home-remedy (alternative to antibiotics, but why?)	affordable? Remote areas do not have access to antibiotics. Hence use, herbal medication?<br><br>**[4_HERBAL SELF-MEDICATION]**
109. Interviewee [XXX]: *starting to speak at the same time* I don’t know the names of any particular I don’t know	Medical professions donot know the ingredients in herbal medication		
112. Interviewer [MS]: Yeah like people that are taking alternative medications<br>113. Interviewee [XXX]: We urmm If I say a lot I really cannot quantify but eh we do come across them very so occasionally when we probe it that is where you really enquire<br><br>125. they just from eh questioning you get information<br><br>149. Interviewer [MS]: Mhmm so its more from like experience or	Encounter of alternative medication user when probed with questions/ investigation	Experience and skill of the practitioner is vital in regard to detecting antibiotic misuse in pregnant women	Improving interpersonal communication skills in health professionals and building trust is a crucial step in-order to identify misuse of antibiotic in pregnant woman

150. Interviewee [XXX]: Yeah			**[5_DETECTING] _ROLE OF PRACTITIONER**
117. if abuse*, so I wouldn't know so they take them secretly<br>118. Interviewer [MS]: mmm so they don't always tell you<br>119. Interviewee [XXX]:No they don't	Patients do not wish to disclose about the antibiotic misuse/ use of alternative medication when they come to hospital.	Building trust and safe environment for pregnant woman instead of making them feel ashamed for misusing antibiotics necessary	
128. Interviewee [XXX]: A questionnaire eh yes,<br><br>131. Interviewee [XXX]: Questionnaire, yes<br><br>132. Interviewer [MS]: So if something was avaliable like that you would be interested in using it?<br>133. Interviewee [XXX]: It is is avaliable whether I would be interested in using it, yeah<br><br>134. Interviewer [MS]: Yeah, so a questionnaire rather than kind of like eh test like a rapid test<br>135. Interviewee [XXX]: Yeah if its available we will use	Positive response towards using a questionnaire that could help identify misuse of antibiotics in pregnant woman	A questionnaire could be beneficial in clinical setting for detecting misuse	A versatile questionnaire developed with the help of the experiences of health care professional could be beneficial for detecting antibiotics misuse.<br><br>**[5_DETECTING]**
139. Interviewee [XXX]: Yes *overlap*<br>140. Interviewer [MS]: So where do you think it would be	Questionnaires are versatile. Can be used in all settings	Features : Versatility of the	

most useful? Do you think it could be used in all those places?<br>141. Interviewee [XXX]: It could be used in all those places. Antenatal ward, labour ward		questionnaires is desirable	**[5_DETECTING]**
113. Interviewee [XXX]: We urmm If I say a lot I really cannot quantify but eh we do come across them very so occasionally when we probe it that is where you really enquire<br><br>149. Interviewer [MS]: Mhmm so its more from like experience or<br>150. Interviewee [XXX]: Yeah	Presently, skills and experience of the practitioner used to help identify women who are potentially misusing antibiotics	Skills and experience helps in detecting antibiotic misuse	
175. Interviewee [XXX]: *unclear speech* its alright. I wish you would design studys that will involve us<br>176. Principal Investigator [KU]: yes we are planning towards that and this is just the first step in *unclear* project so<br>177. Interviewee [XXX]: Okay<br><br>178. Principal Investigator [KU]: Were just trying to work out a method of working together and sharing information so this is just a first step in a number of future	Participant's interest towards being part of this study.	Clinicians interest in the study	

projects eh that will involve *name of hospital* and Liverpool John Moores definitely<br>179. Interviewee [XXX]: Okay okay thank you very much			
142. Interviewer [MS]: Mhmm, so I mean obviously you were saying you would prefer like a questionnaire rather than something medical like a test, but do you think if there was a test it would need to be mobile or easy to carry around like not having to use electrical power or internet, you know something that is very accessible to everyone<br>143. Interviewee [XXX]: Yeah<br>144. Interviewer [MS]: Do you think that would be more useful?<br>145. Interviewee [XXX]: Yes	Leading Questions??)<br><br>Portability of the potential test is desirable. The device that can run without the requirement of electricity.	Agrees to the idea of rapid test which is mobile, portable and can run without electricity	**[5_DETECTING]**
151. Interviewer [MS]: Fine, so have you seen you know generally when a woman takes antibiotics they can have side effects like stomach upset or not feeling well um do you think generally when someone is having side effects from antibiotics that	Side effects of antibiotics are not easily identified/ detectable	Adversities of antibiotics misuse are not easily identified	**[7_SIDE EFFECTS]**

its quite clear from the antibiotics<br>152. Interviewee [XXX]: No, often there is not very certain.			
153. Occasionally nausea that's common, besides that I would not be able to they could			
156. Interviewee [XXX]: Mmmm many years ago actually I I saw something that looked like looked like an allergic reaction to antibiotic medication, I think that person had some *unclear* allergy and some bronchospasms<br>158. Interviewee [XXX]: * at the same time* - from medication and they are pregnant woman yes	Misuse of antibiotics caused allergic reaction and respiratory problem in pregnant women.		Awareness Campaign among general population about the harmful effects of misuse of antibiotics can help reduce the population misusing antibiotics.
117. if abuse*, so I wouldn't know so they take them secretly<br>118. Interviewer [MS]: mmm so they don't always tell you<br>119. Interviewee [XXX]:No they don't	Patients do not wish to disclose about the antibiotic misuse/ use of alternative medication when they come to hospital.	Patients are ashamed about their misuse of antibiotics	**[7_SIDE EFFECTS]**
107. Well I don't know<br><br>but I know that pregnant women consume herbal medications and em my collegue did a study on that and	Herbal medications are commonly used as alternative source to antibiotics	Why do they use herbal remedy instead? Are they ashamed about having a	**[4_HERBAL SELF-MEDICATION]**

found it is really common		disease? Or is medication expensive?	
