# Supplementary material for: Advancing the safe motherhood initiative: A qualitative and sentiment analysis of local physician’s perspectives on antibiotic self-medication during pregnancy in a low- and middle-income country
Source: PLOS Glob Public Health. 2025 Sep 12;5(9):e0004794. doi: 10.1371/journal.pgph.0004794 (PMC12431270; doi:10.1371/journal.pgph.0004794)
Supplement: S1 File — Transcript 4 (CODES & THEMES by KU).pdf. Transcript 6 (CODES & THEMES by KU).pdf. Transcript 7 (CODES & THEMES, by KU).pdf. Transcript 8 (CODES & THEMES by KU).pdf. Transcript 9 (CODES & THEMES by KU).pdf. Transcript 10 (CODES & THEMES by KU).pdf. Transcript 11 (CODES & THEMES, by KU).pdf. Transcript 12 (CODES & THEMES by KU).pdf. Transcript 13 (CODES & THEMES by KU).pdf. Transcript 14 (CODED & THEMES by KU).pdf. Transcript 15_b (CODED & THEMES by KU). pdf. Transcript 16 (CODES & THEMES by KU).pdf. Transcript 17 (CODES & THEMES by KU).pdf. Transcript 18 (CODES & THEMES by KU).pdf. Transcript 19 (CODES & THEMES by HK).pdf. Transcript 20 (CODES & THEMES by HK).pdf. Transcript 21_b (CODES & THEMES by HK).pdfTranscript 22 (CODES & THEMES by HK).pdf. Transcript 25 (CODES & THEMES by HK).pdf. Transcript 27 (CODES & THEMES by HK).pdf. Transcript Sn1 (CODES & THEMES by RS).pdf Transcript Sn6 (pt3) (CODES & THEMES by RS).pdf. Transcript Sn15_a (CODES & THEMES by RS).pdf. Transcript SN17 (pt3) (CODES & THEMES by RS).pd. Transcript Sn21_a (CODES & THEMES by RS).pdf. (ZIP) [file pgph.0004794.s001.zip › Transcript Sn21_a (CODES & THEMES by RS).pdf]

Interview Transcript (Sn 21)	Initial Coding	Open Codes	Axial Codes
2. Interviewee [XXX]: Okay, no no I didn't have time, I didn't see it<br><br>8. Interviewee [XXX]: I will read it after	Busy schedule -> couldn't read form beforehand.	Busy Schedule	
59. Interviewee [XXX]: mmmmm.... do you hear me *distorted sound*<br>60. Interviewer [MS]: Yeah the signal just went abit then. That's great<br><br>65. *distorted sound*<br><br>75. Interviewee [XXX]: *unclear speech*<br>76. Interviewer [MS]: *slight laugh* can you hear me?<br>77. Interviewee [XXX]: I didn't hear you<br><br>81. Interviewee [XXX]: They get them from the p *cuts off*<br><br>83. Interviewee [XXX]: *bad signal, inaudible speech*<br>84. Interviewer [MS]: Where sor sorry the signals not good	Disruption due to unstable Internet connection.	Unstable Internet Connection	Virtual Interview are challenging to conduct

85. Interviewee [XXX]: mm somebodies trying to call me, that’s why the interruption<br>86. Interviewer [MS]: * slight giggle* *pause* okay<br>87. Interviewee [XXX]: Did you hear me?<br><br>130. Interviewee [XXX]: *background noise*	**Disturbance during interview**		
4. Interviewee [XXX]: yes I can see<br>5. Interviewer [MS]: okay so this is just a consent form for the study, em so what im I’m just going to go through it with you quite quickly if you’ve got any questions let me know, im I’m just going to put your initials in, and if youre you’re happy to consent for the study then we can start, is that okay?<br>6. Interviewee [XXX]: it's okay	**Addressing Consent form**		**Consent Form**
11. Interviewee [XXX]: its okay<br>12. Interviewer [MS]: do you consent to take part in the study and you understand that you can refuse to answer questions or	**Verbal Consent given**		

withdraw from the study at anytime?<br>13. Interviewee [XXX]: its okay I understand<br>14. Interviewer [MS]: yeah perfect. So you understand any potential risks with taking part in the study, information will be on the information sheet about that, but again if you want to stop the study at all then you would just let us know<br>15. Interviewee [XXX]: okay<br>16. Interviewer [MS]: okay, so are you happy for the interview to be audio and video recorded?<br>17. Interviewee [XXX]: yes<br>18. Interviewer [MS]: yes, so if you want to turn off your video at any time then you can, you don't have to keep it on, its completely up to you. Okay?<br>19. Interviewee [XXX]: okay yes<br>20. Interviewer [MS]: you understand who will have access to your personal data, so that will be the research team and how the data will be stored and what will happen to the data at the end of the			

project. So at the end of the project, you know we will go through the data, hopefully write a report about the data<br>21. Interviewee [XXX]: It's alright<br>22. Interviewer [MS]: yeah. Urm you understand your personal data will remain confidential and every effort will be made to make sure that you are not identified in any reports or future outputs of the study<br>23. Interviewee [XXX]: its alright I understand<br>24. Interviewer [MS]: perfect and you understand that interview recordings may be used in the final report or any further outputs<br>25. Interviewee [XXX]: yes<br>26. Interviewer [MS]: yep and you understand that parts of the conversation will be used in future publications or presentations, but every effort will be made to make sure you're not identified in that. Okay<br>27. Interviewee [XXX]: yes<br>28. Interviewer [MS]: and you agree to take part in the study?			

29. Interviewee [XXX]: yes<br>30. Interviewer [MS]: perfect so im I’m just going to put, that’s your initials in there right? *asked if initials correct*<br>31. Interviewee [XXX]: *reading outloud*<br>32. Interviewer [MS]: Is that your initials?<br>33. Interviewee [XXX]: * says initials*<br>34. Interviewee [XXX]: *confirming initials*<br>35. Interviewer [MS]: okay ill change that perfect, ill I’ll just do this quickly. Perfect and that’s your name and this is the date today, perfect so we can start the interview now<br>36. Interviewee [XXX]: okay			
51. Interviewer [MS]: Perfect *overlap with interviewee* and what are the most, what are the most common problems which you prescribe antibiotics for?<br>52. Interviewee [XXX]: upper respiratory tract infection	Respiratory (Infection)	Common health problems for which antibiotic is prescribed for	Antibiotic Prescription for **[1_PRESCRIBING]**
54. Interviewee [XXX]: Urinary tract infection,	Urinary (Infection)		
55. eeehh preterm rom	Pregnancy		

56. Interviewer [MS]: mhmm<br>57. Interviewee [XXX]: preterm premature rupture of membrane			
62. post operative antibiotics	Preventive (Post operative)		
63. Interviewer [MS]: okay, so do you have any guidelines that you use when you prescribe antibiotics?<br>64. Interviewee [XXX]: yes	**Guidelines are used when prescribing antibiotic.**	**No known available guideline when prescribing antibiotics**	**[6] A proper and systematic guideline for safe antibiotic use in pregnancy not implemented**<br><br>**[6_GUIDELINES]**
67. Interviewee [XXX]: guidelines, yes sometimes we have eh sensitivity,	**Interprets that sensitivity test is a type of guideline**		
68. but there is no major guideline but	**No major guidelines available when prescribing antibiotics**		
69. eh but what guides our what guides my choice of antibiotic depends on the particular element like preterm prom we have a guideline, apart from preterm prom we don't have any other guideline	**Guidelines available only when there is risk of complication during pregnancy**		
120. Interviewer [MS]: Mhm, that would be most useful, perfect. Um Have you come across any guidelines or methods that help	**No tools available that can recognise side effect of misused antibiotics**	No known tool that can detect side effect of antibiotic misuse	

detect side effects when women take antibiotics without being prescribed?<br>121. Interviewee [XXX]: No			
131. To manage self, okay no no I don't have any	**No recognised guidelines available when managing antibiotic self-medication**	No proper guidelines available	
71. Interviewee [XXX]: From the pharmacy<br>73. Interviewee [XXX]: Hospital pharmacy	**Prescribed antibiotics from hospital pharmacy**	Legal prescription of antibiotics in hospital	**[1] Antibiotic distribution**<br><br>**[2_OBTAINING]**
89. Interviewee [XXX]: They get them from the you know here in Nigeria they patent medicine<br>90. Interviewer [MS]: *mhmm*<br>91. Interviewee [XXX]: dealers can, can they sell some of them can sell antibiotics<br>92. Interviewer [MS]: Okay<br>93. Interviewee [XXX]: So they get them from patent medicine dealers	**Illegal selling of antibiotics from dealers**	Unregulated selling of antibiotics	
78. Interviewer [MS]: Okay, do you often, have you ever come across women who are taking antibiotics that havent been prescribed for them?<br>79. Interviewee [XXX]: Yeah, yes. I have	**Encounter of women who have misused antibiotics**		

<table>
<tr><td></td><td></td><td></td><td></td></tr>
<tr><td>122. Interviewer [MS]: No? Perfect. So you know anyway antibiotics can cause side effects um is it clear when a woman is having side effects to antibiotics?
123. Interviewee [XXX]: Its not usually clear</td><td>Side effects of antibiotic is not very clear.</td><td rowspan="2">Adversities of antibiotics misuse are not easily identified

Hence, the role of a physician is important inorder to identify potential people who have misused antibiotics</td><td rowspan="4">[7] Crucial role of the <u>practitioner</u> in order to identify pregnant woman who have misused antibiotics

[5_DETECTING]</td></tr>
<tr><td>126. Interviewer [MS]: Okay so have you ever had experience of a pregnant woman having side effects after self medicating with antibiotics that werent weren't prescribed?
127. Interviewee [XXX]: I can't remember</td><td>Hasn't encountered with patients who have presented with side effects of antibiotic self-medication</td></tr>
<tr><td>102. Interviewee [XXX]: its just from history

105. Interviewer [MS]: Perfect, so just when you ask them?

106. Interviewee [XXX]: Yeah</td><td>History Taking is key way of identifying women who have misused antibiotics</td><td rowspan="2">Skills and Experience of practitioner is vital in identifying women who have misused antibiotics</td></tr>
<tr><td>124. Interviewer [MS]: So how would you know? How would you know?
125. Interviewee [XXX]: well if you have experience with that particular antibiotic *unclear speech*</td><td>Practitioner's experience is important when detecting side effects of antibiotic misuse</td></tr>
</table>

94. Interviewer [MS]: Okay so are you aware of any pregnant women that might take like herbal preparations or alternative medications that kind of work like antibiotics?<br>95. Interviewee [XXX]: Yes<br><br>100. but I know they take herbal medications	**Encounter of women who take herbal/alternative medication**		[4] Herbal medications are used<br><br>**[4_HERBAL SELF-MEDICATION]**
98. Interviewer [MS]: Like alternative medications or herbal preparations that they might take instead of antibiotics?<br>99. Interviewee [XXX]: No I don't have, I don't know their names	Do not know the name of the alternative medication		
109. Interviewer [MS]: Do you think that would be useful to have that?<br>110. Interviewee [XXX]: yeah it will, it will be useful<br><br>111. Interviewer [MS]: Would you be interested in using a tool like that if it was available?<br>112. Interviewee [XXX]: Yes	Positive response towards using a test to identify misuse of antibiotic		[5] Tool/ Rapid Test could be beneficial in clinical setting for detecting misuse<br><br>**[5_DETECTING]**
113. Interviewer [MS]: Perfect, so just say we did have a tool like that um	Best use for the tool would be in antenatal setting		

do you think it would be useful in in like antenatal care settings, or routine appointments or A&E? Where do you think it would be most useful?<br>114. Interviewee [XXX]: antenatal care settings<br>115. Interviewer [MS]: Mhmm<br>116. Interviewer [MS]: and Do you think it would be useful<br>117. Interviewee [XXX]: most useful *overlap*			
119. Interviewee [XXX]: Yeah, you can use it without internet electricity	Feature of the tool: can run without electricity or internet		
134. Interviewee [XXX]: No	No encounter of memory loss associated with antibiotic use in pregnant women	Memory Loss	**[7_SIDE EFFECTS]**
40. Interviewee [XXX]: eh I think I have airtime<br>41. Interviewer [MS]: okay so if you get an airtime card, you can submit it to *name of doctor* for taking part in the interview if you need to. We just have to let you know<br>42. Interviewee [XXX]: okay<br>43. Interviewer [MS]: Is that okay?	**Airtime Card**		Airtime Card

44. Interviewee [XXX]: okay<br>45. Interviewer [MS]: perfect so im just going to start with the questions now, em so so you prescribe antibiotics to pregnant women?<br>46. Interviewee [XXX]: Yes we do, I do			
47. Interviewer [MS]: Great, How long have you been prescribing them for?<br>48. Interviewee [XXX]: mmmm for up tp to 20 years now	Years of prescribing antibiotic		
49. Interviewer [MS]: Great, so how many times a week do you prescribe them would you say?<br>50. Interviewee [XXX]: I would say 2 times a week	No. of times/ week antibiotic prescribed		
